# The aquastatin biosynthetic gene cluster encodes a versatile polyketide synthase capable of synthesising heteromeric depsides with diverse alkyl side chains†

**DOI:** 10.1039/d4sc05557h

**Published:** 2024-10-24

**Authors:** Nicolau Sbaraini, Andrew Crombie, John A. Kalaitzis, Daniel Vuong, Joe Bracegirdle, Fraser Windsor, Ashli Lau, Rachel Chen, Yu Pei Tan, Alastair Lacey, Ernest Lacey, Andrew M. Piggott, Yit-Heng Chooi

**Affiliations:** a School of Molecular Sciences, The University of Western Australia Perth WA 6009 Australia nicolau.sbarainioliveira@uwa.edu.au yitheng.chooi@uwa.edu.au; b Microbial Screening Technologies Pty. Ltd Smithfield NSW 2164 Australia; c School of Natural Sciences, Macquarie University Sydney NSW 2109 Australia andrew.piggott@mq.edu.au; d Department of Agriculture and Fisheries, Plant Pathology Herbarium Dutton Park QLD 4102 Australia; e Centre for Crop Health, University of Southern Queensland Toowoomba QLD 4350 Australia

## Abstract

Depsides have garnered substantial interest due to the diverse biological activities exhibited by members of this class. Among these are the antibacterial aquastatins, glycosylated heteromeric depsides formed through the condensation of orsellinic acid with corticiolic acid. In this work, we isolated aquastatins and the recently described geministatins, along with several novel aquastatin-related depsides with different alkyl side chains from the fungus *Austroacremonium gemini* MST-FP2131. The structures were determined through comprehensive spectroscopic analysis and chemical degradation. Genome mining and heterologous expression in *Aspergillus nidulans* and *Saccharomyces cerevisiae* revealed that aquastatin biosynthesis requires only two genes: a non-reducing polyketide synthase (SAT-KS-AT-PT-ACP-TE) and a glycosyltransferase. We demonstrated that the single polyketide synthase can synthesise an acetyl-primed orsellinic acid and alkylresorcylate with various chain lengths (C14, C16, or C18) by incorporating different long-chain acyl-CoAs as starter units, and then join these as heteromeric depsides. Using chemical degradation, we generated a series of analogues and showed that several aglycone depsides exhibit antibacterial activity against *Staphylococcus aureus* and methicillin-resistant *S. aureus* (MRSA), as well as antifungal and cytotoxic activities. Interestingly, heterologous expression of the aquastatin gene cluster in *A. nidulans* produced higher levels of geministatins with Δ^15,16^ and Δ^18,19^ double bonds, which have superior bioactivities compared to the aquastatins but are only present as minor compounds in the native fungus *A. gemini*.

## Abstract 2

The *aqu* BGC from *Austroacremonium gemini* encodes a versatile polyketide synthase capable of synthesising heteromeric depsides with diverse alkyl side chains.

## Graphical abstract

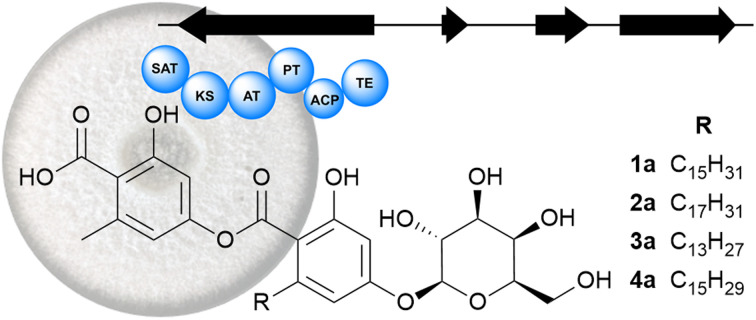

## Introduction

Depsides are a class of compounds produced by the condensation of two or more phenolic carboxylic acids, linked by ester groups. These compounds are commonly found in lichens, but higher plants and free-living fungi can also produce them.^1^ Over the years, these compounds have attracted significant scientific and commercial interest due to the intriguing bioactive properties that some of them exhibit.^1,2^ For example, aspergiside A and MS-3 possess antibacterial activity,^3,4^ while the arenicolins and lecanoric acid demonstrated antitumour activity.^5,6^ Thielavin W exhibited antifouling properties,^7^ and atranorin showcased anti-inflammatory, analgesic, and wound healing capabilities without displaying toxicity in *in vivo* assays.^8^

The biosynthetic mechanisms for depside bond formation were recently elucidated.^9–11^ The initial step in depside biosynthesis is typically catalysed by an iterative non-reducing polyketide synthase (NR-PKS).^9,10^ The resulting molecule is further modified by various enzymes such as oxygenases methyltransferases, glycosyltransferases, and others.

Aquastatin A (1a) is a glycosylated heteromeric depside that was initially isolated from *Fusarium aquaeductuum* as an inhibitor of mammalian adenosine triphosphatases.^12^ In later studies, 1a was also found to inhibit HIV-1 integrase and fatty acid synthase II (FAS II).^13,14^ This molecule, along with its aglycone, aquastatin B (1b), demonstrated antibacterial activity against *Staphylococcus aureus* and methicillin-resistant *S. aureus* (MRSA).^13^ The glycosylated phenolic monomer, aquastatin C (1c), was isolated from *Sporothrix* sp. FN611 along with 1a.^15^ Unlike 1a, 1b and 1c do not demonstrate FAS II inhibitory or antibacterial activities.^15^ Furthermore, 1a was patented in 1994 for the treatment of gastric ulcers, demonstrating its diverse therapeutic potential.^16^

Recently, we described geministatins A and B (2a and 2b) isolated from *Austroacremonium gemini* MST-FP2131.^17^ These fungal depsides are closely related to the aquastatins, with both classes consisting of an orsellinic acid unit and a glycosylated alkylresorcylic acid unit, differing only in the length and saturation of the alkyl chain. Moreover, bioactivity assays on the geministatins demonstrated the importance of an unmodified carboxylic acid for antibiotic activity and that the isolated aglycones (2b and dehydromerulinic acid A) exhibited superior bioactivity.^17^ In this study, we delved further into *A. gemini* MST-FP2131, which led to isolation of the previously described aquastatins (1a and 1b), geministatins (2a and 2b) and a suite of related molecules with different alkyl chain length and saturation. The chemodiversity of this group of compounds led us to investigate their biosynthesis, which revealed a versatile NR-PKS that can accept diverse long-chain acyl-CoAs, in addition to acetyl and malonyl-CoA, to generate a range of heteromeric depsides. The compounds were shown to exhibit varying levels of antibacterial activities, including anti-MRSA.

## Results and discussion

### Isolation and structural characterisation of heteromeric depsides from *A. gemini* MST-FP2131

**Fig. 1:**
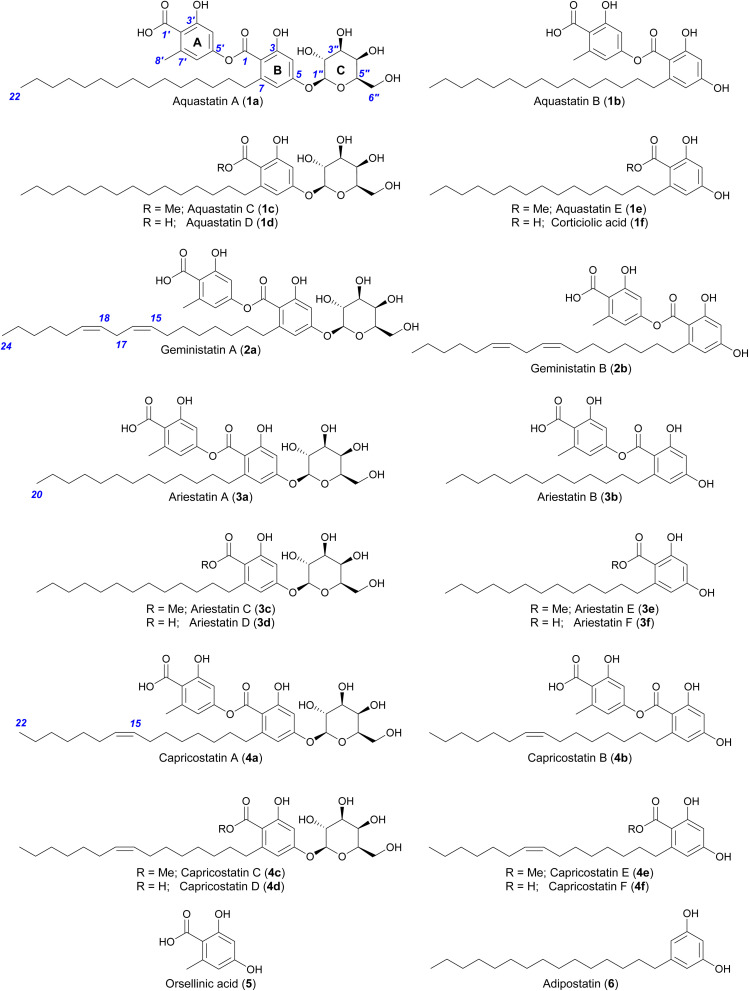
Structures of compounds described in this study. Compounds 1a–b, 2a–b, 3a and 4a were isolated from *A. gemini*, while the others were generated by chemical degradation.

Depsides related to the aquastatins were detected in the acetone extract of *A. gemini* cultivated on jasmine rice, as described previously (Fig. S1†).^17^ The evaporated acetone extract was partitioned between ethyl acetate and water, then fractionated by silica gel chromatography and purified by reversed-phase preparative HPLC (Fig. S1†) to yield the previously reported aquastatins (1a and 1b) and geministatins (2a and 2b), along with two putative novel analogues, which we named ariestatin A (3a) and capricostatin A (4a). The structures of the compounds were determined as described previously,^17^ using a combination of comprehensive ^1^H, ^13^C and 2D NMR analyses, and ozonolysis (Fig. 1). Compound 1a, the major compound in *A. gemini* (Fig. S2†), along with its aglycone (1b), have been previously described from *Fusarium aquaeductuum* as aquastatins A and B, respectively.^15^ We have also recently reported the related metabolites 2a and 2b, which are aquastatin analogues containing a longer (C_17_) unsaturated (Δ^15,16^ and Δ^18,19^) alkyl side chain.^17^

HR-ESI(−)-MS analysis of ariestatin A (3a) revealed a molecular formula C_34_H_48_O_12_, containing two carbon and four hydrogen atoms (C_2_H_4_) fewer than 1a. The ^1^H and ^13^C NMR spectra of 3a were essentially identical to those of 1a, with only differences being in the overlapping regions corresponding to the methylene envelope of the alkyl side chain. Close examination of 2D NMR data confirmed 3a is a lower homologue of 1a containing a saturated C_15_ alkyl side chain (Table S17 and Fig. S26–S31†). HR-ESI(−)-MS analysis of the structurally related capricostatin A (4a) revealed a molecular formula C_36_H_50_O_12_, containing two hydrogen atoms fewer than 1a. The ^1^H and ^13^C NMR spectra of 4a were also very similar to those of 1a, with the main difference being the presence of additional signals associated with an isolated double bond (*δ*_H_ 5.29; *δ*_C_ 129.6) in the alkyl side chain. A *Z* geometry was assigned based on the carbon chemical shifts of the methylene groups adjacent to the double bond (*δ*_C_ 26.5) with literature values for *E*- (*δ*_C_ 34.4) and *Z*- (*δ*_C_ 27.0) palmitoleic acid.^18^ Ozonolysis of 4a in MeOH^17^ confirmed the position of the double bond to be Δ^15,16^ and secured the structure of capricostatin A as 4a (Table S20 and Fig. S46–S51†).

In light of the discovery of the novel metabolites 3a and 4a from *A. gemini* as well as 1a, 1b, 2a and 2b, we set about exploring the possibility that a single biosynthetic gene cluster (BGC) encodes a versatile PKS capable of assembling all of these and possibly other minor analogues not readily observed in organic extracts of *A. gemini* cultures.

### Heterologous expression of *aqu* biosynthetic gene cluster in *Aspergillus nidulans* led to production of aquastatin-like molecules

**Fig. 2:**
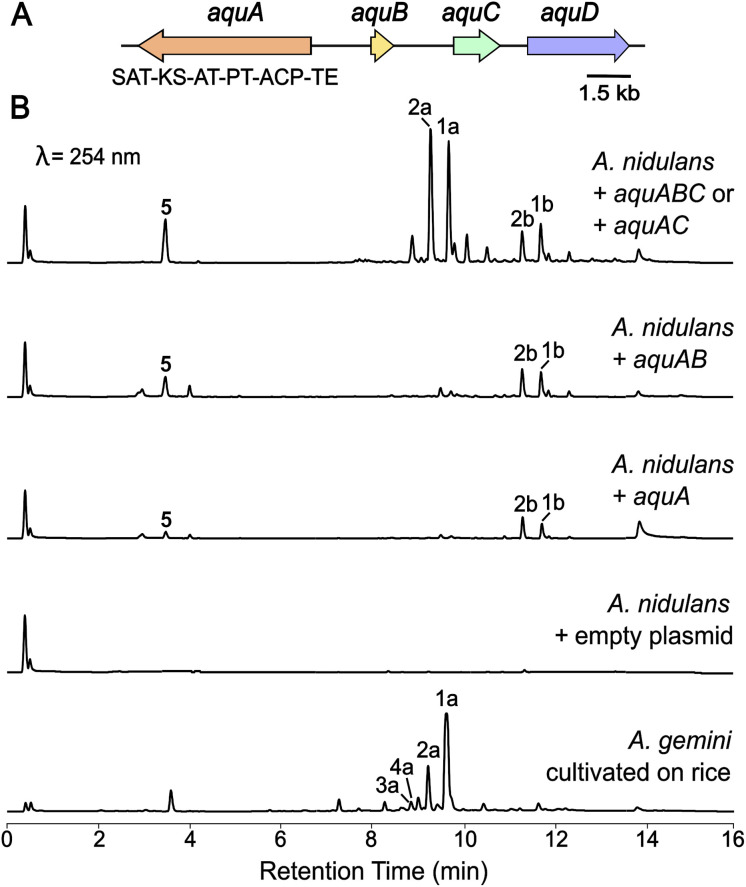
Heterologous expression of the *aqu* biosynthetic gene cluster in *A. nidulans*. (A) The *aqu* BGC in *A. gemini* is depicted. Gene names from the *aqu* cluster are indicated, as well as the backbone gene domain organisation. (B) LC-DAD (254 nm) chromatograms of *A. nidulans* cultures expressing different combinations of the *aqu* genes leading to the production of 1a, 1b, 2a and 2b as well as 5 (Fig. 1), as well as *A. gemini* MST-FP2131 grown on jasmine rice.

In our endeavour to identify the BGC involved in the biosynthesis of aquastatin-related molecules, we sequenced the genome of *A. gemini* MST-FP2131. While not all glycosylated polyketides possess integrated glycosyltransferases (GTs) within their BGCs,^19^ we oriented our search towards BGCs containing a non-reducing polyketide synthase (NR-PKS) and a GT. We located a potential gene cluster (named *aqu*) comprising four genes, which included a NR-PKS gene (named *aquA*) with the following domain organisation: starter unit acyltransferase (SAT) domain, keto-synthase (KS) domain, acyltransferase (AT) domain, product template (PT) domain, acyl carrier protein (ACP) domain, and thioesterase (TE) domain. Additionally, the cluster includes a potential resistance to 7-aminocholesterol (RTA1)-like domain-containing protein gene (named *aquB*), a potential GT from family 28 gene (named *aquC*), and a potential ABC transporter gene (named *aquD*) (Fig. 2A). Since no genetic manipulation tool or transformation method has been standardised for *A. gemini*, we decided to explore this potential gene cluster through heterologous reconstruction of the pathway in *Aspergillus nidulans*. For heterologous expression, genes *aquA*, *aquB*, and *aquC* were cloned into AMA1-based plasmids, replacing the native promoters with *alc* promoters, which can be induced using alcohols or ketones and are repressed by glucose.^20,21^ The heterologous expression of these genes in *A. nidulans* L08030 (ref. 22) led to the production of several additional compounds, which were characterised as 1a–b, 2a–b and orsellinic acid (5) (Fig. 2B). Interestingly, unlike in *A. gemini* MST-FP2131, 2a is more abundant than 1a in *A. nidulans* L08030 (Fig. 2B and S2†). Heterologous expression of *aquAB* resulted in the biosynthesis of only the aglycones 1b and 2b, with complete abolition of the glycosylated products 1a and 2a, confirming AquC as the GT responsible for attaching galactose to the depside core structure (Fig. 2B).

With the function of AquC established, we then set about determining the role of *aquB*. In the absence of *aquB*, *A. nidulans* expressing only *aquA* yielded 1b and 2b. Similarly, *A. nidulans* expressing *aquAC* resulted in the production of the major metabolites 1a and 2a. Taken together, these results suggested that AquB has no catalytic role in the formation of the heteromeric depsides and the genes *aquA* and *aquC* are sufficient to generate the glycosylated depsides 1a and 2a*via* heterologous expression in *A. nidulans*. More importantly, it suggests that the NR-PKS AquA alone can generate heteromeric depsides, incorporate different acyl-CoAs to generate an alkylresorcylic unit (ring B) and use acetyl-CoA as starter unit to generate an orsellinic acid unit (ring A; Fig. 1). Furthermore, no homomeric depsides were observed in either *A. gemini* or *A. nidulans* by LC-MS and HR-ESI-MS analysis (Tables S1 and S2†), suggesting that AquA selectively synthesises heteromeric depsides. Moreover, using extracted ion chromatograms (EIC) and comparison with standards, we could also detect 3a and 4a along with their aglycones (3b and 4b) in *A. nidulans* strains expressing the *aqu* BGC (Tables S3 and S4†).

### Heterologous expression of NR-PKS *aquA* and precursor feeding in *Saccharomyces cerevisiae* led to production of geministatin B

**Fig. 3:**
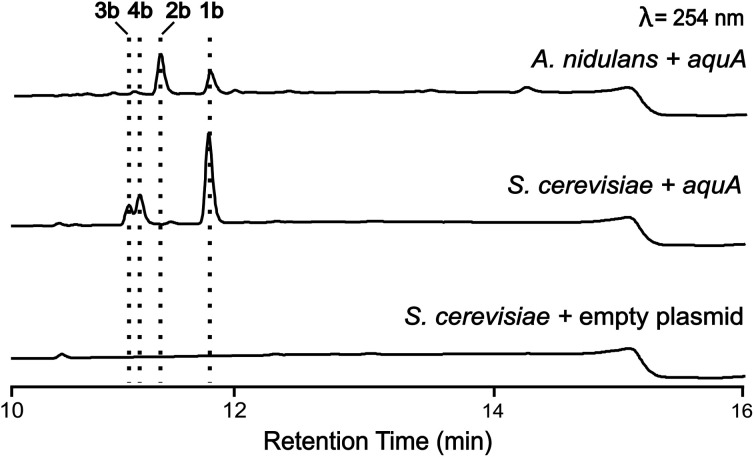
Heterologous expression of the *aquA* gene in *S. cerevisiae*. Expansions of LC-DAD (254 nm) chromatograms of *A. nidulans* and *S. cerevisiae* cultures expressing aquA. Compounds 1b and 2b can be found in *A. nidulans*, while 2b was not produced in *S. cerevisiae*.

*A. nidulans* is known to produce 5 and lecanoric acid^23^ and can encode other potential enzymes that modify such products. In order to rule out the potential effect of other enzymes in the biosynthesis of heteromeric depsides and to prove that AquA is the only enzyme required for the biosynthesis of these compounds, we expressed the *aquA* gene in *Saccharomyces cerevisiae*, which is not known to produce such aromatic polyketides. cDNA was prepared from an *A. nidulans* strain expressing *aquA*, and the intron-free gene was cloned into the plasmid pXW55 (ref. 24) to generate the plasmid pXW55::*aquA*, which was then transformed into *S. cerevisiae* BJ5464-NpgA.^25–27^ Notably, yeast cells expressing *aquA* were able to produce the heteromeric depsides 1b, 3a and 4a (Fig. 3), thus confirming no involvement of endogenous *A. nidulans* enzymes in the biosynthesis of these compounds. Like the *A. nidulans* transformants, no homomeric depsides were detected from these expression cultures through LC-MS and HR-ESI-MS analysis (Table S1†), confirming that the biosynthetic products of AquA are solely heteromeric depsides.

**Fig. 4:**
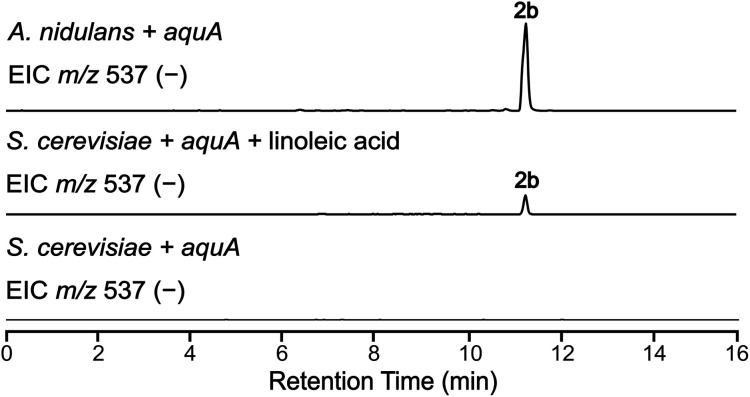
Feeding linoleic acid to *S. cerevisiae*. EIC(−)MS of *S. cerevisiae* expressing *aquA*, both with and without linoleic acid feeding, compared to *A. nidulans*.

The absence of 2b in the above expression (Fig. 3) led us to investigate the assembly of the geministatins, which are related metabolites possessing an unsaturated (Δ^15,16^ and Δ^18,19^) alkyl side chain. We hypothesised that the alkyl side chain observed in the geministatins could be derived from linoleic acid (C18:2) given its abundance in *A. nidulans*.^28^ Moreover, *S. cerevisiae*, was previously shown to be incapable of synthesising polyunsaturated fatty acids,^29^ suggesting that the absence of 2b in *S. cerevisiae* expressing *aquA* could be due to the non-availability of the proposed linoleic acid precursor. To investigate this hypothesis, we carried out feeding assays by supplementing the yeast culture medium with linoleic acid. As predicted, this led to an accumulation of 2b (Fig. 4). This outcome suggested that linoleic acid provided to the yeast can be converted into linoleoyl-CoA, which AquA subsequently utilises as a biosynthetic building block to produce the geministatins.

Based on this result, we deduced that the alkyl chain of aquastatins is likely derived from palmitic acid/palmitoyl-CoA (C16:1), while the alkyl chains of 3a and 4a are likely derived from myristic acid/myristoyl-CoA (C14:0) and palmitoleic acid/palmitoleoyl-CoA (C16:1), respectively. Combining the current knowledge of depside biosynthesis with the results obtained above, we postulated possible mechanisms for the biosynthesis of aquastatin-related depsides, which involved incorporation of available long-chain fatty acyl-CoAs that are formed through enzymes encoded by genes that are not clustered within the *aqu* BGC. The varying abundance of 1a/1b and 2a/2b between *A. gemini* MST-FP2131, *A. nidulans* L08030, and *S. cerevisiae* BJ5464-NpgA suggested that the difference could be attributed to the efficiency of the acyl-CoA ligases in converting various long chain fatty acids to corresponding acyl-CoAs.

NR-PKSs are known to incorporate different acyl-CoAs besides acetyl-CoA. For instance, the norsolorinic acid synthase (PksA) incorporates a hexanoyl-CoA starter unit *via* the starter-unit acyltransferase (SAT) domain.^30^ For depside biosynthesis, Wei *et al.*^9^ initially demonstrated the thioesterase (TE) domain of AN7909 (OrsA) catalysed depside bond formation to generate lecanoric acid and subsequent Smiles rearrangement to form the diaryl ether, diorcinolic acid. More recently, the TE domain of ThiA was shown to be solely responsible for depside bond formation in the biosynthesis of the trimeric depside, thielavin.^31^ On the other hand, Chen *et al.*^10^ demonstrated that in the duricamidepside biosynthetic pathway, the DrcA SAT domain, instead of TE, is responsible for the depside bond formation. Subsequently, Liu *et al.*^11^ used domain swapping and fusions to demonstrate that the PKS Preu6 requires SAT and TE domain–domain interactions to form the depside bond in lecanoric acid biosynthesis.

**Fig. 5:**
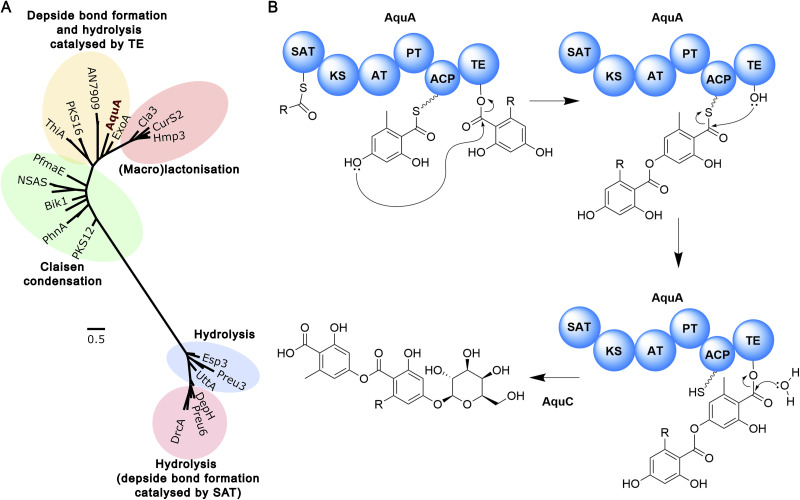
The biosynthesis of aquastatin-related heteromeric depsides. (A) Phylogenetic analysis of TE domain sequences from selected fungal NR-PKSs. TE domain sequences from depside-forming NR-PKSs that utilise the SAT domain for depside bond formation are distantly related to NR-PKSs that employ the TE domain for this purpose. TE-releasing mechanisms are indicated. A more extensive phylogenetic analysis is presented in Fig. S119†. (B) The proposed biosynthetic pathway to the aquastatins.

To gain further insights into the potential mechanism of heteromeric depside formation by AquA, we used phylogenetic analysis to explore the evolutionary proximity of the SAT and TE domains to characterised domains from NR-PKSs producing diorcinolic acid, lecanoric acid, thielavin, and duricamidepside. In the characterisation of thielavin biosynthesis, Ji *et al.*^31^ demonstrated through phylogenetic analysis that the TE domains of ThiA and AN7909 group together, while the TE domains of depside-forming NR-PKSs that use the SAT domain for depside bond formation are distantly related to ThiA/AN7909. Additionally, phylogenetic analyses have been used to propose the potential depside bond formation mechanism in the biosynthesis of exophilic acid.^32^ Interestingly, for both AquA domains, a similar phylogenetic distribution was revealed where the domains of depside-forming NR-PKSs were grouped into two distantly related clades based on whether the depside bond formation involved SAT or TE (Fig. 5B, S118 and S119†). One group includes depside-forming PKS enzymes AN7909 (lecanoric/diorinolic acid), ThiA (thielavin), GyrPKS (gyrophoric acid), ExoA (exophilic acid) as well as aquastatin (AquA), where the TE domains of AN7909 and ThiA have been shown to catalyse depside bond formation (Fig. 5B, S118 and S119†). The other group includes DrcA (duricamidepside) and Preu6 (lecanoric acid), where the SAT domains have been shown to be essential for depside bond formation. This group also includes depside-forming NR-PKSs Atr1 (atranorin), MollE (mollicelin), and DepH (nidulin) (Fig. 5A, S118 and S119†). Moreover, both catalytic residues of the depside bond-forming TE domain identified in ThiA (Ser1937 and His2100) are also conserved in the AquA TE domain.^31^ In particular, His2100 is only conserved in enzymes where the TE domain is believed to catalyse depside bond formation (Fig. S120†).

To explore the function of TE and SAT domains of AquA *in vivo*, we performed domain deletions on *aquA*. Heterologous expression of *aquA*-Δ*TE* in *S. cerevisiae* completely abolished compound production, with neither the depside product 1b nor the monomers 1f, and 5 detected. This is consistent with the phylogenetic analysis, suggesting that the TE domain is not only involved in depside bond formation, but also in the hydrolysis of the nascent compound (Fig. S121†). Similarly, no compounds were detected in strains expressing *aquA*-Δ*SAT* (Fig. S121†), suggesting that the SAT domain is also required for biosynthesis of 1b. As such, we propose that the SAT domain can load the fatty acyl-CoA as starter unit to the ACP for synthesis of the alkylresorcylic acid unit, while acting as a gatekeeper for selecting a specific range of substrates. Based on these results, we propose the biosynthetic mechanism for heteromeric depside biosynthesis by AquA (Fig. 5B). Additional *in vitro* experiments using recombinant proteins are required to shed light on how AquA controls the alternating starter unit selectivity and order to yield the two different aromatic monomers.

Recently, Yang *et al.*^33^ described how DepH synthesises both heteromeric and homomeric depsides in the biosynthesis of nornidulin and emeguisin A.^33^ However, in this pathway, heteromeric depside biosynthesis requires an auxiliary highly-reducing (HR) PKS, DepD. In the absence of this auxiliary HR-PKS, the NR-PKS DepH produces lecanoric acid.^33^ In the case of AquA, it appears that the single NR-PKS can incorporate different long chain acyl-CoAs as starter units to generate an alkylresorcylic unit (ring B) and can also use acetyl-CoA as an alternative starter unit to generate an orsellinic acid unit (ring A), and assemble the two phenolic units in a specific order *via* an ester bond to form a heteromeric depside selectively. To the best of our knowledge, such an NR-PKS is unprecedented.

### MS/MS-based molecular networking and fragment ion search uncovered an even greater diversity of heteromeric depsides

Given that AquA can utilise C14, C16, and C18 substrates (saturated or unsaturated), as evidenced by the production of 1a, 2a, 3a, and 4a, we next explored the extent of this substrate promiscuity using HR-LC-ESI-Orbitrap-MS. Our hypothesis was that other minor compounds with modifications in the alkyl side chain might also be present. Using the Molecular Networking tool in the Thermo Fisher Compound Discoverer software (Fig. S3†) and analysis of the MS/MS spectra, several potential analogues were identified. A characteristic diagnostic fingerprint pattern emerged when we examined the MS/MS spectra of 1a–4a, where the parent ions were fragmented into the decarboxylated ions (−44 u), aglycone ions (1b–4b; −162 u), the glycosylated alkylresorcylate ions (1c–4c; −150 u) and the orsellinate ion (5; *m*/*z* 167.0350 [M–H]^−^) (Fig. S5–S8†). Common to the 1a–4a MS/MS spectra are the [M–H]^−^ ions further fragmented from 5 (*m*/*z* 105.0346, 123.0451, and 149.0244) (Fig. S4†), which are also common MS/MS fragments observed for lichen depsides in a previous study.^34^ This characteristic fragmentation pattern helped to locate several orsellinate-containing depside peaks and to identify other possible structural variations present in the alkyl side chains.

Using the above methods, we uncovered a range of putative aquastatin-like heteromeric depsides with alkyl chain lengths ranging from C14 to C18 (Fig. S9–S13†). Interestingly, molecules with alkyl side chain lengths shorter than C14 were not detected by LC-MS and HR-ESI-MS analysis, indicating that myristoyl-CoA is potentially the smallest substrate that can be accepted (Tables S3 and S4†). Additionally, glycosylated heteromeric depsides with structures potentially similar to 2a (linoleoyl-derived), but containing oxygenated unsaturated alkyl chains, were also identified (Fig. S9 and S10†). Compounds 7a (*m*/*z* 715.3330 [M–H]^−^; C_38_H_52_O_13_) and 8a (*m*/*z* 731.3278 [M–H]^−^; C_38_H_52_O_14_) are potentially analogues of 2a with monooxygenated and dioxygenated unsaturated alkyl side chains, respectively (Fig. S9 and S10†). Oxygenated saturated alkyl side chain compounds were also located, including 9a (*m*/*z* 691.3332 [M–H]^−^; C_36_H_52_O_13_), potentially derived from palmitoyl-CoA (C16:0), and 10a (*m*/*z* 719.3643 [M–H]^−^; C_38_H_56_O_13_), potentially derived from stearoyl-CoA (C18:0) (Fig. S11 and S12†). The modifications in these molecules could mirror the oxygenation seen in oxylipins previously identified in *Aspergillus* spp., which are known to regulate fungal branching and differentiation.^35^ Moreover, a compound potentially featuring an alkyl side chain derived from linolenic acid (C18:3) was also discernible (11a; *m*/*z* 697.3224 [M−H]^−^; C_38_H_50_O_12_) (Fig. S13†).

It is also noteworthy that several alkylresorcylic acids with variable alkyl side chain lengths, and their corresponding methylated derivatives, were also detected in *A. nidulans* expressing *aqu* BGC and *S. cerevisiae* expressing *aquA* (Tables S4–S7 and S9†). These monomers could be generated by abortive cycles of AquA or as degradation products, and could be further modified by host enzymes, such as *O*-methyltransferases or orsellinate decarboxylase^36^ (to give rise to 6 observed in *A. gemini*).

### Chemical degradation and bioassays reveal the structure–activity relationships of aquastatin-like depsides

The impressive array of bioactivities previously reported for the aquastatins and geministatins,^12–14,16^ as well as for other depsides,^37^ and the detection of a range of putative analogues in our MS/MS analysis above, prompted us to generate additional semisynthetic analogues of 1a–4a*via* chemical degradation to generate a structure–activity relationship for these molecules. This led to production of the corresponding heteromeric depsides (3b and 4b), glycosylated alkylresorcylic acids (1d–4d) and their methyl ester derivatives (1c–4c), as well as the alkylresorcylic acid monomers (1f–4f) and their methyl esters (1e–4e). The structures of the semisynthetic analogues of aquastatins (1c–1f), ariestatins (3b–3f), and capricostatins (4b–4f) were all confirmed using extensive NMR analysis (Tables S15–S19, S21–S25, and Fig. S18–S25, S32–S45 and S52–S81†) and HRMS measurements (Fig. S84–S87, S89–S92 and S94–S98†).

We evaluated all the isolated parent molecules and semisynthetic derivatives in bioassays against the Gram-positive bacteria *Bacillus subtilis*, *S. aureus*, and MRSA, the yeast *S. cerevisiae*, mouse myeloma NS-1, and neonatal foreskin fibroblast (NFF) mammalian cell lines (Tables 1 and S13†). Geministatins 2a and 2b have previously displayed antibiotic activity against *B. subtilis*, *S. aureus*, and MRSA, and our results here show that 2a and 2b have superior antibacterial activities when compared to the aquastatin depsides (1a and 1b), but with similar cytotoxicity (Table 1). Interestingly, ariestatin (3a and 3b) and capricostatin (4a and 4b) depsides also displayed antibiotic activities. Similar to geministatins, where the aglycone (2b) exhibited increased bioactivity compared to the glycosylated form (2a), aquastatin B (1b), ariestatin B (3b), and capricostatin B (4b) also displayed stronger inhibitory activities in bioassays than their glycosylated forms (Table 1). In particular, the capricostatin aglycone (4b) displayed stronger cytotoxic activity, showing a half-maximal inhibitory concentration (IC_50_) of 0.05 μM after 96 h against mouse myeloma NS-1 cells (Table 1). These results are similar to those of the related 4f, which also displayed a similar MIC against the same cell line. Interestingly, while 3c did not display antibiotic activity, cytotoxicity was observed against NS-1 cells, while no activity was detected against NFF cells (Table 1). Previously, we reported that geministatin D (the glycosylated alkylresorcylic acid derivative) displayed antifungal activity against *S. cerevisiae*. Notably, similar molecules 1d, 3d, and 4d exhibited even better antifungal activity than that reported for geministatin D (Table 1). Moreover, these molecules did not exhibit cytotoxicity against either of the mammalian cell lines tested.

**Table 1:** Bioassay results for compounds investigated in this study^a^

Compound	Minimum inhibitory concentration (MIC; μg mL^−1^)				IC_50_ (μM)	
	*B. subtilis*	*S. aureus*	MRSA	*S. cerevisiae*	NS-1	NFF
Aquastatin A (1a)	1.6	12.5	1.6	>200	>100	>100
Aquastatin B (1b)	1.6	>100	>50	>200	>50	>100
Aquastatin D (1d)	3.1	12.5	6.3	3.1	>50	>100
Geministatin A (2a)*	1.6	6.3	6.3	>200	>50	>100
Geministatin B (2b)*	0.4	3.1	1.6	>200	>50	>100
Ariestatin A (3a)	1.6	3.1	6.3	>200	>100	>100
Ariestatin B (3b)	1.6	12.5	12.5	>200	>50	>50
Ariestatin C (3c)	>100	>100	>100	>200	0.2	>100
Ariestatin D (3d)	25	>50	>50	3.1	>50	>50
Capricostatin A (4a)	1.6	3.1	6.3	>200	>100	>100
Capricostatin B (4b)	1.6	12.5	12.5	>200	0.05	>100
Capricostatin D (4d)	25	>50	>50	3.1	>50	>100
Capricostatin F (4f)	0.8	6.3	6.3	>200	0.05	>100
Controls	6.3	3.1	>100	3.1	1.7	1.7

a Controls: *B. subtilis* ATCC 6633 = tetracycline; *S. aureus* ATCC 25923 and MRSA ATCC 33592 = ampicillin; *S. cerevisiae* ATCC 9763 = blasticidin S HCl; NS-1 ATCC TIB-18 and NFF TCC PCS-201 = sparsomycin. * Results from Crombie *et al.*^17^

In summary, our results suggest that molecules derived from the *aqu* BGC are attractive targets for further therapeutic investigation, with a myriad of activities. Moreover, our findings demonstrate how modifications in the alkyl side chain and glycosylation can significantly affect the bioactivity of such compounds.

## Conclusions

Naturally occurring depsides have garnered significant scientific and commercial interest due to their diverse biological activities, and unsurprisingly, the biosynthetic pathways to these compounds have also attracted considerable recent attention. Here, we have investigated the biosynthetic pathway to the heteromeric fungal depside, aquastatin A. Through compound isolation, heterologous expression, and feeding assays, we have demonstrated the versatility of AquA, a single PKS that can generate a series of heteromeric depsides consisting of orsellinate and alkylresorcylate subunits with variable alkyl side chain lengths. Future experiments could investigate how the PKS selectively couples alkylresorcylic acids with orsellinate in a specific order, without generating any homomeric depsides, and how the PKS utilises an acetyl-CoA starter unit to generate the orsellinate while utilising a range of fatty acyl-CoAs to generate diverse alkylresorcylic acids. Furthermore, bioassays have highlighted the potential of these compounds as antibacterial agents against *S. aureus*, including MRSA, and demonstrated the antifungal and cytotoxic effects of selected molecules. Given their demonstrated bioactivity efficacy, aquastatin-like molecules could serve as a promising foundation for future modifications. The biosynthesis knowledge uncovered here could lead to biosynthetic approaches to generate novel analogues, which may facilitate the development of novel anti-infectives and anticancer compounds.

## Data availability

All experimental procedures, and additional experimental and characterisation data are available in the ESI.† The DNA sequence of the *aqu* BGC has been deposited in NCBI GenBank with the accession number PQ095580.

## Author contributions

Conceptualisation – NS, EL, AMP, and YHC; investigation – NS, AC, JAK, DV, JB, FW, AL, RC, and YPT; data curation – NS, AC, and JAK; formal analysis – NS, AC, JAK, AMP, and YHC; funding acquisition – AL, EL, AMP, and YHC; supervision – EL, AMP, and YHC; writing – original draft – NS, AC, JAK, and YHC; writing – review & editing – EL, AMP, and YHC.

## Conflicts of interest

The authors declare that the research was conducted in the absence of any commercial or financial relationships that could be construed as a potential conflict of interest.

## Supplementary Material

SC-OLF-D4SC05557H-s001
