# Supplementary material for: The aquastatin biosynthetic gene cluster encodes a versatile polyketide synthase capable of synthesising heteromeric depsides with diverse alkyl side chains
Source: Chem Sci. 2024 Oct 24;15(45):18872–80. doi: 10.1039/d4sc05557h (PMC11514314; doi:10.1039/d4sc05557h)
Supplement: SC-015-D4SC05557H-s001 [file SC-015-D4SC05557H-s001.pdf]

Supplementary Information (SI) for Chemical Science.
This journal is © The Royal Society of Chemistry 2024

SUPPORTING INFORMATION

The aquastatin biosynthetic gene cluster encodes a versatile polyketide synthase capable of synthesising heteromeric depsides with diverse alkyl side chains

Nicolau Sbaraini [a*], Andrew Crombie [b], John A. Kalaitzis [c], Daniel Vuong [b], Joe Bracegirdle [a,b], Fraser Windsor [a], Ashli Lau [a], Rachel Chen [b], Alastair Lacey [b], Yu Pei Tan [d,e], Ernest Lacey [b,c], Andrew M. Piggott [c*], Yit-Heng Chooi [a*]

[a] *School of Molecular Sciences, The University of Western Australia, Perth, WA 6009, Australia. Email:*
[b] *Microbial Screening Technologies Pty. Ltd., Smithfield, NSW 2164, Australia.*
[c] *School of Natural Sciences, Macquarie University, Sydney, NSW 2109, Australia.*
[d] *Department of Agriculture and Fisheries, Plant Pathology Herbarium, Dutton Park, QLD 4102, Australia.*
[e] *Centre for Crop Health, University of Southern Queensland, Toowoomba, QLD 4350, Australia.*

Table of Contents

Experimental Procedures 6

1. Strains, strain maintenance, and transformation conditions. 6
2. Isolation and purification of compounds from *A. gemini* MST-FP2131. 6
3. Chemical degradation studies. 7
4. Compound Characterisation. 9
5. *A. nidulans* metabolite profiles analysis. 11
6. Nucleic acid extraction and genome assembly and annotation. 12
7. RNA extraction and cDNA synthesis. 12
8. Genome mining and heterologous expression of *aqu* cluster in *A. nidulans* and *S. cerevisiae*. 13
9. *S. cerevisiae* BJ5464-NpgA culturing and feeding assay. 13
10. HR-ESI-MS and MS-based molecular networking analysis. 14
11. Phylogenetic analysis. 15
12. Bioactivity screening. 15

Supplementary Tables 17

Supplementary Figures 37

* Corresponding authors:
NS, nicolau.sbarainioliveira@uwa.edu.au; AMP, andrew.piggott@mq.edu.au; YHC, yitheng.chooi@uwa.edu.au

References......140

Supplementary Tables

Table S1. Presence or absence of homomeric depside aglycone molecules in extracts derived from *A. nidulans*, *S. cerevisiae* and *A. gemini*. The presence of compounds was examined using LC-MS and HR-ESI-MS analysis. 17
Table S2. Presence or absence of glycosylated homomeric depside molecules in extracts derived from *A. nidulans* and *A. gemini*. The presence of compounds was examined using LC-MS and HR-ESI-MS analysis. 17
Table S3. Presence or absence of potential glycosylated heteromeric depside molecules in extracts derived from *A. nidulans* expressing all genes from the *aqu* BGC. The presence of compounds was examined using LC-MS and HR-ESI-MS analysis. 18
Table S4. Presence or absence of potential heteromeric depside aglycone molecules in extracts derived from *A. nidulans* expressing all genes from the *aqu* BGC. The presence of compounds was examined using LC-MS and HR-ESI-MS analysis. 19
Table S5. Presence or absence of *O*-methylated and glycosylated alkylresorcylic acid molecules in extracts derived from *A. nidulans* expressing all genes from the *aqu* BGC. 19
Table S6. Presence or absence of glycosylated alkylresorcylic acid molecules in extracts derived from *A. nidulans* expressing all genes from the *aqu* BGC. 20
Table S7. Presence or absence of *O*-methylated alkylresorcylic acid molecules in extracts derived from *A. nidulans* expressing all genes from the *aqu* BGC. 20
Table S8. Presence or absence of alkylresorcylic acid molecules in extracts derived from *A. nidulans* expressing all genes from the *aqu* BGC. 20
Table S9. Presence or absence of alkylresorcylic acid molecules and orsellinic acid in extracts derived from *S. cerevisiae* expressing *aquA* 21
Table S10. Vectors used in this study. 22
Table S11. Strains built in this study for heterologous expression. 22
Table S12. Oligonucleotides used in this study. 23
Table S13. Additional bioassay results for compounds investigated in this study 24
Table S14. ^{1}H (600 MHz) and ^{13}C (150 MHz) NMR data for **1a** and **1b** in DMSO-d_6 25
Table S15. ^{1}H (600 MHz) and ^{13}C (150 MHz) NMR data for **1c** and **1d** in DMSO-d_6 26
Table S16. ^{1}H (600 MHz) and ^{13}C (150 MHz) NMR data for **1e** and **1f** in DMSO-d_6 27
Table S17. ^{1}H (600 MHz) and ^{13}C (150 MHz) NMR data for **3a** and **3b** in DMSO-d_6 28
Table S18. ^{1}H (600 MHz) and ^{13}C (150 MHz) NMR data for **3c** and **3d** in DMSO-d_6 29
Table S19. ^{1}H (600 MHz) and ^{13}C (150 MHz) NMR data for **3e** and **3f** in DMSO-d_6 30
Table S20. ^{1}H (600 MHz) and ^{13}C (150 MHz) NMR data for capricostatin A (**4a**) in DMSO-d_6 31
Table S21. ^{1}H (600 MHz) and ^{13}C (150 MHz) NMR data for capricostatin B (**4b**) in DMSO-d_6 32
Table S22. ^{1}H (600 MHz) and ^{13}C (150 MHz) NMR data for capricostatin C (**4c**) in DMSO-d_6 33
Table S23. ^{1}H (600 MHz) and ^{13}C (150 MHz) NMR data for capricostatin D (**4d**) in DMSO-d_6 34
Table S24. ^{1}H (600 MHz) and ^{13}C (150 MHz) NMR data for capricostatin E (**4e**) in DMSO-d_6 35
Table S25. ^{1}H (600 MHz) and ^{13}C (150 MHz) NMR data for capricostatin F (**4f**) in DMSO-d_6 36

Supplementary Figures

Figure S1. Fractionation scheme for the isolation of metabolites from *A. gemini* MST-FP2131 38
Figure S2. Comparison of the metabolic profiles of *A. nidulans* and *A. gemini* 39
Figure S3. MS-based molecular networking analysis. 40
Figure S4. HRESI(−)MS/MS fragmentation pattern of orsellinic acid (**5**). 41
Figure S5. HRESI(−)MS/MS fragmentation pattern of aquastatin A (**1a**). 42
Figure S6. HRESI(−)MS/MS fragmentation pattern of geministatin A (**2a**). 43
Figure S7. HRESI(−)MS/MS fragmentation pattern of ariestatin A (**3a**). 44
Figure S8. HRESI(−)MS/MS fragmentation pattern of capricostatin A (**4a**). 45
Figure S9. HRESI(−)MS/MS fragmentation pattern of **7a**. 46
Figure S10. HRESI(−)MS/MS fragmentation pattern of **8a**. 47
Figure S11. HRESI(−)MS/MS fragmentation pattern of **9a.** 48
Figure S12. HRESI(−)MS/MS fragmentation pattern of **10a**. 49
Figure S13. HRESI(−)MS/MS fragmentation pattern of **11a**. 50
Figure S14. ^{1}H NMR spectrum (600 MHz) of aquastatin A (**1a**) in DMSO-d_6 51
Figure S15. ^{13}C NMR spectrum (150 MHz) of aquastatin A (**1a**) in DMSO-d_6 52
Figure S16. ^{1}H NMR spectrum (600 MHz) of aquastatin B (**1b**) in DMSO-d_6 53
Figure S17. ^{13}C NMR spectrum (150 MHz) of aquastatin B (**1b**) in DMSO-d_6 54
Figure S18. ^{1}H NMR spectrum (600 MHz) of aquastatin C (**1c**) in DMSO-d_6 55
Figure S19. ^{13}C NMR spectrum (150 MHz) of aquastatin C (**1c**) in DMSO-d_6 56
Figure S20. ^{1}H NMR spectrum (600 MHz) of aquastatin D (**1d**) in DMSO-d_6 57
Figure S21. ^{13}C NMR spectrum (150 MHz) of aquastatin D (**1d**) in DMSO-d_6 58
Figure S22. ^{1}H NMR spectrum (600 MHz) of aquastatin E (**1e**) in DMSO-d_6 59
Figure S23. ^{13}C NMR spectrum (150 MHz) of aquastatin E (**1e**) in DMSO-d_6 60
Figure S24. ^{1}H NMR spectrum (600 MHz) of corticiolic acid (**1f**) in DMSO-d_6 61
Figure S25. ^{13}C NMR spectrum (150 MHz) of corticiolic acid (**1f**) in DMSO-d_6 62
Figure S26. ^{1}H NMR spectrum (600 MHz) of ariestatin A (**3a**) in DMSO-d_6 63
Figure S27. ^{13}C NMR spectrum (150 MHz) of ariestatin A (**3a**) in DMSO-d_6 64
Figure S28. ^{1}H-^{13}C HSQC NMR spectrum (600 MHz) of ariestatin A (**3a**) in DMSO-d_6 65
Figure S29. ^{1}H-^{13}C HMBC NMR spectrum (600 MHz) of ariestatin A (**3a**) in DMSO-d_6 66
Figure S30. COSY NMR spectrum (600 MHz) of ariestatin A (**3a**) in DMSO-d_6 67
Figure S31. ROESY NMR spectrum (150 MHz) of ariestatin A (**3a**) in DMSO-d_6 68
Figure S32. ^{1}H NMR spectrum (600 MHz) of ariestatin B (**3b**) in DMSO-d_6 69
Figure S33. ^{13}C NMR spectrum (150 MHz) of ariestatin B (**3b**) in DMSO-d_6 70
Figure S34. ^{1}H-^{13}C HSQC NMR spectrum (600 MHz) of ariestatin B (**3b**) in DMSO-d_6 71
Figure S35. ^{1}H-^{13}C HMBC NMR spectrum (600 MHz) of ariestatin B (**3b**) in DMSO-d_6 72
Figure S36. COSY NMR spectrum (600 MHz) of ariestatin B (**3b**) in DMSO-d_6 73
Figure S37. ROESY NMR spectrum (150 MHz) of ariestatin B (**3b**) in DMSO-d_6 74
Figure S38. ^{1}H NMR spectrum (600 MHz) of ariestatin C (**3c**) in DMSO-d_6 75
Figure S39. ^{13}C NMR spectrum (150 MHz) of ariestatin C (**3c**) in DMSO-d_6 76
Figure S40. ^{1}H NMR spectrum (600 MHz) of ariestatin D (**3d**) in DMSO-d_6 77
Figure S41. ^{13}C NMR spectrum (150 MHz) of ariestatin D (**3d**) in DMSO-d_6 78
Figure S42. ^{1}H NMR spectrum (600 MHz) of ariestatin E (**3e**) in DMSO-d_6 79
Figure S43. ^{13}C NMR spectrum (150 MHz) of ariestatin E (**3e**) in DMSO-d_6 80
Figure S44. ^{1}H NMR spectrum (600 MHz) of ariestatin F (**3f**) in DMSO-d_6 81
Figure S45. ^{13}C NMR spectrum (150 MHz) of ariestatin F (**3f**) in DMSO-d_6 82
Figure S46. ^{1}H NMR spectrum (600 MHz) of capricostatin A (**4a**) in DMSO-d_6 83
Figure S47. ^{13}C NMR spectrum (150 MHz) of capricostatin A (**4a**) in DMSO-d_6 84
Figure S48. ^{1}H-^{13}C HSQC NMR spectrum (600 MHz) of capricostatin A (**4a**) in DMSO-d_6 85
Figure S49. ^{1}H-^{13}C HMBC NMR spectrum (600 MHz) of capricostatin A (**4a**) in DMSO-d_6 86
Figure S50. COSY NMR spectrum (600 MHz) of capricostatin A (**4a**) in DMSO-d_6 87
Figure S51. ROESY NMR spectrum (150 MHz) of capricostatin A (**4a**) in DMSO-d_6 88
Figure S52. ^{1}H NMR spectrum (600 MHz) of capricostatin B (**4b**) in DMSO-d_6 89
Figure S53. ^{13}C NMR spectrum (150 MHz) of capricostatin B (**4b**) in DMSO-d_6 90
Figure S54. ^{1}H-^{13}C HSQC NMR spectrum (600 MHz) of capricostatin B (**4b**) in DMSO-d_6 91
Figure S55. ^{1}H-^{13}C HSQC NMR spectrum (600 MHz) of capricostatin B (**4b**) in DMSO-d_6 92
Figure S56. COSY NMR spectrum (600 MHz) of capricostatin B (**4b**) in DMSO-d_6 93

Figure S57. ROESY NMR spectrum (600 MHz) of capricostatin B (**4b**) in DMSO-d_6 94
Figure S58. ^{1}H NMR spectrum (600 MHz) of capricostatin C (**4c**) in DMSO-d_6 95
Figure S59. ^{13}C NMR spectrum (150 MHz) of capricostatin C (**4c**) in DMSO-d_6 96
Figure S60. ^{1}H-^{13}C HSQC NMR spectrum (600 MHz) of capricostatin C (**4c**) in DMSO-d_6 97
Figure S61. ^{1}H-^{13}C HSQC NMR spectrum (600 MHz) of capricostatin C (**4c**) in DMSO-d_6 98
Figure S62. COSY NMR spectrum (600 MHz) of capricostatin C (**4c**) in DMSO-d_6 99
Figure S63. ROESY NMR spectrum (600 MHz) of capricostatin C (**4c**) in DMSO-d_6 100
Figure S64. ^{1}H NMR spectrum (600 MHz) of capricostatin D (**4d**) in DMSO-d_6 101
Figure S65. ^{13}C NMR spectrum (150 MHz) of capricostatin D (**4d**) in DMSO-d_6 102
Figure S66. ^{1}H-^{13}C HSQC NMR spectrum (600 MHz) of capricostatin D (**4d**) in DMSO-d_6 103
Figure S67. ^{1}H-^{13}C HSQC NMR spectrum (600 MHz) of capricostatin D (**4d**) in DMSO-d_6 104
Figure S68. COSY NMR spectrum (600 MHz) of capricostatin D (**4d**) in DMSO-d_6 105
Figure S69. ROESY NMR spectrum (600 MHz) of capricostatin D (**4d**) in DMSO-d_6 106
Figure S70. ^{1}H NMR spectrum (600 MHz) of capricostatin E (**4e**) in DMSO-d_6 107
Figure S71. ^{13}C NMR spectrum (150 MHz) of capricostatin E (**4e**) in DMSO-d_6 108
Figure S72. ^{1}H-^{13}C HSQC NMR spectrum (600 MHz) of capricostatin E (**4e**) in DMSO-d_6 109
Figure S73. ^{1}H-^{13}C HSQC NMR spectrum (600 MHz) of capricostatin E (**4e**) in DMSO-d_6 110
Figure S74. COSY NMR spectrum (600 MHz) of capricostatin E (**4e**) in DMSO-d_6 111
Figure S75. ROESY NMR spectrum (600 MHz) of capricostatin E (**4e**) in DMSO-d_6 112
Figure S76. ^{1}H NMR spectrum (600 MHz) of capricostatin F (**4f**) in DMSO-d_6 113
Figure S77. ^{13}C NMR spectrum (150 MHz) of capricostatin F (**4f**) in DMSO-d_6 114
Figure S78. ^{1}H-^{13}C HSQC NMR spectrum (600 MHz) of capricostatin F (**4f**) in DMSO-d_6 115
Figure S79. ^{1}H-^{13}C HSQC NMR spectrum (600 MHz) of capricostatin F (**4f**) in DMSO-d_6 116
Figure S80. COSY NMR spectrum (600 MHz) of capricostatin F (**4f**) in DMSO-d_6 117
Figure S81. ROESY NMR spectrum (600 MHz) of capricostatin F (**4f**) in DMSO-d_6 118
Figure S82. HRESI(−) mass spectrum of aquastatin A (**1a**) 119
Figure S83. HRESI(−) mass spectrum of aquastatin B (**1b**) 119
Figure S84. HRESI(−) mass spectrum of aquastatin C (**1c**) 119
Figure S85. HRESI(−) mass spectrum of aquastatin D (**1d**) 120
Figure S86. HRESI(−) mass spectrum of aquastatin E (**1e**) 120
Figure S87. HRESI(−) mass spectrum of corticiolic acid (**1f**) 120
Figure S88. HRESI(−) mass spectrum of ariestatin A (**3a**) 121
Figure S89. HRESI(−) mass spectrum of ariestatin B (**3b**) 121
Figure S90. HRESI(−) mass spectrum of ariestatin C (**3c**) 121
Figure S91. HRESI(−) mass spectrum of ariestatin D (**3d**) 122
Figure S92. HRESI(−) mass spectrum of ariestatin F (**3f**) 122
Figure S93. HRESI(−) mass spectrum of capricostatin A (**4a**) 123
Figure S94. HRESI(−) mass spectrum of capricostatin B (**4b**) 123
Figure S95. HRESI(−) mass spectrum of capricostatin C (**4c**) 123
Figure S96. HRESI(−) mass spectrum of capricostatin D (**4d**) 124
Figure S97. HRESI(−) mass spectrum of capricostatin E (**4e**) 124
Figure S98. HRESI(−) mass spectrum of capricostatin F (**4f**) 124
Figure S99. HRESI(−) mass spectrum of capricostatin A (**4a**) ozonolysis product 125
Figure S100. UV-vis spectrum of ariestatin A (**3a**) in MeCN. 126
Figure S101. UV-vis spectrum of ariestatin B (**3b**) in MeCN. 126
Figure S102. UV-vis spectrum of ariestatin C (**3c**) in MeCN. 127
Figure S103. UV-vis spectrum of ariestatin D (**3d**) in MeCN. 127
Figure S104. UV-vis spectrum of capricostatin A (**4a**) in MeCN. 128
Figure S105. UV-vis spectrum of capricostatin B (**4b**) in MeCN. 128
Figure S106. UV-vis spectrum of capricostatin C (**4c**) in MeCN. 129
Figure S107. UV-vis spectrum of capricostatin D (**4d**) in MeCN. 129
Figure S108. UV-vis spectrum of capricostatin F (**4f**) in MeCN. 130
Figure S109. IR spectrum (ATR) of ariestatin A (**3a**). 131
Figure S110. IR spectrum (ATR) of ariestatin B (**3b**). 131
Figure S111. IR spectrum (ATR) of ariestatin C (**3c**). 132
Figure S112. IR spectrum (ATR) of ariestatin D (**3d**). 132
Figure S113. IR spectrum (ATR) of capricostatin A (**4a**). 133
Figure S114. IR spectrum (ATR) of capricostatin B (**4b**). 133
Figure S115. IR spectrum (ATR) of capricostatin C (**4c**). 134

Figure S116. IR spectrum (ATR) of capricostatin D (**4d**). 134
Figure S117. IR spectrum (ATR) of capricostatin F (**4f**). 135
Figure S118. Phylogenetic analysis of SAT domain sequences of selected fungal NR-PKSs. 136
Figure S119. Phylogenetic analysis of TE domain sequences of selected fungal NR-PKSs. 137
Figure S120. Sequence alignment of TE domain sequences of depside-forming enzymes. 138
Figure S121. Heterologous expression of truncated versions of *aquA* (Δ*TE* and Δ*SAT*) in *S. cerevisiae*. 139

Experimental Procedures

1. Strains, strain maintenance, and transformation conditions.

A. gemini MST-FP2131 has been maintained in YPD agar or Malt Extract agar (MEA) plates. *Saccharomyces cerevisiae* strain BJ5464-NpgA was employed for yeast-mediated homologous recombination cloning and heterologous expression of *aquA* gene.[1] The preparation of competent cells and transformation processes have been previously described.[1] *A. nidulans* LO8030 was used for heterologous expression.[2] The methods for protoplast preparation, transformation, and selection have been described in previous studies.[3,4] *A. nidulans* was cultured in Glucose Minimal Medium (GMM) for all experiments.[3] GMM was supplemented with riboflavin, and pyridoxine if necessary.[3] *Escherichia coli* NEB10-beta® electrocompetent cells were used to store and propagate the generated plasmids.

2. Isolation and purification of compounds from *A. gemini* MST-FP2131.

A. gemini MST-FP2131 was cultivated on sterilised (121 °C for 40 min) jasmine rice in 90 × 250 mL Erlenmeyer flasks each containing 50 g of rice. Agar squares from a 7 day culture on Petri plates were used as inoculum for the flasks. Flasks were incubated at 24 °C for 14 days and the grains were pooled and extracted with acetone (2 × 4 L). The combined extracts were reduced in vacuo to produce an aqueous slurry (1 L). The slurry was partitioned against EtOAc (2 × 4 L) and the combined EtOAc layer was reduced in vacuo to give a crude extract (42 g). The extract was redissolved in 90% MeOH/H_2O (500 mL) and defatted with hexane (2 × 500 mL) to provide an enriched extract (38 g). The enriched extract was adsorbed onto silica gel (85 g) and dry-loaded onto a silica gel column (100 g, 300 × 50 mm). The column was washed once with hexane, then eluted with 50% $CHCl_3$/hexane, 75% $CHCl_3$/hexane and 100% $CHCl_3$, followed by a stepwise gradient of 1, 2, 4, 8, 16, 32 and 100% MeOH/$CHCl_3$ (500 mL each step), to yield 11 fractions (Fr 1–11). Fr 10 (11.1 g) was purified by isocratic preparative HPLC (Zorbax C18, isocratic 60% MeCN/H_2O containing 0.1% TFA, 60 mL/min) to yield **3a** (t_R 19.19 min; 67 mg), **4a** (t_R 22.15 min; 81 mg), **2a** (t_R 28.67 min; 343 mg) and **1a** (t_R 46.89 min; 2.8 g). Fr 7 (430 mg) was purified by isocratic preparative HPLC (Zorbax C18, isocratic MeCN containing 0.1% TFA, 60 mL/min) to yield **1b** (t_R 17.82 min; 38 mg) and an enriched fraction that was further purified by isocratic preparative HPLC (Zorbax C18, isocratic 90% MeCN/H_2O containing 0.1% TFA, 20 mL/min) to yield **2b** (t_R 23.10 min; 7.9 mg). A workflow of the purification process is presented in Figure S1.

3. Chemical degradation studies.

Preparation of ariestatin B (3b). A solution of **3a** (15 mg) in 5% aqueous acetone (2 mL) was treated with 37% aqueous HCl (200 µL) and incubated at 24 °C in a sealed vial for 48 h. The reaction mixture was diluted with H_2O (200 mL) and partitioned against EtOAc (200 mL). The EtOAc was evaporated in vacuo and purified by preparative HPLC (Zorbax C18; isocratic 85% MeCN/H_2O containing 0.1% TFA, 20 mL/min) to yield **3b** (t_R 31.42 min; 2.4 mg, 16%).

Preparation of ariestatin C (3c). A solution of **3a** (15 mg) in MeOH (2 mL) was heated at 80 °C in a sealed vial for 8 h. The reaction mixture was purified by preparative HPLC (Zorbax C18, isocratic 90% MeCN/H_2O containing 0.1% TFA, 20 mL/min) to yield **3c** (t_R 9.78 min; 3.7 mg, 25%).

Preparation of ariestatin D (3d). A solution of **3a** (15 mg) in 5% aqueous acetone (2 mL) was heated at 80 °C in a sealed vial for 8 h. The reaction mixture was purified by preparative HPLC (Zorbax C18, isocratic 70% MeCN/H2O containing 0.1% TFA, 20 mL/min) to yield **3d** (t_R 8.60 min; 1.8 mg, 12%).

Preparation of ariestatin E (3e). A solution of **3b** (10 mg) in MeOH (2 mL) was heated at 80 °C in a sealed vial for 8 h. The reaction mixture was purified by preparative HPLC (Zorbax C18, isocratic 90% MeCN/H_2O containing 0.1% TFA, 20 mL/min) to yield **3e** (t_R 38.24 min; 5.5 mg, 55%).

Preparation of ariestatin F (3f). A solution of **3b** (10 mg) 5% aqueous acetone (2 mL) was heated at 80 °C in a sealed vial for 6 h. The reaction mixture was purified by preparative HPLC (Zorbax C18, isocratic 95% MeCN/H_2O containing 0.1% TFA, 20 mL/min) to yield **3f** (t_R 9.58 min; 4.2 mg, 42%).

Preparation of capricostatin B (4b). A solution of **4a** (25 mg) in 5% aqueous acetone (2 mL) was treated with 37% aqueous HCl (200 µL) and incubated at 24 °C in a sealed vial for 72 h. The reaction mixture was diluted with H_2O (200 mL) and partitioned against EtOAc (200 mL). The EtOAc was evaporated in vacuo and purified by preparative HPLC (Zorbax C18; isocratic 95% MeCN/H_2O containing 0.1% TFA, 20 mL/min) to yield **4b** (t_R 10.02 min; 20 mg, 80%).

Preparation of capricostatin C (4c). A solution of **4a** (25 mg) in MeOH (2 mL) was heated at 80 °C in a sealed vial for 24 h. The reaction mixture was purified by preparative HPLC (Zorbax C18, isocratic 87.5% MeCN/H_2O containing 0.1% TFA, 20 mL/min) to yield **4c** (t_R 12.74 min; 9.3 mg, 37%).

Preparation of capricostatin D (4d). A solution of **4a** (25 mg) in 5% aqueous acetone (2 mL) was heated at 80 °C in a sealed vial for 24 h. The reaction mixture was purified by preparative HPLC (Zorbax C18, isocratic 70% MeCN/H_2O containing 0.1% TFA, 20 mL/min) to yield **4d** (t_R 10.05 min; 3.9 mg, 16%).

Preparation of capricostatin E (4e). A solution of **4c** (15 mg) in MeOH (2 mL) was treated with 37% aqueous HCl (200 µL) and incubated at 24 °C in a sealed vial for 6 h. The reaction mixture was diluted with H_2O (200 mL) and partitioned against EtOAc (200 mL). The EtOAc was evaporated in vacuo and purified by preparative HPLC (Zorbax C18, isocratic 95% MeCN/H_2O containing 0.1% TFA, 20 mL/min) to yield **4e** (t_R 37.87 min; 5.2 mg, 35%).

Preparation of capricostatin F (4f). A solution of **4b** (15 mg) in MeOH (2 mL) was heated at 80 °C in a sealed vial for 8 h. The reaction mixture was purified by preparative HPLC (Zorbax C18, isocratic 85% MeCN/H_2O containing 0.1% TFA, 20 mL/min) to yield **4f** (t_R 19.92 min; 5.2 mg, 35%).

Ozonolysis of capricostatin A. Capricostatin A (**4a**; 30 mg) was dissolved in MeOH (10 mL) and ozone was bubbled through the solution for 1 min at a rate of 10 mg/min. The reaction mixture was purified by preparative HPLC (Zorbax C18; isocratic 40% MeCN/H_2O containing 0.1% TFA, 20 mL/min) to yield ozonolysis product (t_R 6.55 min; 4.2 mg, 14%).

4. **Compound Characterisation.**

Ariestatin A (3a). White powder; $[\alpha]_D^{24}$ –29 (*c* 0.25, MeCN); UV (MeCN) λ_{max} (log ε) 216 (4.59), 266 (4.25), 308 (3.96) nm; IR (ATR) ν_{max} 3776, 3714, 3336, 2923, 2849, 2376, 2321, 2234, 1658, 1609, 1456, 1291, 1244, 1199, 1180, 1139, 1064, 1032, 890, 848, 780, 708, 608, 589, 539, 512 cm^{-1}; ^{1}H and ^{13}C NMR see Table S17. HR-ESI(–)-MS *m/z* 647.3072; calcd for $C_{34}H_{47}O_{12}^-$ [M – H]$^-$, 647.3073.

Ariestatin B (3b). White powder; UV (MeCN) λ_{max} (log ε) 217 (4.63), 269 (4.27), 307 (4.03) nm; IR (ATR) ν_{max} 3083, 2920, 2851, 1654, 1594, 1453, 1379, 1294, 1241, 1197, 1135, 1070, 1017, 888, 829, 797, 712, 690, 606, 530 cm^{-1}; ^{1}H and ^{13}C NMR see Table S17. HR-ESI(–)-MS *m/z* 485.2544; calcd for $C_{28}H_{37}O_7^-$ [M – H]$^-$, 485.2544.

Ariestatin C (3c). White powder; $[\alpha]_D^{24}$ –29 (*c* 0.20, MeCN); UV (MeCN) λ_{max} (log ε) 217 (4.44), 259 (4.09), 303 (3.66) nm; IR (ATR) ν_{max} 3665, 3522, 3422, 3326, 2972, 2918, 2850, 1932, 1644, 1620, 1572, 1455, 1434, 1388, 1324, 1261, 1221, 1176, 1067, 957, 897, 870, 837, 784, 711, 634, 597, 544 cm^{-1}; ^{1}H and ^{13}C NMR see Table S18. HR-ESI(–)-MS *m/z* 511.2911; calcd for $C_{27}H_{43}O_9^-$ [M – H]$^-$, 511.2912.

Ariestatin D (3d). White powder; $[\alpha]_D^{24}$ –96 (*c* 0.10, MeCN); UV (MeCN) λ_{max} (log ε) 218 (4.28), 258 (3.84), 303 (3.48) nm; IR (ATR) ν_{max} 5729, 3365, 2922, 2848, 2667, 2358, 2297, 1609, 1463, 1358, 1243, 1210, 1175, 1141, 1085, 1030, 843, 789, 709, 680, 585, 544 cm^{-1}; ^{1}H and ^{13}C NMR see Table S18. HR-ESI(–)-MS *m/z* 497.2754; calcd for $C_{26}H_{41}O_9^-$ [M – H]$^-$, 497.2756.

Capricostatin A (4a). White powder; $[\alpha]_D^{24}$ –16 (*c* 0.50, MeCN); UV (MeCN) λ_{max} (log ε) 214 (4.62), 266 (4.24), 309 (3.95) nm; IR (ATR) ν_{max} 3728, 3315, 2924, 2852, 2359, 2297, 1658, 1610, 1582, 1454, 1345, 1242, 1198, 1178, 1138, 1063, 1034, 927, 886, 835, 763, 695, 612, 583, 526 cm^{-1}; ^{1}H and ^{13}C NMR see Table S20. HR-ESI(–)-MS *m/z* 673.3227; calcd for $C_{36}H_{49}O_{12}^-$ [M – H]$^-$, 673.3229.

Capricostatin B (4b). White powder; UV (MeCN) λ_{max} (log ε) 215 (4.59), 269 (4.25), 307 (4.00) nm; IR (ATR) ν_{max} 3728, 3070, 2923, 2851, 2614, 2559, 2359, 2299, 1878, 1646, 1609, 1452, 1342, 1308, 1238, 1194, 1166, 1136, 1070, 1020, 994, 899, 843, 796, 688, 603, 528 cm^{-1}; ^{1}H and ^{13}C NMR see Table S21. HR-ESI(−)-MS *m/z* 511.2697; calcd for $C_{30}H_{39}O_7^-$ [M – H]⁻, 511.2701.

Capricostatin C (4c). White powder; $[\alpha]_D^{24}$ −30 (*c* 0.50, MeCN); UV (MeCN) λ_{max} (log ε) 218 (4.46), 259 (4.11), 303 (3.69) nm; IR (ATR) ν_{max} 3662, 3608, 3357, 2923, 2854, 156, 1650, 1617, 1577, 1435, 1391, 1324, 1299, 1257, 1208, 1175, 1138, 1075, 956, 888, 852, 802, 711, 575, 539 cm^{-1}; ^{1}H and ^{13}C NMR see Table S22. HR-ESI(−)-MS *m/z* 537.3068; calcd for $C_{29}H_{45}O_9^-$ [M – H]⁻, 537.3069.

Capricostatin D (4d). White powder; $[\alpha]_D^{24}$ −31(*c* 0.20, MeCN); UV (MeCN) λ_{max} (log ε) 218 (4.43), 258 (4.02), 303 (3.62) nm; IR (ATR) ν_{max} 3729, 3595, 3365, 2925, 2850, 2665, 2359, 2296, 1612, 1461, 1357, 1245, 1213, 1175, 1141, 1086, 1031, 985, 948, 845, 782, 680, 583, 542 cm^{-1}; ^{1}H and ^{13}C NMR see Table S23. HR-ESI(−)-MS *m/z* 523.2912; calcd for $C_{28}H_{43}O_9^-$ [M – H]⁻, 523.2912.

Capricostatin F (4f). White powder; UV (MeCN) λ_{max} (log ε) 218 (4.39), 260 (3.99), 303 (3.56) nm; IR (ATR) ν_{max} 3798, 3706, 3387, 3006, 2921, 2851, 2537, 2359, 1894, 1619, 1461, 1356, 1242, 1176, 1113, 1022, 854, 802, 754, 717, 671, 619, 538 cm^{-1}; ^{1}H and ^{13}C NMR see Table S25. HR-ESI(−)-MS *m/z* 361.2401; calcd for $C_{22}H_{33}O_4^-$ [M – H]⁻, 361.2384.

5. *A. nidulans* metabolite profiles analysis.

Metabolite profile analyses of the *A. nidulans* strain LO8030, harbouring the constructed plasmids, as well as the control plasmids, were performed using an Agilent 1260 liquid chromatography (LC) system, coupled to a diode array detector (DAD) and an Agilent 6130 Quadrupole MS with an ESI source. Chromatographic separation was carried out at 40 °C, employing a Kinetex C18 column (2.6 µm, 2.1 × 100 mm; Phenomenex). For small-scale metabolic profile analyses, *A. nidulans* spores were inoculated (~10^8/L) into 50 mL of liquid GMM medium in 250 mL Erlenmeyer flasks. The cultures were incubated at 37 °C with shaking at 180 rpm for 24 h. After this period, 3 mL/L of methyl ethyl ketone was introduced to induce the expression of the *alcA* promoter. Subsequently, the cultures were incubated at 25 °C with shaking at 180 rpm for 120 h to allow for the accumulation of secondary metabolites. Experiments were conducted employing three biological replicates. The supernatant was isolated by vacuum filtration and extracted employing an organic solvent mixture containing EtOAc, MeOH, and AcOH in a ratio of 89.5:10:0.5. While mycelia were extracted using acetone. The crude extracts were dried in vacuo and re-dissolved in MeOH for LC-DAD-MS analysis. Chromatographic separation was achieved with a linear gradient of 5–95% MeCN-H_2O (0.1% (v/v) formic acid) over 10 min, followed by 95% MeCN for 3 min, with a flow rate of 0.6 mL/min. The MS data were collected in the *m/z* range of 100–1000.

To compare compound production between the *A. gemini* MST-FP2131 and *A. nidulans* transformants harbouring the *aqu* BGC, both fungi were cultured for 13 days in GMM supplemented with pyridoxine. The *A. gemini* MST-FP2131 cultures were maintained at 25 °C with shaking at 180 rpm throughout the experiment. In contrast, the *A. nidulans* cultures were maintained at 37 °C and induced as previously described, followed by 12 days at 25 °C with shaking at 180 rpm. Crude extract preparation and chromatographic separation was performed as described previously.

6. Nucleic acid extraction and genome assembly and annotation.

For genomic DNA extraction, *A. gemini* MST-FP2131 was cultivated in malt extract broth at room temperature for 7 days with gentle shaking. DNA was extracted from mycelia using the PowerSoil DNA isolation kit (Mo Bio Laboratories, Carlsbad, CA, USA). For genome sequencing, Nextera XT libraries were prepared using Illumina kits (sample prep kit, Illumina, San Diego, CA, USA) and then subjected to quality control using a Bioanalyzer 2100 (Agilent Technologies) before whole genome sequencing on the Illumina HiSeq 2000 platform with 100 bp paired-end reads. Raw sequencing reads were processed and assembled using the AAFTF pipeline.[5] BBDuk (BBTools software package, 2014, https://sourceforge.net/projects/bbmap/) was used to trim adapter sequences as well as filter reads for common contaminants and mitochondrial DNA. Reads were then assembled using SPAdes v.3.13.0.[6] Assembly scaffolds containing non-ascomycete sequences identified by sourmash,[7] low-coverage scaffolds identified by BWA[8] and SAMtools,[9] and duplicate scaffolds identified by Minimap2,[10] were removed. Gene model prediction and functional annotation was performed using the Funannotate v1.5.2 pipeline.[11]

7. RNA extraction and cDNA synthesis.

For cDNA synthesis, the *A. nidulans* LO8030 harbouring the *aqu* BGC was cultured and induced as described earlier, but maintained for only 24 h at 25 °C. The resulting fungal mass was ground into a powder in liquid nitrogen, and RNA extraction was performed using a phenol: chloroform protocol as previously described.[12] The extracted RNA was then treated with DNase I (Sigma-Aldrich, Burlington, MA, USA). Reverse transcription and cDNA synthesis were carried out using ProtoScript® II Reverse Transcriptase (New England Biolabs, Ipswich, MA, USA) with a mix of oligo-dT and random hexamer primers.

8. **Genome mining and heterologous expression of *aqu* cluster in *A. nidulans* and *S. cerevisiae.***

To identify potential gene cluster of interest in the genome of *A. gemini* MST-FP2131, it was initially analysed using antiSMASH v. 7.0.[13] The boundaries of BGCs were defined based on cblaster analysis (Fig. S56).[14] The GenBank accession number for the *aqu* BGC is available at PQ095580.

For the heterologous expression of the *aqu* BGC in *A. nidulans* LO8030, the promoters of *aquA* and *aquC* were replaced with the *aclA* promoter, while the promoter of *aquB* was replaced with the *alcS* promoter using our previously described pYFAC fungal episomal vector system.[15] *AquA* was cloned into the PacI site of the plasmid pYFAC-CH2, resulting in the creation of plasmid pYFACNS00020. *AquA* and *aquB* were cloned into the PacI and NotI sites, respectively, of the plasmid pYFAC-CH2, which led to the creation of plasmid pYFACNS00021. *AquC* was cloned into the PacI site of the plasmid pYFAC-CH3, resulting in the creation of plasmid pYFACNS00022.

For the heterologous expression of *aquA* gene and truncated versions in *S. cerevisiae* BJ5464-NpgA, intron-free fragments amplified from the generated cDNA were cloned into the SpeI and PmlI sites of the plasmid pXW55. This led to the creation of plasmid pXW55::*aquA*. The vectors, constructed strains, and primer sequences used and generated in the cloning steps are listed in Table S10–Table S12.

9. ***S. cerevisiae* BJ5464-NpgA culturing and feeding assay.**

Generated *S. cerevisiae* BJ5464-NpgA transformants expressing the *aquA* gene were screened for compound production in both liquid YPD and Drop-Out (without uracil and uridine)[4] media. For the feeding assay, a selected *S. cerevisiae* BJ5464-NpgA transformant was initially cultured in 50 mL of liquid Drop-Out medium (without uracil and uridine) in a 250 mL Erlenmeyer flask. The culture was incubated at 30 °C with shaking at 220 rpm for 48 h. Subsequently, 2 mL of the starter culture was transferred to new flasks containing 50 mL of the same liquid Drop-Out (without uracil and uridine) medium. These cultures were then incubated at 30 °C with shaking at 220 rpm for 24 h, after which they were fed with 200 µL of a 2.5 M solution of linoleic acid (Sigma-Aldrich, Burlington, MA, USA) in ethanol. Control cultures were fed with an ethanol solution only. The cultures were further incubated

for 96 h under the same conditions. Cells and supernatants were separated by centrifugation (5 min at 3220 × *g*). Crude extract preparation and chromatographic separation were performed as previously described. The experiment was conducted in triplicate.

10. HR-ESI-MS and MS-based molecular networking analysis.

High resolution metabolite profile analyses of the *A. nidulans* LO8030, harbouring the *aqu* BGC, as well as the control plasmids, were performed using a Vanquish UHPLC System, coupled to a diode array detector (DAD) and an Orbitrap Exploris 120 Quadrupole MS with an ESI source. Chromatographic separation was carried out at 25 °C, employing an InfinityLab Poroshell 120 SB-C18 column (2.7 µm, 2.1 × 100 mm; Agilent). Chromatographic separation was achieved with a linear gradient of 5–95% MeCN-H_2O (0.1% (v/v) formic acid) over 13 min, followed by 95% MeCN for 3 min, with a flow rate of 0.3 mL/min. The MS data were collected in the *m/z* range of 100–1000.

MS-based molecular networking analysis was conducted using Compound Discoverer Software (Thermo Fisher Scientific, Waltham, MA, USA) to compare a control strain (*A. nidulans* LO8030 with empty plasmids) with *A. nidulans* LO8030 expressing the *aqu* BGC. The generated network was inspected for known and unknown compounds linked to the nodes of **1a**, **2a**, **3a**, and **4a**. Subsequently, the fragmentation patterns of the identified compounds were inspected using FreeStyle Software v1.5 (Thermo Fisher Scientific, Waltham, MA, USA), with potential fragments being manually assigned.

11. Phylogenetic analysis.

Phylogenetic analyses were conducted by incorporating AquA sequences together with previously described entries.[16,17] SAT and TE domain boundaries were predicted employing the Conserved Domain Database (CDD).[18] The amino acid alignments were built using Clustal Omega, without manual curation.[19] The phylogenetic reconstruction was conducted employing PhyML 3.1 (Maximum Likelihood) with aLRT SH-like (approximate likelihood ratio test Shimodaira–Hasegawa) branch support estimation, and the best-fit evolutionary model predicted by the Smart Model Selection (SMS), implemented in the PhyML environment.[20,21]

12. Bioactivity screening.

Purified metabolites were dissolved in DMSO to create stock solutions of 10,000 µg/mL. An aliquot from each stock solution was transferred to the first well of Rows B to G on a 96-well microtitre plate and then serially diluted two-fold with DMSO across the 12 wells of the plate to achieve a 2,048-fold concentration gradient. Bioassay medium was added to an aliquot of each diluted solution to achieve a 100-fold dilution in the final bioassay. This resulted in a testing range from 100 to 0.05 µg/mL in 1% DMSO for most of our test organisms, and from 200 to 0.1 µg/mL in 2% DMSO for yeasts. Row A served as a control with no test compound (indicating no inhibition), and Row H was left uninoculated (serving as a control for complete inhibition).

B. subtilis (ATCC 6633), *S. aureus* (ATCC 25923) and MRSA (ATCC 33592) were used as indicator species for antibacterial activity, employing the ProTOX bioassay platform. A bacterial suspension (50 mL in a 250 mL flask) was prepared in nutrient media by cultivating it for 24 h at 250 rpm and 28 °C. The suspension was diluted to an absorbance of 0.01 absorbance units per mL, and 10 µL aliquots were added to the wells of a 96-well microtitre plate containing the test compounds dispersed in 190 µL of nutrient broth (Amyl) with 10 µL resazurin (12.5 µg/mL). The plates were incubated at 28 °C for 48 h, during which the positive control wells changed colour from blue to light pink. Tetracycline was employed as a control for *B. subtilis*, while ampicillin was used for *S. aureus* and MRSA. MIC endpoints were determined visually. Absorbance was measured using a Multiskan Skyhigh Microplate Spectrophotometer (Thermo Fisher Scientific) at 605 nm.

Saccharomyces cerevisiae (ATCC 9763) was used as indicator species for antifungal activity, employing the EuTOX bioassay platform. A yeast suspension (50 mL in a 250 mL flask) was prepared in 1% malt extract broth by cultivating it for 24 h at 250 rpm and 24 °C. The suspension was diluted to absorbances of 0.03 absorbance units per mL and 30 µL were added to the wells of a 96-well microtitre plate containing the test compounds dispersed in 100 µL of malt extract agar with bromocresol green (50 µg/mL). The plates were incubated at 28 °C for 48 h, during which the positive control wells changed colour from blue to yellow. Blasticidin S HCl was used as a control. MIC endpoints were determined visually. Absorbance was measured using a Multiskan Skyhigh Microplate Spectrophotometer (Thermo Fisher Scientific) at 605 nm.

NS-1 (ATCC TIB-18) mouse myeloma and NFF (ATCC PCS-201) human neonatal foreskin fibroblast cells were each inoculated in 96-well microtitre plates (190 µL) at 50,000 cells/mL in DMEM (Dulbecco's Modified Eagle Medium + 10% fetal bovine serum (FBS) + 1% penicillin/streptomycin (10,000 U/mL / 10,000 µg/mL, Life Technologies Cat. No. 15140122), together with resazurin (250 µg/mL; 10 µL) and incubated in 37 °C (5% CO_2) incubator. The plates were incubated for 96 h during which time the positive control wells change colour from a blue to pink colour. The absorbance of each well was measured using Multiskan Skyhigh Microplate Spectrophotometer (Thermofisher Scientific) at 605 nm. Sparsomycin was used as a control. The IC_{50} values were determined using a sigmoidal dose-response model with variable slope in GraphPad Prism 8.

Supplementary Tables

Table S1. Presence or absence of homomeric depside aglycone molecules in extracts derived from *A. nidulans*, *S. cerevisiae* and *A. gemini*. The presence of compounds was examined using LC-MS and HR-ESI-MS analysis.

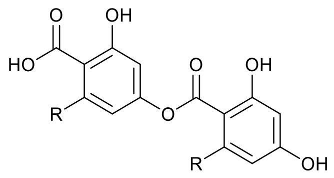

R	*A. nidulans*	*S. cerevisiae*	*A. gemini*
CH_3	-	-	-
$C_{15}H_{31}$	-	-	-
$C_{17}H_{31}$	-	-	-

Table S2. Presence or absence of glycosylated homomeric depside molecules in extracts derived from *A. nidulans* and *A. gemini*. The presence of compounds was examined using LC-MS and HR-ESI-MS analysis.

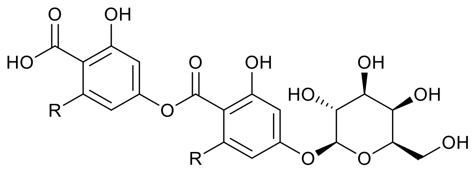

R	*A. nidulans*	*A. gemini*
CH_3	-	-
$C_{15}H_{31}$	-	-
$C_{17}H_{31}$	-	-

Table S3. Presence or absence of potential glycosylated heteromeric depside molecules in extracts derived from *A. nidulans* expressing all genes from the *aqu* BGC. The presence of compounds was examined using LC-MS and HR-ESI-MS analysis.

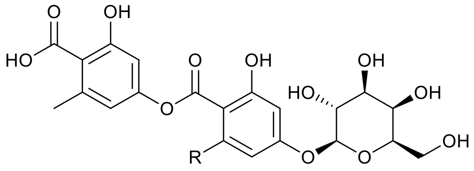

R	Building block	Identifier	Present /Absent
C_2H_5	(C3:0)		-
C_3H_7	(C4:0)		-
C_4H_9	(C5:0)		-
C_5H_{11}	(C6:0)		-
C_6H_{13}	(C7:0)		-
C_7H_{15}	(C8:0)		-
C_9H_{19}	(C10:0)		-
$C_{11}H_{23}$	(C12:0)		-
$C_{12}H_{25}$	(C13:0)		-
$C_{13}H_{27}$	(C14:0)	ariestatin A (**3a**)	**Present**
$C_{15}H_{31}$	(C16:0)	aquastatin A (**1a**)	**Present**
$C_{15}H_{29}$	(C16:1)	capricostatin A (**4a**)	**Present**
$C_{17}H_{35}$	(C18:0)		-
$C_{17}H_{31}$	(C18:2)	geministatin A (**2a**)	**Present**
$C_{18}H_{37}$	(C19:0)		-
$C_{19}H_{39}$	(C20:0)		-
$C_{19}H_{31}$	(C20:4)		-

Table S4. Presence or absence of potential heteromeric depside aglycone molecules in extracts derived from *A. nidulans* expressing all genes from the *aqu* BGC. The presence of compounds was examined using LC-MS and HR-ESI-MS analysis.

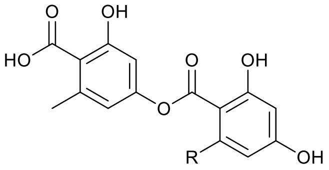

R	Building block	Identifier	Present /Absent
C_2H_5	(C3:0)		-
C_3H_7	(C4:0)		-
C_4H_9	(C5:0)		-
C_5H_{11}	(C6:0)		-
C_6H_{13}	(C7:0)		-
C_7H_{15}	(C8:0)		-
C_9H_{19}	(C10:0)		-
$C_{11}H_{23}$	(C12:0)		-
$C_{12}H_{25}$	(C13:0)		-
$C_{13}H_{27}$	(C14:0)	ariestatin B (**3b**)	**Present**
$C_{15}H_{31}$	(C16:0)	aquastatin B (**1b**)	**Present**
$C_{15}H_{29}$	(C16:1)	capricostatin B (**4b**)	**Present**
$C_{17}H_{35}$	(C18:0)		-
$C_{17}H_{31}$	(C18:2)	geministatin B (**2b**)	**Present**
$C_{18}H_{37}$	(C19:0)		-
$C_{19}H_{39}$	(C20:0)		-
$C_{19}H_{31}$	(C20:4)		-

Table S5. Presence or absence of *O*-methylated and glycosylated alkylresorcylic acid molecules in extracts derived from *A. nidulans* expressing all genes from the *aqu* BGC.

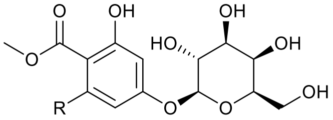

R	Building block	Number	Present /Absent
$C_{13}H_{27}$	(C14:0)	ariestatin C (**3c**)	**Present**
$C_{15}H_{31}$	(C16:0)	aquastatin C (**1c**)	-
$C_{15}H_{29}$	(C16:1)	capricostatin C (**4c**)	-
$C_{17}H_{31}$	(C18:2)		-

Table S6. Presence or absence of glycosylated alkylresorcylic acid molecules in extracts derived from *A. nidulans* expressing all genes from the *aqu* BGC.

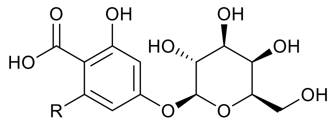

R	Building block	Number	Presence/Absence
$C_{13}H_{27}$	(C14:0)	ariestatin C (**3d**)	**Present**
$C_{15}H_{31}$	(C16:0)	aquastatin D (**1d**)	**Present**
$C_{15}H_{29}$	(C16:1)	capricostatin D (**4d**)	**Present**
$C_{17}H_{31}$	(C18:2)		**Present**

Table S7. Presence or absence of *O*-methylated alkylresorcylic acid molecules in extracts derived from *A. nidulans* expressing all genes from the *aqu* BGC.

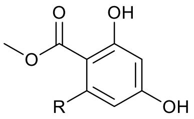

R	Building block	Number	Present / Absent
$C_{13}H_{27}$	(C14:0)	ariestatin E (**3e**)	**Present**
$C_{15}H_{31}$	(C16:0)	aquastatin E (**1e**)	**-**
$C_{15}H_{29}$	(C16:1)	capricostatin E (**4e**)	**Present**
$C_{17}H_{31}$	(C18:2)		**Present**

Table S8. Presence or absence of alkylresorcylic acid molecules in extracts derived from *A. nidulans* expressing all genes from the *aqu* BGC.

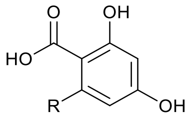

R	Building block	Identifier	Presence/Absence
$C_{13}H_{27}$	(C14:0)	ariestatin F (**3f**)	**-**
$C_{15}H_{31}$	(C16:0)	corticiolic acid (**1f**)	**Present**
$C_{15}H_{29}$	(C16:1)	capricostatin F (**4f**)	**Present**
$C_{17}H_{31}$	(C18:2)		**Present**

Table S9. Presence or absence of alkylresorcylic acid molecules and orsellinic acid in extracts derived from *S. cerevisiae* expressing *aquA*.

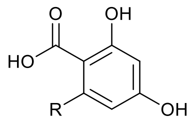

R	Building block	Identifier	Presence/Absence
CH_3	(C2:0)	Orsellinic acid (**5**)	**Present**
$C_{13}H_{27}$	(C14:0)	ariestatin F (**3f**)	**-**
$C_{15}H_{31}$	(C16:0)	corticiolic acid (**1f**)	**Present**
$C_{15}H_{29}$	(C16:1)	capricostatin F (**4f**)	**Present**
$C_{17}H_{31}$	(C18:2)		**-**

Table S10. Vectors used in this study.

Vector name	Backbone plasmid	Fungal marker	Purpose/Genes
pYFAC-CH2	--	*pyrG*	Heterologous expression *A. nidulans*
pYFAC-CH3	--	*ribo*	Heterologous expression *A. nidulans*
pXW55	--	*URA3*	Heterologous expression *S. cerevisiae*
pYFACNS00020	pYFAC-CH2	*pyrG*	*aquA*
pYFACNS00021	pYFAC-CH2	*pyrG*	*aquAB*
pYFACNS00022	pYFAC-CH3	*ribo*	*aquC*
pXW55::*aquA*	pXW55	*URA3*	*aquA*
pXW55::*aquA-ΔSAT*	pXW55	*URA3*	*aquA-ΔSAT*
pXW55::*aquA-ΔTE*	pXW55	*URA3*	*aquA-ΔTE*

Table S11. Strains built in this study for heterologous expression.

Strain number	Strain name
1	*A. nidulans aquABC*
2	*A. nidulans aquAB*
3	*A. nidulans aquAC*
4	*A. nidulans aquA*
5	*S. cerevisiae aquA*
6	*S. cerevisiae aquA-ΔSAT*
7	*S. cerevisiae aquA-ΔTE*

Table S12. Oligonucleotides used in this study.

Oligonucleotide	Sequence (5′ to 3′)	Purpose
FP2131_AquA_fg1CH2_PacI_F	TTAATTAGAACTCTTCCAATCCTATCACCTCGC CTTAATGGCCTCTCCACCCCG	Heterologous expression of *aquA* in *A. nidulans*
FP2131_AquA_fg1CH2_R	GAGGTACTCTGCGCTGAAGG	Heterologous expression of *aquA* in *A. nidulans* and *S. cerevisiae*
FP2131_AquA_fg2CH2_F	TCGTCCTCAGCGGTAAAGTC	Heterologous expression of *aquA* in *A. nidulans* and *S. cerevisiae*
FP2131_AquA_fg2CH2_PacI_R	CGCGCTCCACGGGGACTCGCTTCAATTTGTTCC GCTTAATCTAGATCGTGAACTTCTCC	Heterologous expression of *aquA* in *A. nidulans*
FP2131_AquB_CH2_NotI_F	TGAGATACCAAAGCATTGAGCCCAGAAACAGCA GAAGCATGAGCGCAACGACGACTC	Heterologous expression of *aquB* in *A. nidulans*
FP2131_AquB_CH2_NotI_R	AGTCTAAAGGTCTACAATCAATTCAGGCCGTAT TCAGGGCCTAGAATGCAGCTGGCCTC	Heterologous expression of *aquB* in *A. nidulans*
FP2131_AquC_CH3_PacI_F	TTAATTAGAACTCTTCCAATCCTATCACCTCGC CTTAATGACTCTCCCGTCGATTC	Heterologous expression of *aquC* in *A. nidulans*
FP2131_AquC_CH3_PacI_R	CGCGCTCCACGGGGACTCGCTTCAATTTGTTCC GCTTAATCTAAAAAAAGCTTCGCGC	Heterologous expression of *aquC* in *A. nidulans*
FP2131_AquA_pXW55_SpeI_F	CTAGCGATTATAAGGATGATGATGATAAGACTA GTATGGCCTCTCCACCCCG	Heterologous expression in *S. cerevisiae*
FP2131_AquA_pXW55_PmlI_R	ATTTAAATTAGTGATGGTGATGGTGATGCACGT GGATCGTGAACTTCTCCAGCG	Heterologous expression in *S. cerevisiae*
FP2131_KS-AT-PT-ACP_pXW55_SpeI_F	CTAGCGATTATAAGGATGATGATGATAAGACTA GTATCGCTGTTGTGGGCATGGCAGGTC	Domain deletion in *S. cerevisiae*
FP2131_KS-AT-PT-ACP_pXW55_PmlI_R	ATTTAAATTAGTGATGGTGATGGTGATGCACGT GGCTCGCGGCGTTGGAGCTGCGCTTGG	Domain deletion in *S. cerevisiae*

Table S13. Additional bioassay results for compounds investigated in this study.

Compound	Minimum inhibitory concentration (MIC; µg mL^{-1})				IC_{50} (µM)	
	B. subtilis	***S. aureus***	**MRSA**	***S. cerevisiae***	**NS-1**	**NFF**
aquastatin C (**1c**)	>100	>100	>100	>200	>100	>100
aquastatin E (**1e**)	>100	>100	>100	>200	>100	>100
corticiolic acid (**1f**)	0.8	3.1	3.1	>200	25	>100
capricostatin C (**4c**)	>100	>100	>100	>200	6.3	6.3
orsellinic acid (**5**)	>100	>100	>100	>200	>100	>100
adipostatin (**6**)	>100	>100	25	>200	6.3	6.3
Controls	6.3	3.1	>100	3.1	1.7	1.7

Controls: *B. subtilis* ATCC 6633 = tetracycline; *S. aureus* ATCC 25923 and MRSA ATCC 33592 = ampicillin; *S. cerevisiae* ATCC 9763 = blasticidin S HCl; NS-1 ATCC TIB-18 and NFF TCC PCS-201) = sparsomycin.

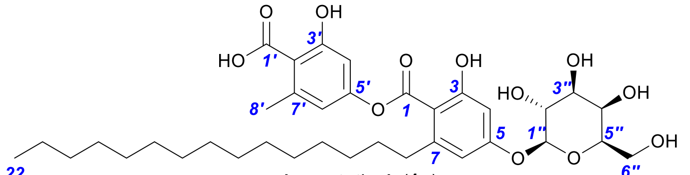

Aquastatin A (**1a**)

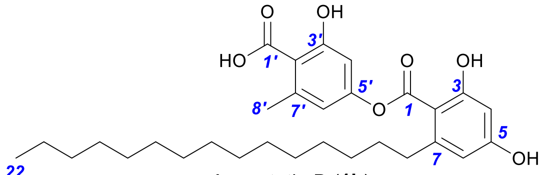

Aquastatin B (**1b**)

Table S14. ^{1}H (600 MHz) and ^{13}C (150 MHz) NMR data for **1a** and **1b** in DMSO-d_6

Pos.	Aquastatin A (1a)		Aquastatin B (1b)	
	δ_C, type	δ_H, mult (*J* in Hz)	δ_C, type	δ_H, mult (*J* in Hz)
1	166.2, C		166.8, C	
2	113.0, C		109.3, C	
3	157.2, C		158.7, C	
3-OH		10.20, s		10.12, s
4	101.4, CH	6.46, d (2.2)	100.5, CH	6.22, d (2.2)
5	159.6, C		160.5, C	
5-OH				9.84, s
6	108.4, CH	6.43, d (2.2)	108.4, CH	6.17, d (2.2)
7	143.1, C		144.1, C	
8	33.5, CH_2	2.60, m	33.8, CH_2	2.59, m
9	30.8, CH_2	1.54, m	31.0, CH_2	1.51, m
10–19	28.7-29.9[a], CH_2	1.18–1.30[b], m	28.7–29.9[a], CH_2	1.20–1.27[b], m
20	31.3, CH_2	1.21[b], m	31.3, CH_2	1.21[b], m
21	22.1, CH_2	1.24[b], m	22.1, CH_2	1.24[b], m
22	13.9, CH_3	0.84, t (6.9)	13.9, CH_2	0.83, t (7.0)
1′	170.6, C		170.7, C	
1′-OH		13.33, br s		13.33, br s
2′	116.6, C		116.3, C	
3′	158.9, C		159.2, C	
3′-OH		11.18, br s		11.12, br s
4′	107.2, CH	6.58, d (2.1)	107.2, CH	6.56, d (2.2)
5′	152.3, C		152.4, C	
6′	114.4, CH	6.52, br d (2.1)	114.5, CH	6.51, dd (2.2, 0.6)
7′	139.6, C		139.6, C	
8′	21.0, CH_3	2.36, s	21.1, CH_3	2.36, br s
1″	100.5, CH	4.81[c], d (7.7)		
2″	70.2, CH	3.54[d], m		
3″	73.2, CH	3.41, dd (9.7, 3.2)		
4″	67.9, CH	3.71, br d (3.2)		
5″	75.4, CH	3.55[d], m		
6″	60.0, CH_2	3.56[d], m		
		3.48, m		
2″-OH		5.15[e], br s		
3″-OH		4.81[c,e], br s		
4″-OH		4.49[e], br s		
6″-OH		4.62[e], br s		

[a] assignments interchangeable, [b-d] overlapping resonances, [e] assignments based on those for **4c** (Table S22).

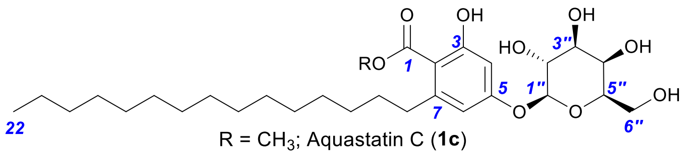

Table S15. ^{1}H (600 MHz) and ^{13}C (150 MHz) NMR data for **1c** and **1d** in DMSO-*d*$_6$

**Pos.**	**Aquastatin C (1c)**		**Aquastatin D (1d)**	
	**δ$_C$, type**	**δ$_H$, mult (*J* in Hz)**	**δ$_C$, type**	**δ$_H$, mult (*J* in Hz)**
1	169.0, C		171.9, C	
1-OCH$_3$	51.6	3.74, s		
1-OH				13.38, br s
2	113.5, C		109.3, C	
3	157.9, C		161.6, C	
3-OH		9.78, br s		11.45, br s
4	101.4, CH	6.37, d (2.2)	101.3, CH	6.39, d (2.2)
5	159.3, C		160.3, C	
6	108.0, CH	6.32, d (2.2)	109.8, CH	6.36, d (2.2)
7	143.0, C		145.8, C	
8	33.7, CH$_2$	2.47, m	34.9, CH$_2$	2.27, m
9	30.7, CH$_2$	1.44, m	31.2, CH$_2$	1.48, m
10–19	28.7–29.0[a], CH$_2$	1.21–1.26[b], m	28.7–29.1[a], CH$_2$	1.15–1.29[b], m
20	31.3, CH$_2$	1.21[b], m	31.3, CH$_2$	1.21[b], m
21	22.1, CH$_2$	1.24[b], m	22.1, CH$_2$	1.23[b], m
22	13.9, CH$_3$	0.84, t (7.0)	13.9, CH$_2$	0.84, t (6.9)
1″	100.5, CH	4.76[c], d (7.5)	100.2, CH	4.83, d (7.7)
2″	70.2, CH	3.52[d], m	70.1, CH	3.52[d], m
3″	73.2, CH	3.39, dd (9.5, 3.3)	73.2, CH	3.39, dd (9.5, 3.4)
4″	67.9, CH	3.69, br d (3.3)	68.0, CH	3.69, br d (3.2)
5″	75.4, CH	3.53[d], m	75.5, CH	3.53[d], m
6″	60.0, CH$_2$	3.54[d], m	60.2, CH$_2$	3.57[d], m
		3.46, m		3.45, m
2″-OH		5.15[e], br s		5.12[e], br s
3″-OH		4.81[c,e], br s		4.83[c,e], br s
4″-OH		4.49[e], br s		4.47[e], br s
6″-OH		4.62[e], br s		4.61[e], br s

[a] assignments interchangeable, [b-d] overlapping resonances, [e] assignments based on those for **4c** (Table S22).

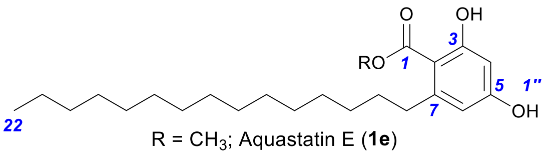

R = CH_3; Aquastatin E (**1e**)
R = H; Corticiolic acid (**1f**)

Table S16. ^{1}H (600 MHz) and ^{13}C (150 MHz) NMR data for **1e** and **1f** in DMSO-d_6

**Pos.**	**Aquastatin E (1e)**		**Corticiolic acid (1f)**	
	**δ_C, type**	**δ_H, mult (*J* in Hz)**	**δ_C, type**	**δ_H, mult (*J* in Hz)**
1	169.6, C		172.7, C	
1-OCH_3	51.6	3.74, s		
1-OH				13.31, s
2	108.8, C		105.2, C	
3	159.3, C		163.5, C	
3-OH		10.20, br s		11.78, s
4	100.4, CH	6.13, d (2.2)	100.5, CH	6.14, d (2.4)
5	160.7, C		161.5, C	
5-OH		10.20, br s		9.99, s
6	108.8, CH	6.10, d (2.2)	109.9, CH	6.11, d (2.4)
7	144.4, C		147.1 C	
8	34.2, CH_2	2.51, m	35.3, CH_2	2.72, m
9	30.9, CH_2	1.42, m	31.3, CH_2	1.46, m
10–19	28.7–29.0[a], CH_2	1.20–1.27[b], m	28.7–29.1[a], CH_2	1.21–1.27[b], m
20	31.3, CH_2	1.22[b], m	31.2, CH_2	1.22, m
21	22.1, CH_2	1.24[b], m	22.1, CH_2	1.24, m
22	13.9, CH_3	0.84, t (6.9)	13.9, CH_2	0.84, t (6.9)

[a] assignments interchangeable, [b] overlapping resonances

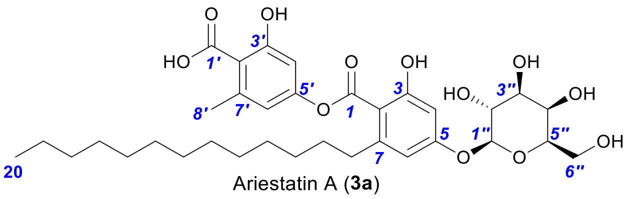

Ariestatin A (**3a**)

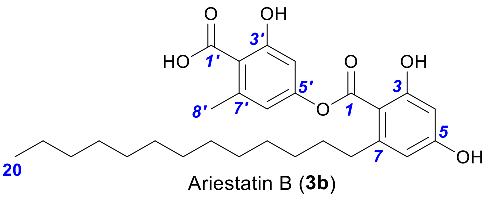

Ariestatin B (**3b**)

Table S17. ^{1}H (600 MHz) and ^{13}C (150 MHz) NMR data for **3a** and **3b** in DMSO-d_6

Pos.	Ariestatin A (3a)		Ariestatin B (3b)	
	δ_C, type	δ_H, mult (*J* in Hz)	δ_C, type	δ_H, mult (*J* in Hz)
1	166.2, C		166.8, C	
2	113.0, C		109.3, C	
3	157.2, C		158.7, C	
3-OH		10.20, s		10.11, s
4	101.4, CH	6.46, d (2.2)	100.4, CH	6.22, d (2.2)
5	159.6, C		160.5, C	
5-OH				9.84, s
6	108.4, CH	6.43, d (2.2)	108.4, CH	6.17, d (2.2)
7	143.2, C		144.1, C	
8	33.5, CH_2	2.60, m	33.8, CH_2	2.59, m
9	30.8, CH_2	1.54, m	31.0, CH_2	1.51, m
10–17	28.7–29.0[a], CH_2	1.18–1.30[b], m	28.7–29.0[a], CH_2	1.19–1.27[b], m
18	31.3, CH_2	1.21, m	31.2, CH_2	1.20, m
19	22.1, CH_2	1.24, m	22.0, CH_2	1.23, m
20	13.9, CH_3	0.83, t (6.9)	13.9, CH_2	0.83, t (7.2)
1′	170.6, C		170.7, C	
1′-OH		13.33, br s		13.44, br s
2′	116.6, C		116.3, C	
3′	158.9, C		159.2, C	
3′-OH		11.27, br s		11.06, br s
4′	107.2, CH	6.58, d (1.9)	107.2, CH	6.57, d (2.2)
5′	152.3, C		152.4, C	
6′	114.4, CH	6.52, br d (1.9)	114.6, CH	6.52, dd (2.2, 0.6)
7′	139.6, C		139.6, C	
8′	21.0, CH_3	2.36, s	21.1, CH_3	2.36, br s
1″	100.5, CH	4.81[c], d (7.7)		
2″	70.2, CH	3.54[d], m		
3″	73.2, CH	3.41, dd (9.7, 3.2)		
4″	67.9, CH	3.71, br d (3.2)		
5″	75.4, CH	3.55[d], m		
6″	60.0, CH_2	3.56[d], m		
		3.48, m		
2″-OH		5.15[e], br s		
3″-OH		4.81[c,e], br s		
4″-OH		4.50[e], br s		
6″-OH		4.63[e], br s		

[a] assignments interchangeable, [b-d] overlapping resonances, [e] assignments based on those for **4c** (Table S22).

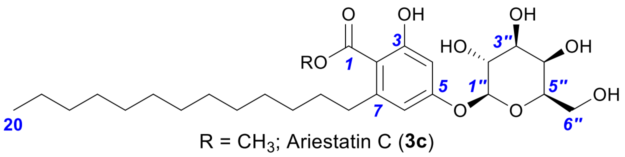

R = CH_3; Ariestatin C (**3c**)
R = H; Ariestatin D (**3d**)

Table S18. ^{1}H (600 MHz) and ^{13}C (150 MHz) NMR data for **3c** and **3d** in DMSO-d_6

**Pos.**	**Ariestatin C (3c)**		**Ariestatin D (3d)**	
	**δ_C, type**	**δ_H, mult (*J* in Hz)**	**δ_C, type**	**δ_H, mult (*J* in Hz)**
1	168.8, C		172.0, C	
1-OCH_3	51.7, CH_3	3.75, s		
				13.43, br s
2	113.2, C		109.6, C	
3	157.4, C		160.9, C	
3-OH		10.11, s		11.47, br s
4	101.2, CH	6.39, d (2.3)	101,1, CH	6.35, br s
5	159.3, C		160.13, C	
5-OH				
6	108.5, CH	6.36, d (2.3)	109.8, CH	6.31, br s
7	143.2, C		146.0, C	
8	33.7, CH_2	2.49, m	34.9, CH_2	2.75, m
9	30.7, CH_2	1.44, m	31.3, CH_2	1.47, m
10–17	28.7–29.0[a], CH_2	1.22–1.24[b], m	28.7–29.2[a], CH_2	1.20–1.29[b], m
18	31.3, CH_2	1.22[b], m	31.3, CH_2	1.22[b], m
19	22.1, CH_2	1.24[b], m	22.1, CH_2	1.24[b], m
20	13.9, CH_3	0.84, t (6.8)	14.0, CH_2	0.84, t (7.0)
1″	100.5, CH	4.77, d (7.7)	100.3, CH	4.82[c], d (7.7)
2″	70.2, CH	3.51[c], m	70.1, CH	3.52[d], m
3″	73.2, CH	3.39, ddd (9.5, 5.8, 3.3)	73.2, CH	3.39, m
4″	67.9, CH	3.69, br dd (4.7, 3.3)	68.0, CH	3.69, m
5″	75.4, CH	3.53[c], m	75.4, CH	3.56[d], m
6″	60.1, CH_2	3.54[c], m	60.2, CH_2	3.53[d], m
		3.46, m		3.45, m
2″-OH		5.12, d (5.2)		5.11, d (5.3)
3″-OH		4.82, d (5.8)		4.82, m
4″-OH		4.46, d (4.7)		4.47, d (4.5)
6″-OH		4.61, m		4.61, dd (5.5, 5.1)

[a] assignments interchangeable, [b-c] overlapping resonances

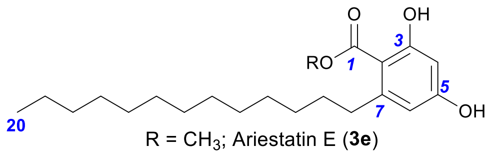

R = CH_3; Ariestatin E (**3e**)
R = H; Ariestatin F (**3f**)

Table S19. ^{1}H (600 MHz) and ^{13}C (150 MHz) NMR data for **3e** and **3f** in DMSO-d_6

Pos.	Ariestatin E (3e)		Ariestatin F (3f)	
	δ_C, type	δ_H, mult (*J* in Hz)	δ_C, type	δ_H, mult (*J* in Hz)
1	169.5, C		172.7, C	
1-OCH_3	51.6	3.74, s		
1-OH				13.31, s
2	109.1, C		105.2, C	
3	159.1, C		163.5, C	
3-OH		10.24, br s		11.78, s
4	100.4, CH	6.14, d (2.3)	100.5, CH	6.14, d (2.4)
5	160.2, C		161.5, C	
5-OH		9.79, br s		9.99, s
6	108.6, CH	6.11, d (2.3)	109.9, CH	6.11, d (2.4)
7	144.3, C		147.1 C	
8	34.1, CH_2	2.51, m	35.3, CH_2	2.72, m
9	30.9, CH_2	1.42, m	31.3, CH_2	1.46, m
10–17	28.7–29.6[a], CH_2	1.20–1.26[b], m	28.7–29.1[a], CH_2	1.21–1.27[b], m
18	31.2, CH_2	1.22, m	31.3, CH_2	1.22[b], m
19	22.0, CH_2	1.24, m	22.1, CH_2	1.24[b], m
20	13.9, CH_3	0.84, t (6.8)	13.9, CH_2	0.84, t (6.9)

[a] assignments interchangeable, [b] overlapping resonances

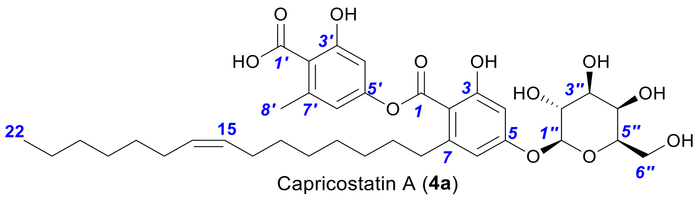

Capricostatin A (**4a**)

Table S20. ^1H (600 MHz) and ^{13}C (150 MHz) NMR data for capricostatin A (**4a**) in DMSO-d_6

Pos.	δ_C, type	δ_H, mult (*J* in Hz)	HMBC	COSY	ROESY
1	166.2, C				
2	113.0, C				
3	157.2, C				
3-OH		10.20, s	2, 3, 4		4
4	101.4, CH	6.46, d (2.2)	2, 3, 5, 6	6	3-OH, 1″
5	159.6, C				
6	108.4, CH	6.43, d (2.2)	2, 4, 6, 8	4	8, 1″
7	143.1, C				
8	33.5, CH_2	2.60, m	2, 6, 7, 9, 10		6
9	30.8, CH_2	1.54, m	7, 8, 10, 11		
10	28.7, CH_2	1.23–1.29[c], m			
11	28.9[a], CH_2	1.23–1.29[c], m			
12	28.5[b], CH_2	1.23–1.29[c], m			
13	29.1[a], CH_2	1.23–1.29[c], m	15	14	15
14	26.5, CH_2	1.94[d], m	12, 15, 16	13, 15	
15	129.6, CH	5.29[e], m	13, 14, 16, 17	14	13
16	129.6, CH	5.29[e], m	14, 15, 17, 18	17	18
17	26.5, CH_2	1.94[d], m	15, 16, 18	16, 18	
18	29.0[a], CH_2	1.23–1.29[c], m	16	17	16
19	28.2[b], CH_2	1.22[c], m			
20	31.0, CH_2	1.20[c], m			
21	22.0, CH_2	1.23[c], m		22	
22	13.9, CH_3	0.82, t (7.0)	20, 21	21	
1′	170.6, C				
1′-OH		11.42, br s			
2′	116.7, C				
3′	159.1, C				
3′-OH		13.13, br s			
4′	107.2, CH	6.57, d (2.2)	2′, 3′, 5′, 6′	6′	
5′	152.3, C				
6′	114.4, CH	6.51, br d (2.2)	2′, 4′, 5′, 8′	4′	8′
7′	139.6, C				
8′	21.0, CH_3	2.36, s	2′, 6′, 7′		6′
1″	100.5, CH	4.80, d (7.8)	5, 2″, 3″, 5″	2″	4, 6, 3″, 5″
2″	70.2, CH	3.54[f], m	1″, 3″	1″, 3″	3″
3″	73.2, CH	3.41, dd (9.5, 3.3)	2″, 5″	2″, 4″	1″, 2″, 4″
4″	67.9, CH	3.71, br d (3.3)	2″, 3″	3″	3″, 5″
5″	75.4, CH	3.55[f], m	4″, 6″		1″, 4″
6″	60.0, CH_2	3.55[f], m	4″, 5″		
		3.48, m	4″, 5″		
2″-OH		5.15[g] br s			
3″-OH		4.84[g] br s			
4″-OH		4.49[g] br s			
6″-OH		4.62[g] br s			

[a-b] assignments interchangeable, [c-f] overlapping resonances, [g] assignments based on those for **4c** (Table S22).

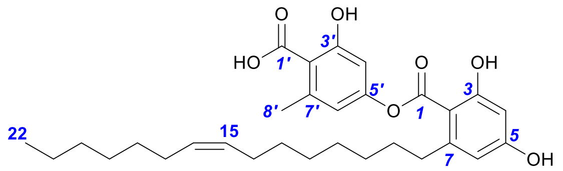

Capricastatin B (**4b**)

Table S21. ^{1}H (600 MHz) and ^{13}C (150 MHz) NMR data for capricostatin B (**4b**) in DMSO-d_6

**Pos.**	**δC, type**	**δH, mult (*J* in Hz)**	**HMBC**	**COSY**	**ROESY**
1	166.7, C				
2	109.3, C				
3	158.7, C				
3-OH		10.11, s	2, 3, 4		4
4	100.4, CH	6.22, d (2.2)	2, 3, 5 ,6	6	3-OH, 5-OH
5	160.5, C				
5-OH		9.84, s	4, 5, 6		4, 6
6	108.4, CH	6.17, d (2.2)	2, 4, 5, 8	4	8, 5-OH
7	144.1, C				
8	33.8, CH_2	2.59, m	2, 6, 7, 9, 10	9	6, 9
9	31.0, CH_2	1.51, m	8, 10, 11	8, 10	8
10	28.6[a], CH_2	1.18–1.29[c], m		9	
11	28.9[b], CH_2	1.18–1.29[c], m			
12	28.5[a], CH_2	1.18–1.29[c], m			
13	29.0[b], CH_2	1.18–1.29[c], m	15	14	
14	26.5, CH_2	1.94, m	12, 13, 15, 16	13, 15	15
15	129.6, CH	5.29, m	13, 14, 17	14, 16	14
16	129.6, CH	5.29, m	14, 17, 18	15, 17	17
17	26.5, CH_2	1.94, m	15, 16, 18, 19	16, 18	16
18	29.0[b], CH_2	1.18–1.29[c], m	16	17	
19	28.2, CH_2	1.18–1.29[c], m			
20	31.1, CH_2	1.21[c], m	22		
21	22.0, CH_2	1.23[c], m		22	
22	13.9, CH_3	0.82, t (7.0)	20, 21	21	
1′	170.6, C				
1′-OH		11.05, br s			
2′	116.4, C				
3′	159.0, C				
3′-OH		13.44, br s			
4′	107.2, CH	6.57, d (2.0)	2′, 3′, 5′, 6′	6′	
5′	152.4, C				
6′	114.5, CH	6.52, d (2.0)	2′, 4′, 5′, 8′	4′	8′
7′	139.5, C				
8′	21.0, CH_3	2.36, s	2′, 6′, 7′		6′

[a-b] assignments interchangeable, [c] overlapping resonances

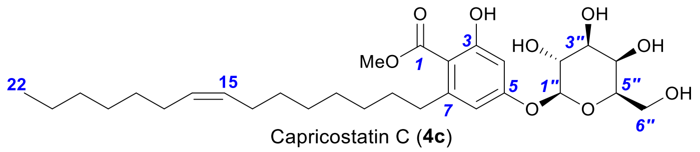

Capricostatin C (**4c**)

Table S22. ^{1}H (600 MHz) and ^{13}C (150 MHz) NMR data for capricostatin C (**4c**) in DMSO-d_6

Pos.	δ$_C$, type	δ$_H$, mult (*J* in Hz)	HMBC	COSY	ROESY
1	168.8, C				
1-OCH$_3$	51.7, CH$_3$	3.75, s	1		
2	113.2, C				
3	157.4, C				
3-OH		10.11, s	2, 3, 4		4
4	101.2, CH	6.39, d (2.3)	2, 3, 5, 6	6	1″, 3-OH
5	159.3, C				
6	108.5, CH	6.36, d (2.3)	2, 4, 5, 8	4	1″
7	143.2, C				
8	33.7, CH$_2$	2.49, m	2, 6, 7, 9, 10	9	10
9	30.7, CH$_2$	1.44, m	11	8, 10	
10	28.6[a], CH$_2$	1.22–1.29[c], m		9	8
11	28.8[b], CH$_2$	1.22–1.29[c], m			
12	28.5[a], CH$_2$	1.22–1.29[c], m			
13	28.6[a], CH$_2$	1.22–1.29[c], m		14	
14	29.0[b], CH$_2$	1.97, m	12, 13, 15, 16	13, 15	
15	129.6, CH	5.31, m	14, 17	14, 16	
16	129.6, CH	5.31, m	14, 17	15, 17	
17	26.6, CH$_2$	1.97, m	15, 16, 18	16, 18	
18	29.0[b], CH$_2$	1.22-1.29[c], m		17	
19	28.2[a], CH$_2$	1.22-1.29[c], m			
20	31.1, CH$_2$	1.23[c], m			
21	22.0, CH$_2$	1.24[c], m	20	22	
22	13.9, CH$_3$	0.84, t (6.8)	20, 21	21	
1″	100.5, CH	4.77, d (7.7)	5, 2″, 5″	2″	4, 6, 5″
2″	70.2, CH	3.51, m	2″, 3″	1″, 3″, 2″-OH	
3″	73.2, CH	3.39, m	2″	2″, 4″, 3″-OH	
4″	67.9, CH	3.69, m	2″, 3″	3″, 4″-OH	
5″	75.4, CH	3.53[d], m	6″		1″
6″a	60.1, CH$_2$	3.54[d], m	4″, 5″	6″b, 6″-OH	
6″b		3.46, m	4″, 5″	6″a, 6″-OH	
2″-OH		5.11, d (5.1)	1″, 2″, 3″	2″	
3″-OH		4.82, d (5.7)	2″, 3″, 4″	3″	
4″-OH		4.46, d (4.6)	4″, 5″	4″	
6″-OH		4.61, m	5″, 6″	6″a, 6″b	

[a-b] assignments interchangeable, [c-d] overlapping resonances

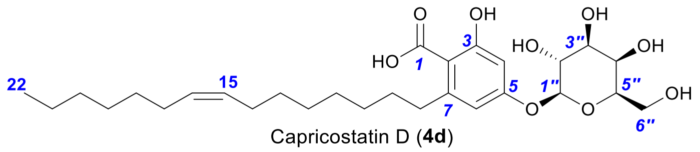

Capricostatin D (**4d**)

Table S23. ^{1}H (600 MHz) and ^{13}C (150 MHz) NMR data for capricostatin D (**4d**) in DMSO-d_6

**Pos.**	**δ_C, type**	**δ_H, mult (*J* in Hz)**	**HMBC**	**COSY**	**ROESY**
1	171.9, C				
1-OH		11.48, br s			
2	109.3, C				
3	161.7, C				
3-OH		13.40, br s			
4	101.1, CH	6.38, d (2.2)	2, 3, 5, 6	6	1″
5	160.3, C				
6	109.8, CH	6.35, d (2.2)	4, 5, 8, 9	4	8, 1″
7	145.8, C				
8	34.9, CH_2	2.73, m	2, 6, 7, 9		6
9	31.2, CH_2	1.48, m	7, 8		
10	28.7[a], CH_2	1.20–1.31[c], m			
11	29.0[b], CH_2	1.20–1.31[c], m			
12	28.6[a], CH_2	1.20–1.31[c], m			
13	29.1[b], CH_2	1.20–1.31[c], m	15		
14	26.6, CH_2	1.97, m	12, 13, 15, 16	13, 15	
15	129.6, CH	5.31, m	13, 14, 17	14, 16	
16	129.6, CH	5.31, m	14, 17, 18	15, 17	
17	26.6, CH_2	1.97, m	15, 16, 18, 19	16, 18	
18	29.1[b], CH_2	1.20–1.31[c], m	16		
19	28.2, CH_2	1.20–1.31[c], m			
20	31.1, CH_2	1.23[c], m	22		
21	22.0, CH_2	1.24[c], m	20, 22		
22	13.9, CH_3	0.84, t (7.1)	20, 21		
1″	100.2, CH	4.83[d], d (7.7)	5, 2″, 3″, 5″	2″	4, 6, 3″, 5″
2″	70.1, CH	3.53[e], m	1″	1″, 2″-OH	
3″	73.2, CH	3.39, br dd (9.8, 2.3)		3″-OH	1″
4″	68.0, CH	3.69, br s	2″, 3″	3″-OH	5″
5″	75.5, CH	3.56[e], m	4″, 6″		1″, 4″
6″a	60.2, CH_2	3.53[e], m	5″	6″-OH	
6″b		3.46, m	5″	6″-OH	
2″-OH		5.11, br d (3.8)		2″	
3″-OH		4.83[d,f], br s		3″	
4″-OH		4.47[f], br m		4″	
6″-OH		4.61[f], br m		6″a, 6″b	

[a-b] assignments interchangeable, [c-e] overlapping resonances, [f] assignments based on those for **4c** (Table S22).

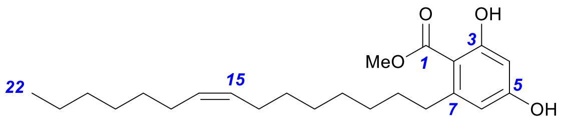

Capricostatin E (**4e**)

Table S24. ^{1}H (600 MHz) and ^{13}C (150 MHz) NMR data for capricostatin E (**4e**) in DMSO-d_6

**Pos.**	**δ_C, type**	**δ_H, mult (*J* in Hz)**	**HMBC**	**COSY**	**ROESY**
1	169.5, C				
1-OCH$_3$	51.6, CH$_3$	3.74, s	1		
2	108.9, C				
3	159.3, C				
3-OH		10.28, br s			
4	100.4, CH	6.13, d (2.3)	2, 3, 5,6		
5	160.6, C				
5-OH		9.83, br s			
6	108.7, CH	6.10, d (2.3)	2, 4, 5, 8		8
7	144.4, C				
8	34.2, CH$_2$	2.51, m	2, 6, 7, 9, 10	9	6, 9
9	30.9, CH$_2$	1.42, br m		8, 10	8
10	28.6^a, CH$_2$	1.26–1.29^c, m		9	
11	28.9^b, CH$_2$	1.26–1.29^c, m			
12	28.5^a, CH$_2$	1.26–1.29^c, m			
13	29.0^b, CH$_2$	1.21–1.26^c, m			
14	26.5, CH$_2$	1.97, dt (7.0, 6.9)	12, 13, 15, 16	13, 15	15
15	129.6, CH	5.31, m	14, 17,	14	14
16	129.6, CH	5.31, m	14, 17	17	17
17	26.5, CH$_2$	1.97, dt (7.0, 6.9)	15, 16, 18, 19	16, 18	16
18	29.0^b, CH$_2$	1.21–1.26^c, m			
19	28.2, CH$_2$	1.21–1.26^c, m			
20	31.1, CH$_2$	1.22^c, m			
21	22.0, CH$_2$	1.24^c, m		22	
22	13.9, CH$_3$	0.84, t, (6.9)	20, 21	21	

[a-b] assignments interchangeable, [c] overlapping resonances

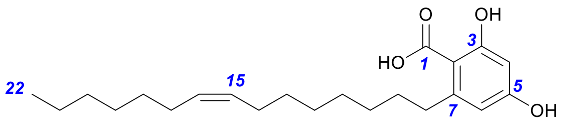

Capricostatin F (**4f**)

Table S25. ^{1}H (600 MHz) and ^{13}C (150 MHz) NMR data for capricostatin F (**4f**) in DMSO-d_6

Pos.	δ_C, type	δ_H, mult (J in Hz)	HMBC	COSY	ROESY
1	172.7, C				
1-OH		13.19, br s			
2	105.2, C				
3	161.5, C				
3-OH		12.00, br s			
4	100.5, CH	6.10, d (2.4)	1, 2, 3, 5, 6		
5	163.6, C				
5-OH		9.99, s			6
6	109.9, CH	6.13, d (2.4)	1, 2, 5, 8		8, 5-OH
7	147.1, C				
8	35.3, CH_2	2.73, m	2, 6, 7	9	6, 10
9	31.3, CH_2	1.46, m	7, 8, 10, 11	8	
10	28.7[a], CH_2	1.22–1.29[c], m			
11	29.0[b], CH_2	1.22–1.29[c], m			
12	28.5[a], CH_2	1.22–1.29[c], m			
13	29.1[b], CH_2	1.22–1.29[c], m			
14	26.6, CH_2	1.97, m	12, 13, 15, 16	13, 15	
15	129.6, CH	5.31, m	13, 14, 16, 17	14	13
16	129.6, CH	5.31, m	14, 15, 17, 18	17	18
17	26.5, CH_2	1.97, m		16, 18	
18	29.1[b], CH_2	1.22–1.29 [c], m			
19	28.2, CH_2	1.22–1.29 [c], m			
20	31.1, CH_2	1.22[c], m			
21	22.0, CH_2	1.24[c], m		22	
22	13.9, CH_3	0.83, t (7.0) m	20, 21	21	

[a-b] assignments interchangeable, [c] overlapping resonances

Supplementary Figures

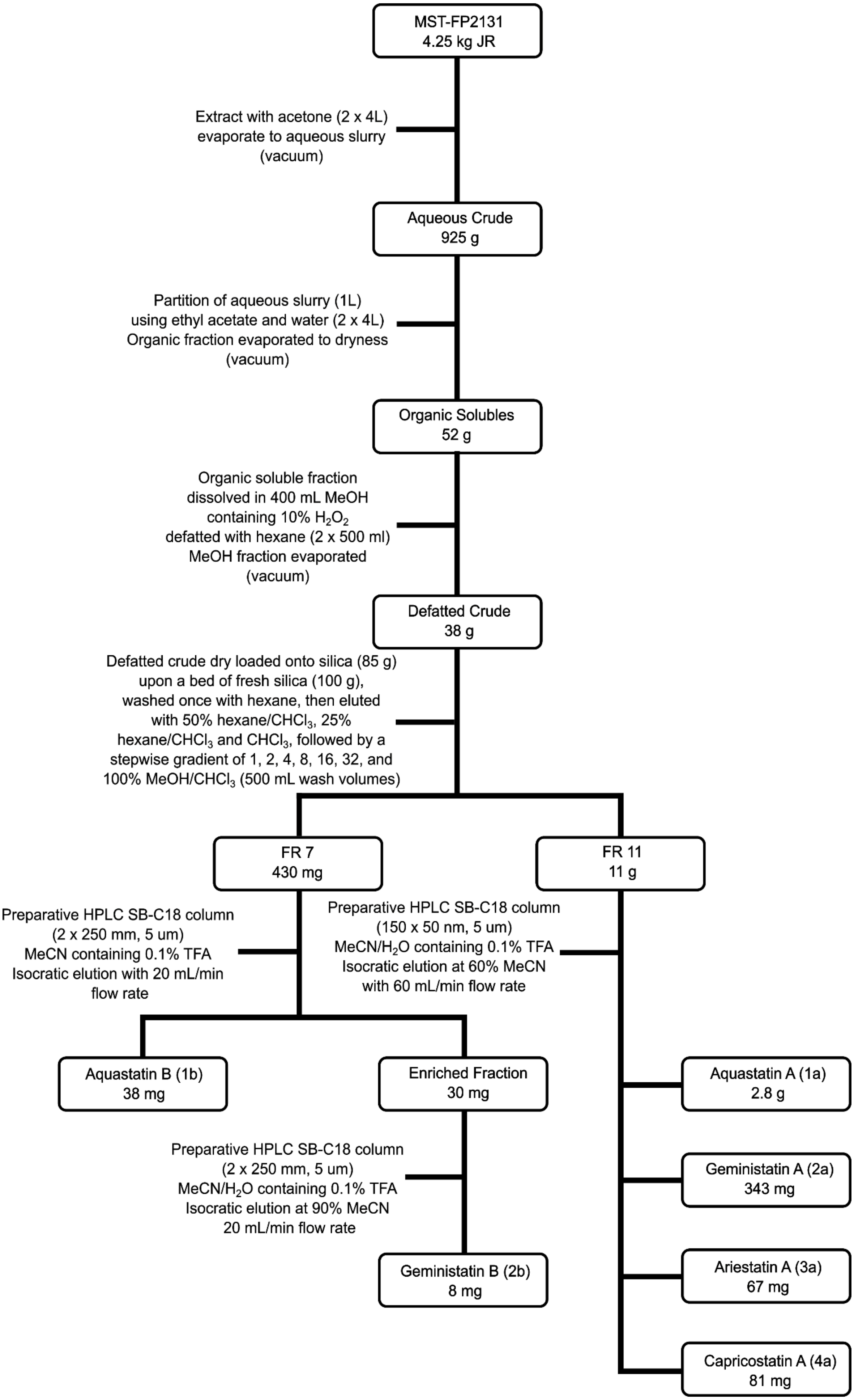

Figure S1. Fractionation scheme for the isolation of metabolites from *A. gemini* MST-FP2131

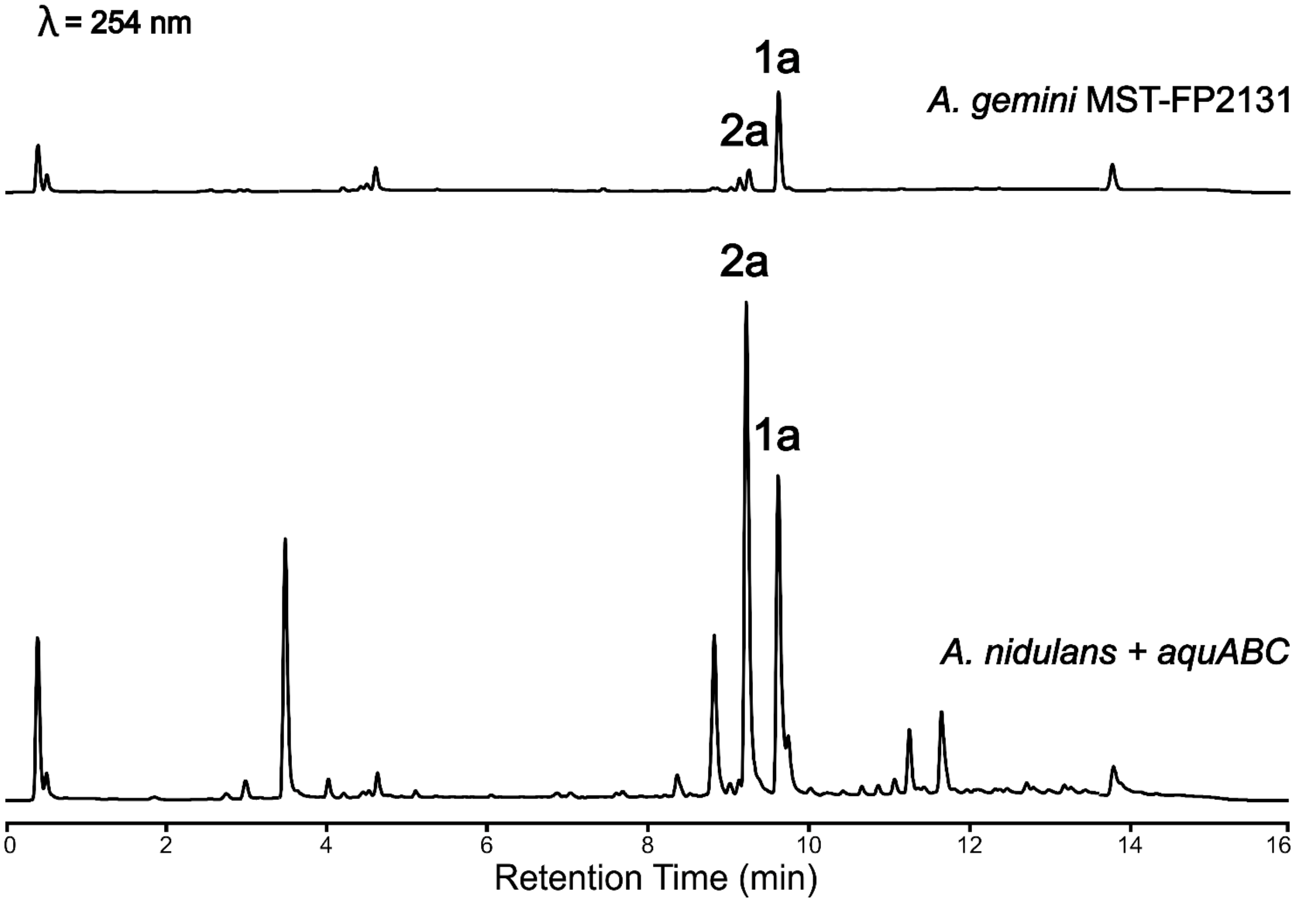

Figure S2. Comparison of the metabolic profiles of *A. nidulans* and *A. gemini*

LC-DAD (254 nm) chromatograms of *A. nidulans* LO8030 harbouring the *aqu* BGC and *A. gemini* MST-FP2131 grown under similar conditions and in the same medium. The peaks for aquastatin A (**1a**) and geministatin A (**2a**) are highlighted to emphasize the differences in the ratio between both compounds in the two species. Both samples are in the same y-axis scale.

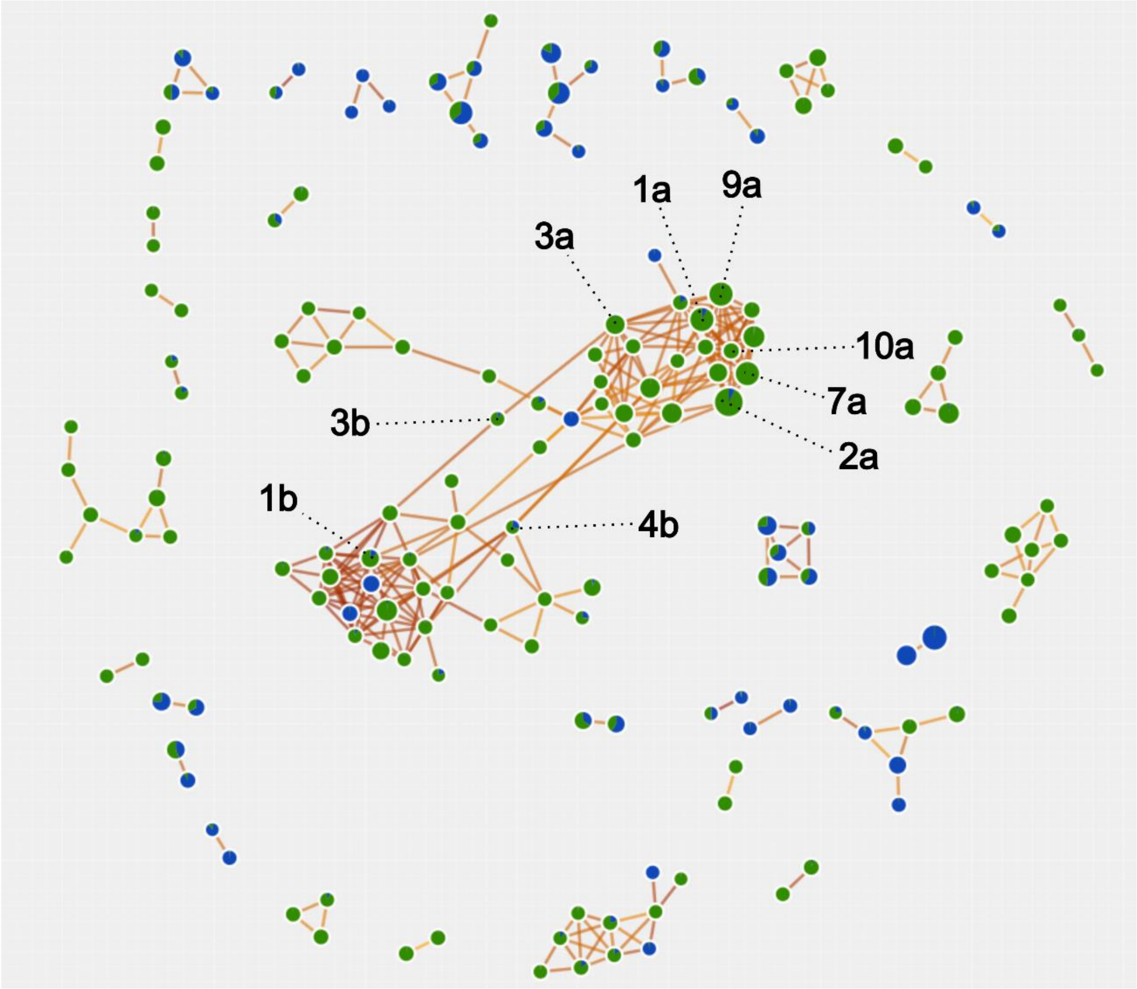

Figure S3. MS-based molecular networking analysis.

The MS-based molecular networking analysis was conducted using Compound Discoverer Software to compare a control strain (*A. nidulans* LO8030 with empty plasmids - Blue) with *A. nidulans* LO8030 expressing the *aqu* BGC (Green). Node styles are represented as pie charts, and node size is determined by the area of the peaks. Link styles are coloured according to the coverage. Node links and cluster size are defined based on default values (10 and 100, respectively). The score threshold is set to 40, the coverage threshold to 70, and the matched fragments threshold to 40. The nodes of some compounds are indicated in the figure.

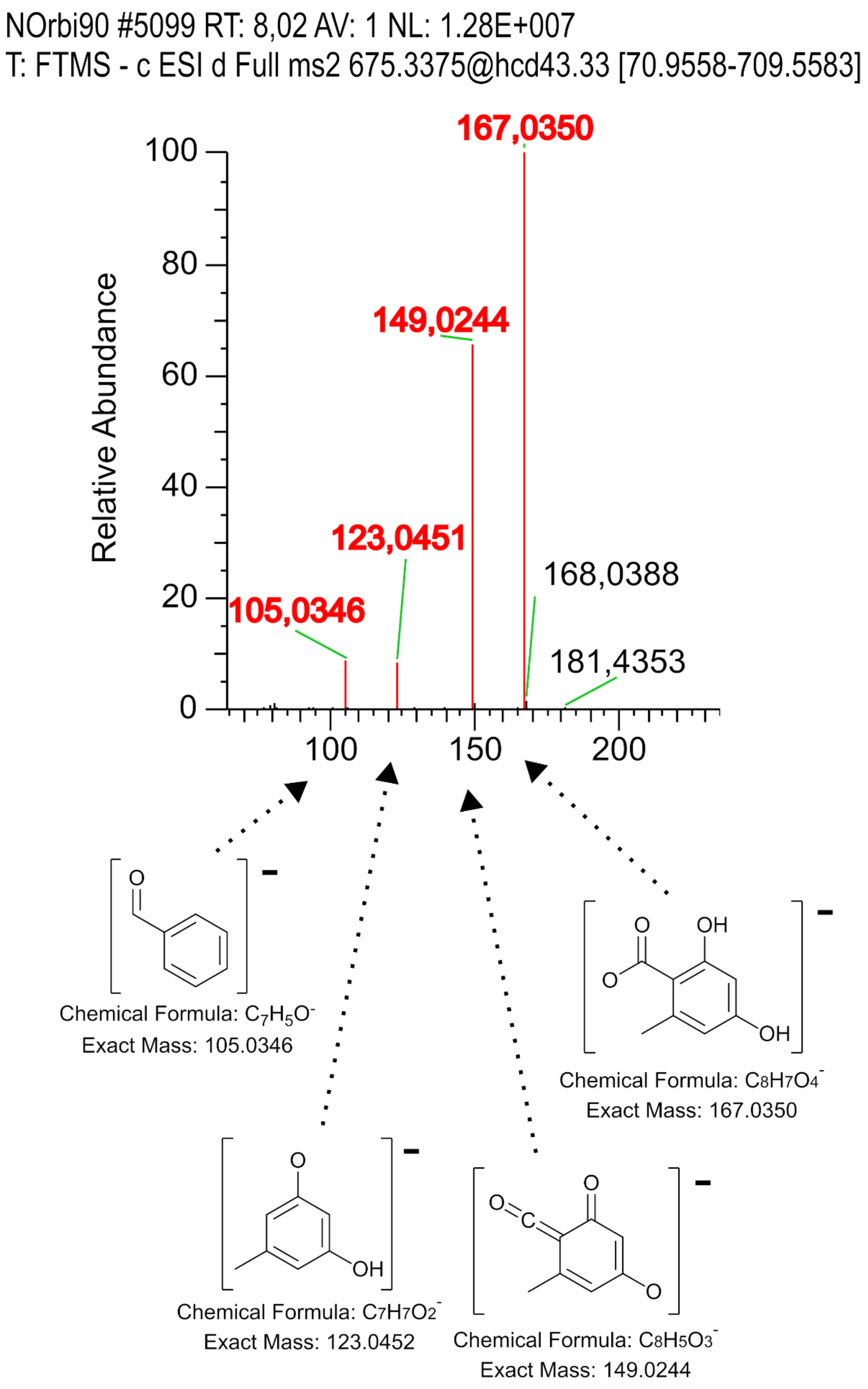

Figure S4. HRESI(−)MS/MS fragmentation pattern of orsellinic acid (**5**).

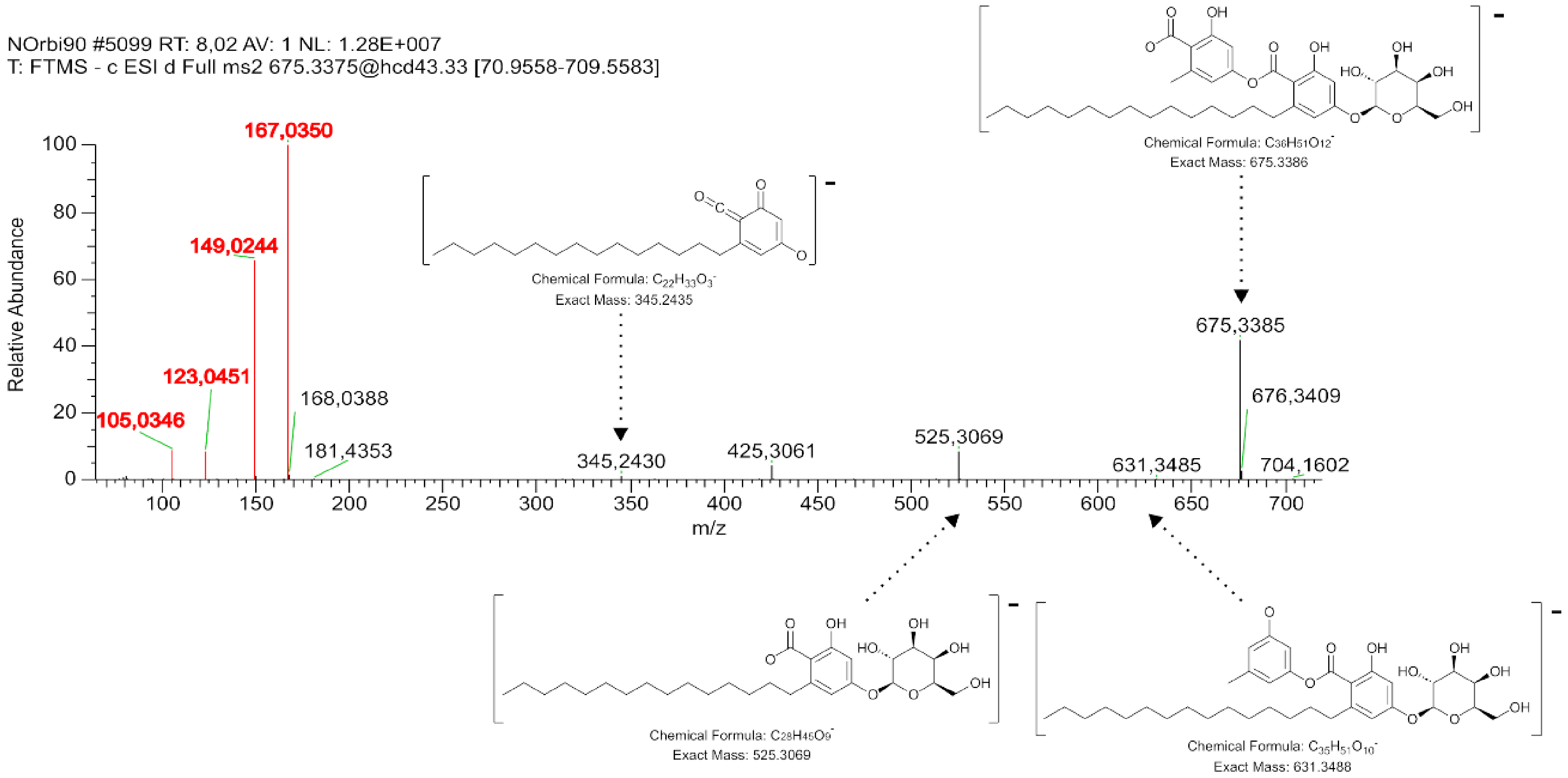

Figure S5. HRESI(−)MS/MS fragmentation pattern of aquastatin A (**1a**).

Fragments highlighted in red are described in Figure S4.

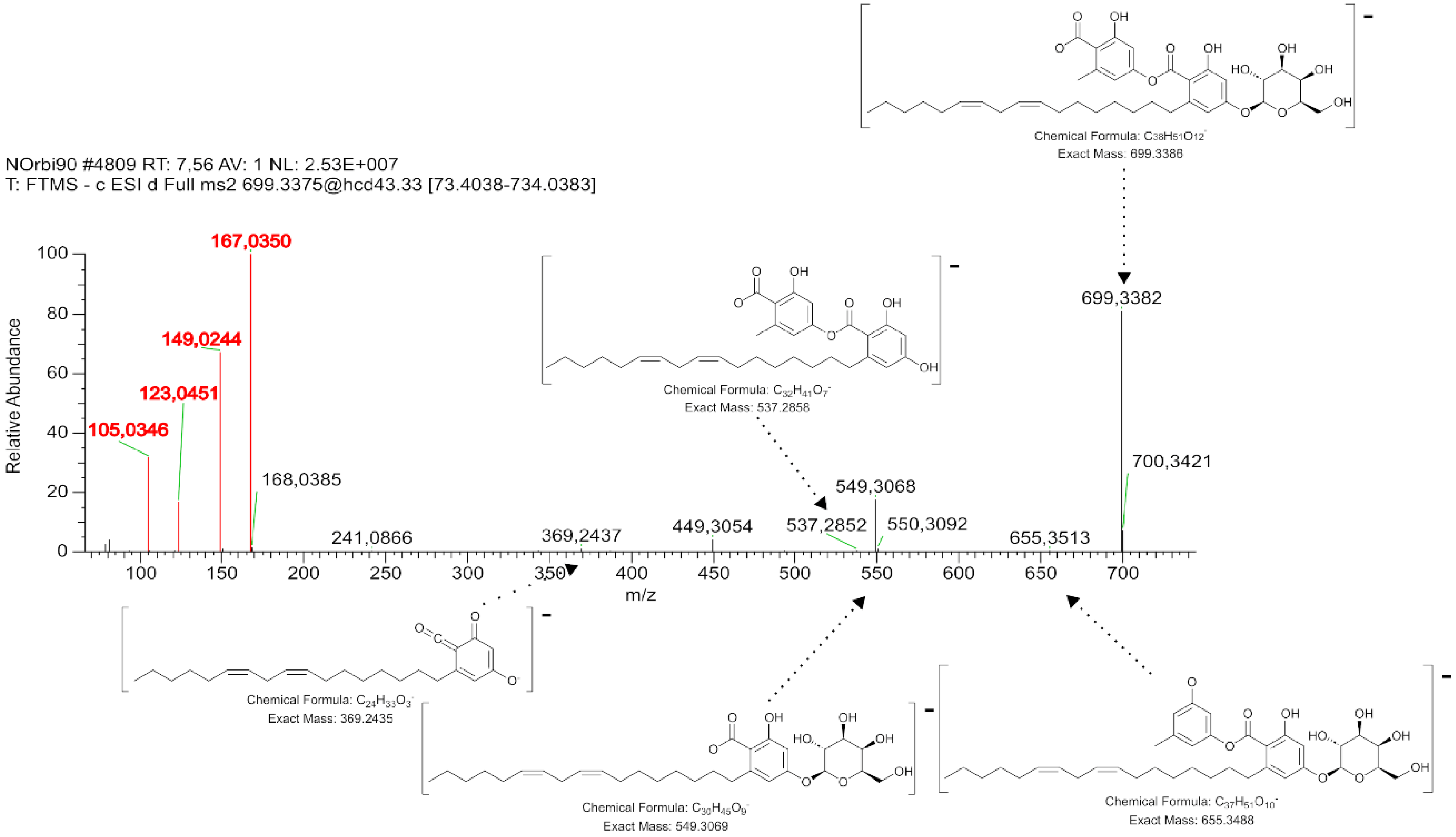

Figure S6. HRESI(−)MS/MS fragmentation pattern of geministatin A (**2a**).

Fragments highlighted in red are described in Figure S4.

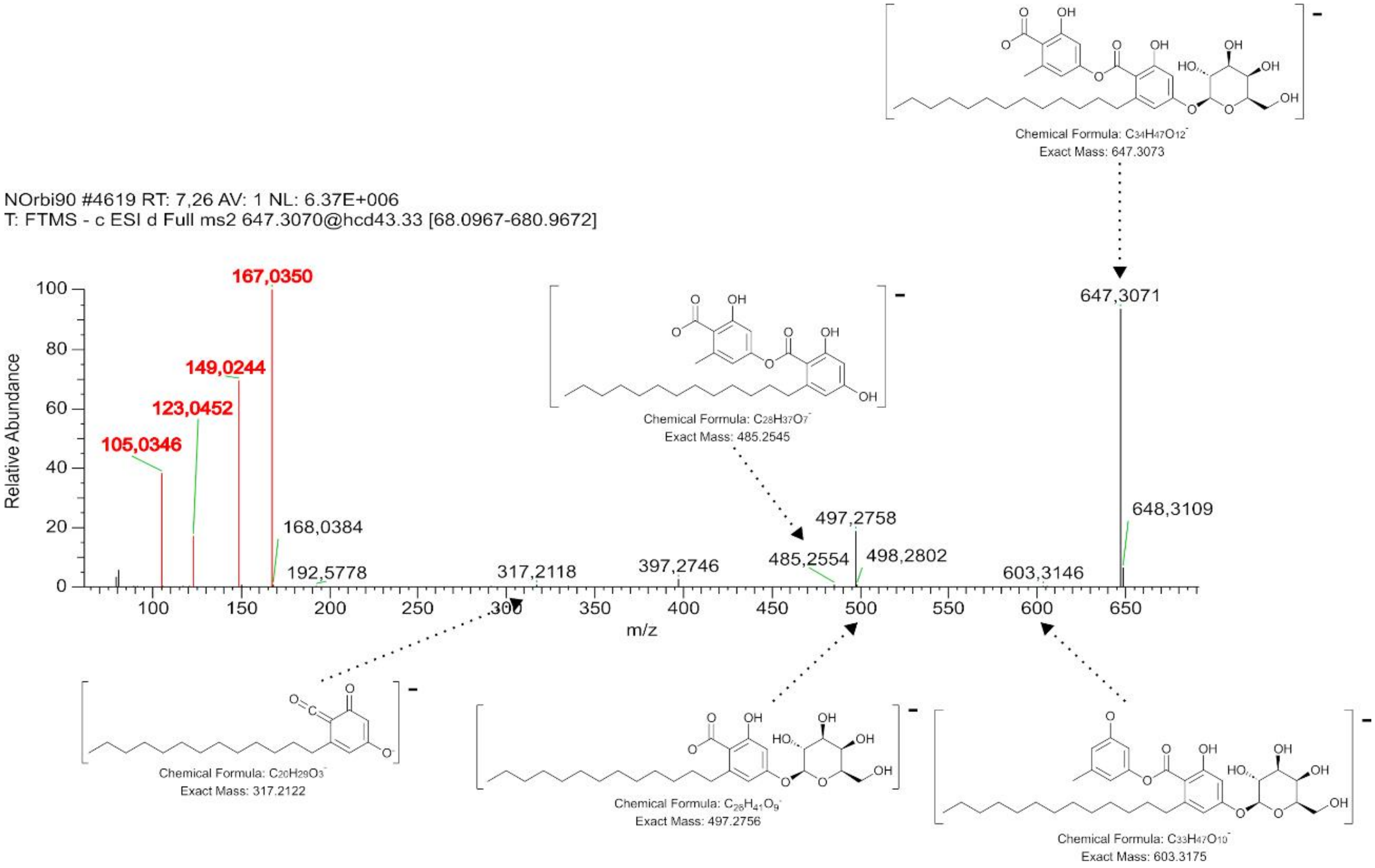

Figure S7. HRESI(−)MS/MS fragmentation pattern of ariestatin A (**3a**).

Fragments highlighted in red are described in Figure S4.

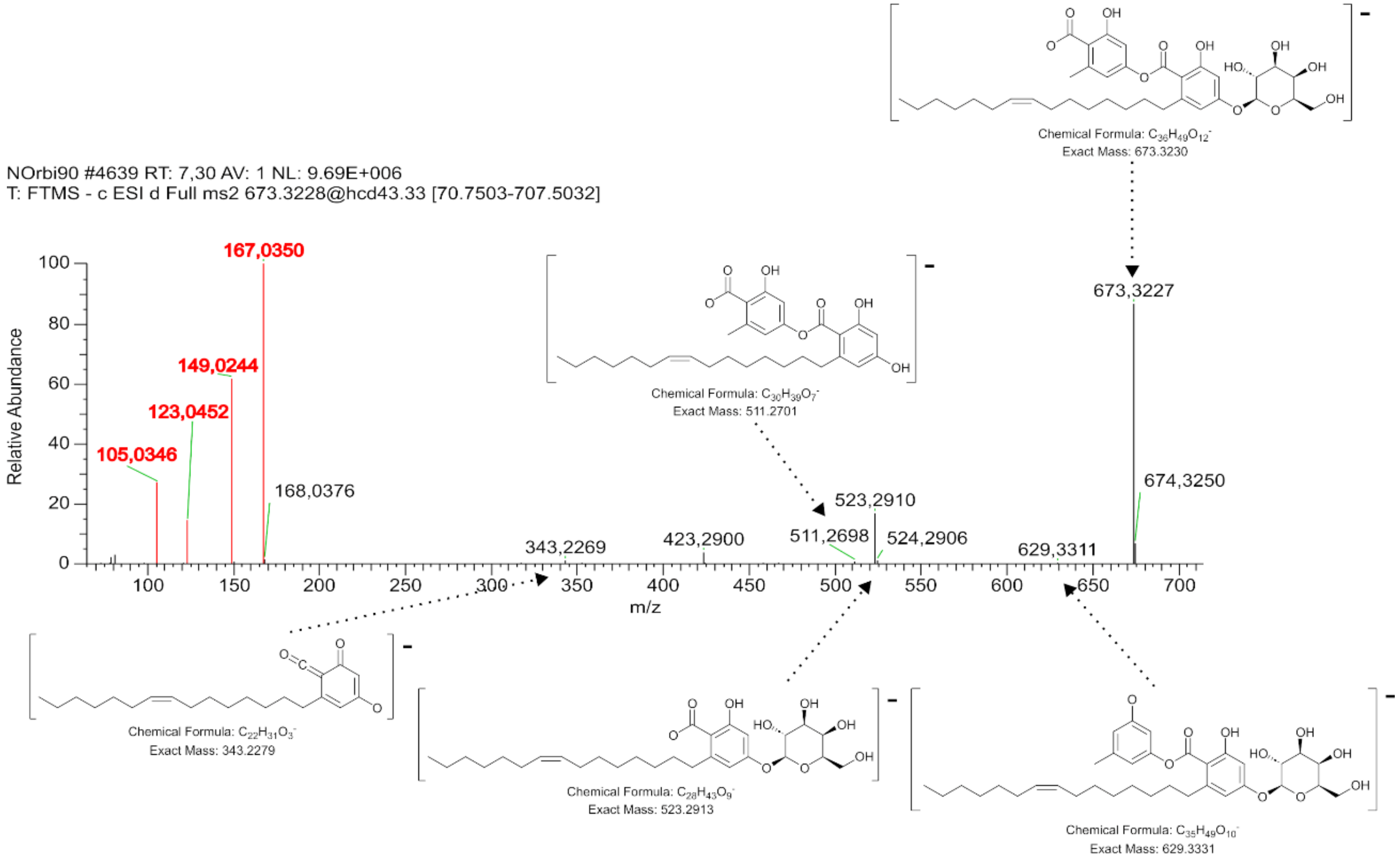

Figure S8. HRESI(−)MS/MS fragmentation pattern of capricostatin A (**4a**).

Fragments highlighted in red are described in Figure S4.

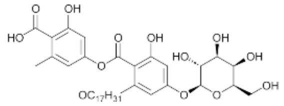

7a

Chemical Formula: $C_{38}H_{52}O_{13}$
Exact Mass: 716.3408

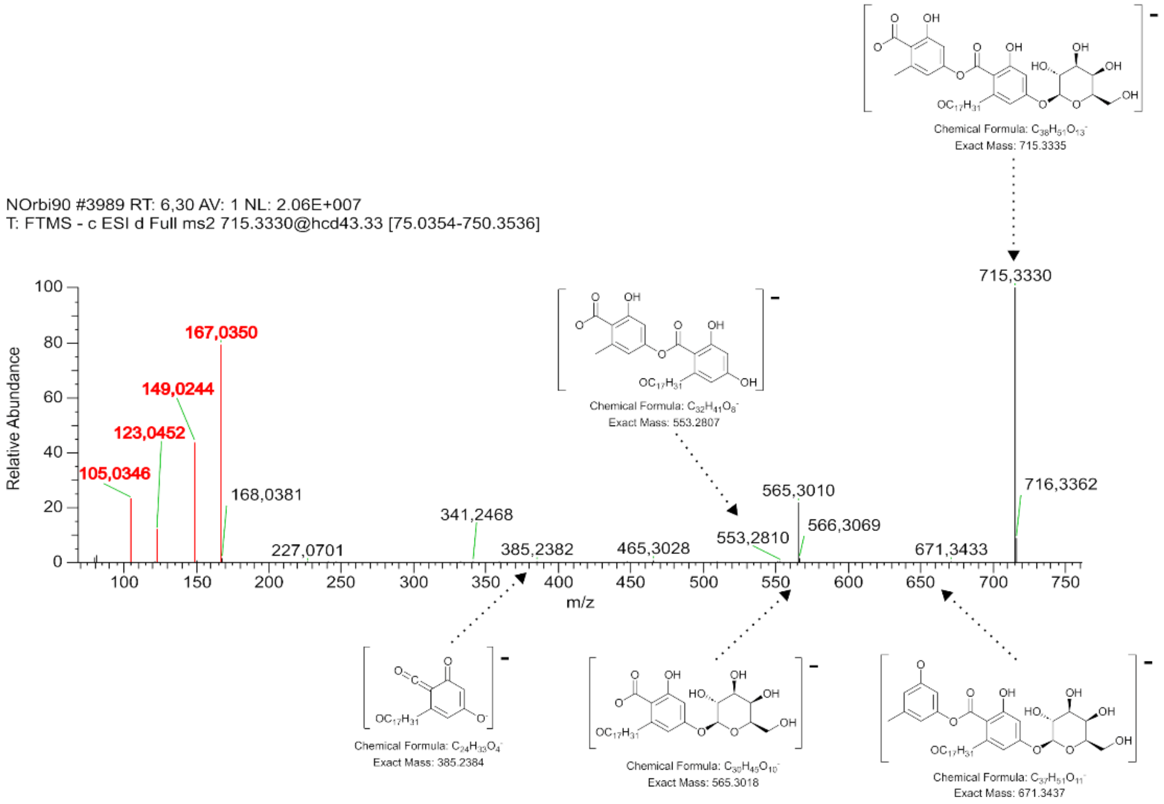

Figure S9. HRESI(−)MS/MS fragmentation pattern of **7a**.

Fragments highlighted in red are described in Figure S4.

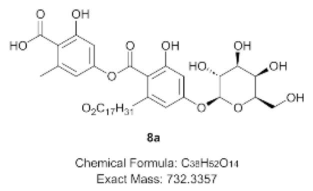

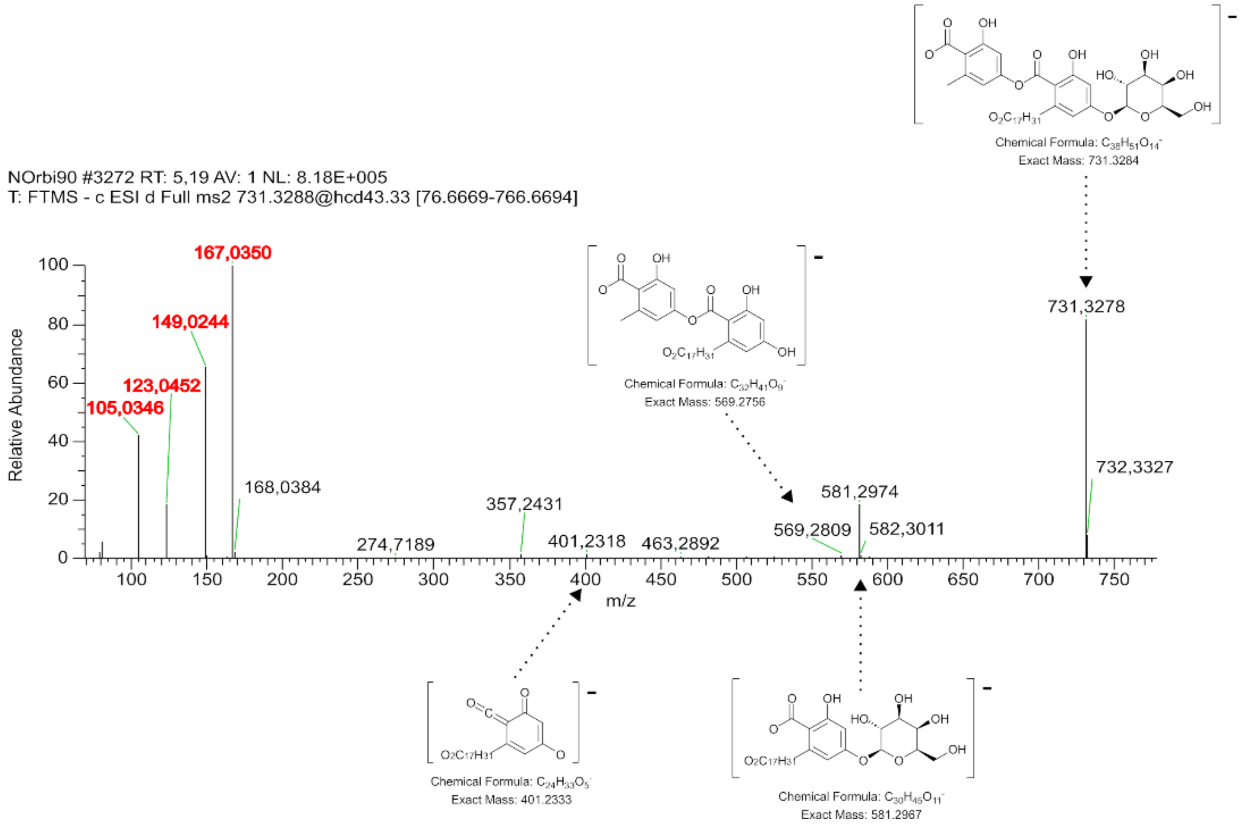

Figure S10. HRESI(−)MS/MS fragmentation pattern of **8a**.

Fragments highlighted in red are described in Figure S4.

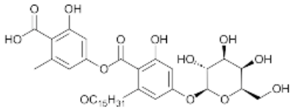

9a

Chemical Formula: $C_{36}H_{52}O_{13}$
Exact Mass: 692.3408

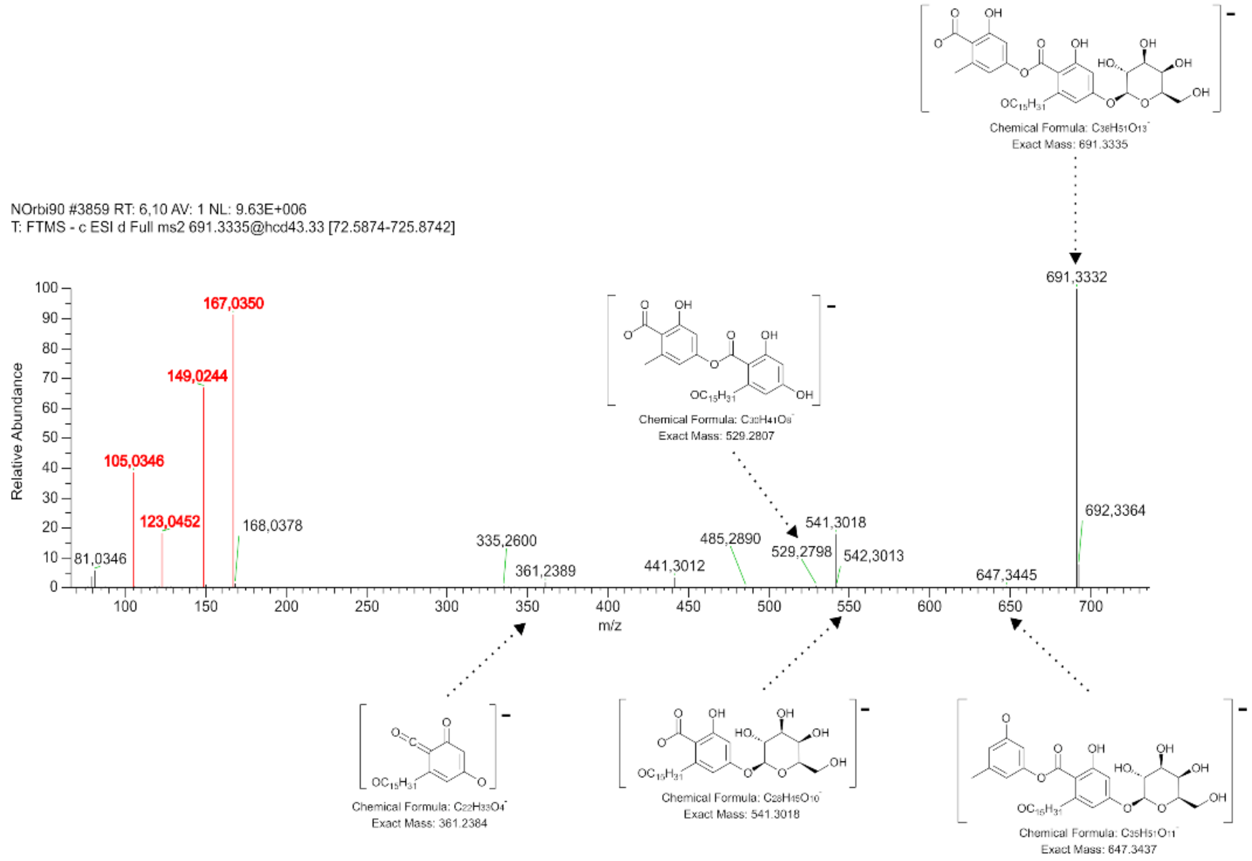

Figure S11. HRESI(−)MS/MS fragmentation pattern of **9a.**

Fragments highlighted in red are described in Figure S4.

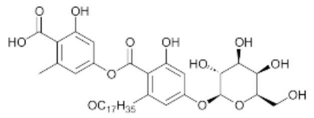

10a

Chemical Formula: $C_{38}H_{56}O_{13}$
Exact Mass: 720.3721

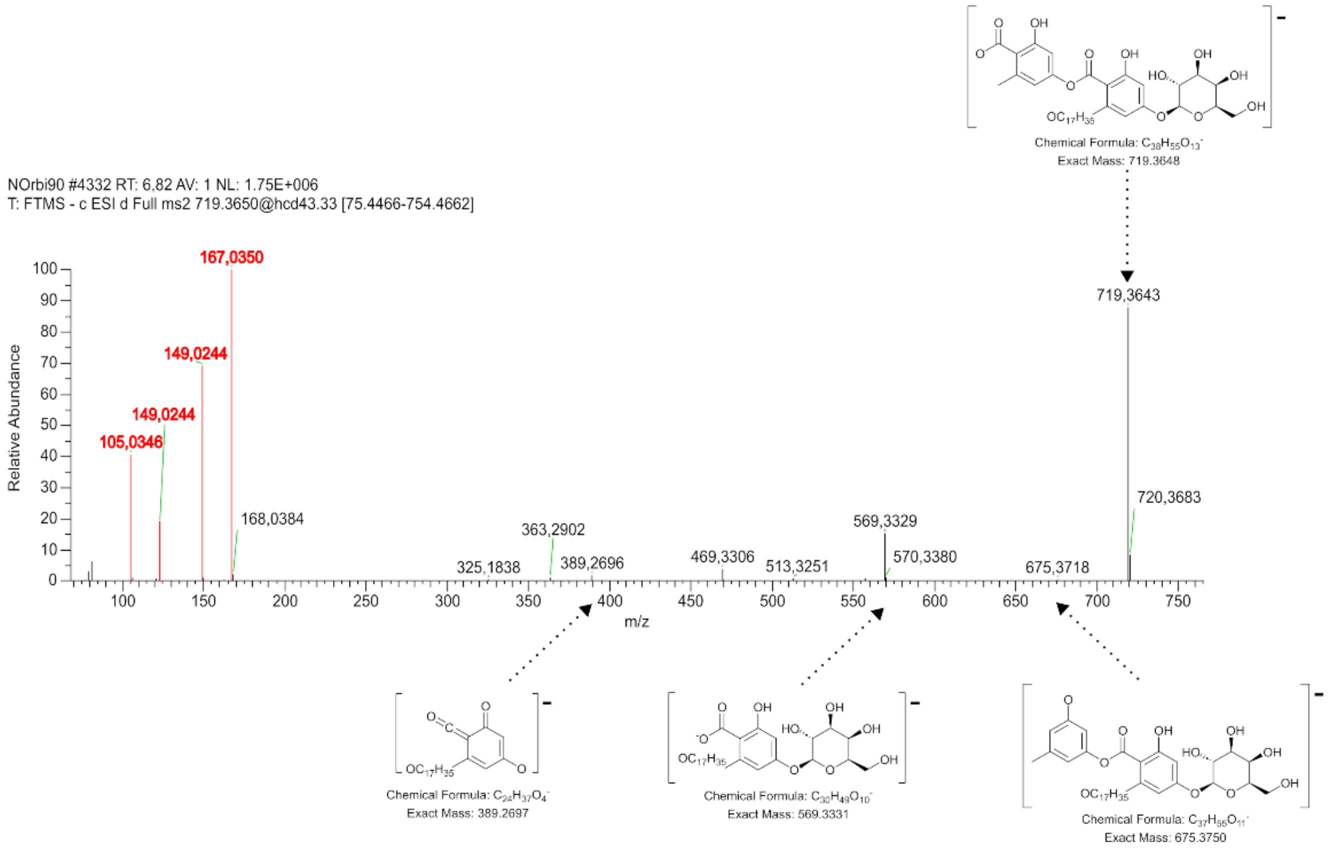

Figure S12. HRESI(−)MS/MS fragmentation pattern of **10a**.

Fragments highlighted in red are described in Figure S4.

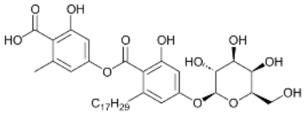

11a

Chemical Formula: $C_{38}H_{50}O_{12}$
Exact Mass: 698.3302

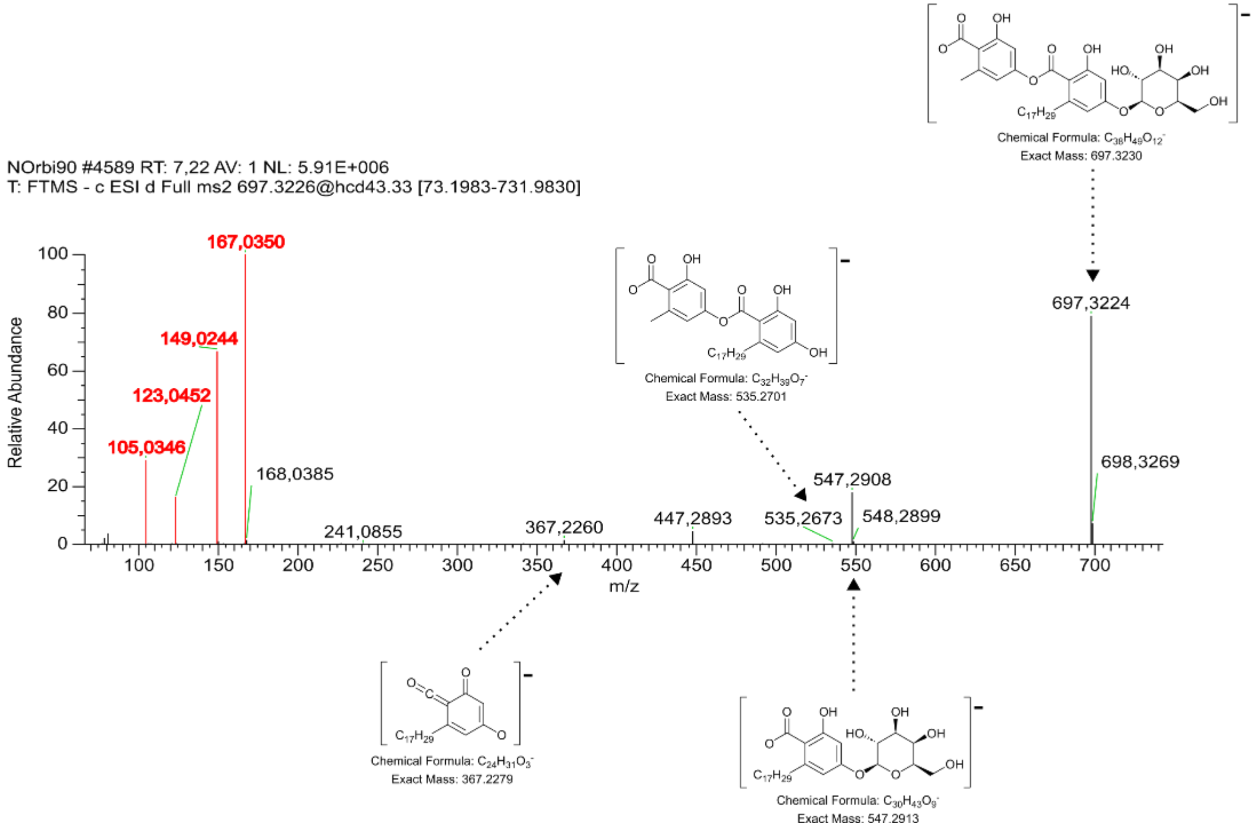

Figure S13. HRESI(−)MS/MS fragmentation pattern of **11a**.

Fragments highlighted in red are described in Figure S4.

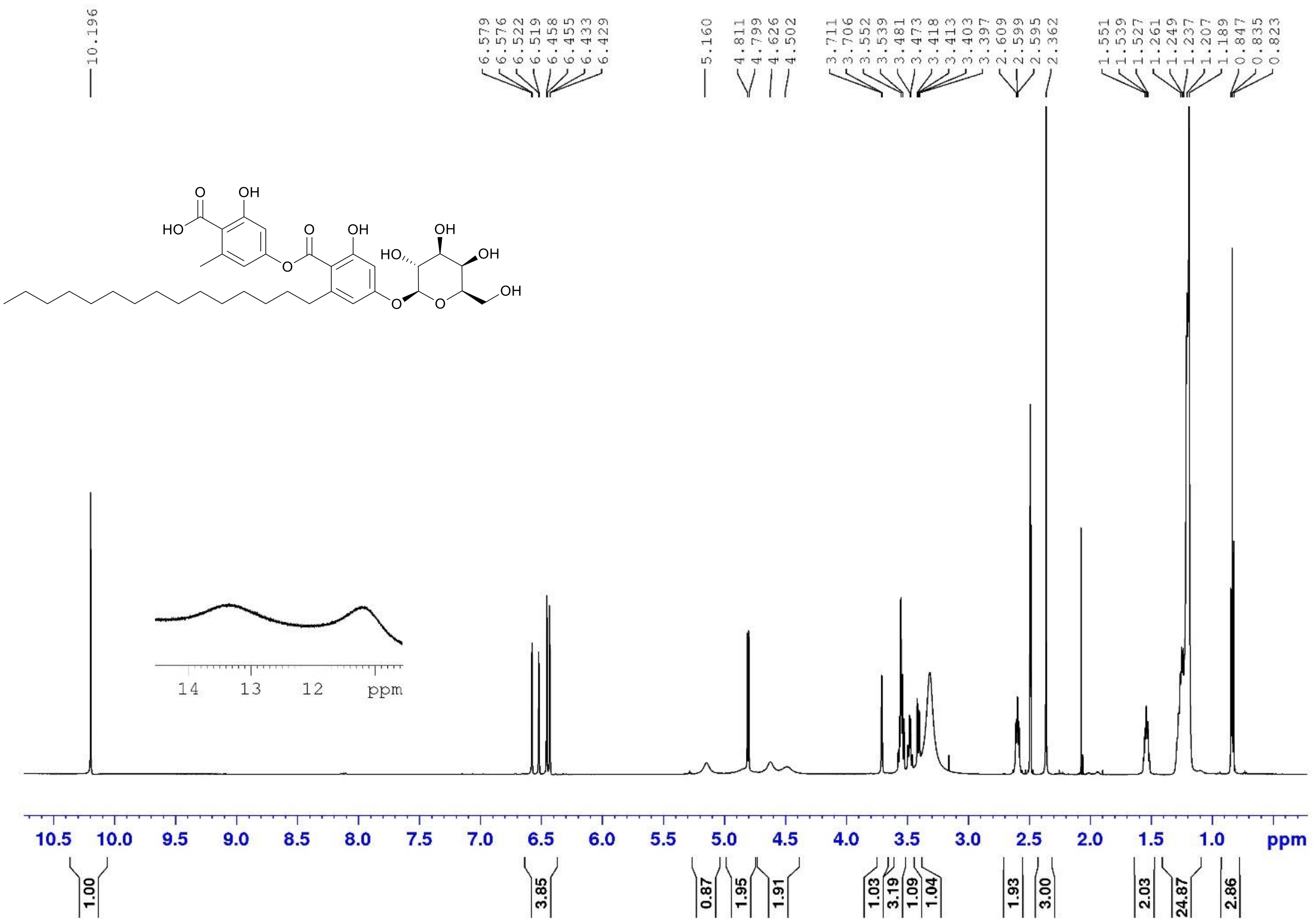

Figure S14. ^{1}H NMR spectrum (600 MHz) of aquastatin A (**1a**) in DMSO-d_6

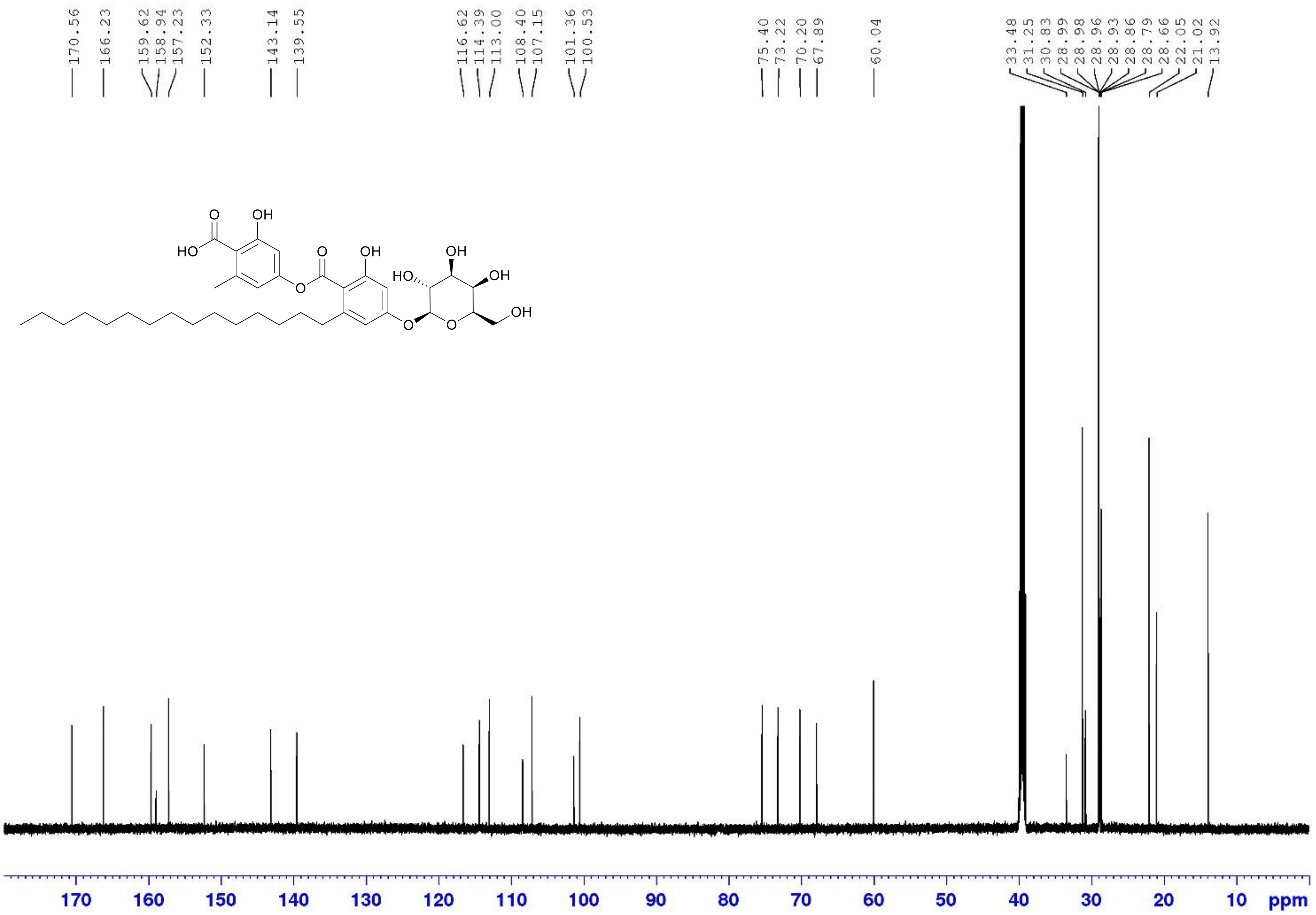

Figure S15. ^{13}C NMR spectrum (150 MHz) of aquastatin A (**1a**) in DMSO-d_6

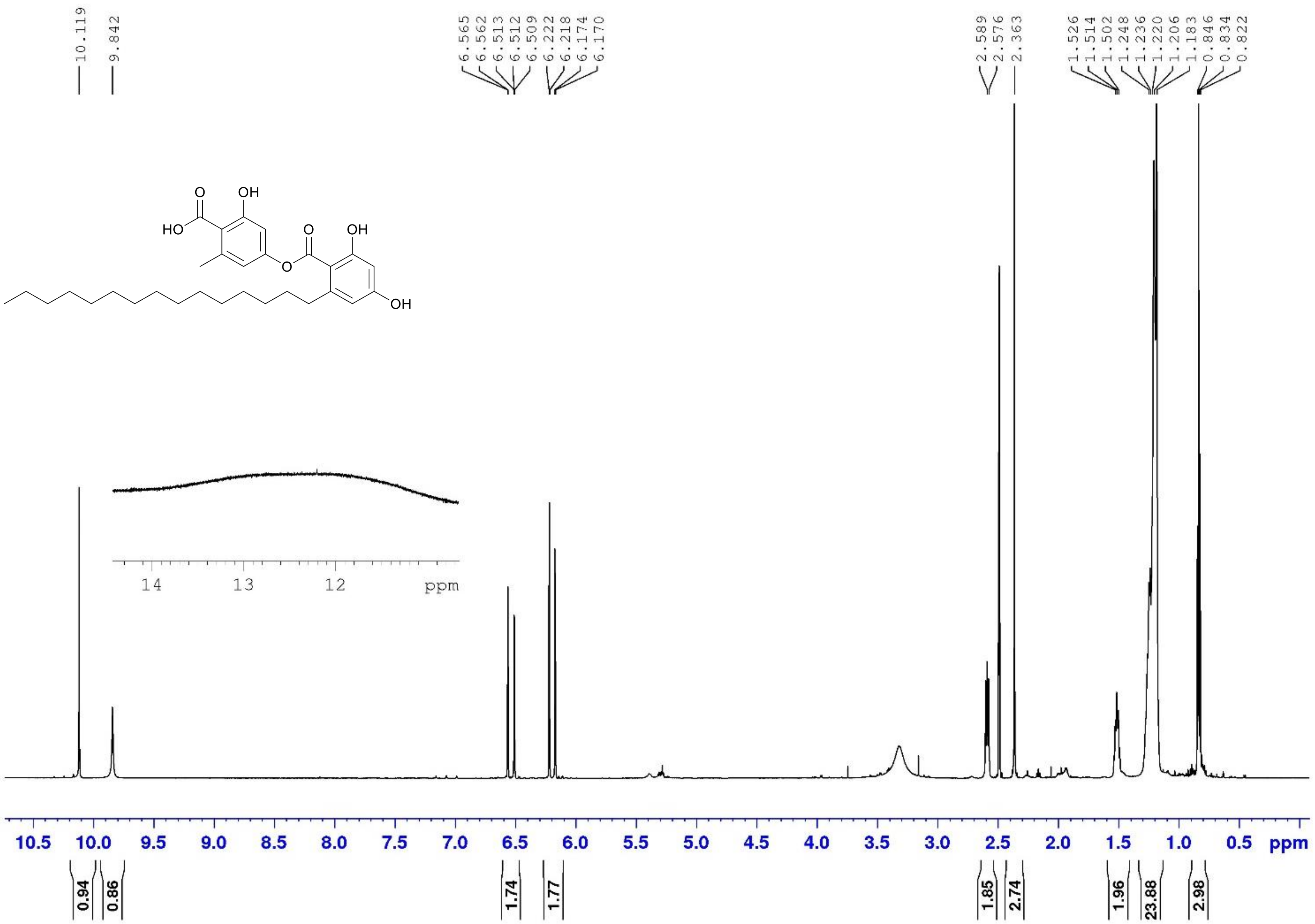

Figure S16. ^{1}H NMR spectrum (600 MHz) of aquastatin B (**1b**) in DMSO-d_6

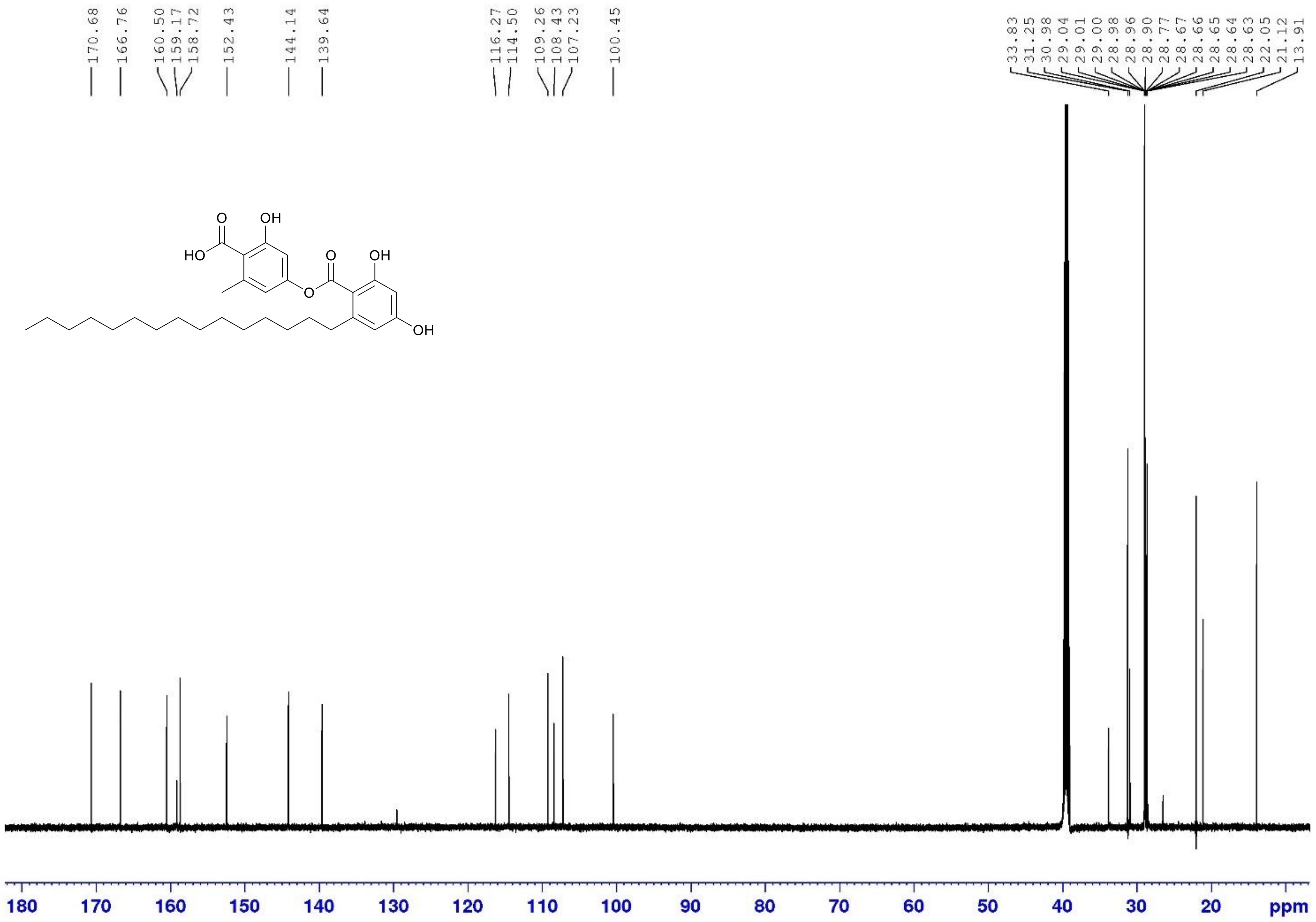

Figure S17. ^{13}C NMR spectrum (150 MHz) of aquastatin B (**1b**) in DMSO-d_6

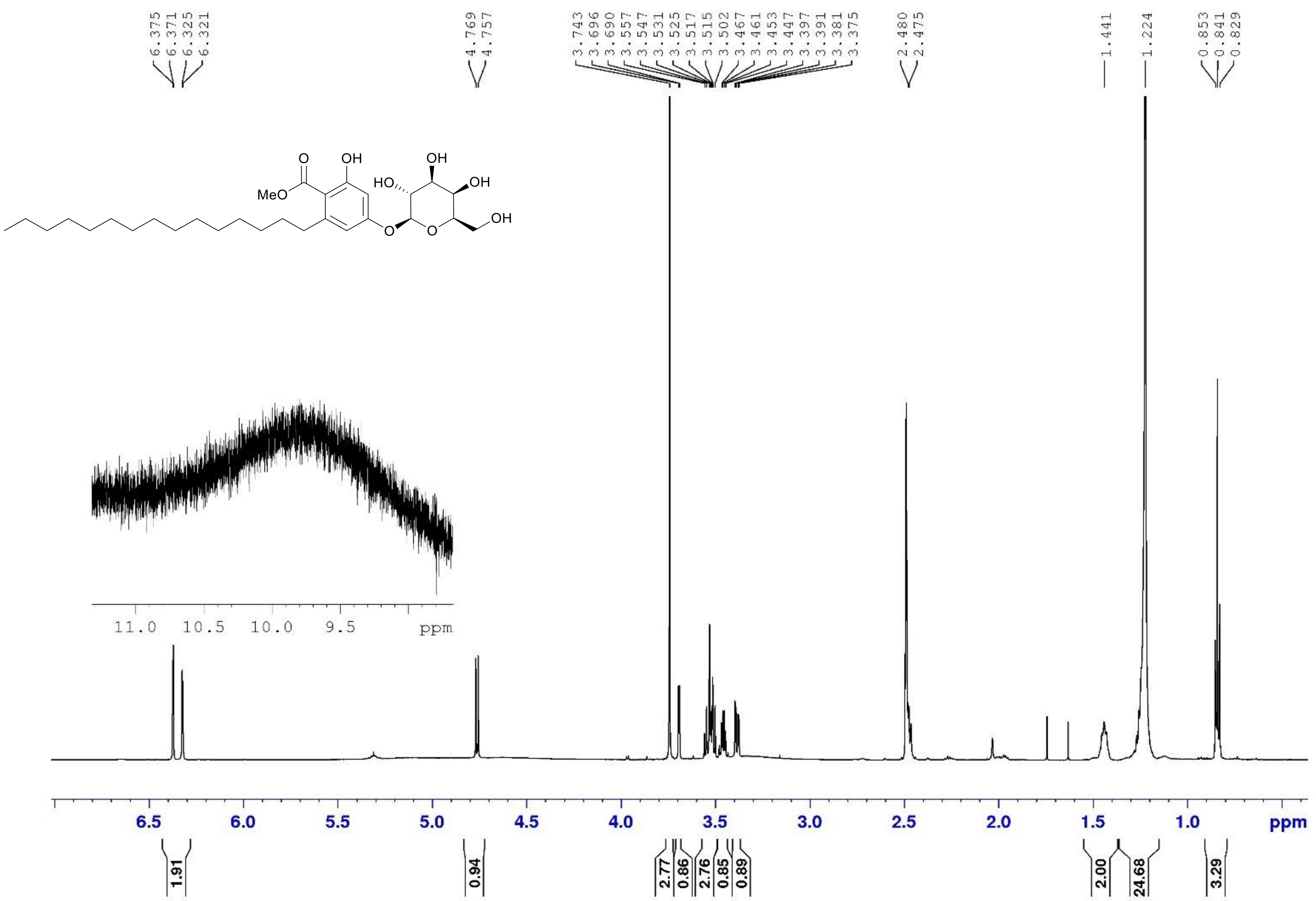

Figure S18. ^1H NMR spectrum (600 MHz) of aquastatin C (**1c**) in DMSO-d_6

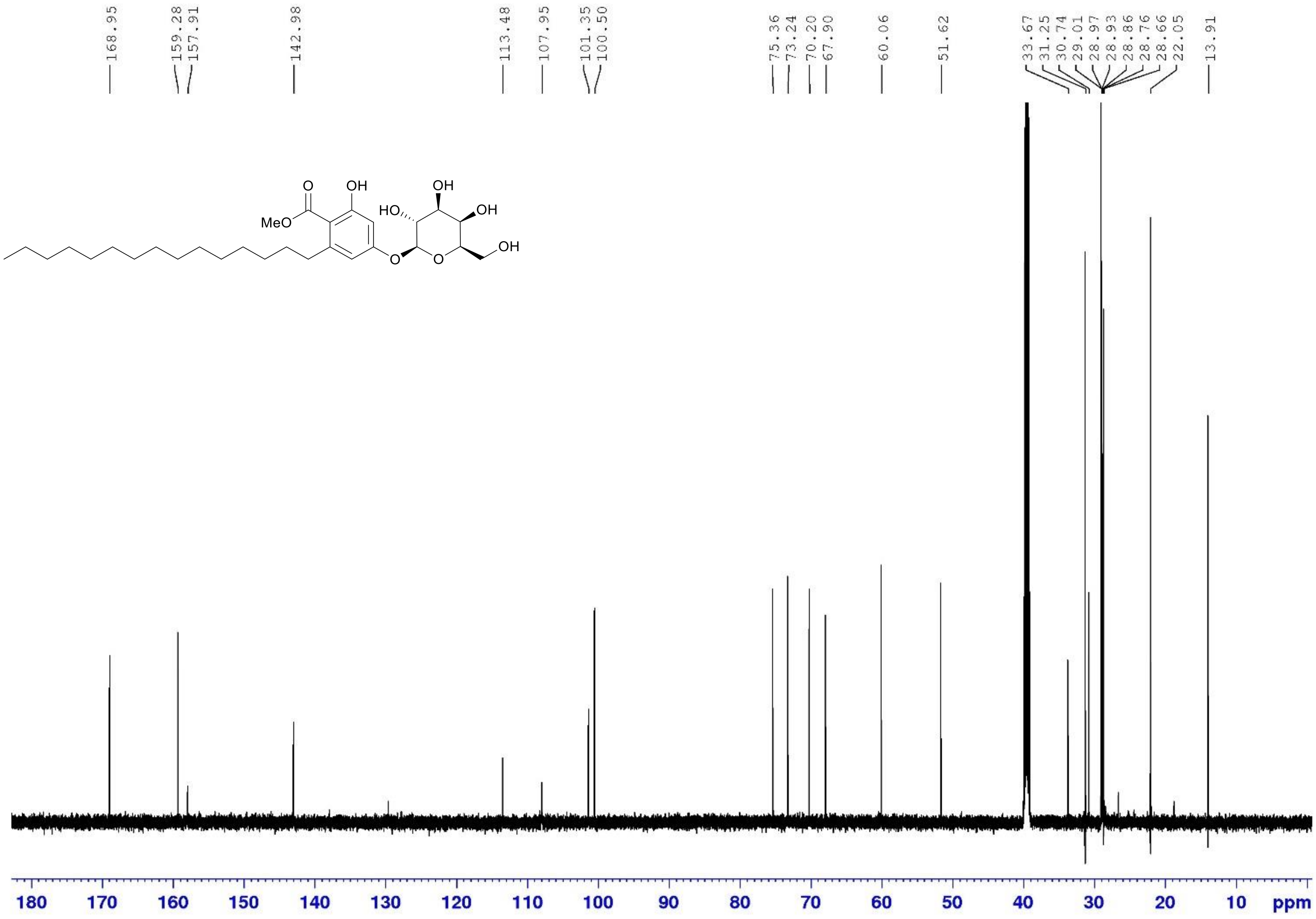

Figure S19. ^{13}C NMR spectrum (150 MHz) of aquastatin C (**1c**) in DMSO-d_6

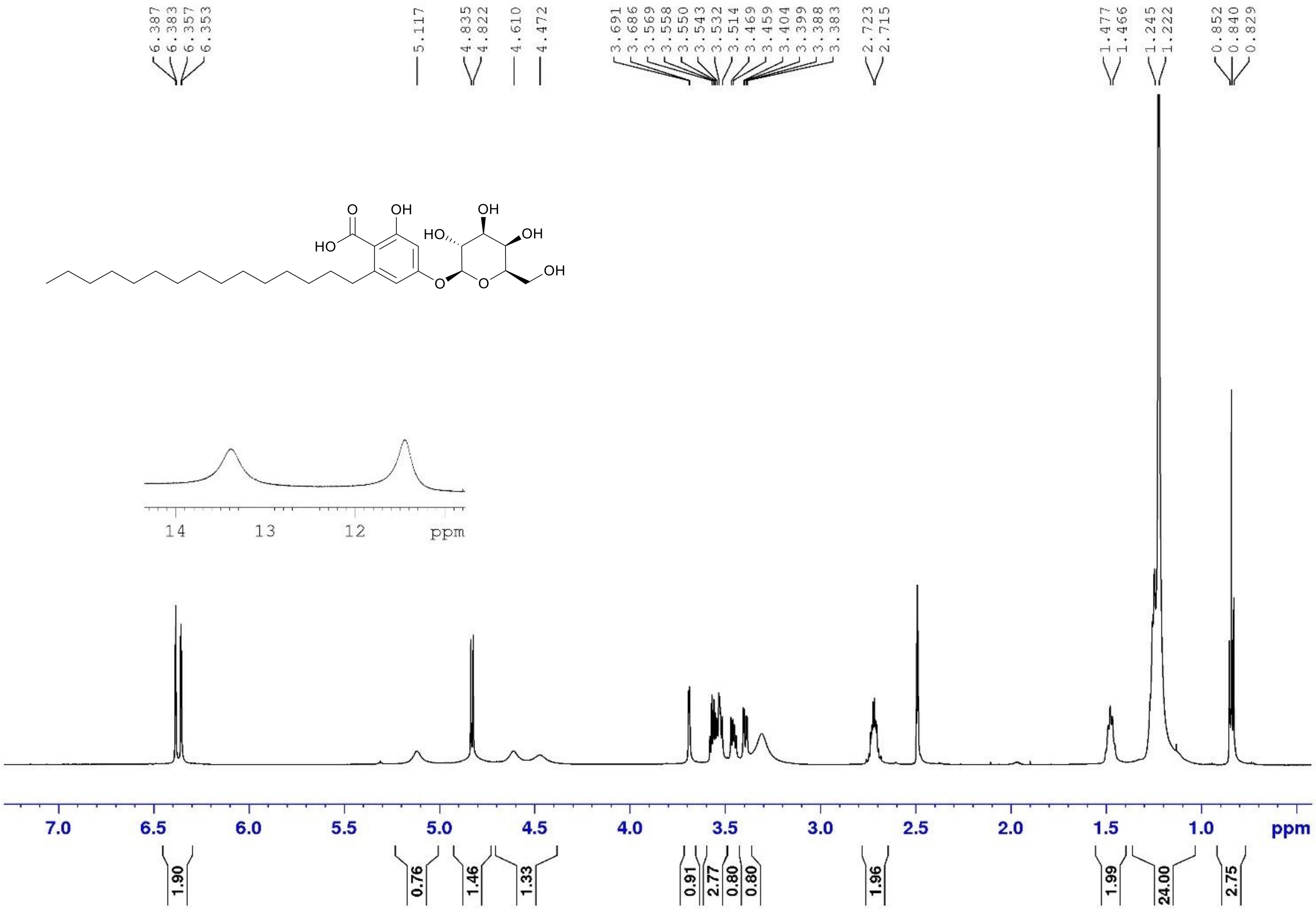

Figure S20. ^{1}H NMR spectrum (600 MHz) of aquastatin D (**1d**) in DMSO-d_6

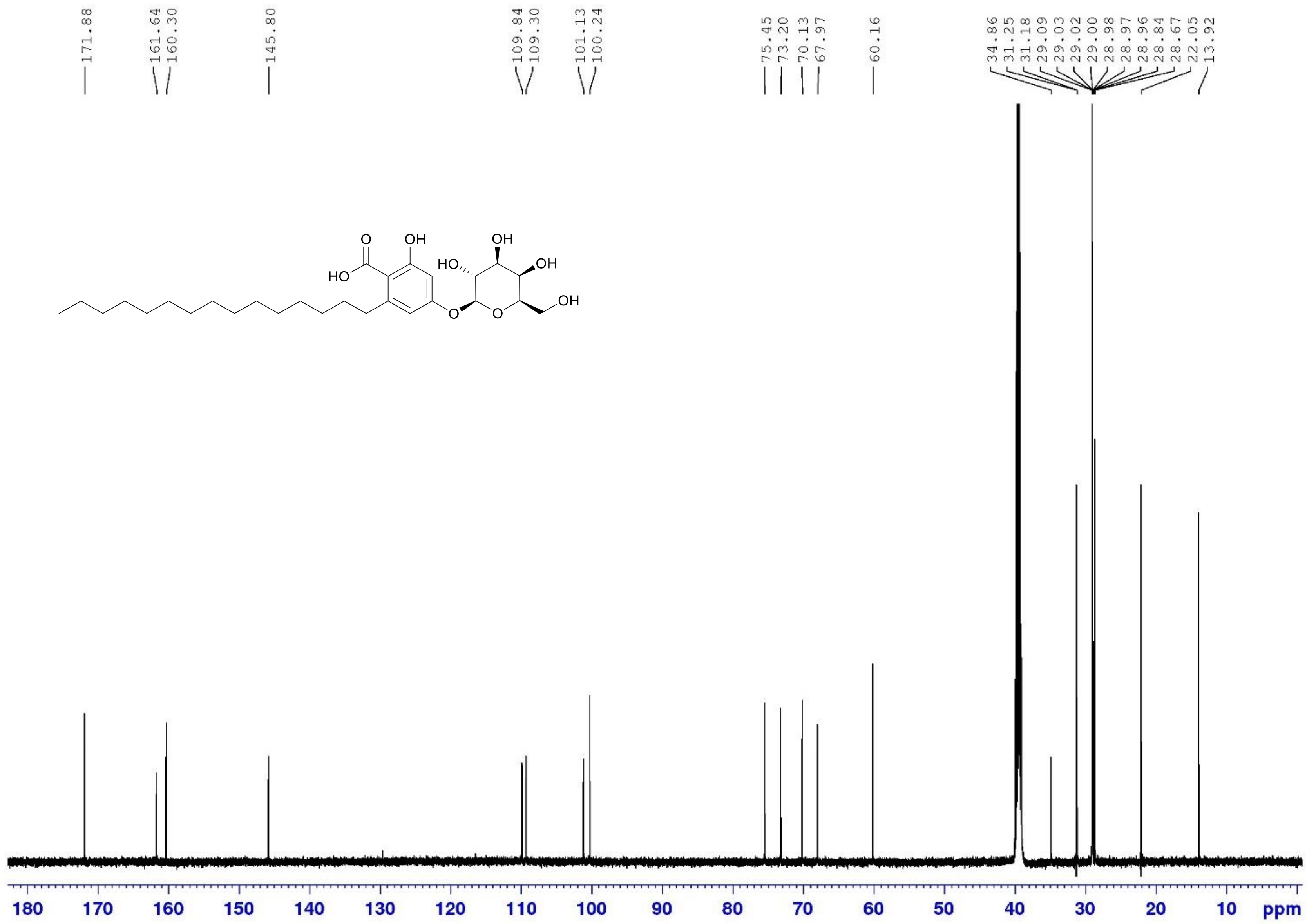

Figure S21. ^{13}C NMR spectrum (150 MHz) of aquastatin D (**1d**) in DMSO-d_6

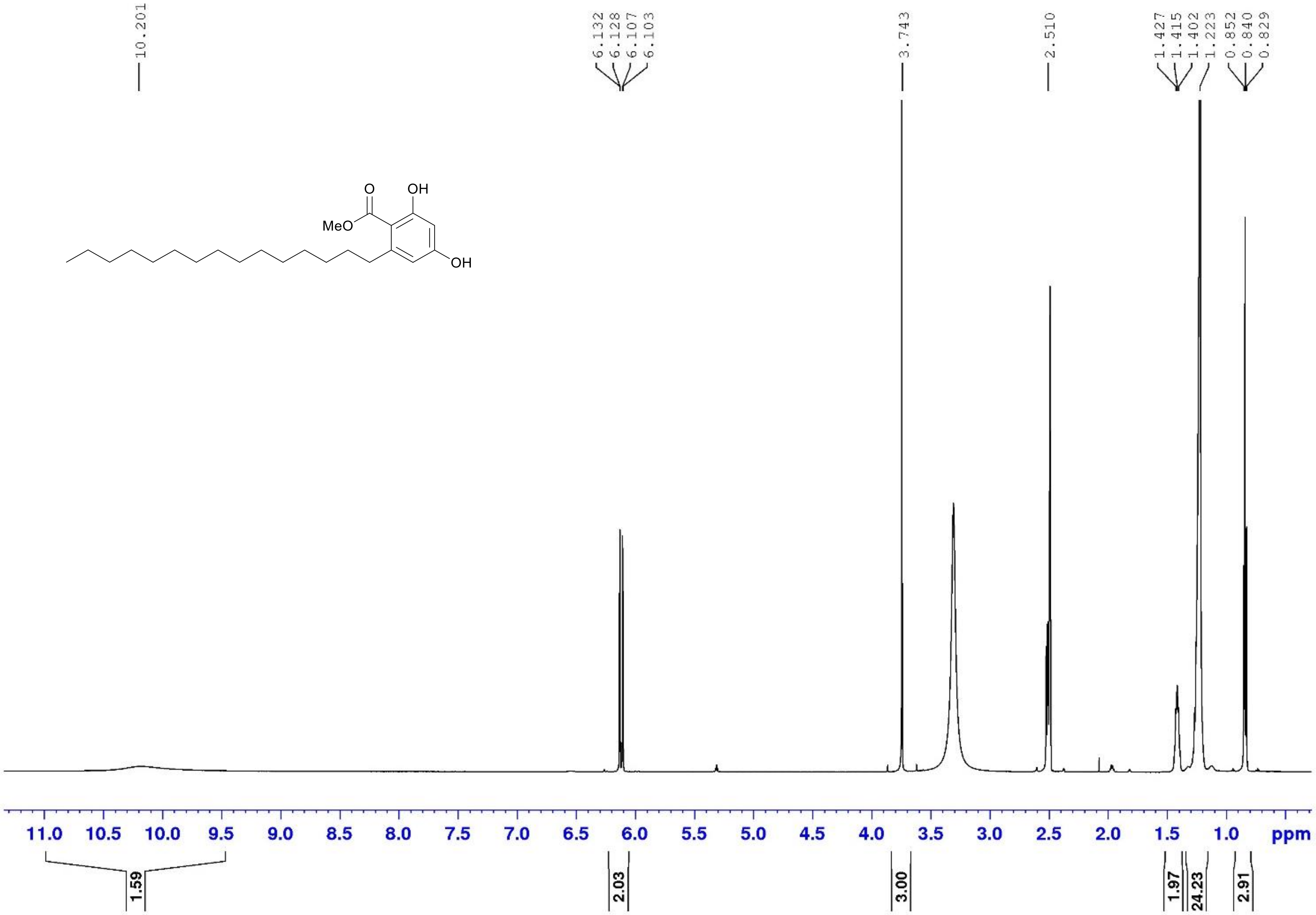

Figure S22. ^{1}H NMR spectrum (600 MHz) of aquastatin E (**1e**) in DMSO-d_6

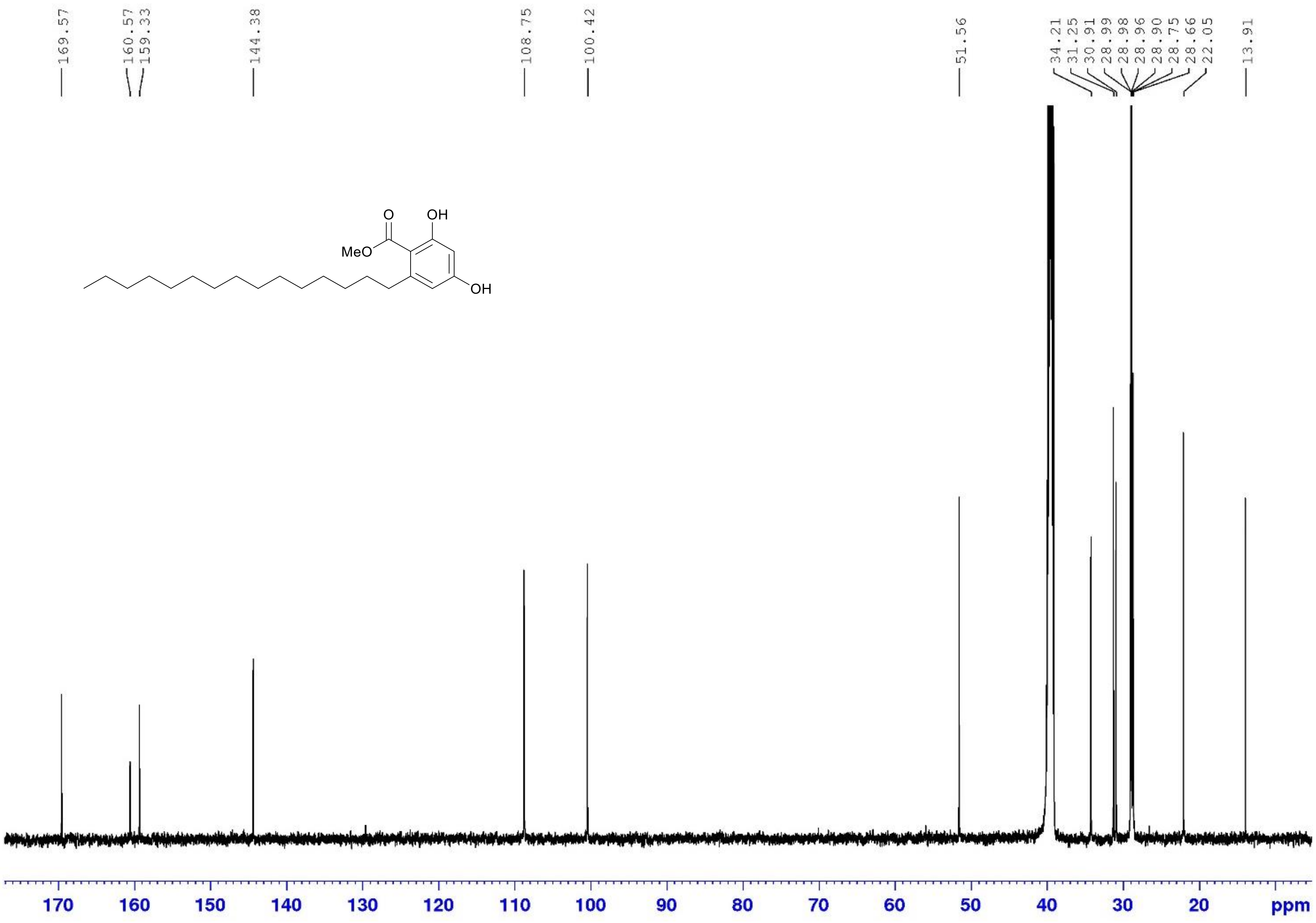

Figure S23. ^{13}C NMR spectrum (150 MHz) of aquastatin E (**1e**) in DMSO-d_6

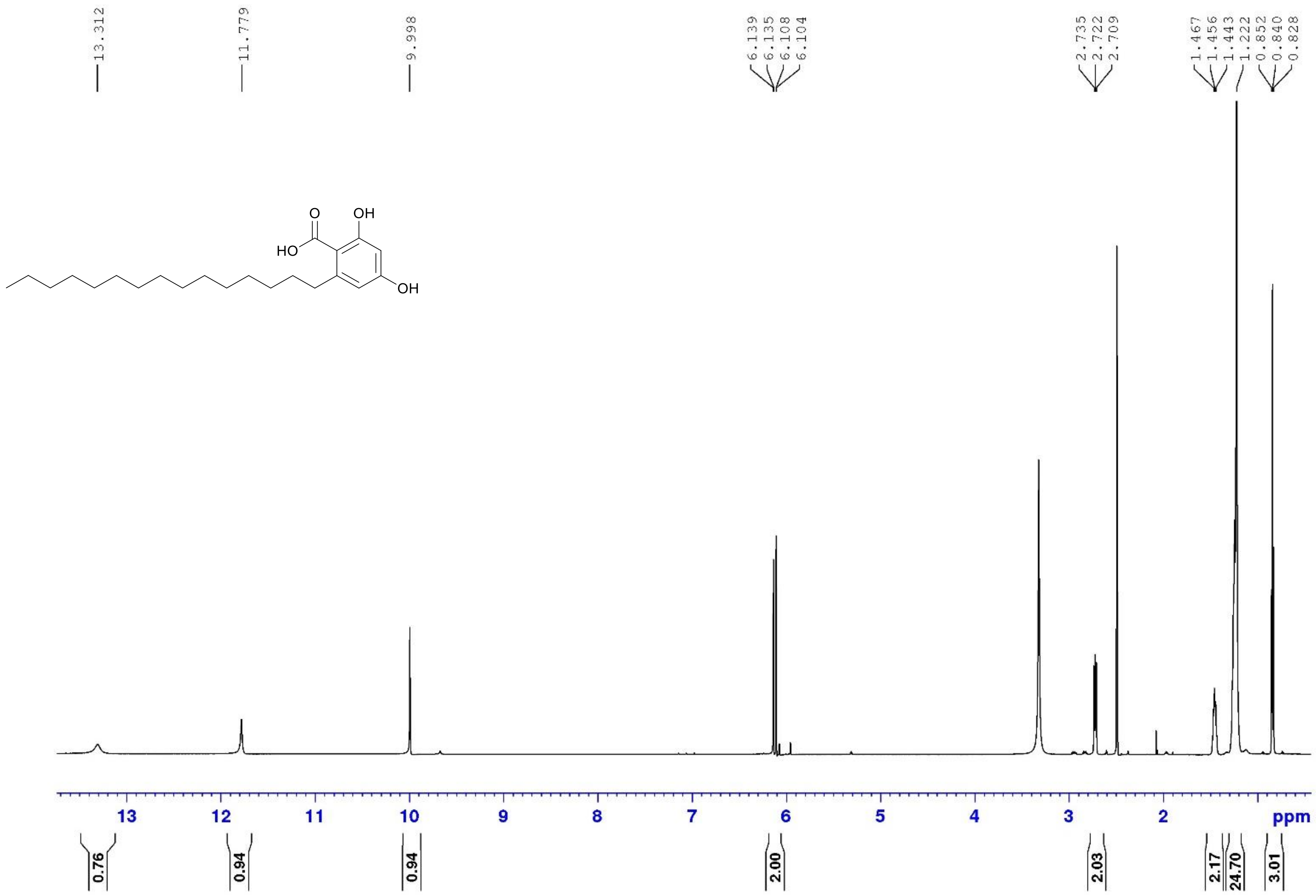

Figure S24. ^{1}H NMR spectrum (600 MHz) of corticiolic acid (**1f**) in DMSO-d_6

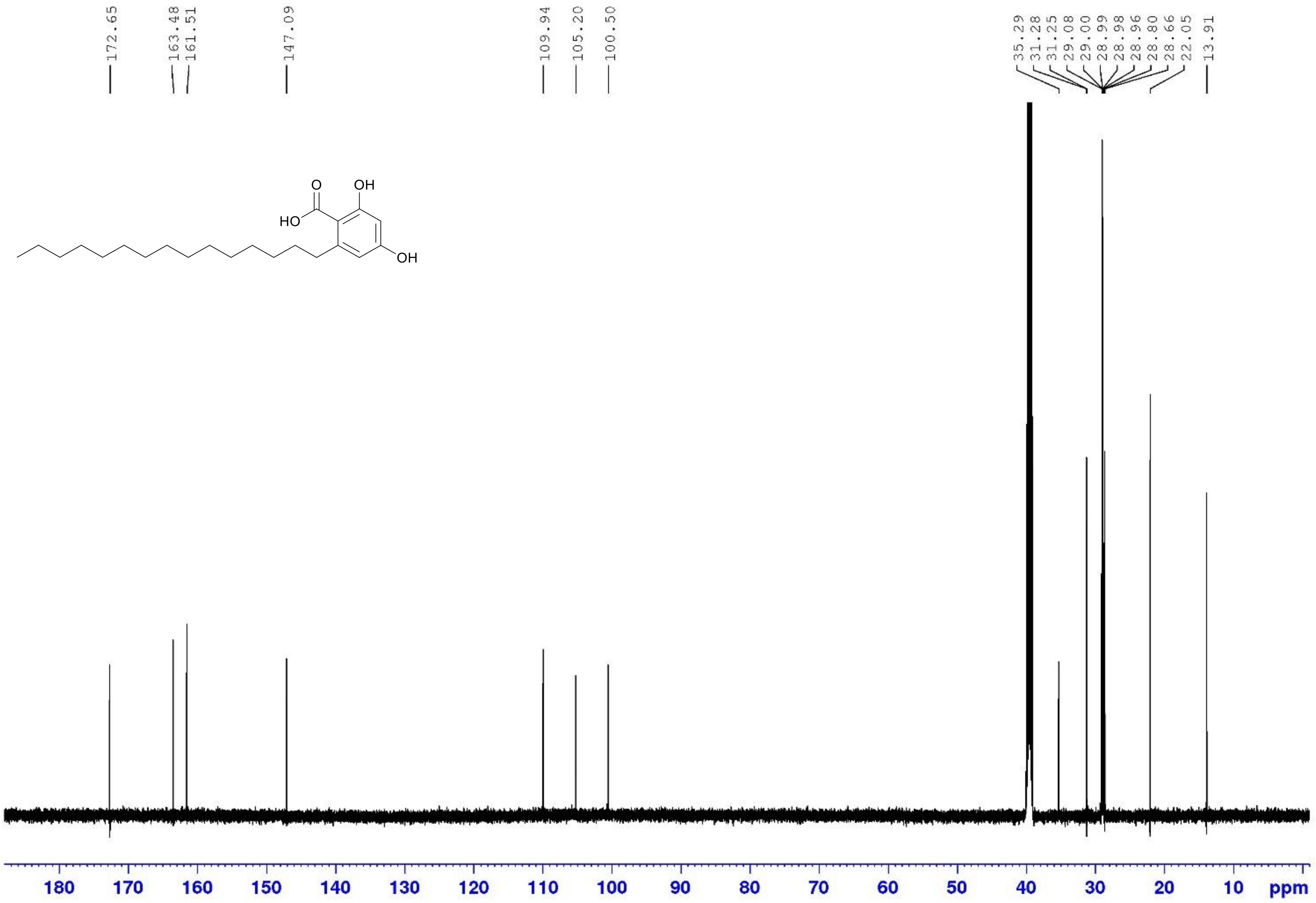

Figure S25. ^{13}C NMR spectrum (150 MHz) of corticiolic acid (**1f**) in DMSO-d_6

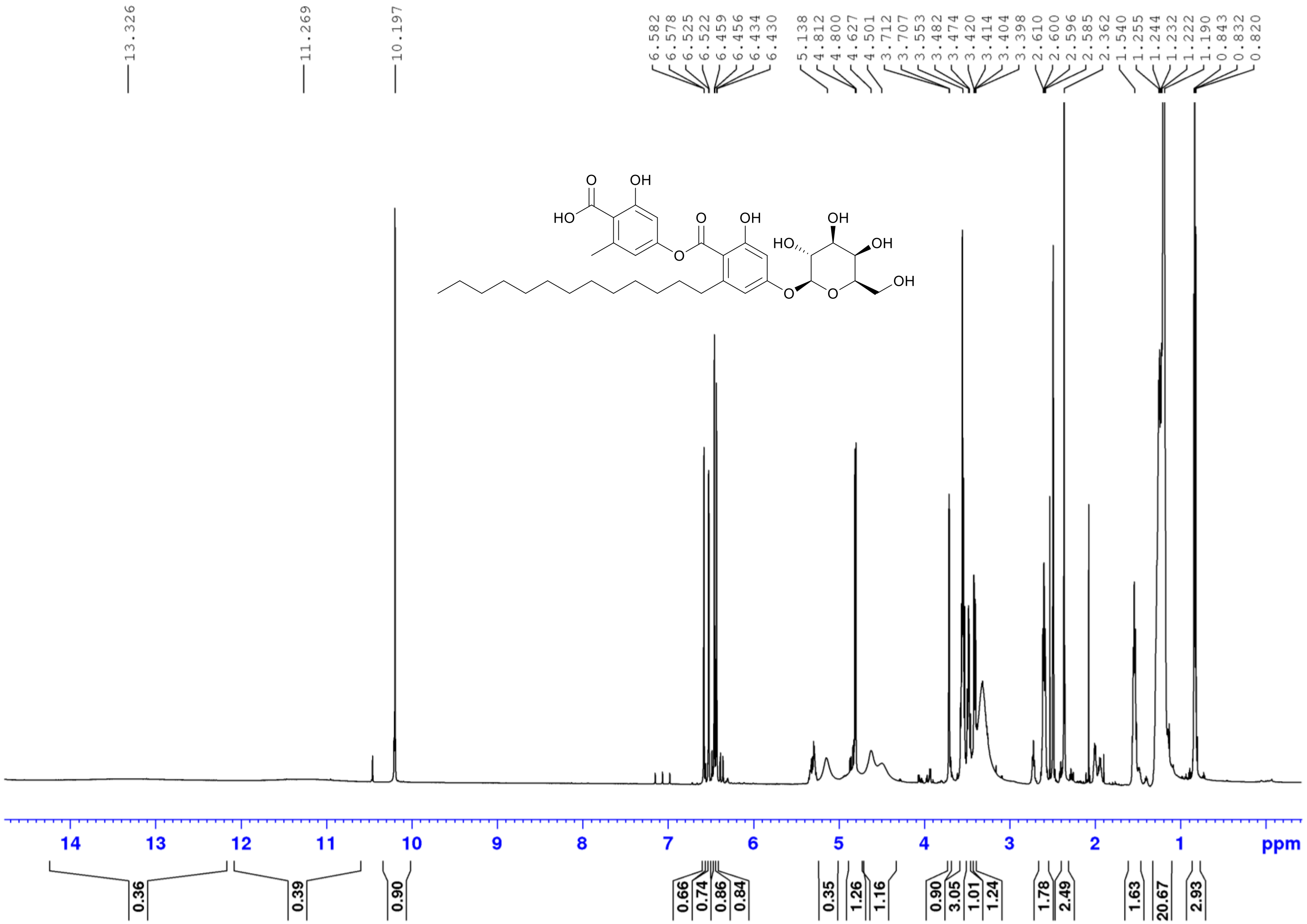

Figure S26. ^{1}H NMR spectrum (600 MHz) of ariestatin A (**3a**) in DMSO-d_6

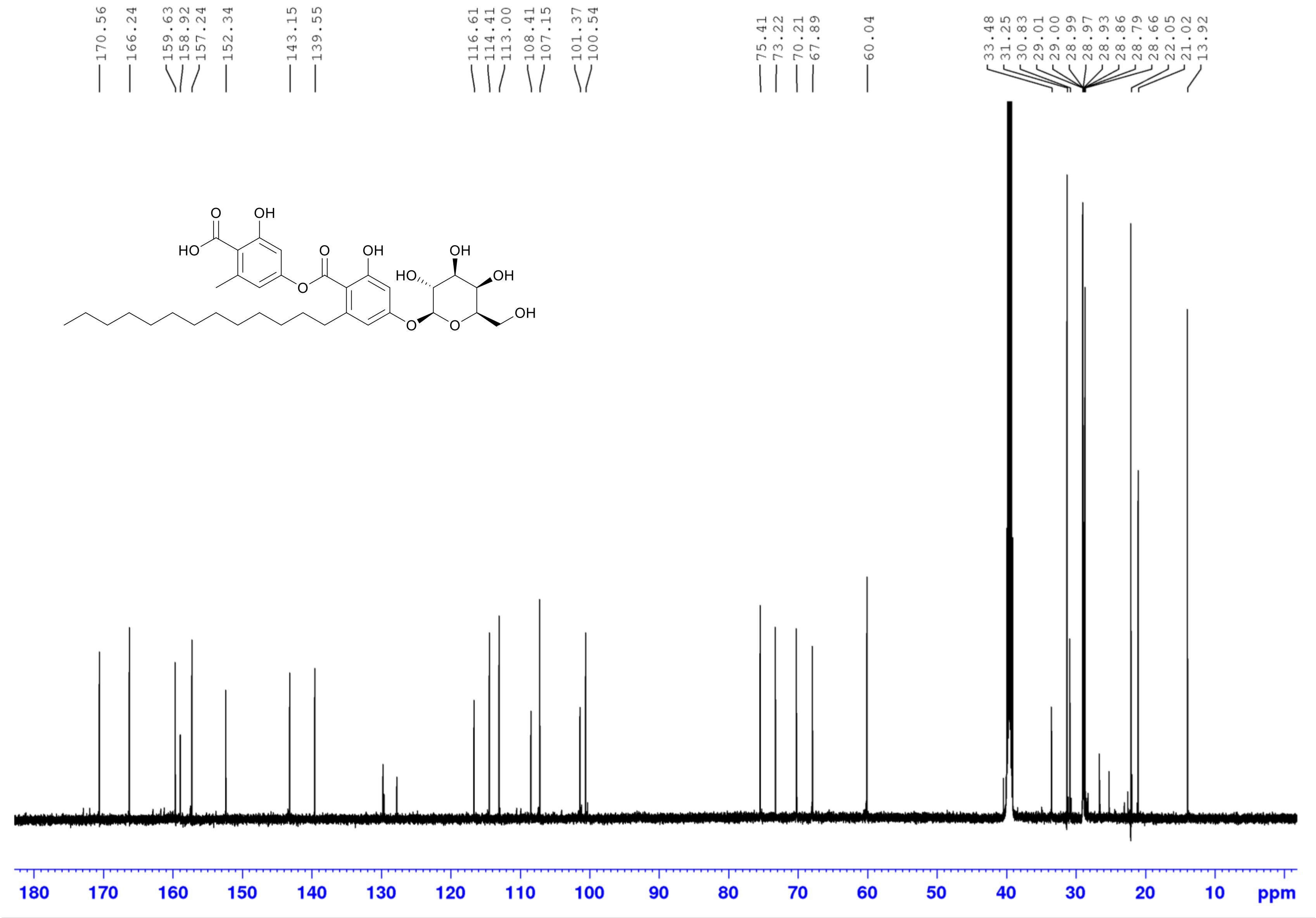

Figure S27. ^{13}C NMR spectrum (150 MHz) of ariestatin A (**3a**) in DMSO-d_6

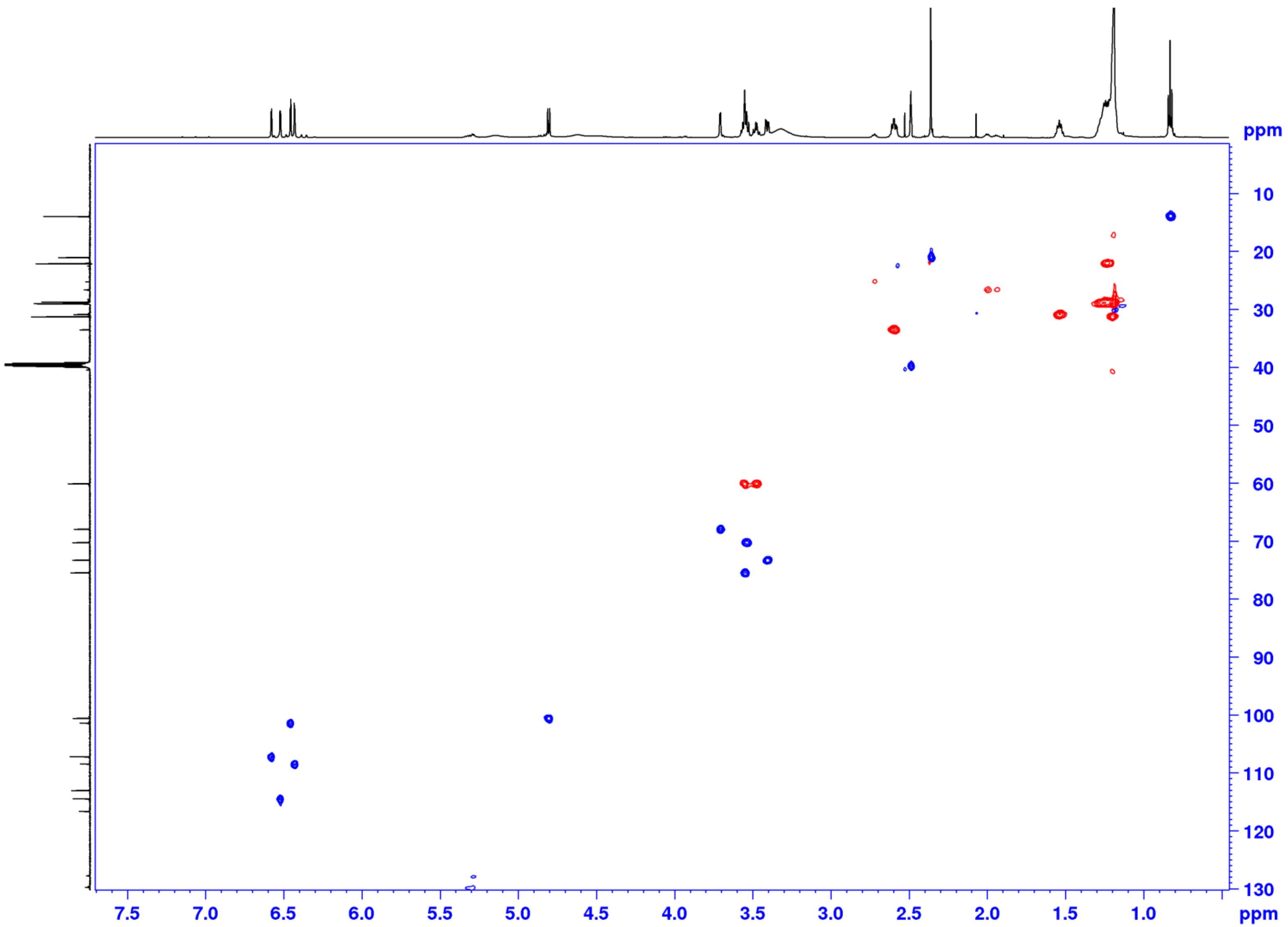

Figure S28. ^{1}H-^{13}C HSQC NMR spectrum (600 MHz) of ariestatin A (**3a**) in DMSO-d_6

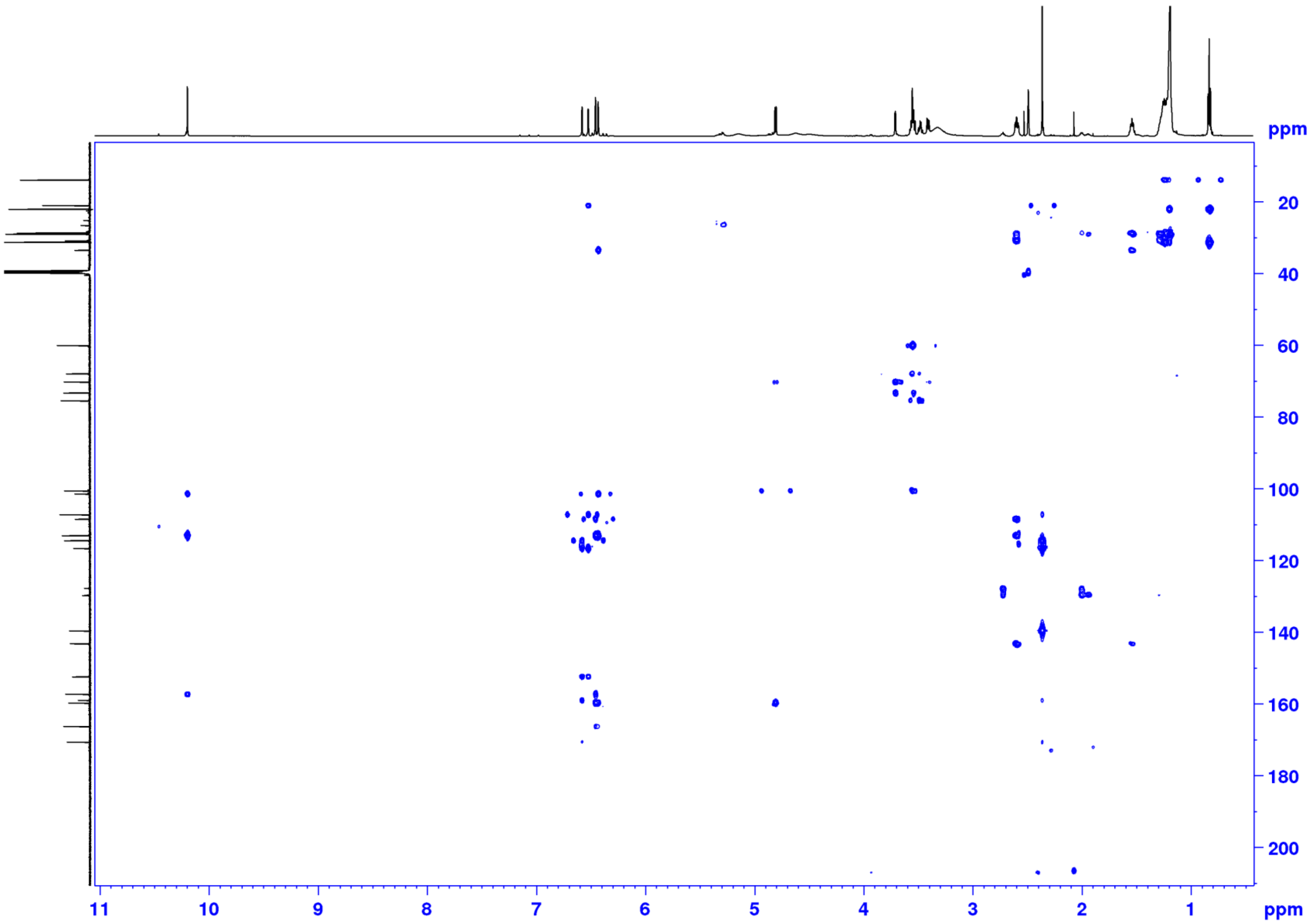

Figure S29. ^{1}H-^{13}C HMBC NMR spectrum (600 MHz) of ariestatin A (**3a**) in DMSO-d_6

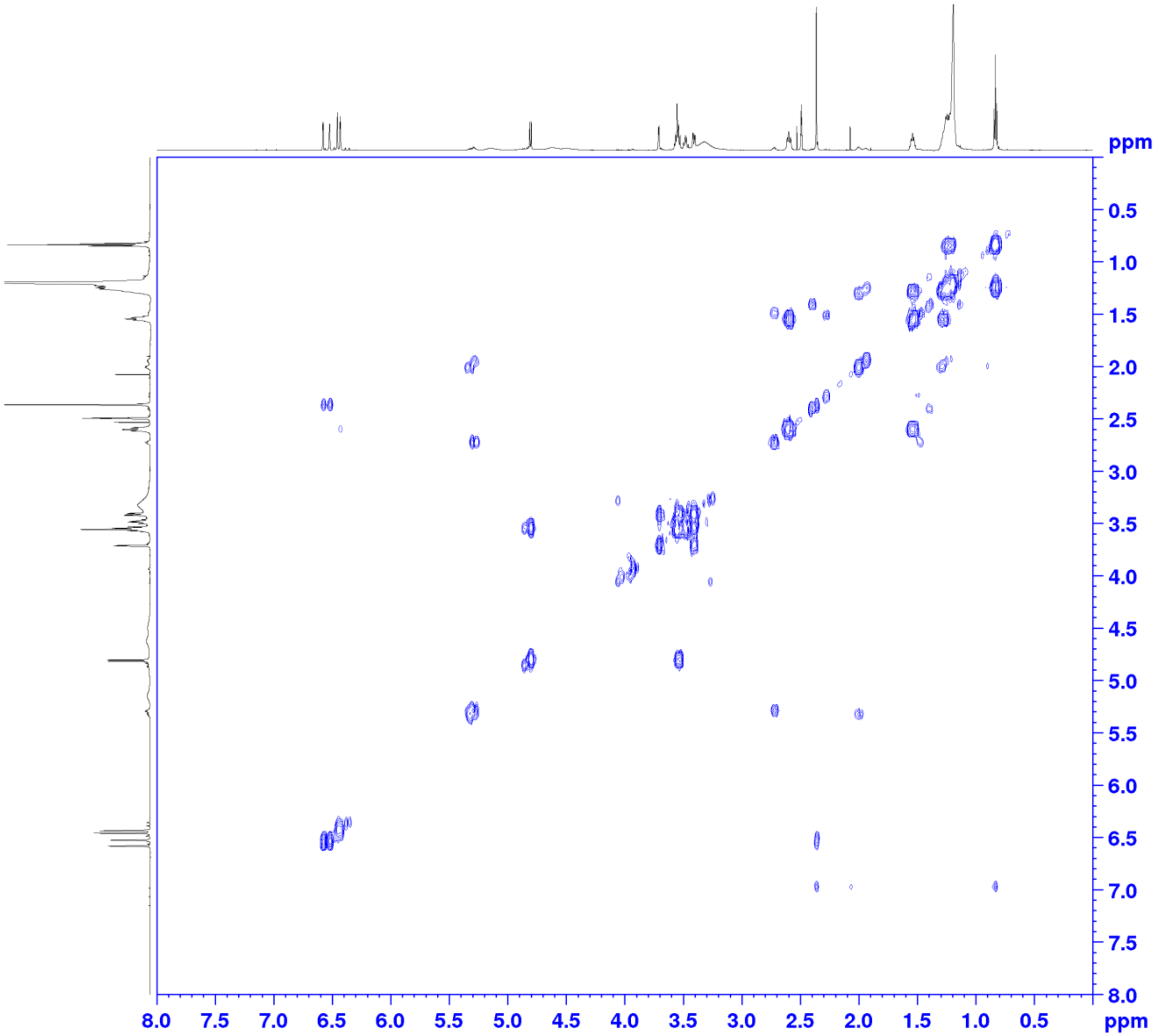

Figure S30. COSY NMR spectrum (600 MHz) of ariestatin A (**3a**) in DMSO-d_6

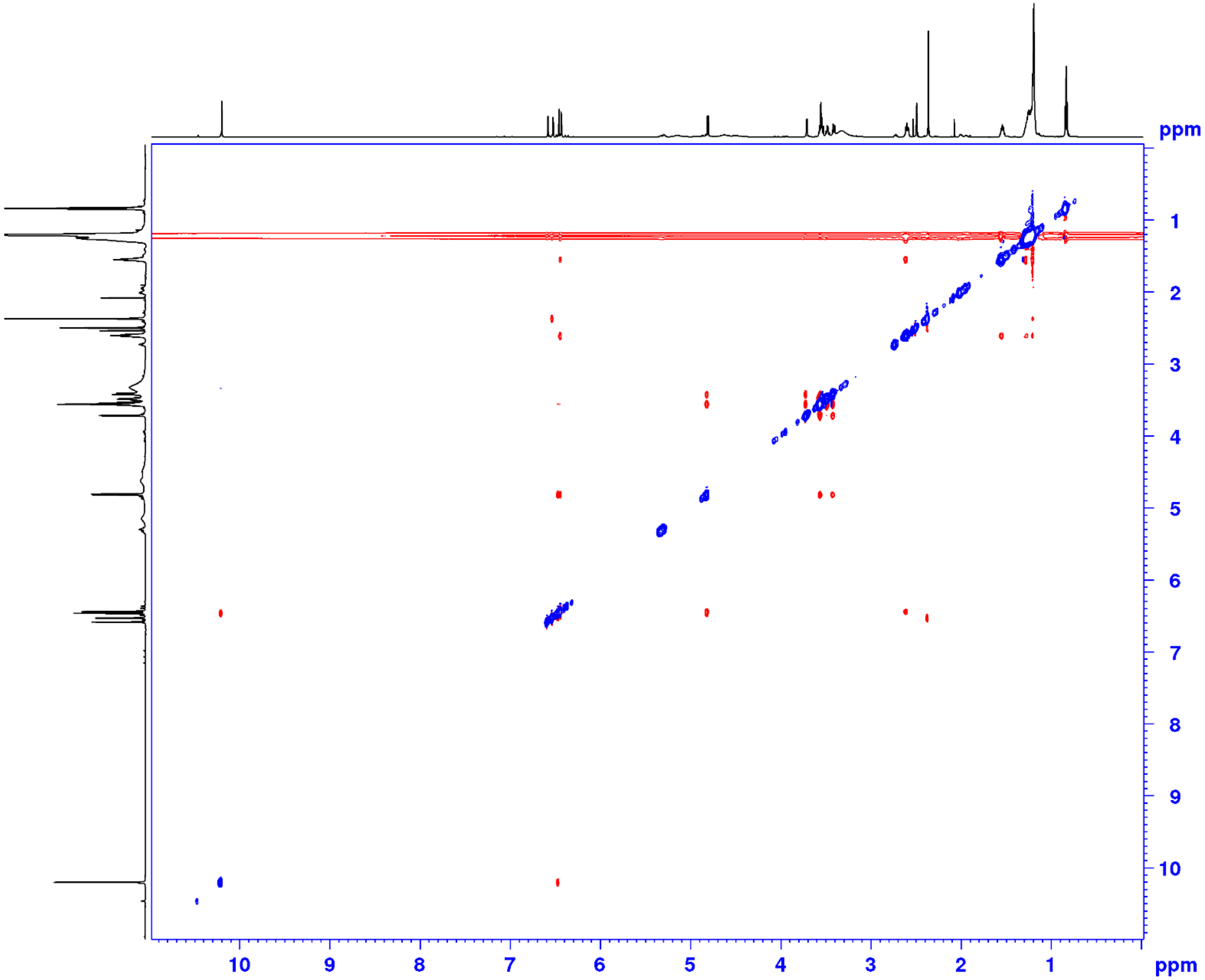

Figure S31. ROESY NMR spectrum (150 MHz) of ariestatin A (**3a**) in DMSO-d_6

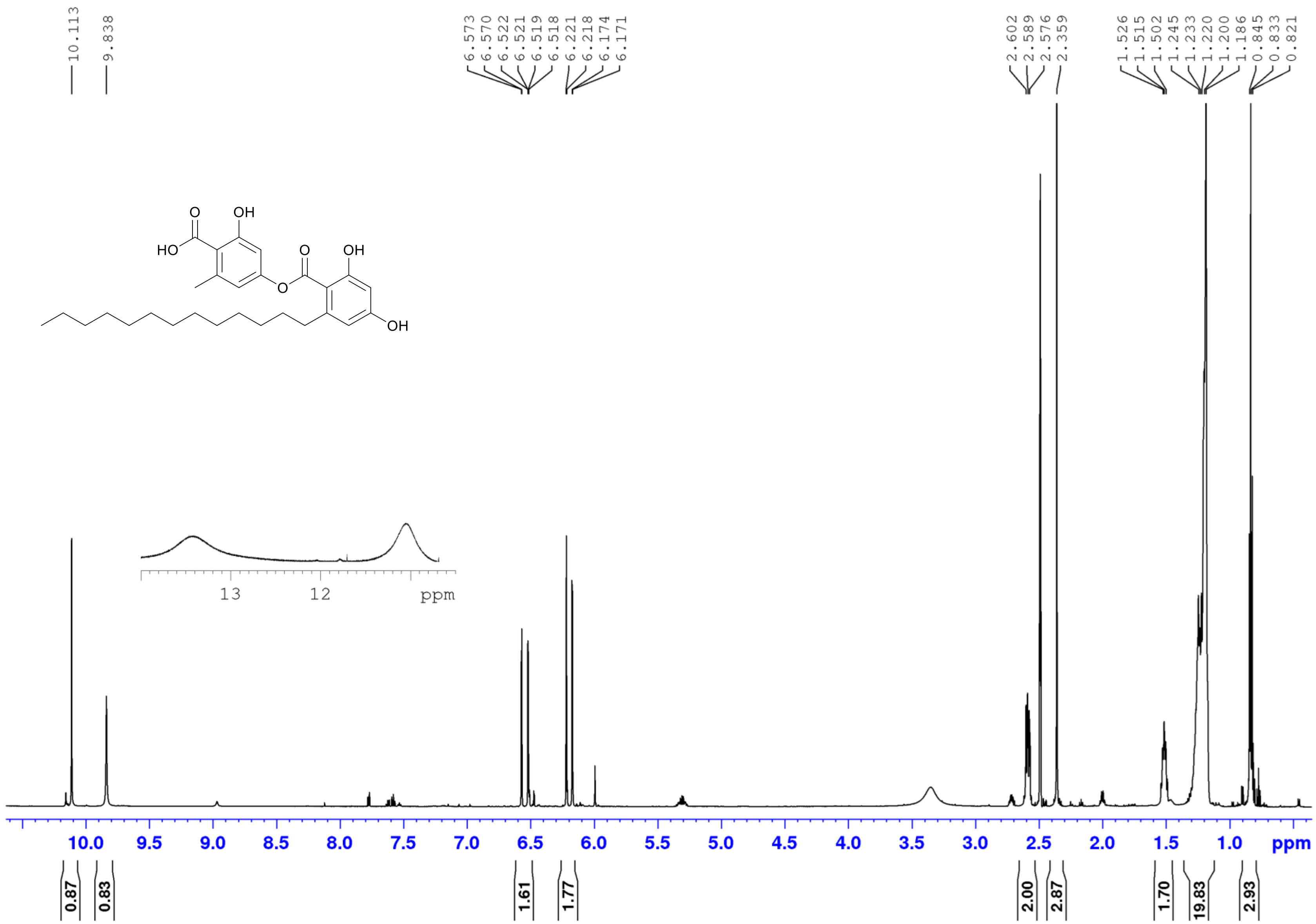

Figure S32. ^{1}H NMR spectrum (600 MHz) of ariestatin B (**3b**) in DMSO-d_6

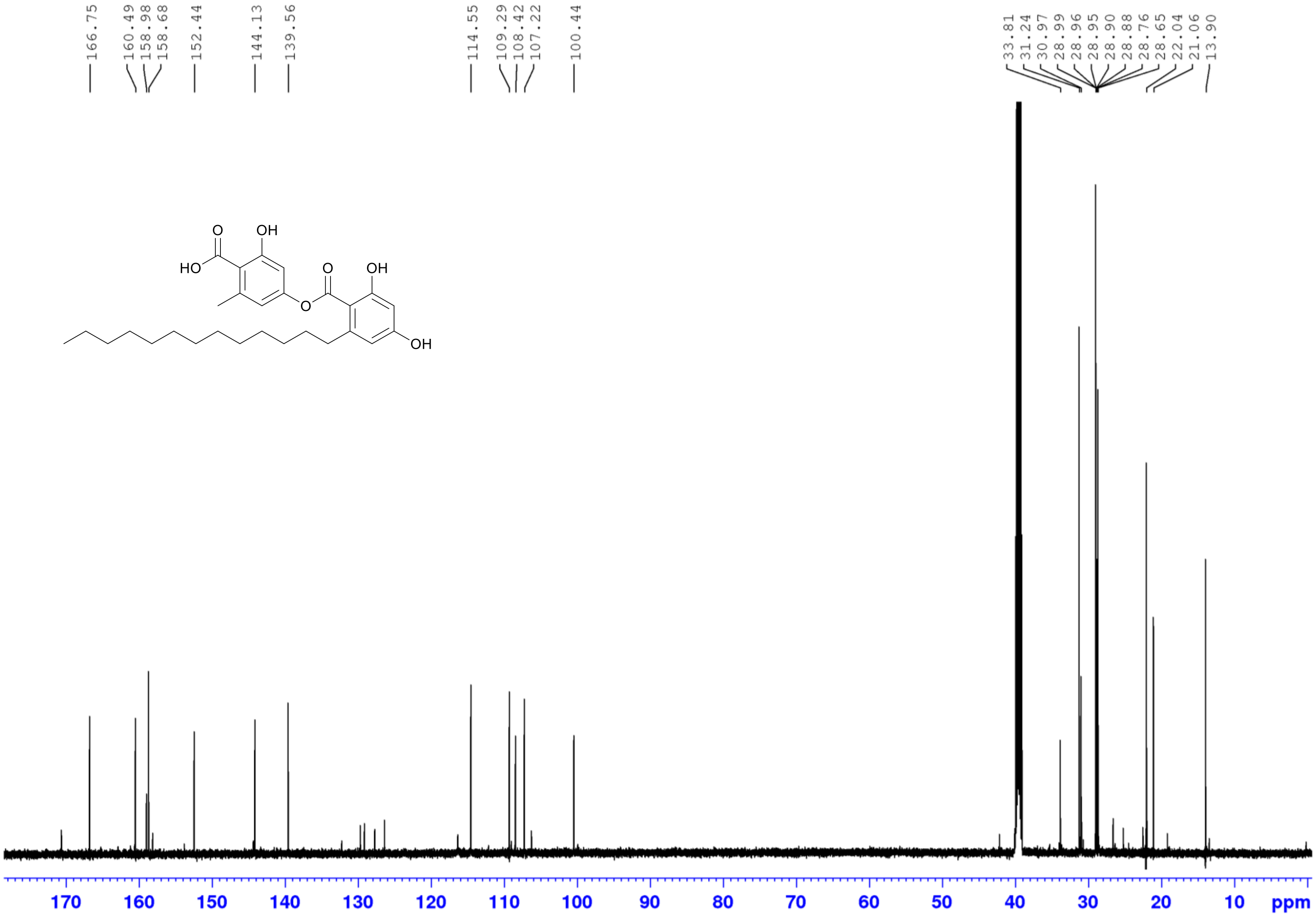

Figure S33. ^{13}C NMR spectrum (150 MHz) of ariestatin B (**3b**) in DMSO-d_6

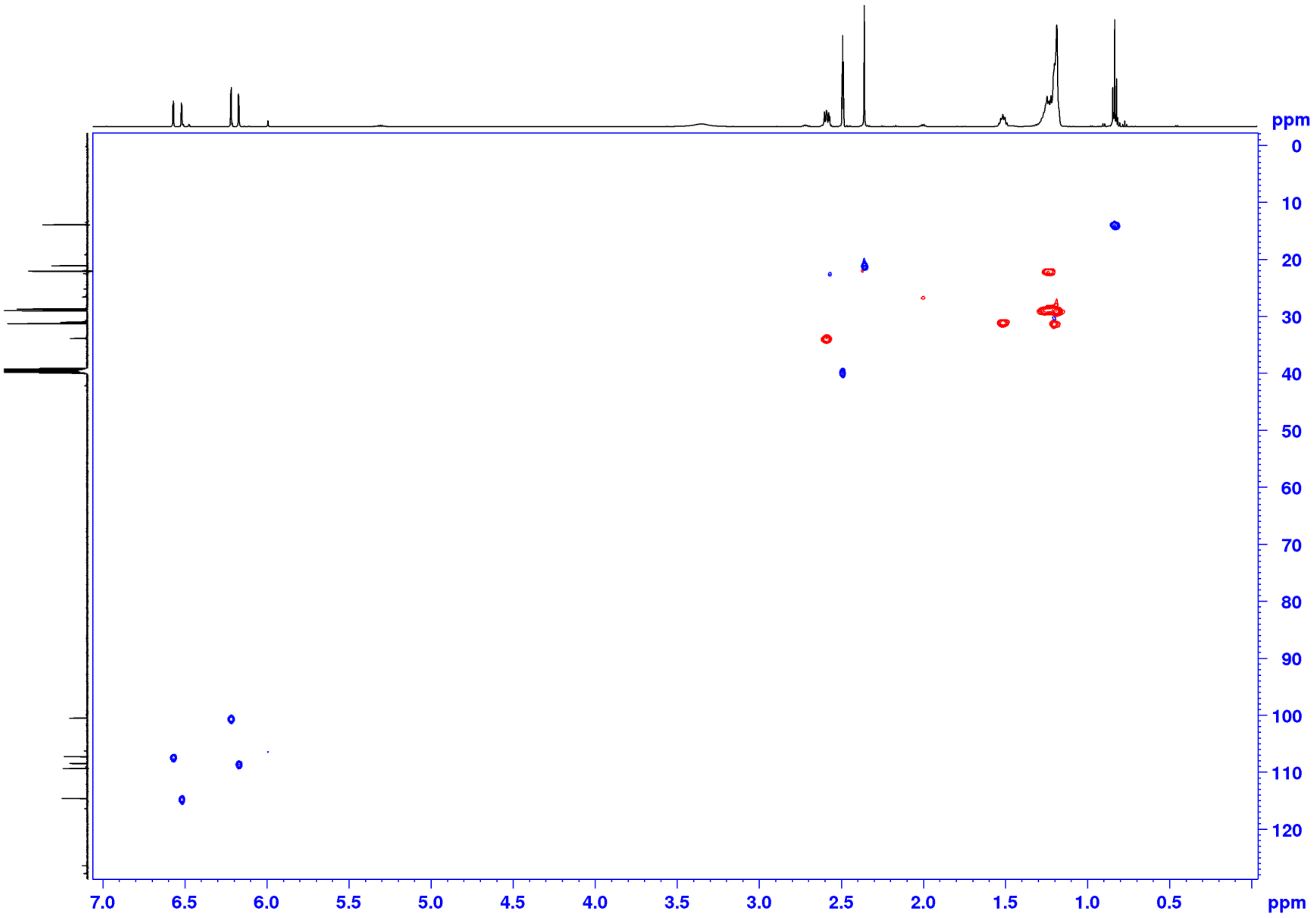

Figure S34. ^{1}H-^{13}C HSQC NMR spectrum (600 MHz) of ariestatin B (**3b**) in DMSO-d_6

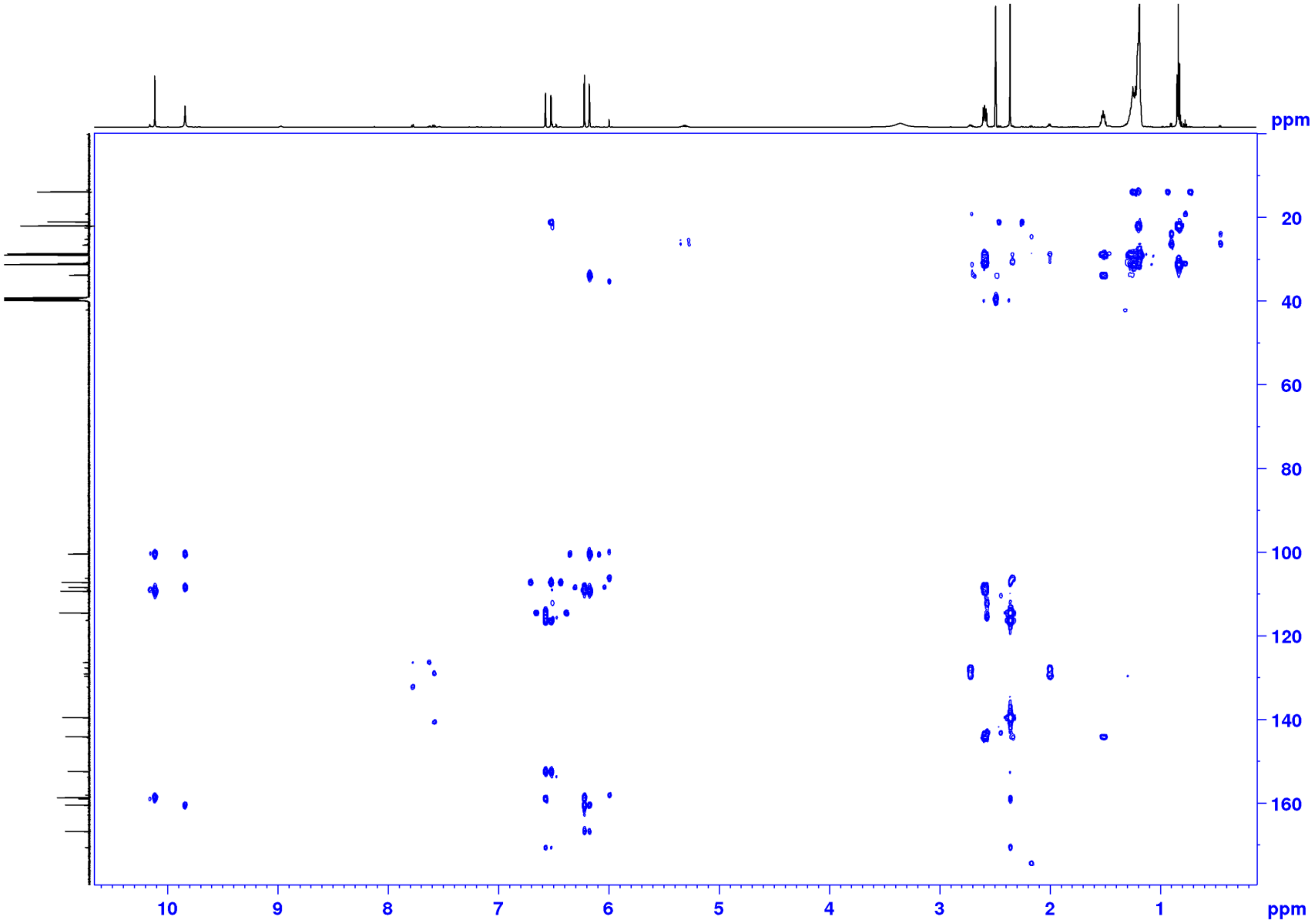

Figure S35. ^{1}H-^{13}C HMBC NMR spectrum (600 MHz) of ariestatin B (**3b**) in DMSO-d_6

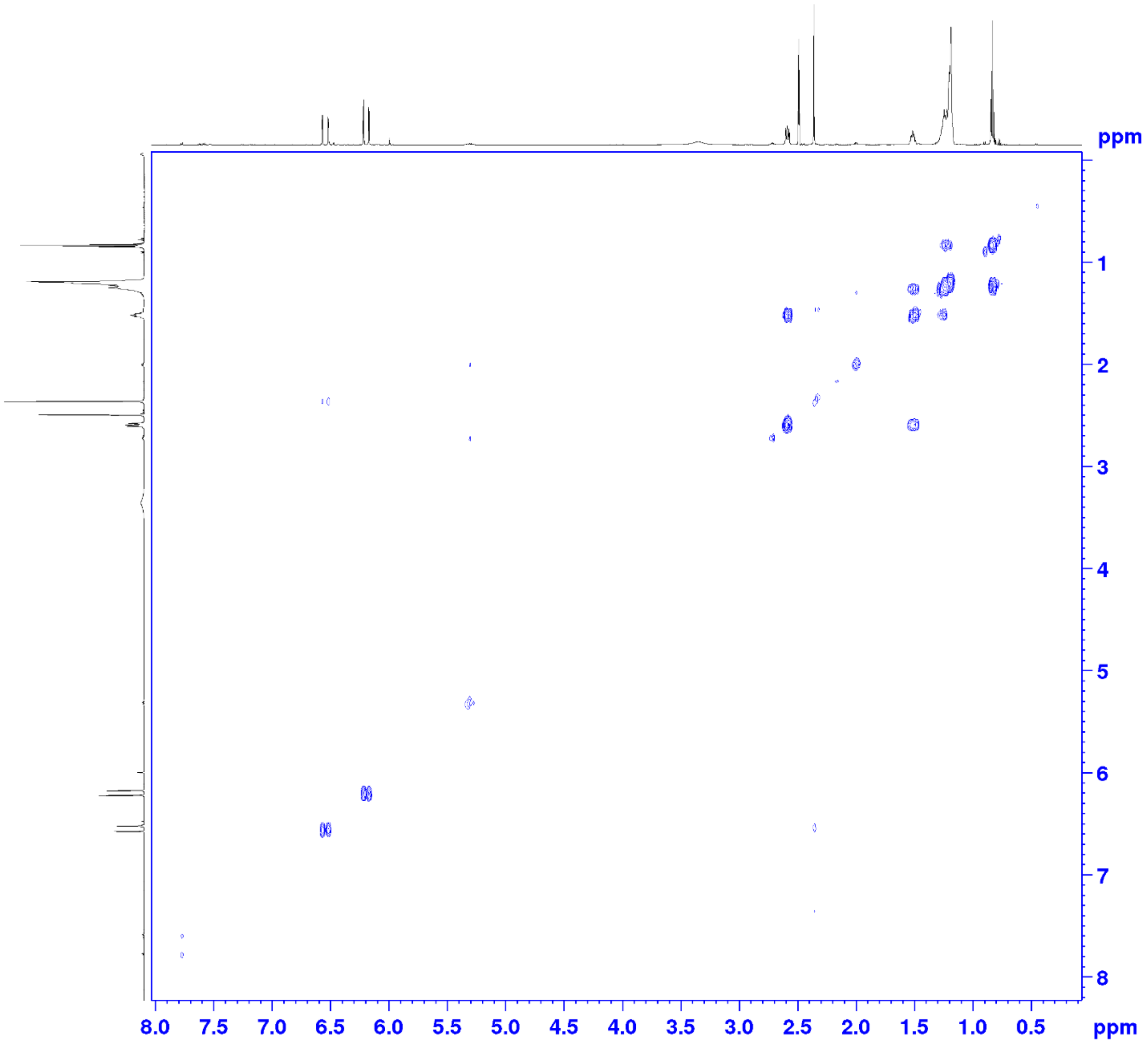

Figure S36. COSY NMR spectrum (600 MHz) of ariestatin B (**3b**) in DMSO-d_6

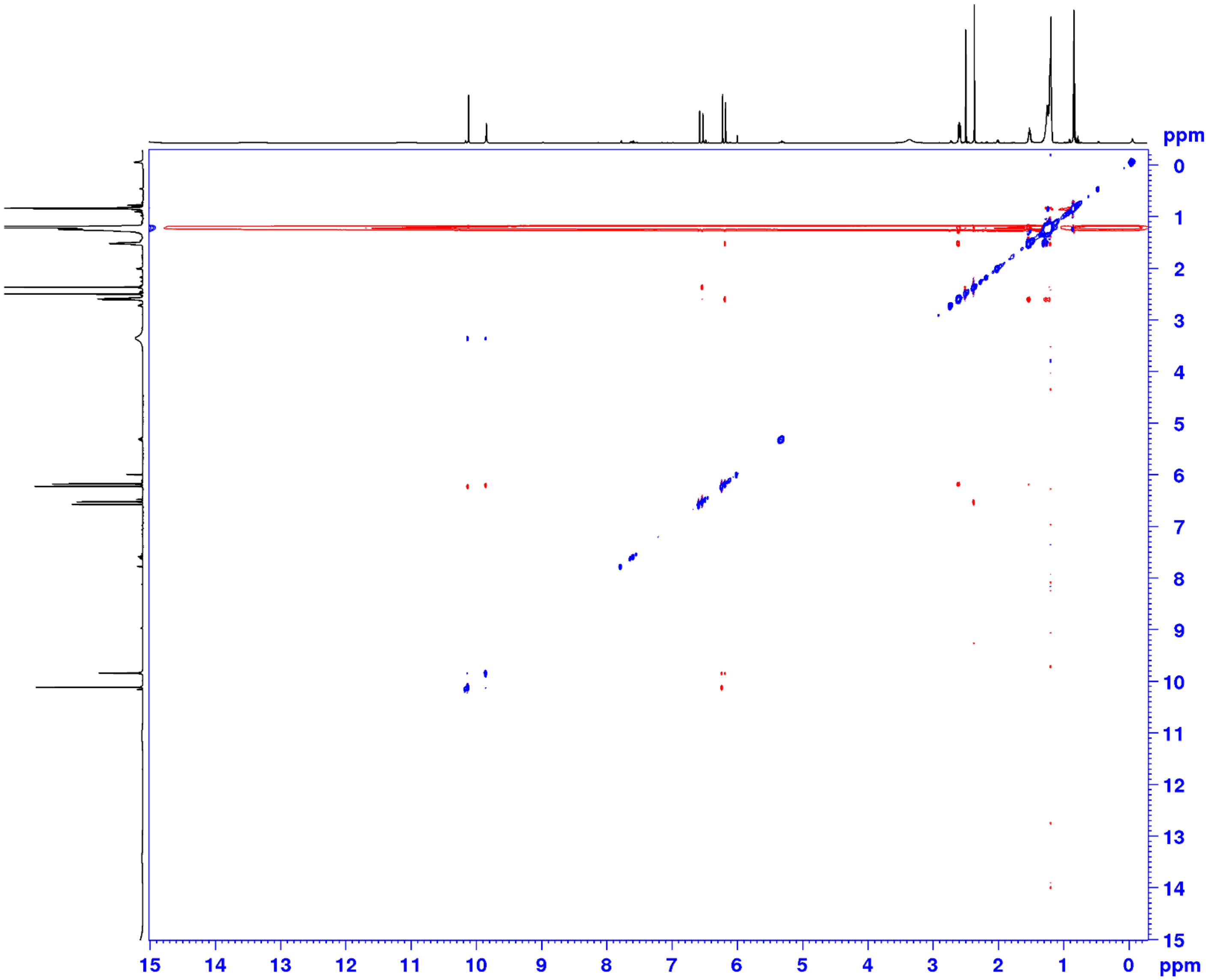

Figure S37. ROESY NMR spectrum (150 MHz) of ariestatin B (**3b**) in DMSO-d_6

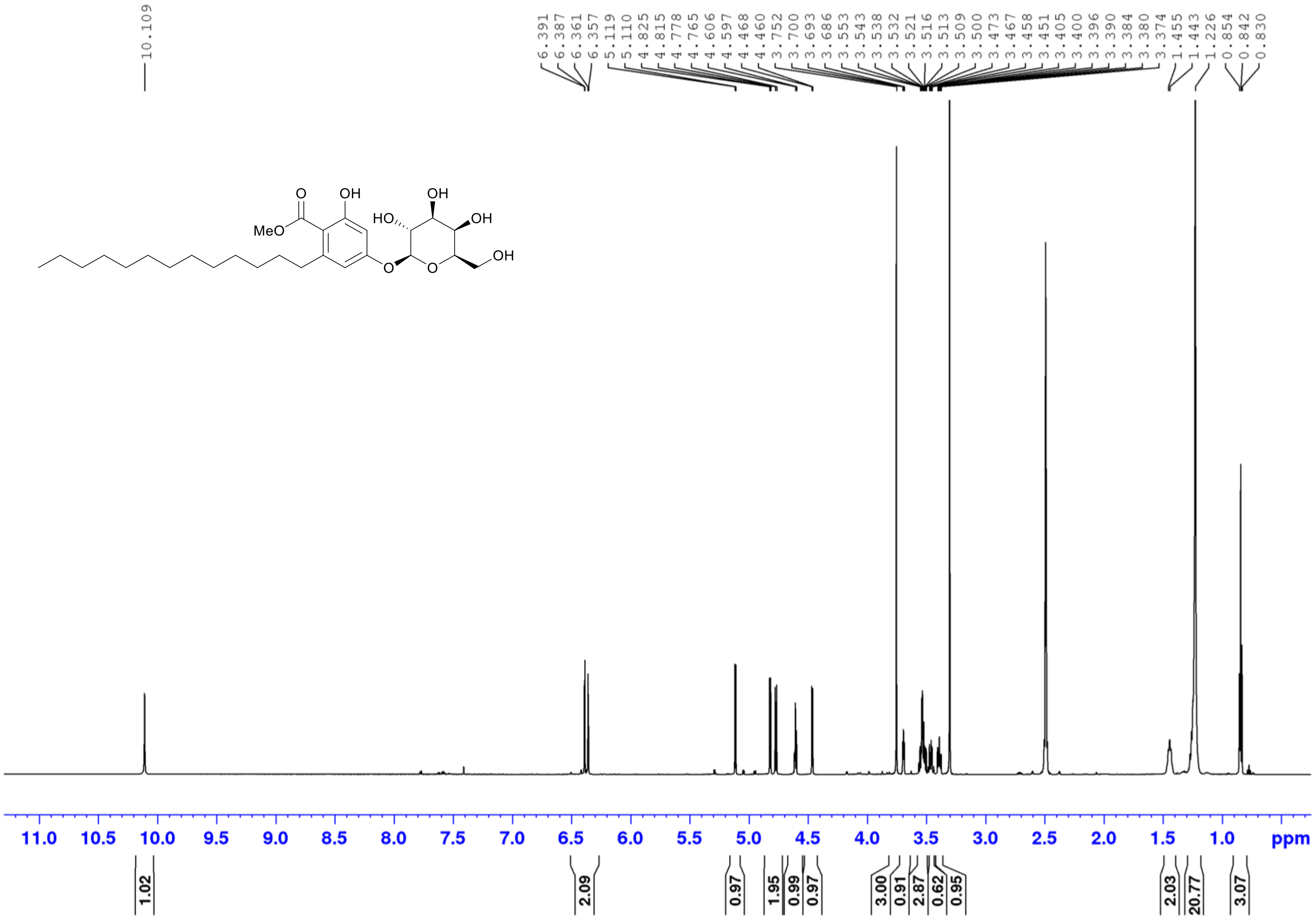

Figure S38. ^{1}H NMR spectrum (600 MHz) of ariestatin C (**3c**) in DMSO-d_6

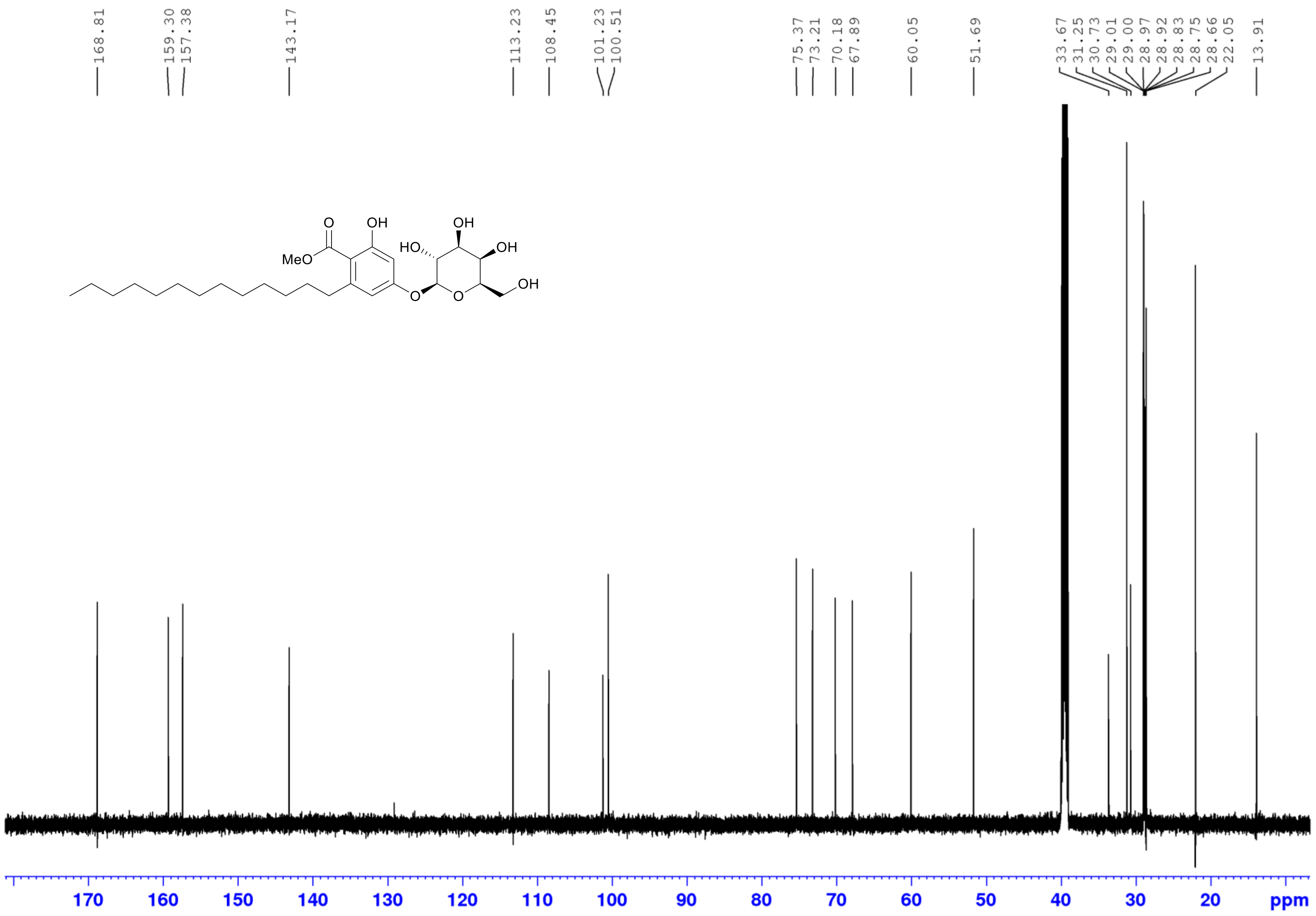

Figure S39. ^{13}C NMR spectrum (150 MHz) of ariestatin C (**3c**) in DMSO-d_6

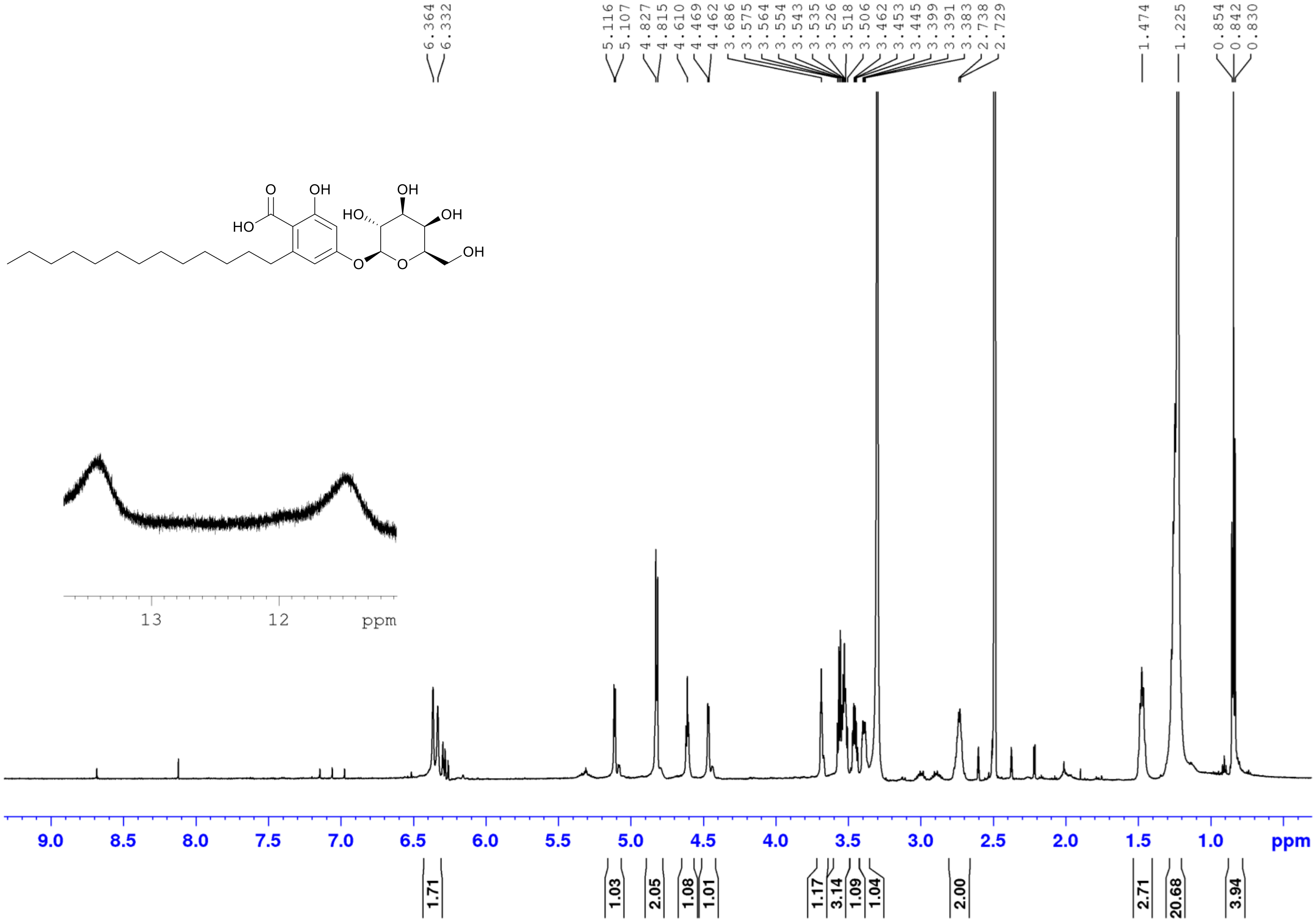

Figure S40. ^{1}H NMR spectrum (600 MHz) of ariestatin D (**3d**) in DMSO-d_6

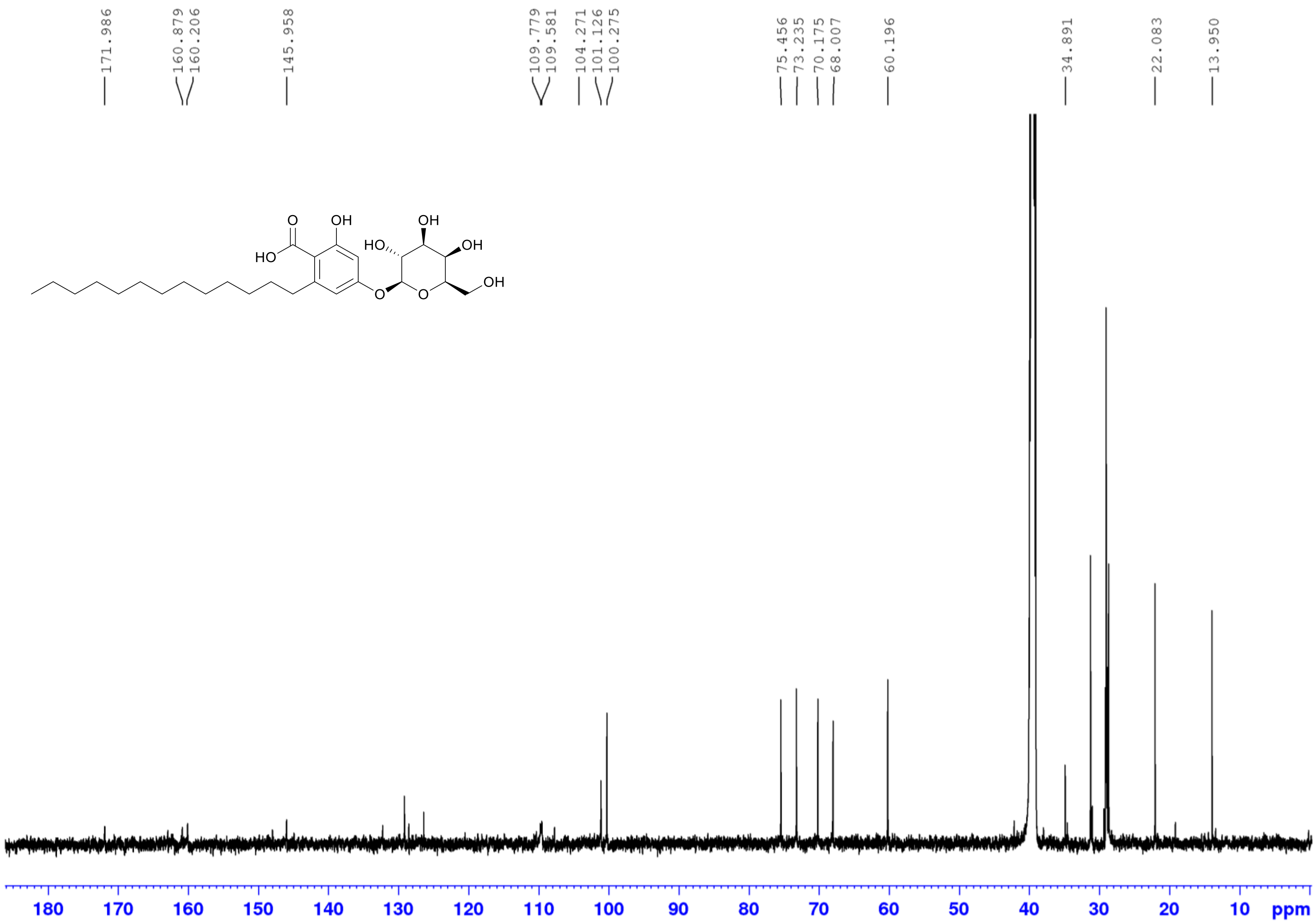

Figure S41. ^{13}C NMR spectrum (150 MHz) of ariestatin D (**3d**) in DMSO-d_6

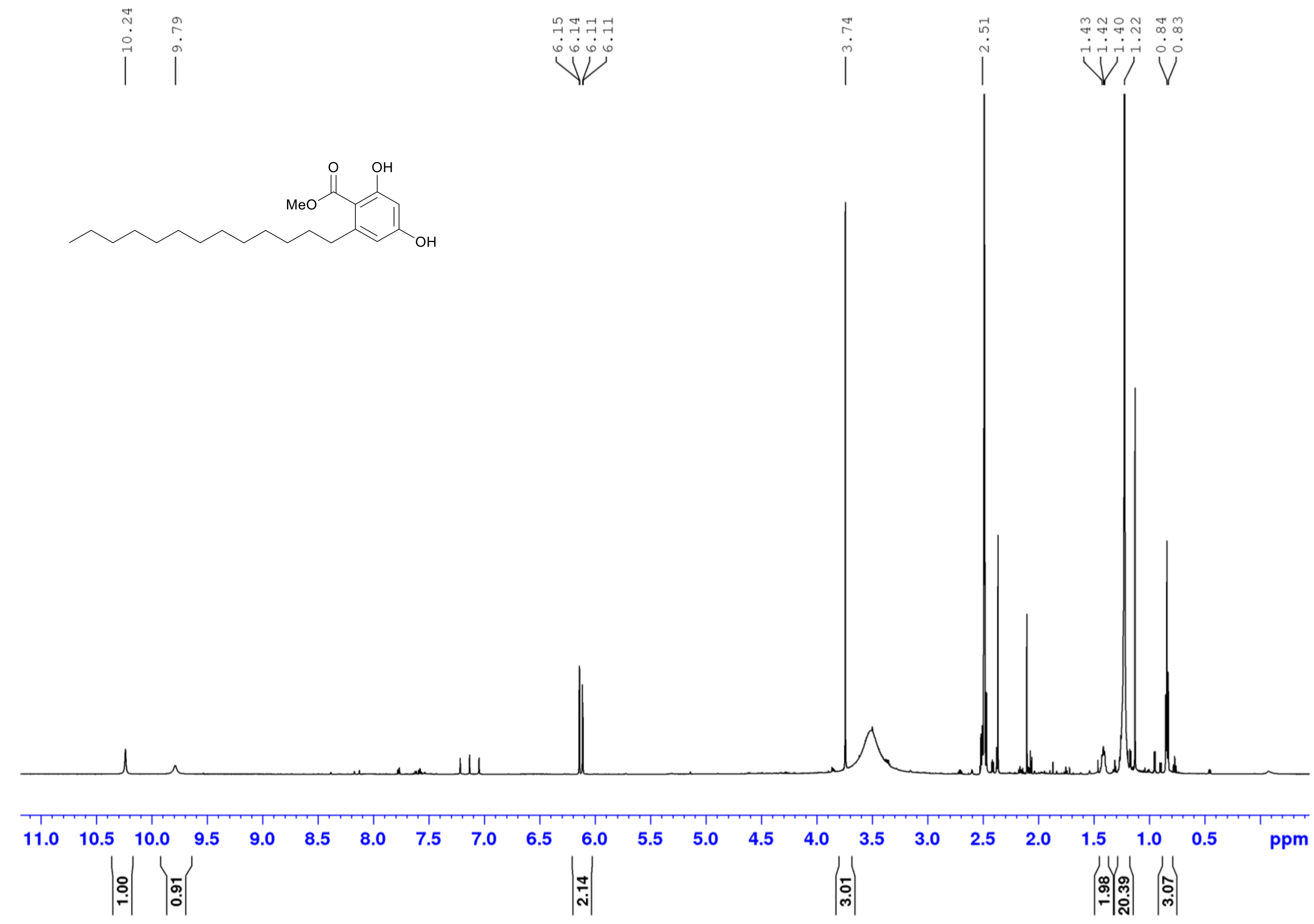

Figure S42. ^{1}H NMR spectrum (600 MHz) of ariestatin E (**3e**) in DMSO-d_6

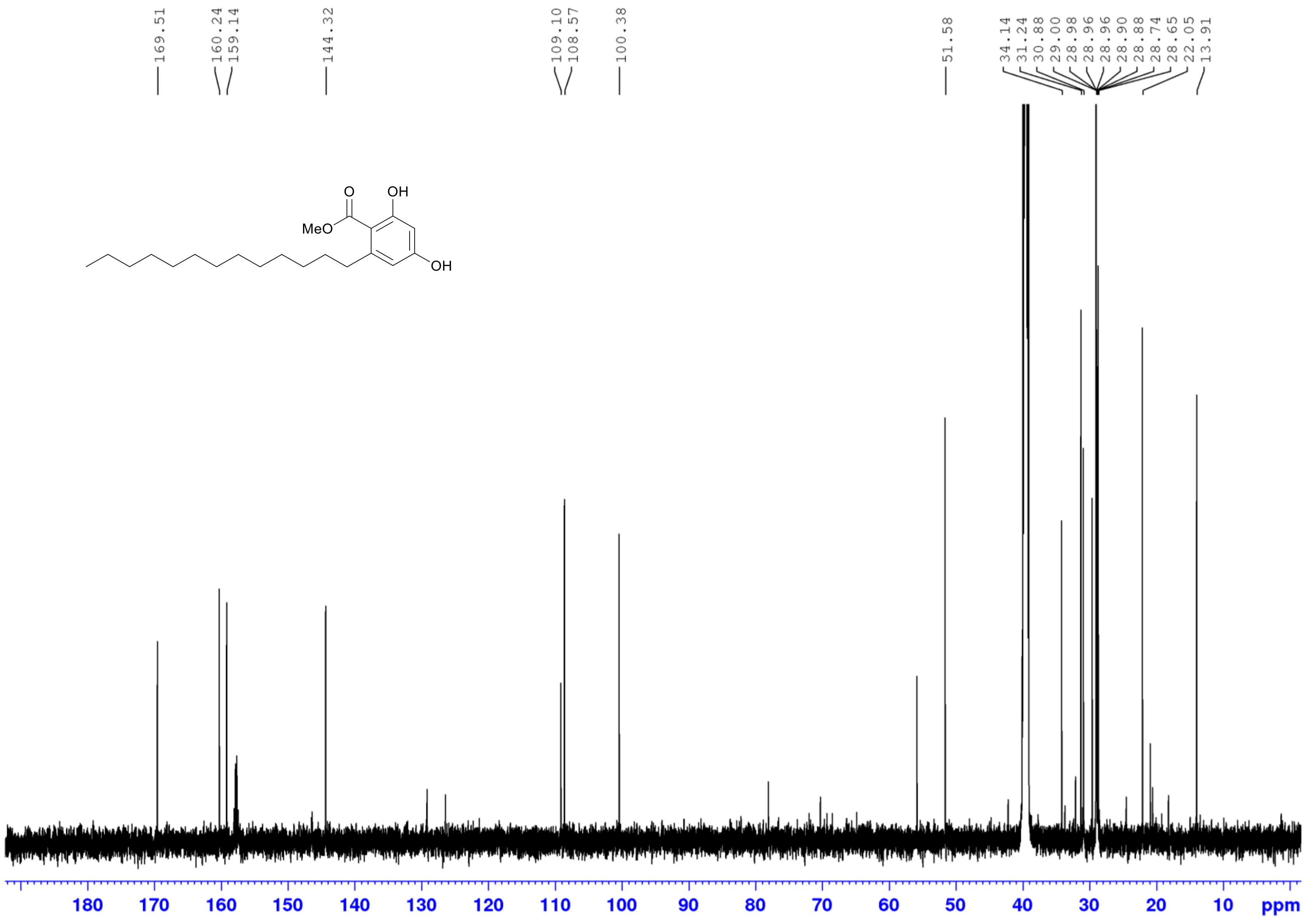

Figure S43. ^{13}C NMR spectrum (150 MHz) of ariestatin E (**3e**) in DMSO-d_6

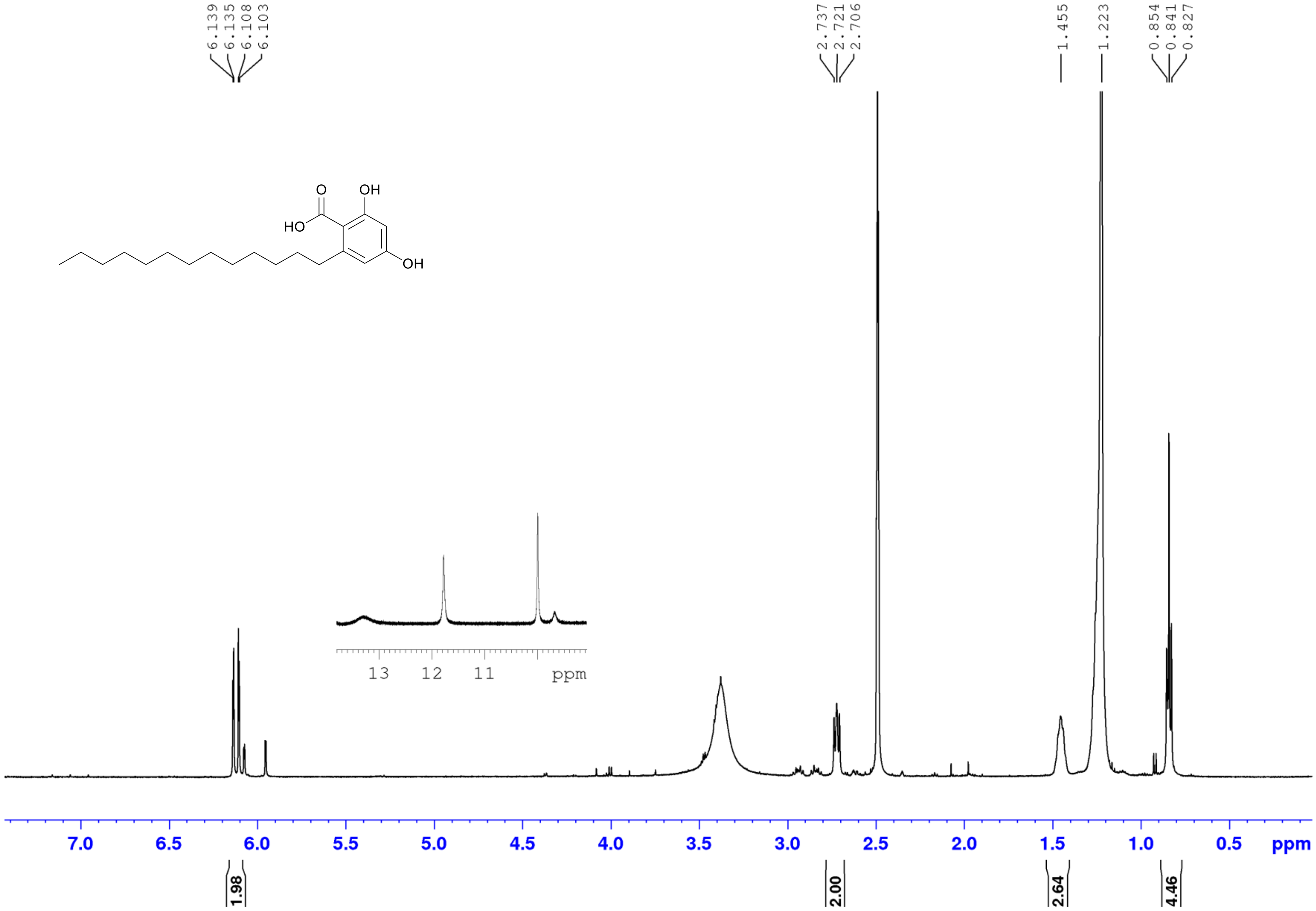

Figure S44. ^{1}H NMR spectrum (600 MHz) of ariestatin F (**3f**) in DMSO-d_6

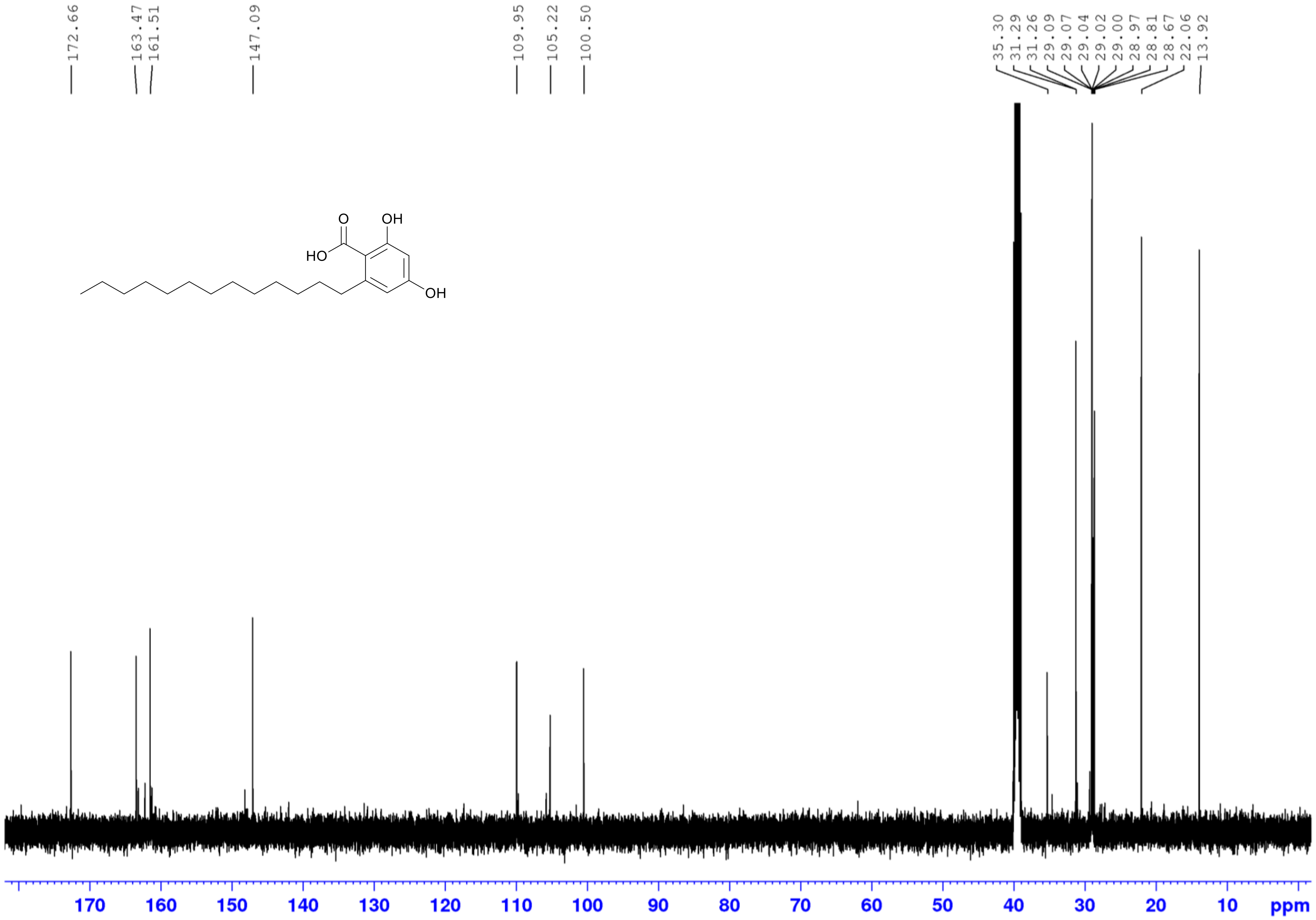

Figure S45. ^{13}C NMR spectrum (150 MHz) of ariestatin F (**3f**) in DMSO-d_6

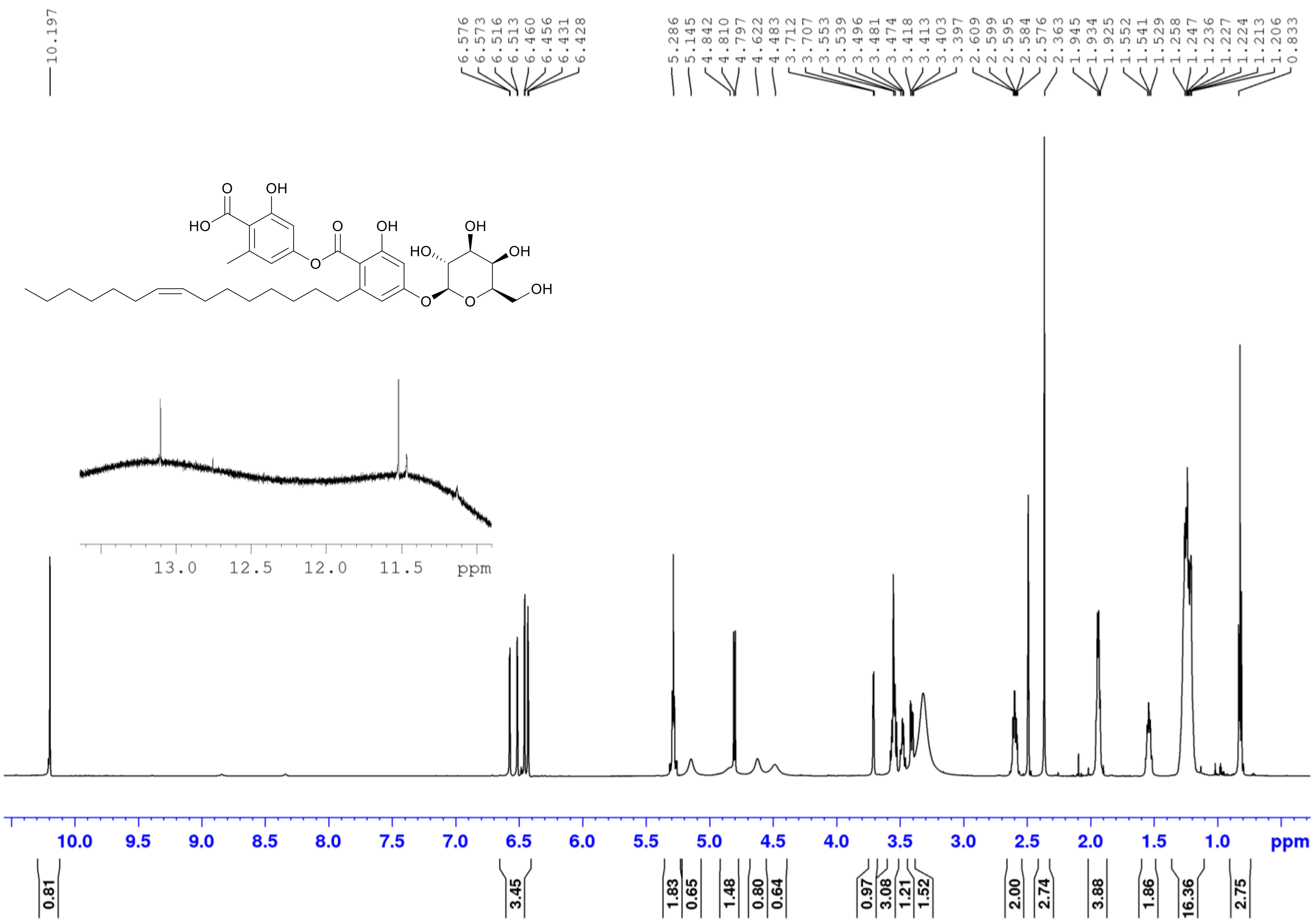

Figure S46. ^{1}H NMR spectrum (600 MHz) of capricostatin A (**4a**) in DMSO-d_6

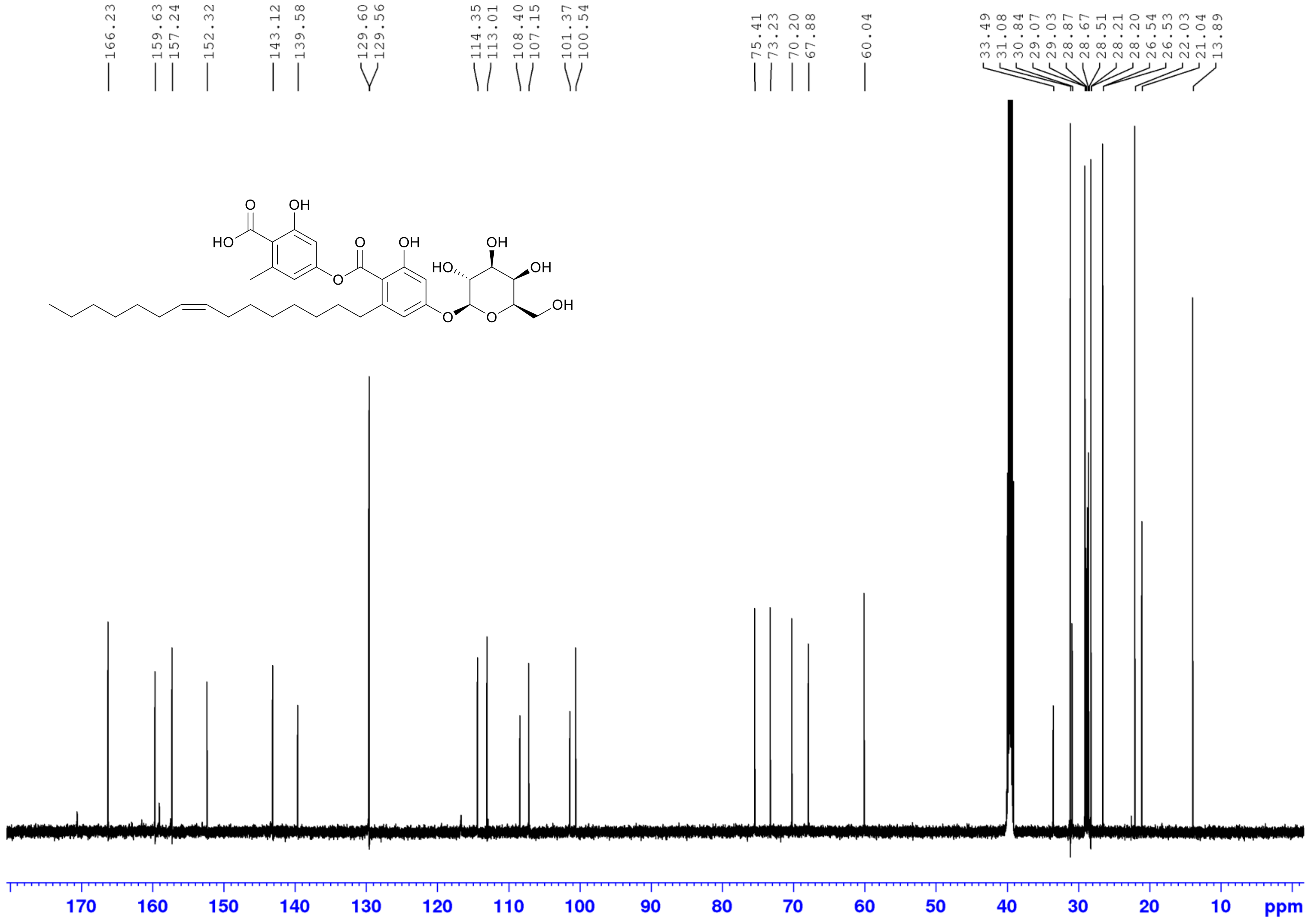

Figure S47. ^{13}C NMR spectrum (150 MHz) of capricostatin A (**4a**) in DMSO-d_6

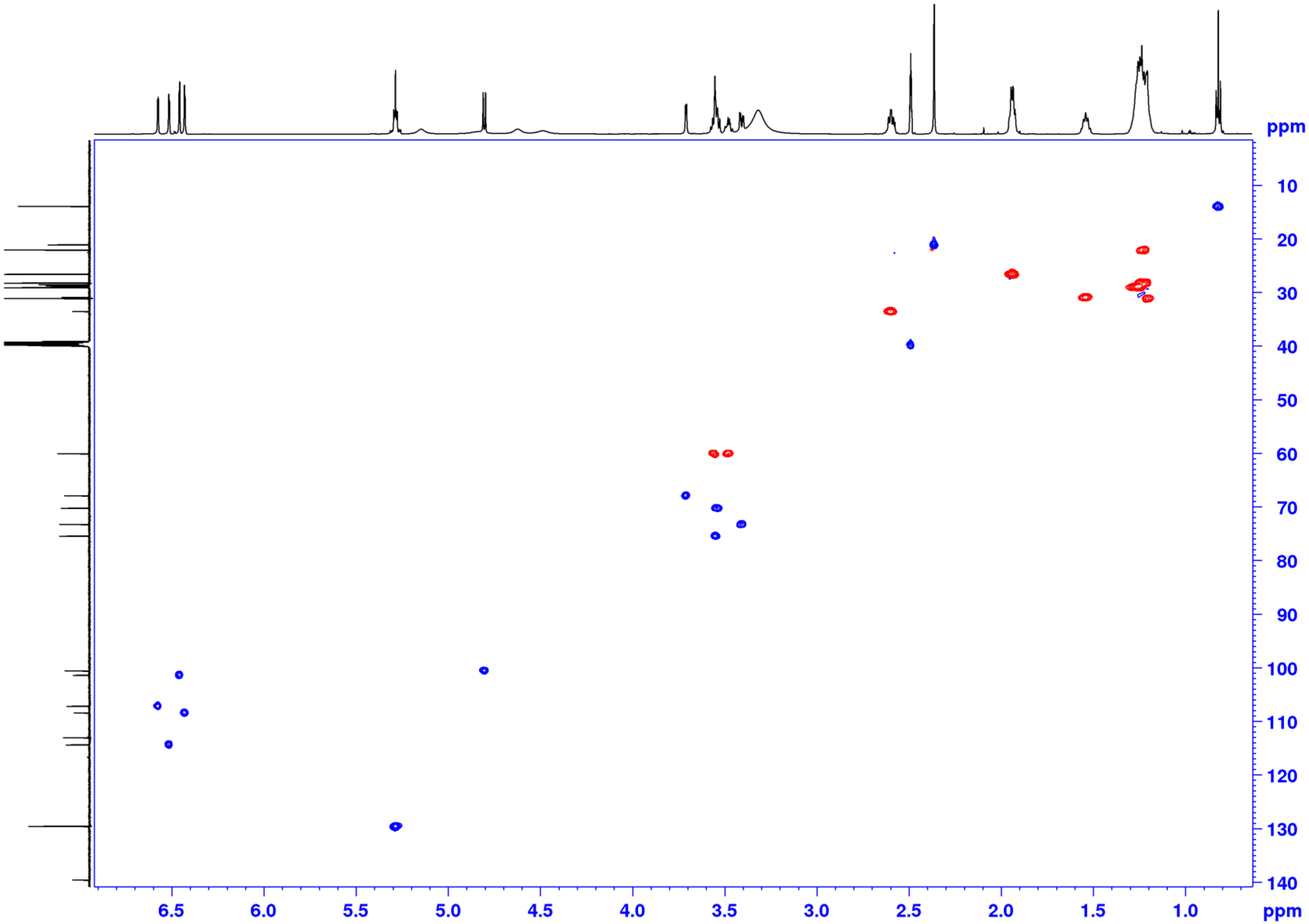

Figure S48. ^{1}H-^{13}C HSQC NMR spectrum (600 MHz) of capricostatin A (**4a**) in DMSO-d_6

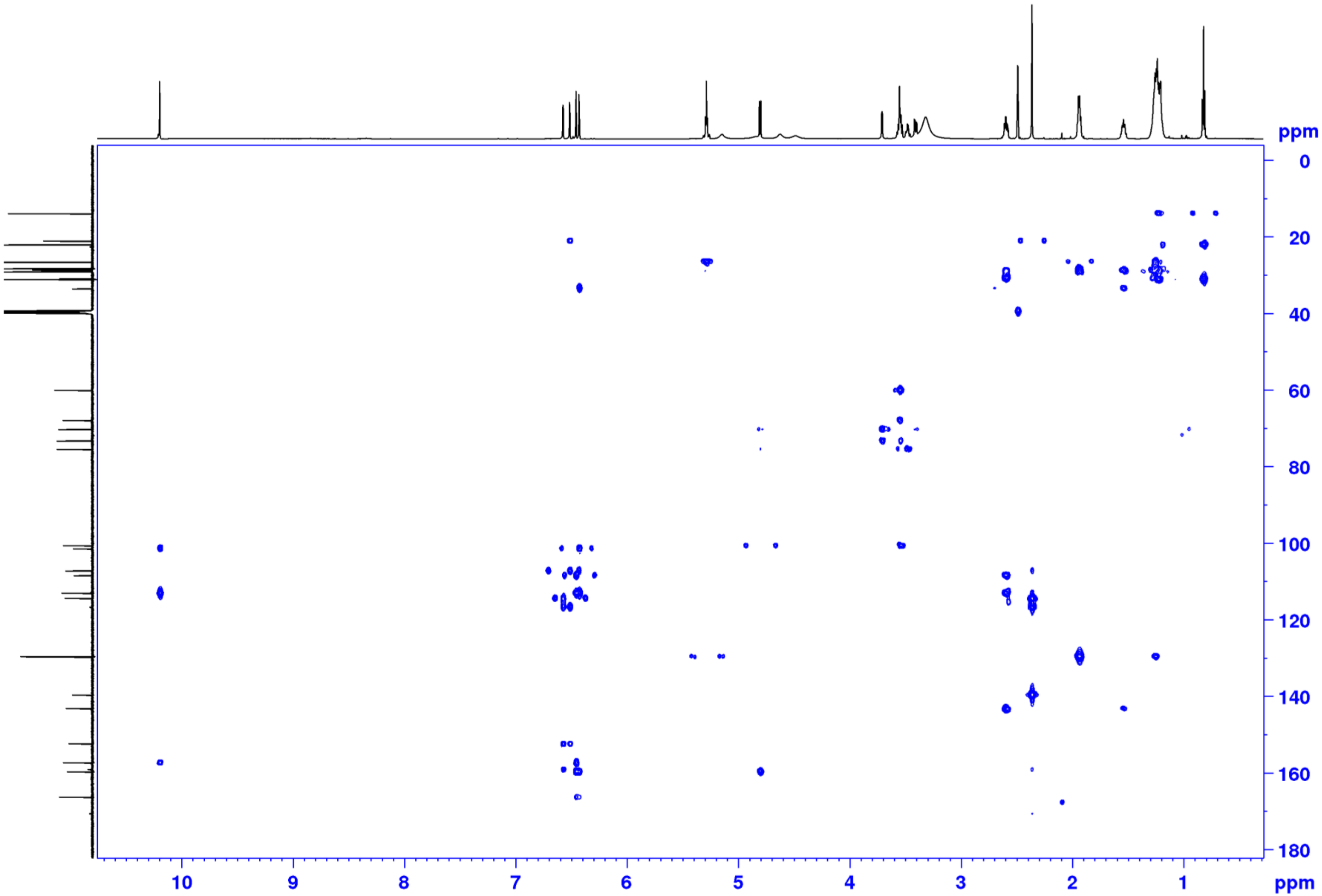

Figure S49. ^{1}H-^{13}C HMBC NMR spectrum (600 MHz) of capricostatin A (**4a**) in DMSO-d_6

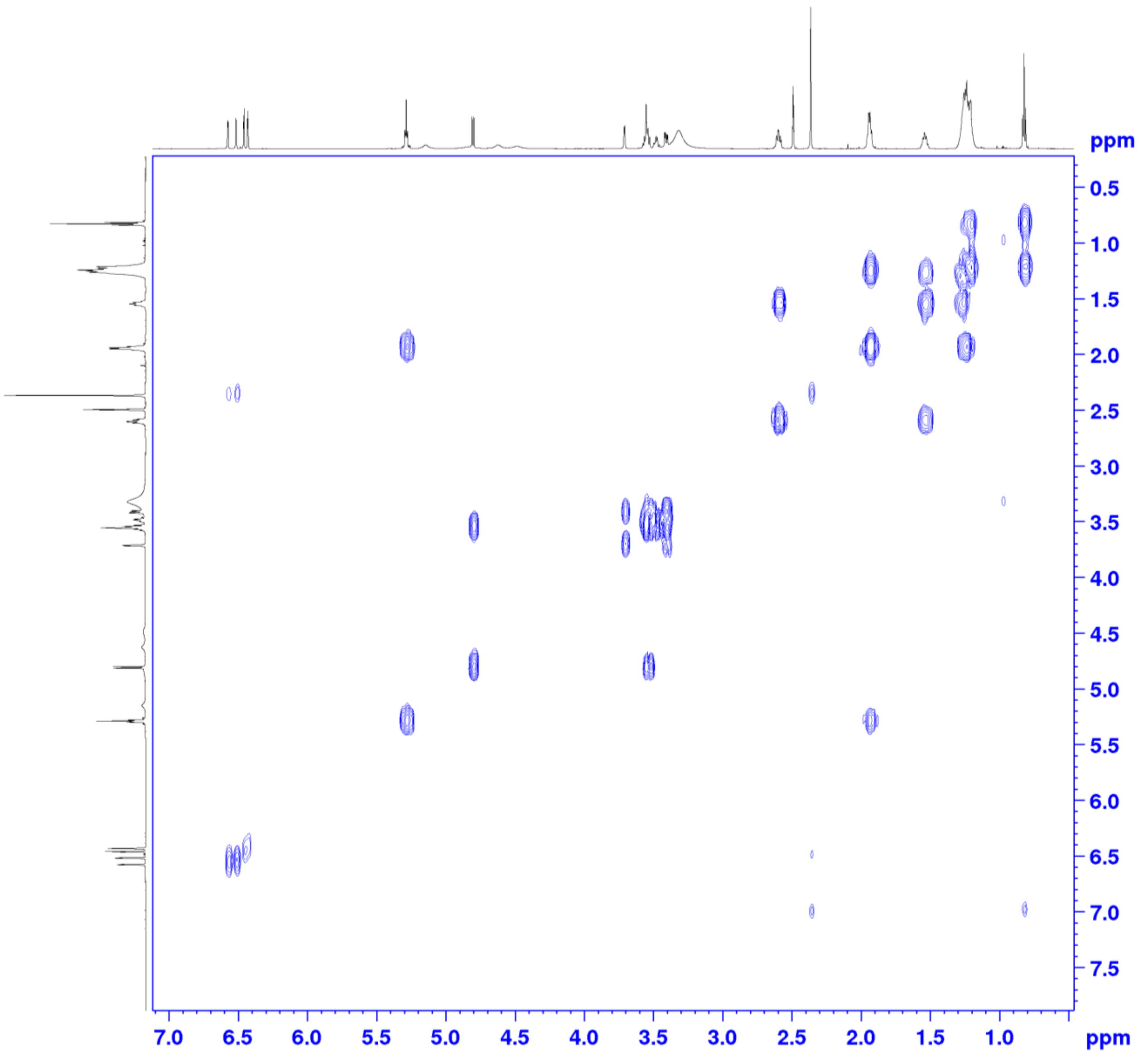

Figure S50. COSY NMR spectrum (600 MHz) of capricostatin A (**4a**) in DMSO-d_6

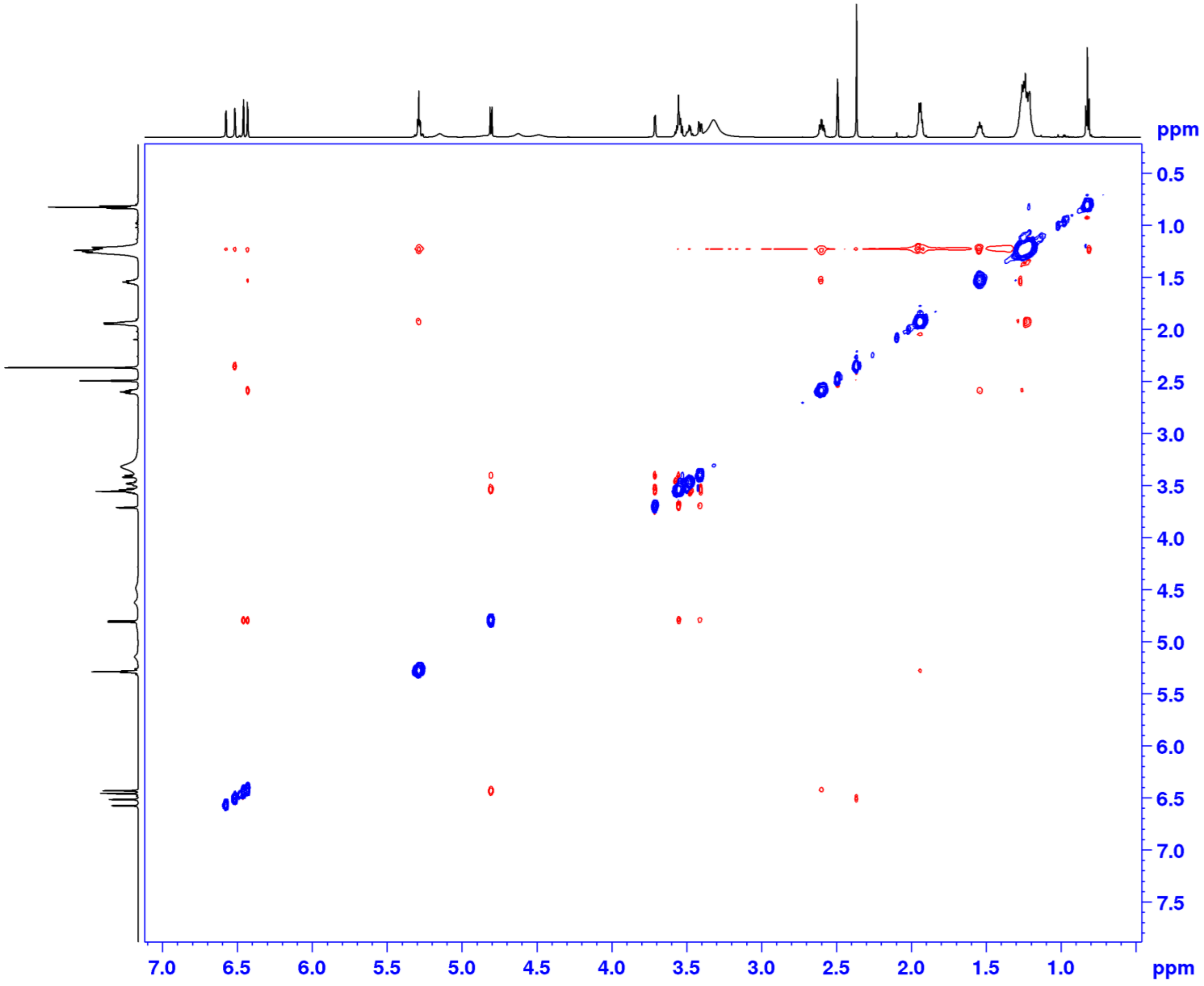

Figure S51. ROESY NMR spectrum (150 MHz) of capricostatin A (**4a**) in DMSO-d_6

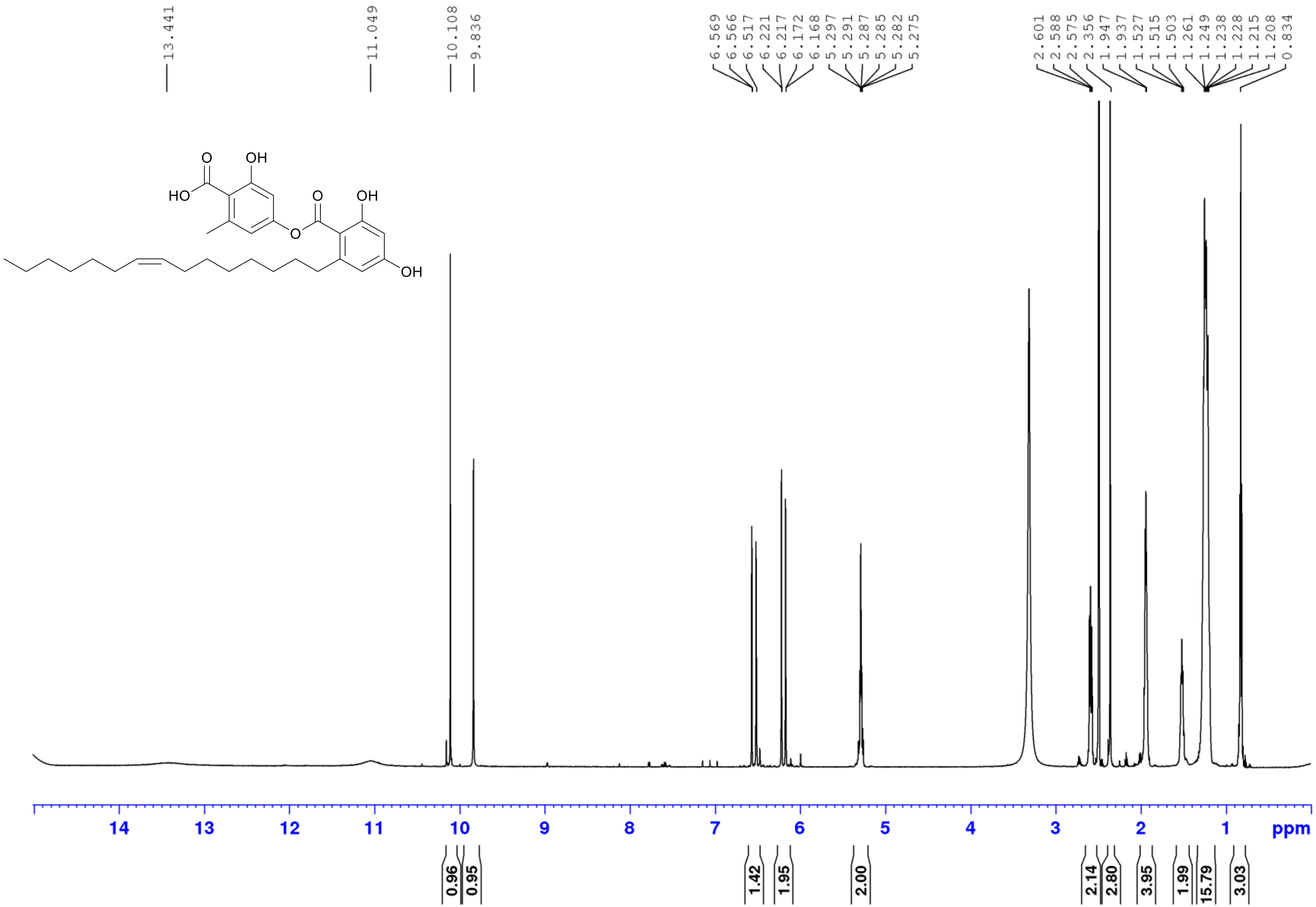

Figure S52. ^{1}H NMR spectrum (600 MHz) of capricostatin B (**4b**) in DMSO-d_6

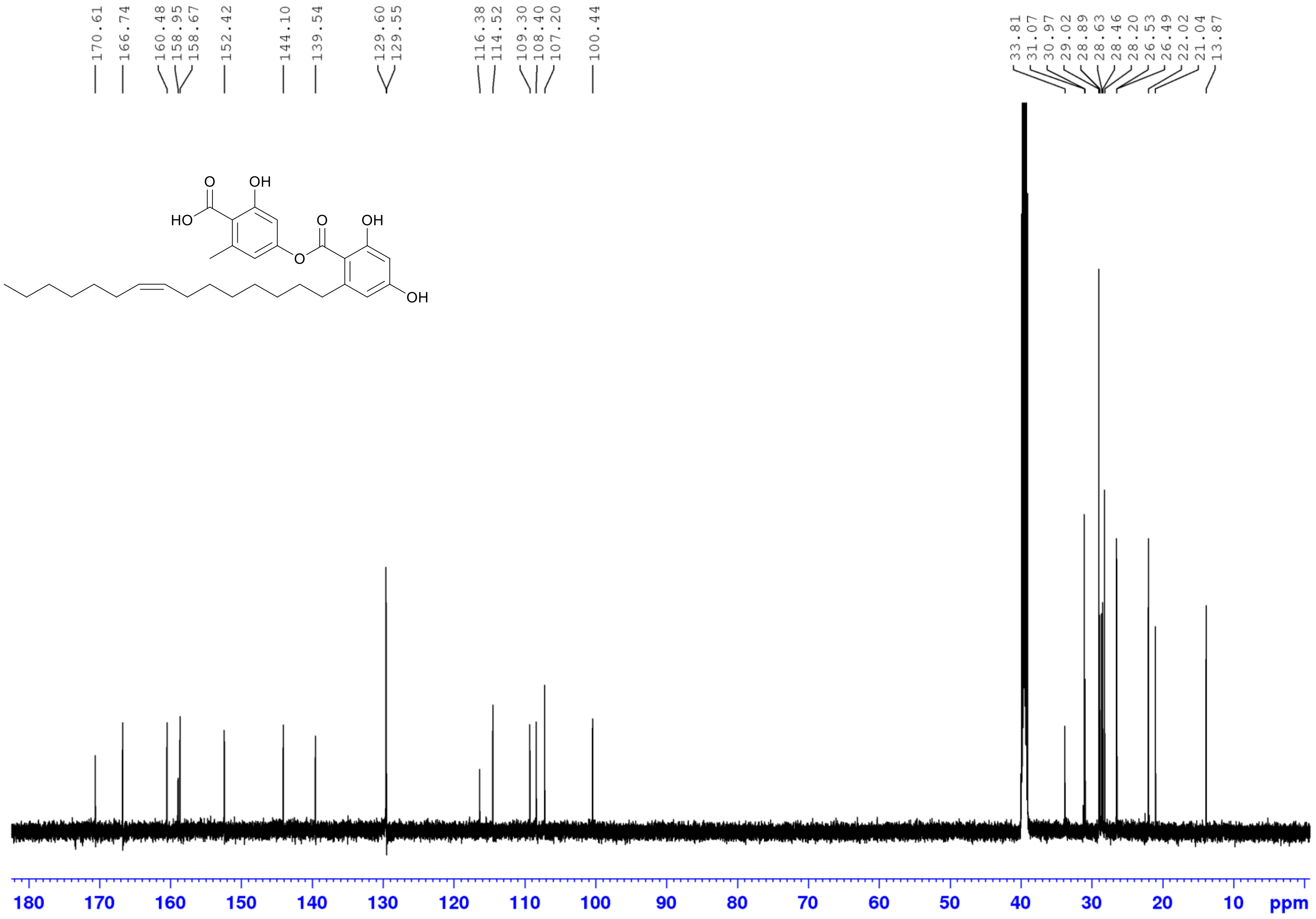

Figure S53. ^{13}C NMR spectrum (150 MHz) of capricostatin B (**4b**) in DMSO-d_6

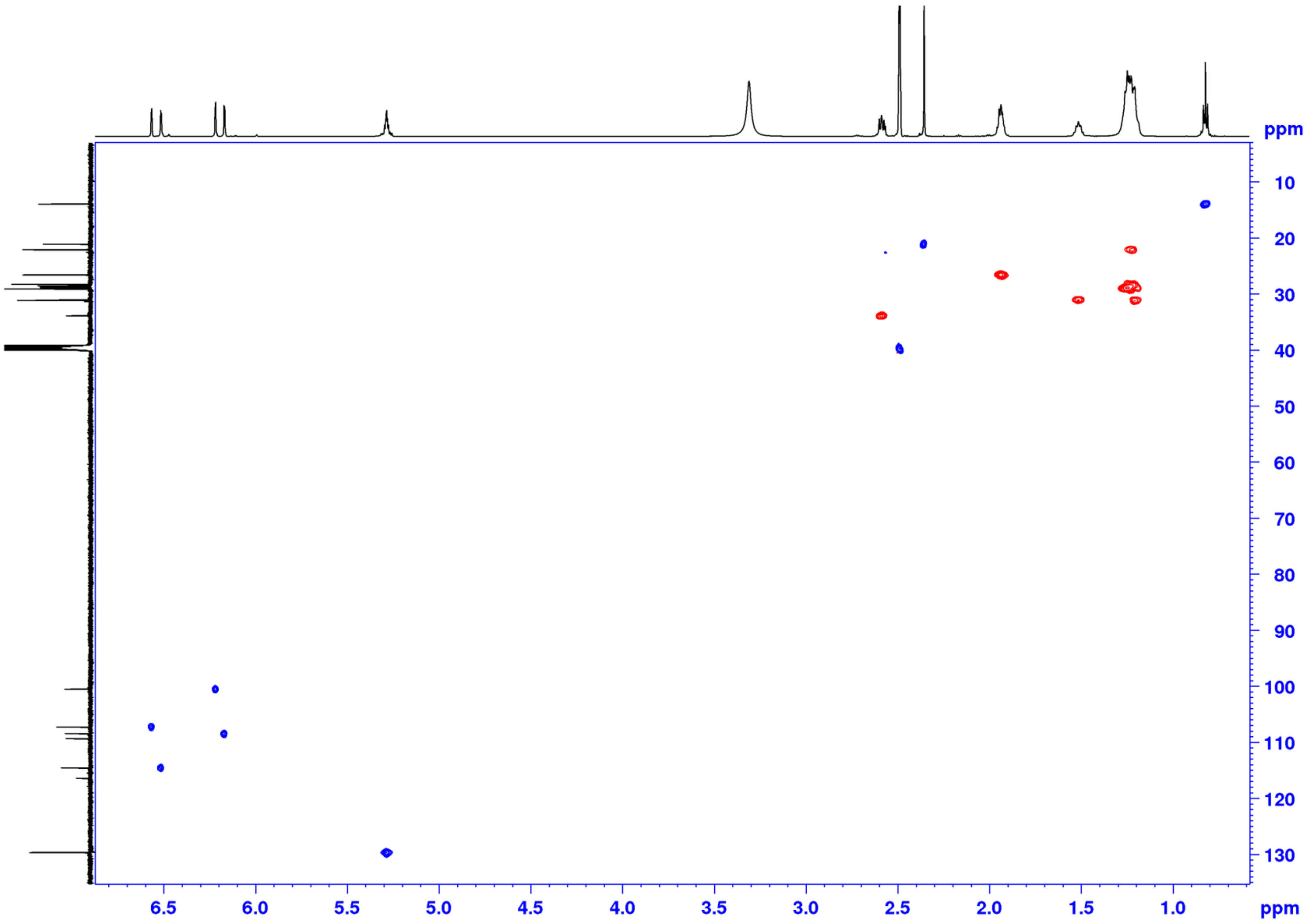

Figure S54. ^{1}H-^{13}C HSQC NMR spectrum (600 MHz) of capricostatin B (**4b**) in DMSO-d_6

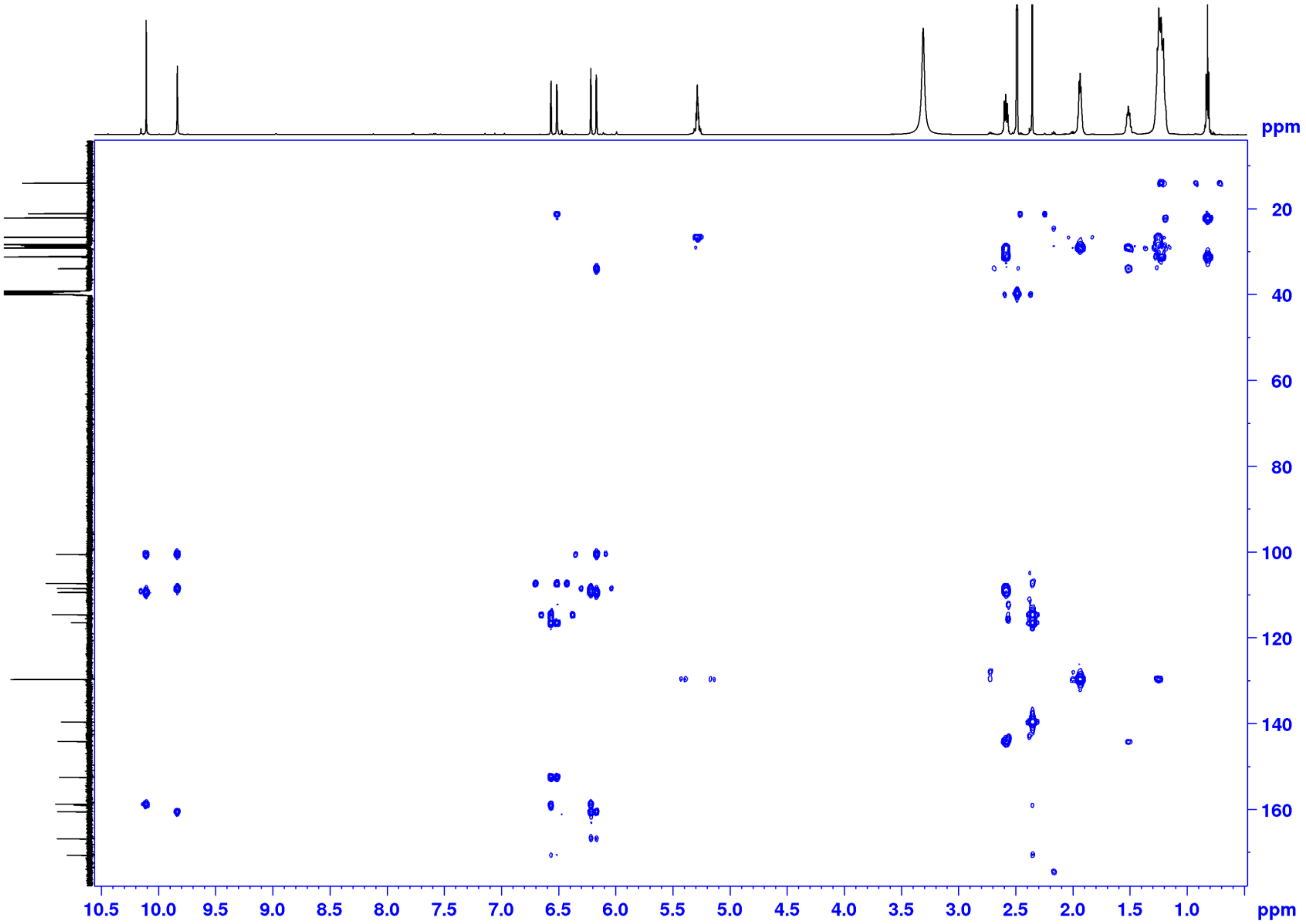

Figure S55. ^{1}H-^{13}C HSQC NMR spectrum (600 MHz) of capricostatin B (**4b**) in DMSO-d_6

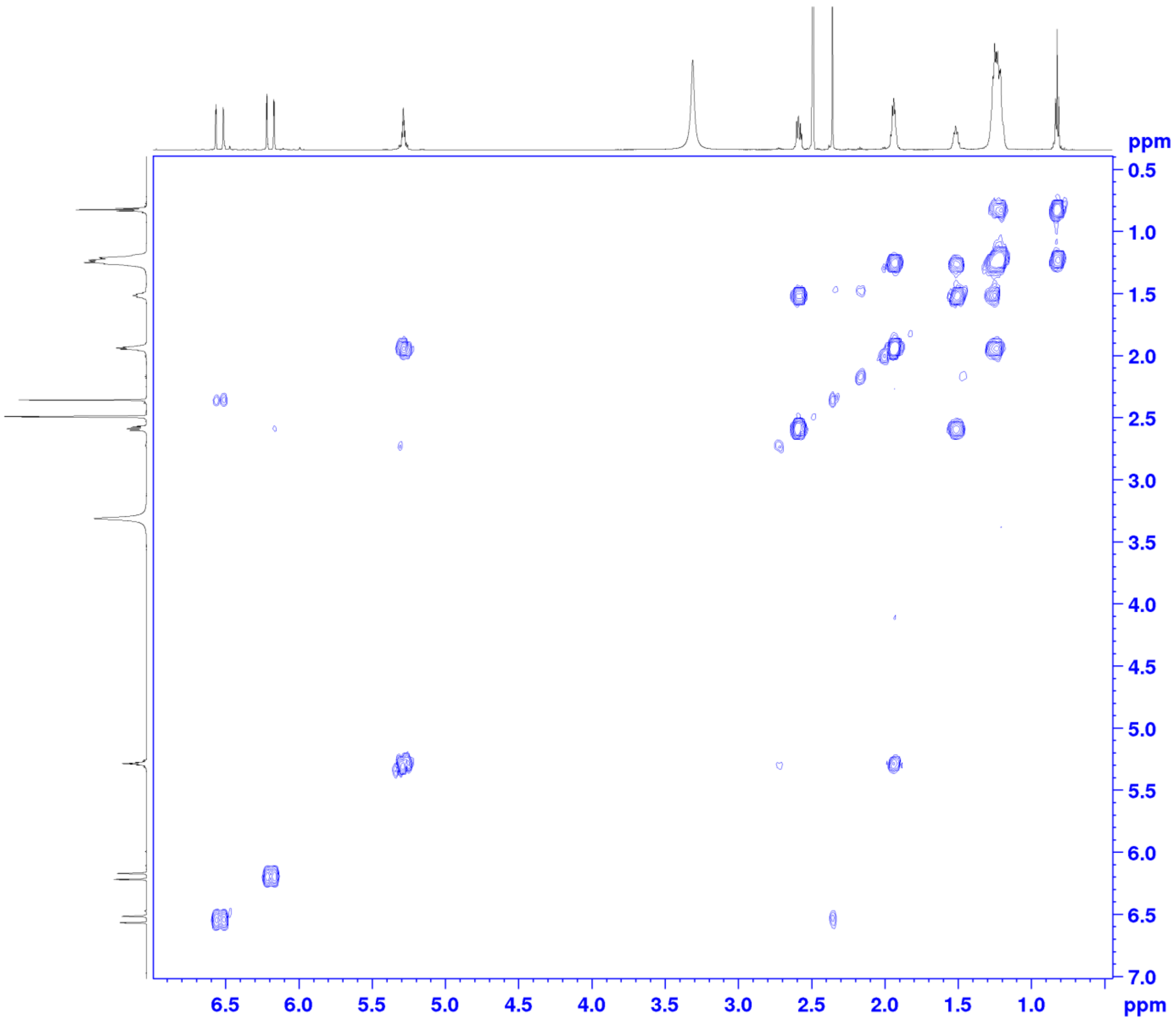

Figure S56. COSY NMR spectrum (600 MHz) of capricostatin B (**4b**) in DMSO-d_6

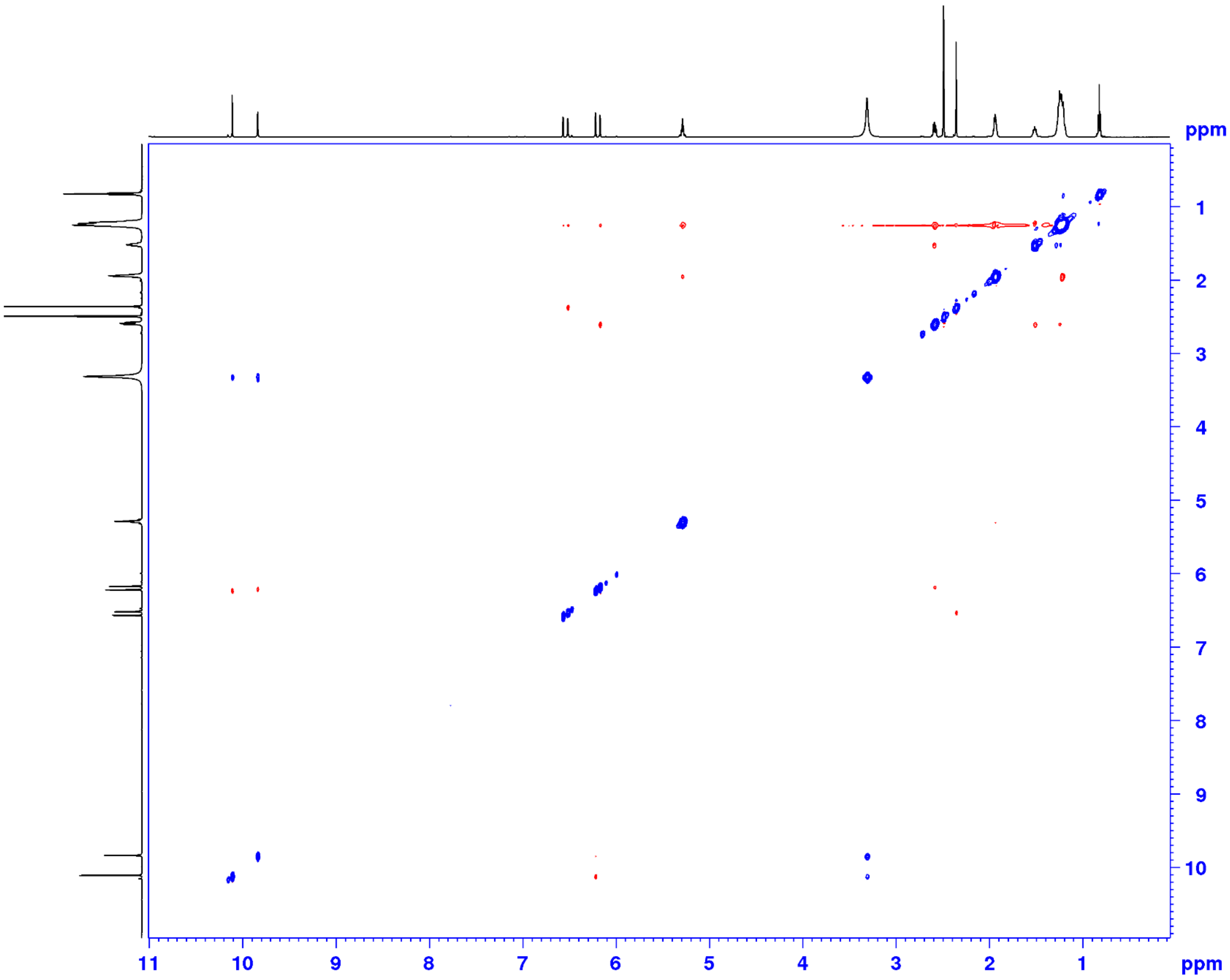

Figure S57. ROESY NMR spectrum (600 MHz) of capricostatin B (**4b**) in DMSO-d_6

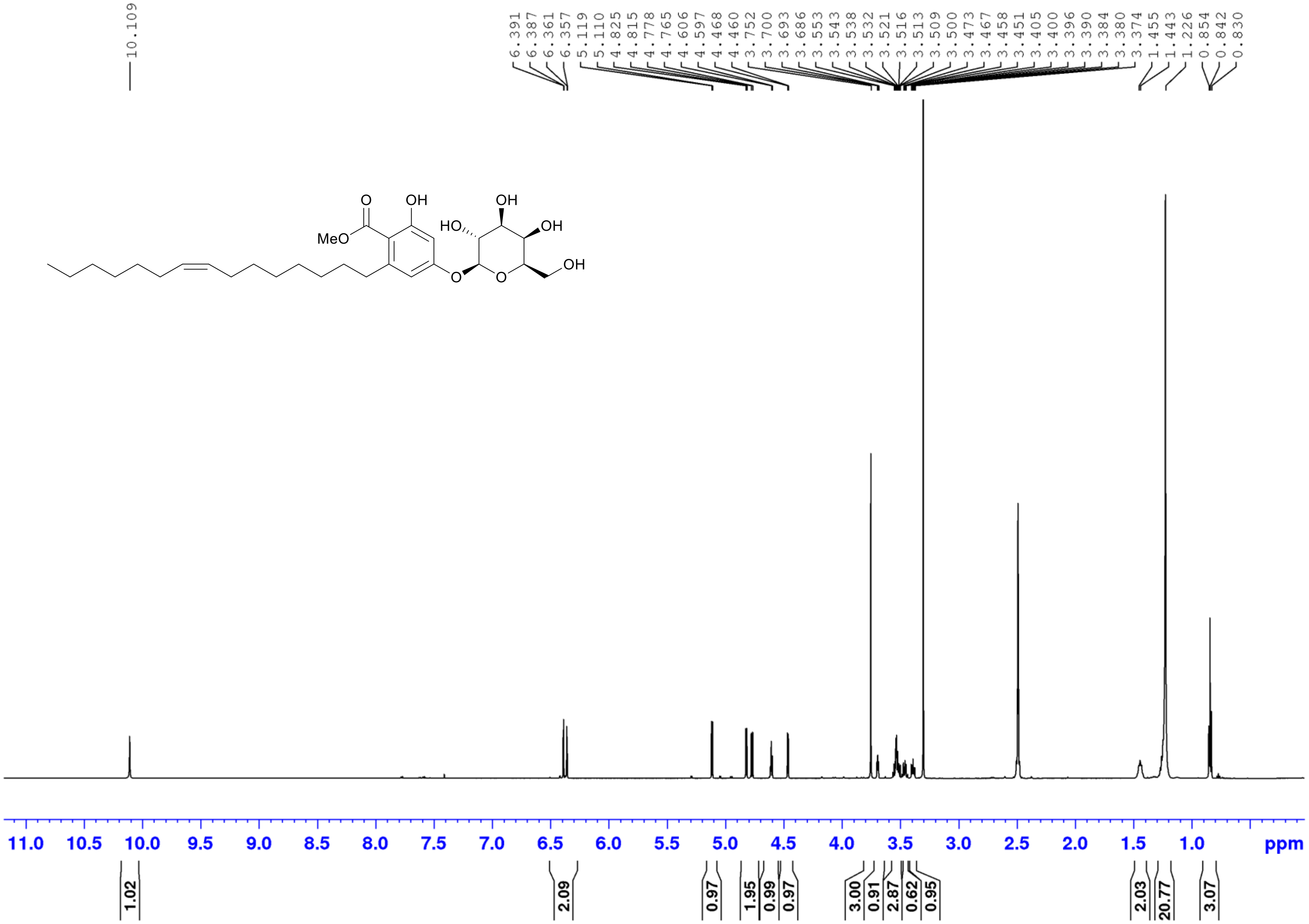

Figure S58. ^{1}H NMR spectrum (600 MHz) of capricostatin C (**4c**) in DMSO-d_6

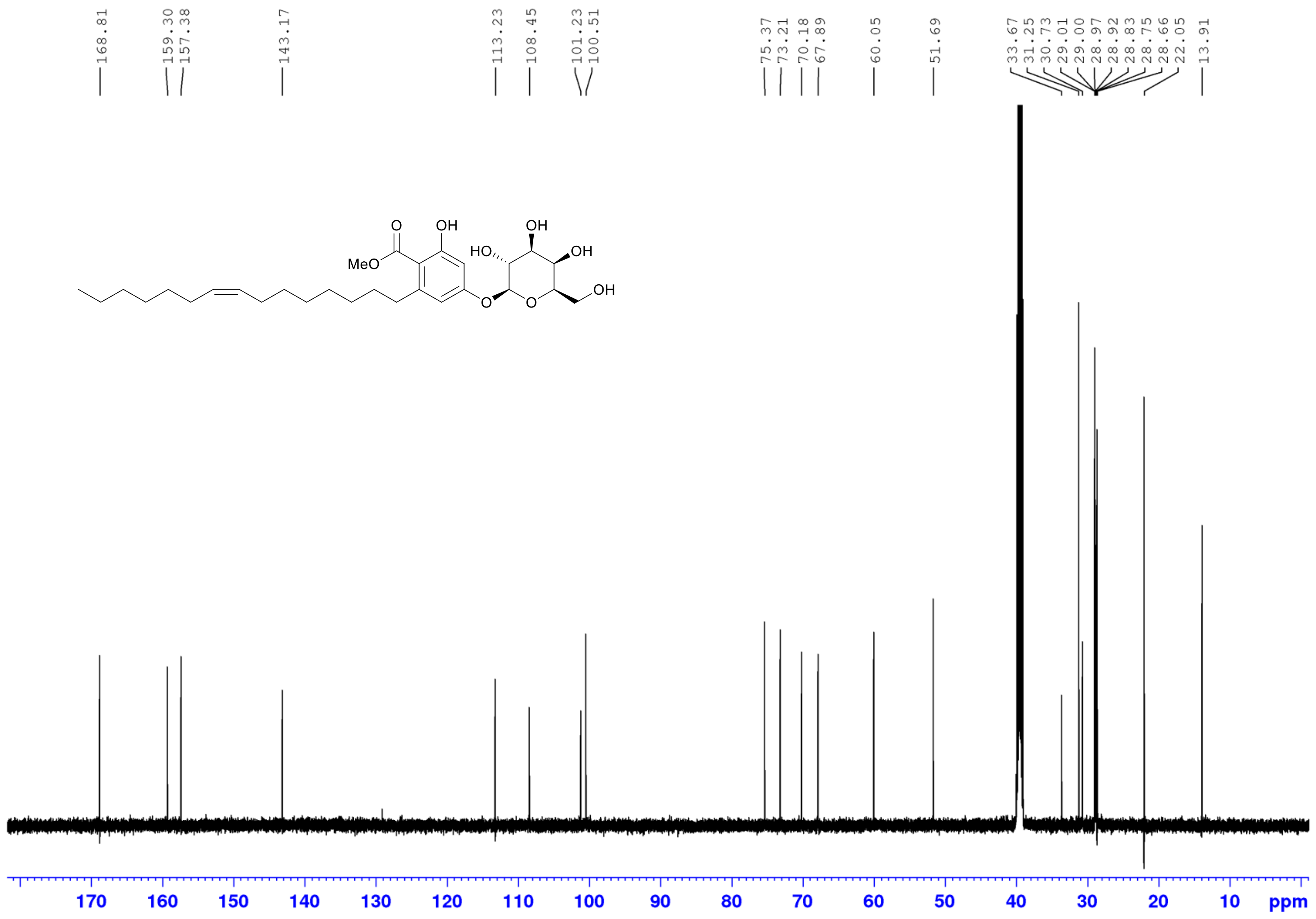

Figure S59. ^{13}C NMR spectrum (150 MHz) of capricostatin C (**4c**) in DMSO-d_6

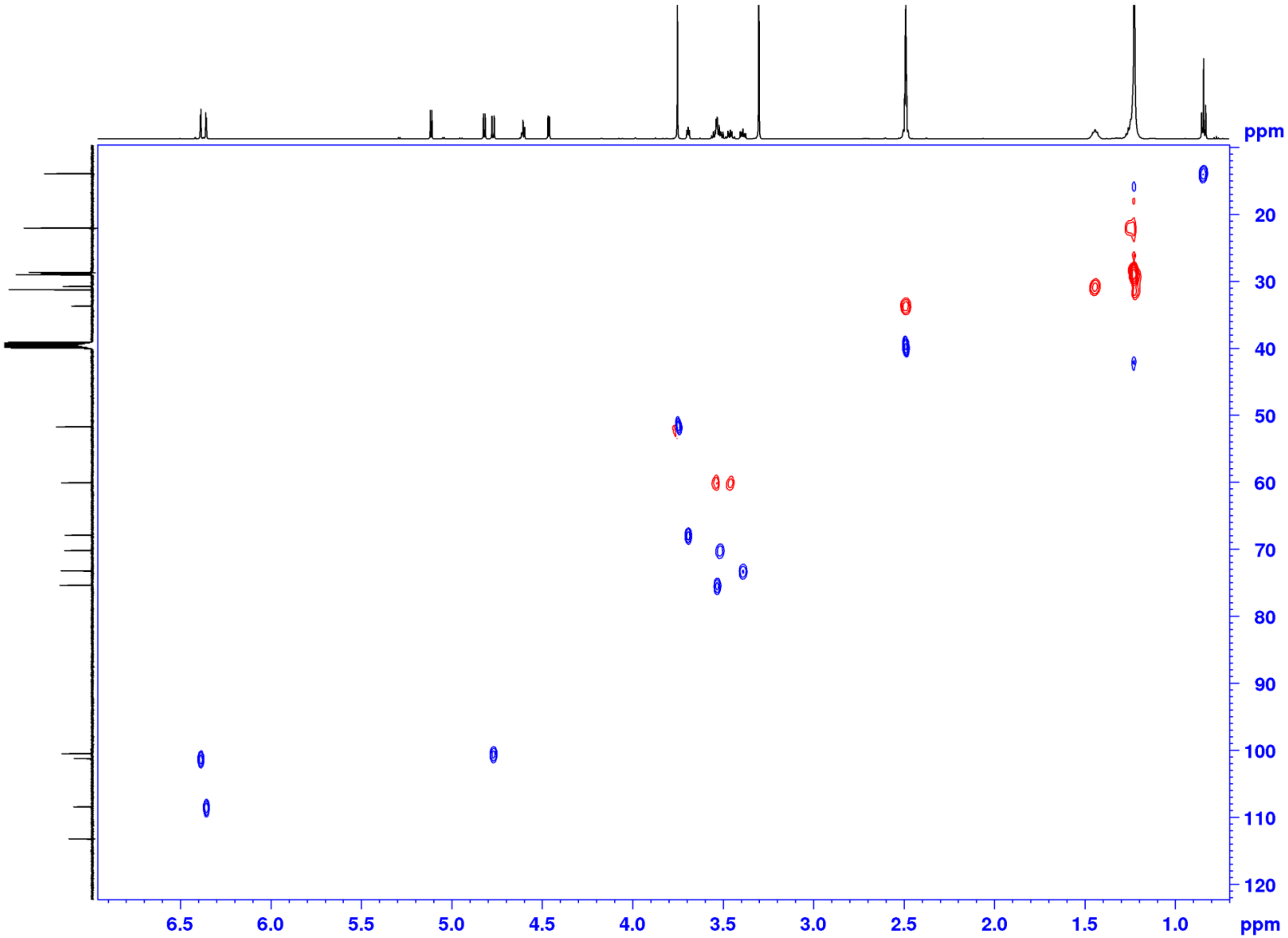

Figure S60. ^{1}H-^{13}C HSQC NMR spectrum (600 MHz) of capricostatin C (**4c**) in DMSO-d_6

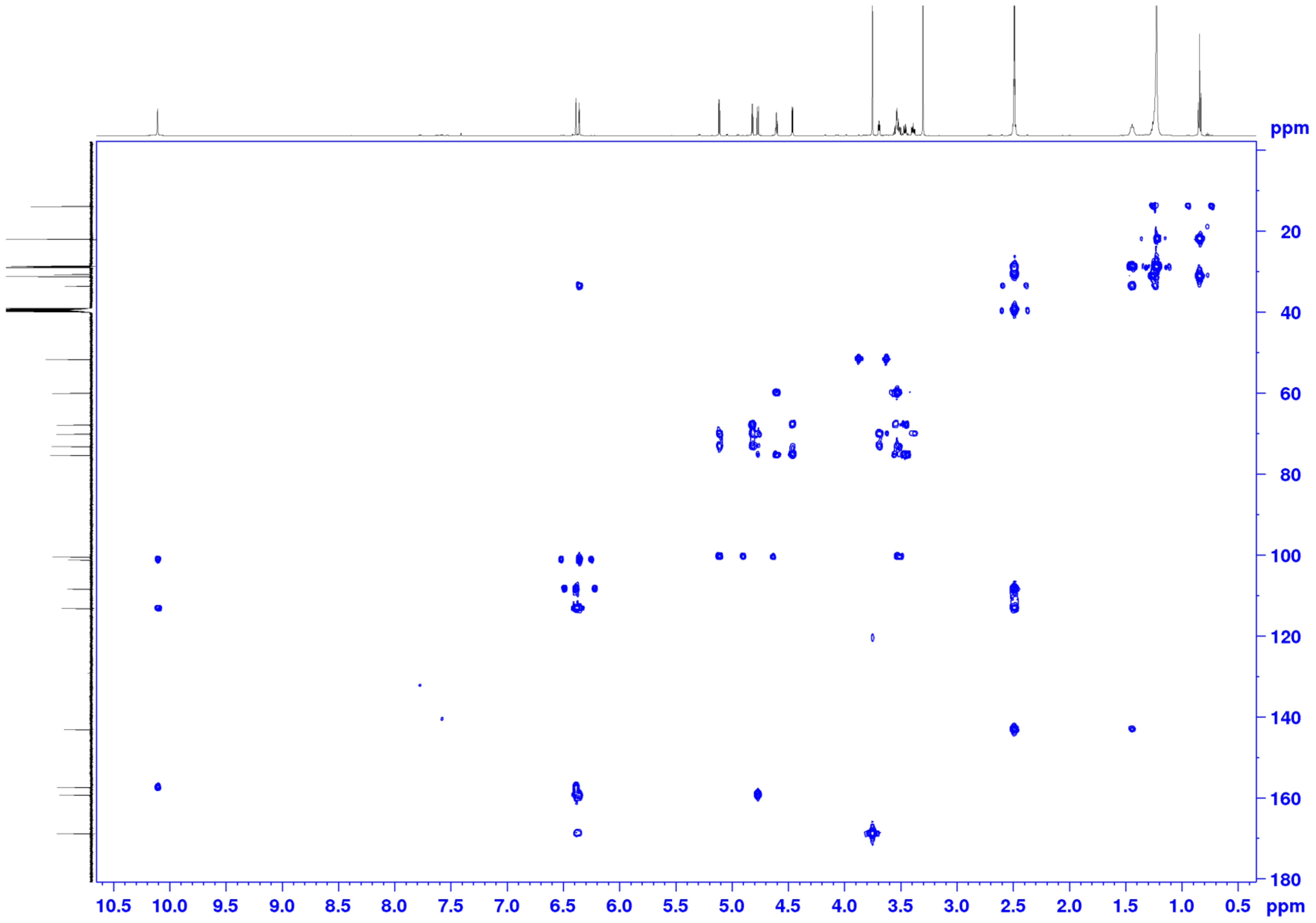

Figure S61. ^{1}H-^{13}C HSQC NMR spectrum (600 MHz) of capricostatin C (**4c**) in DMSO-d_6

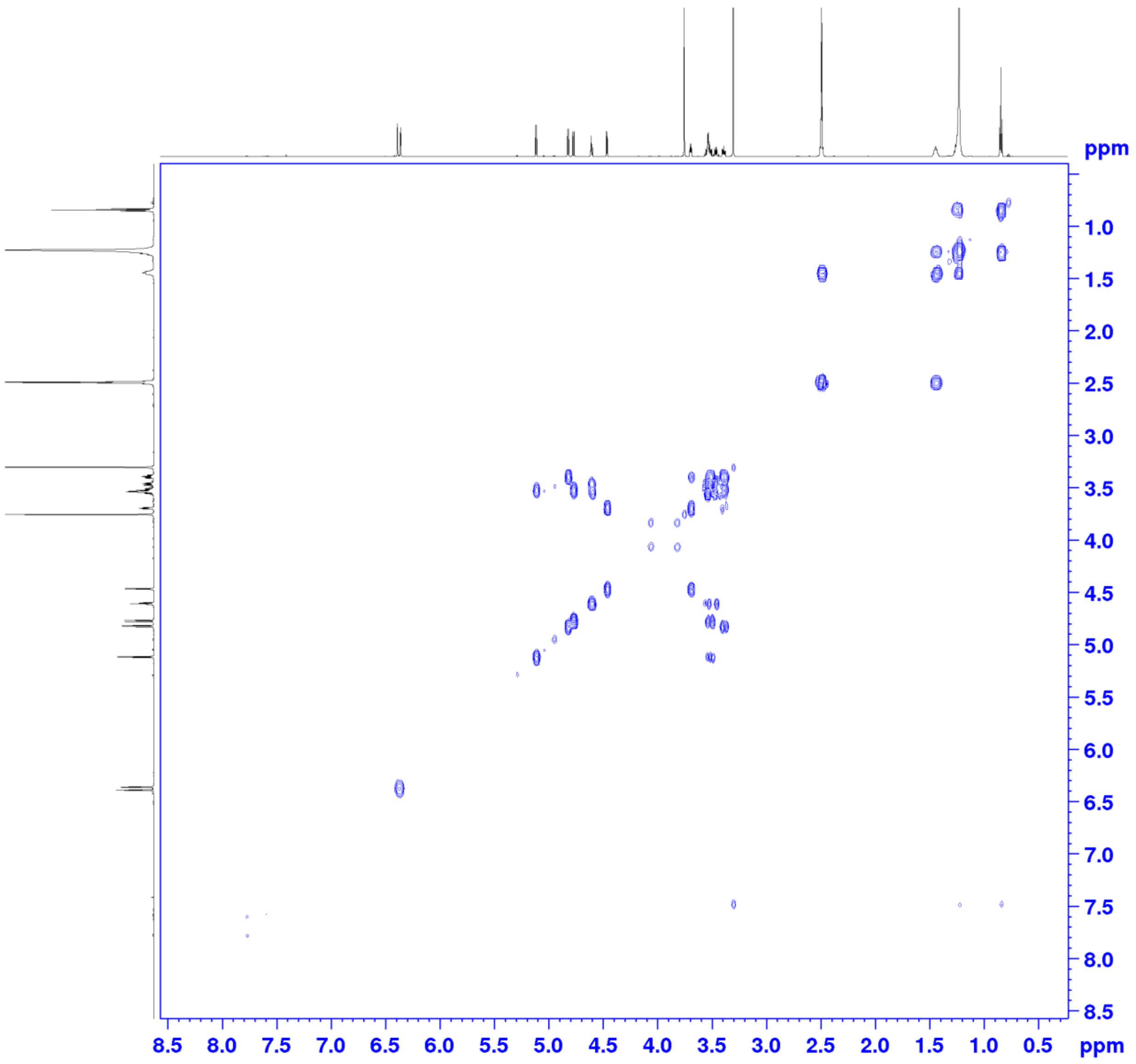

Figure S62. COSY NMR spectrum (600 MHz) of capricostatin C (**4c**) in DMSO-d_6

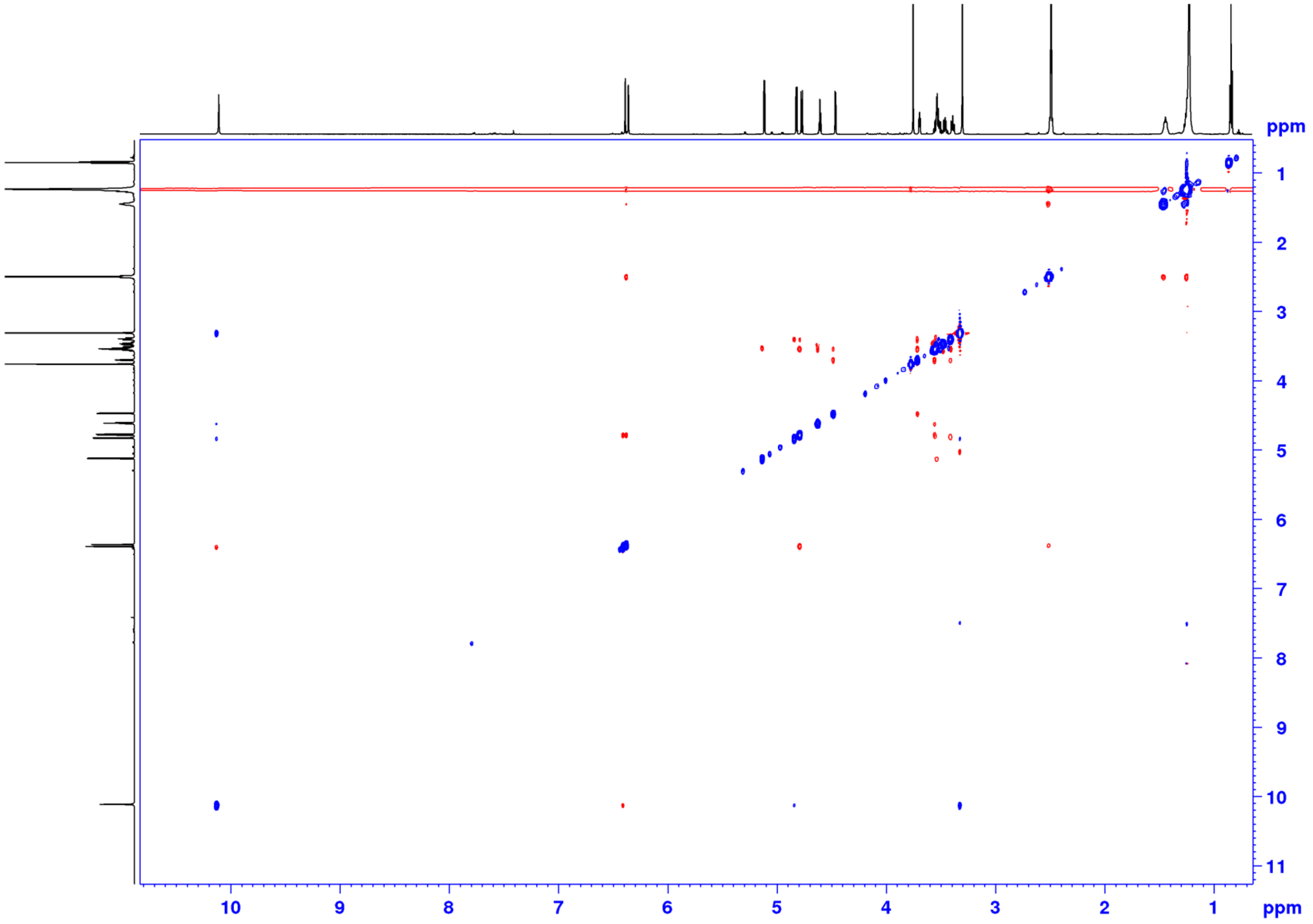

Figure S63. ROESY NMR spectrum (600 MHz) of capricostatin C (**4c**) in DMSO-d_6

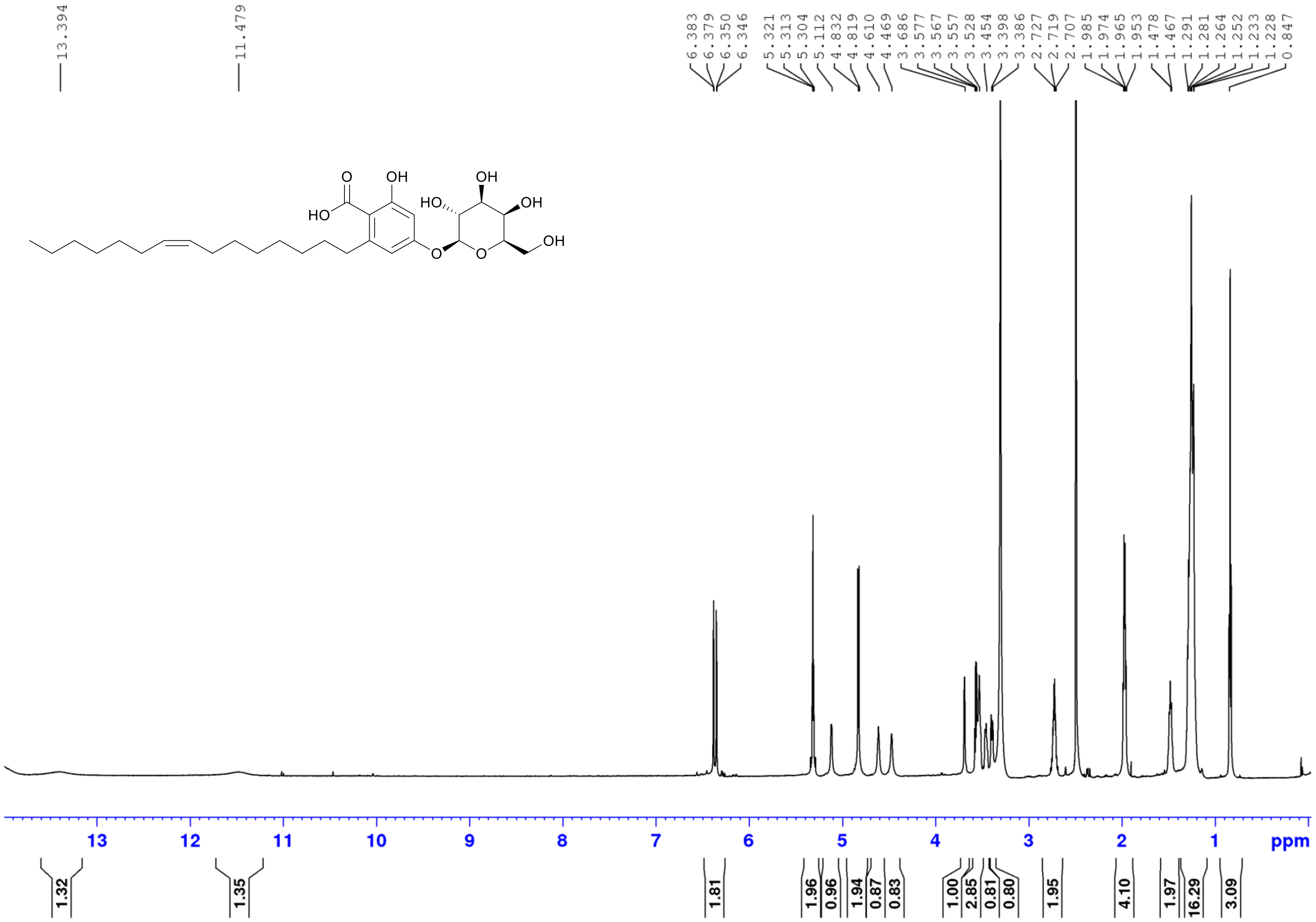

Figure S64. ^{1}H NMR spectrum (600 MHz) of capricostatin D (**4d**) in DMSO-d_6

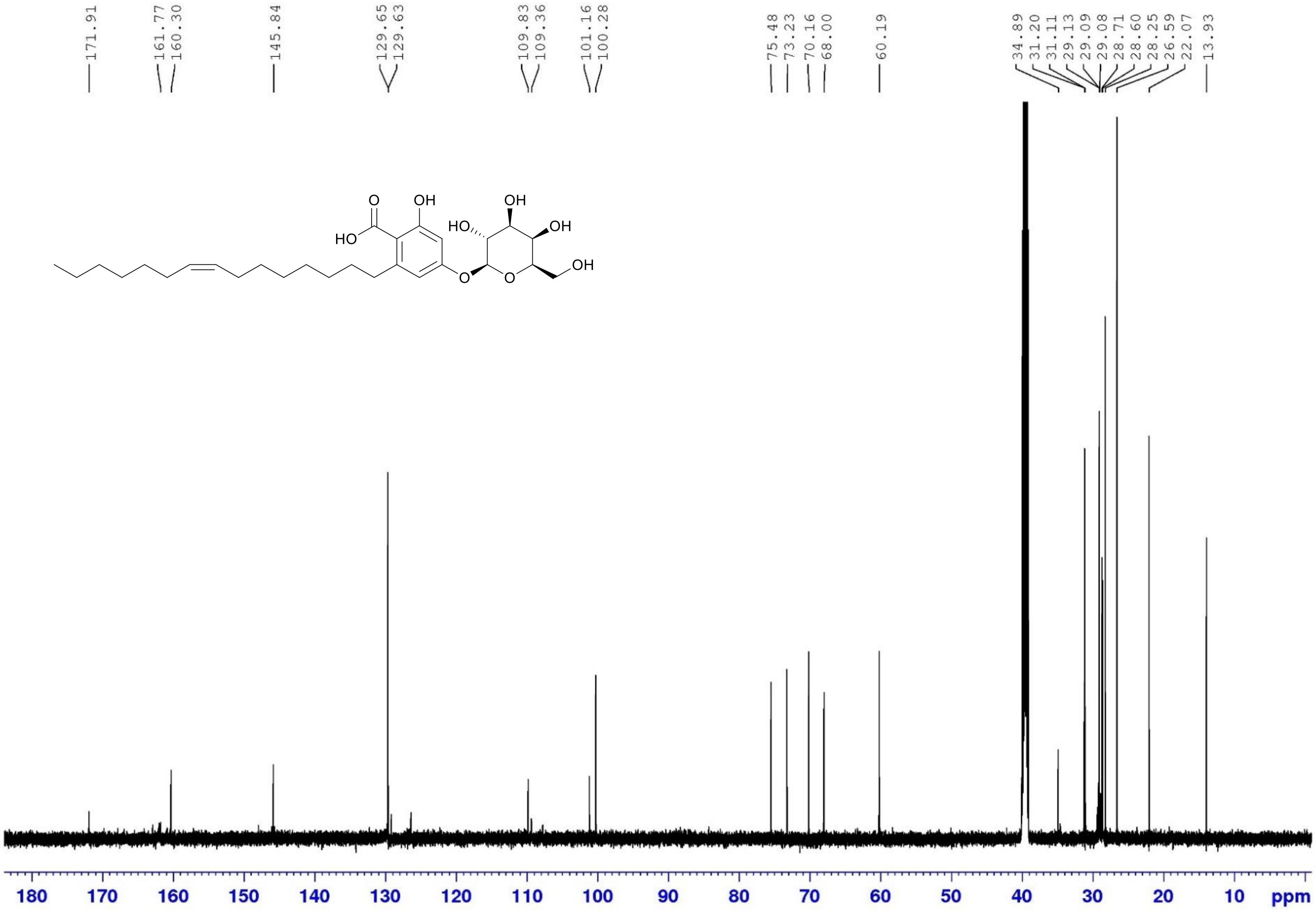

Figure S65. ^{13}C NMR spectrum (150 MHz) of capricostatin D (**4d**) in DMSO-d_6

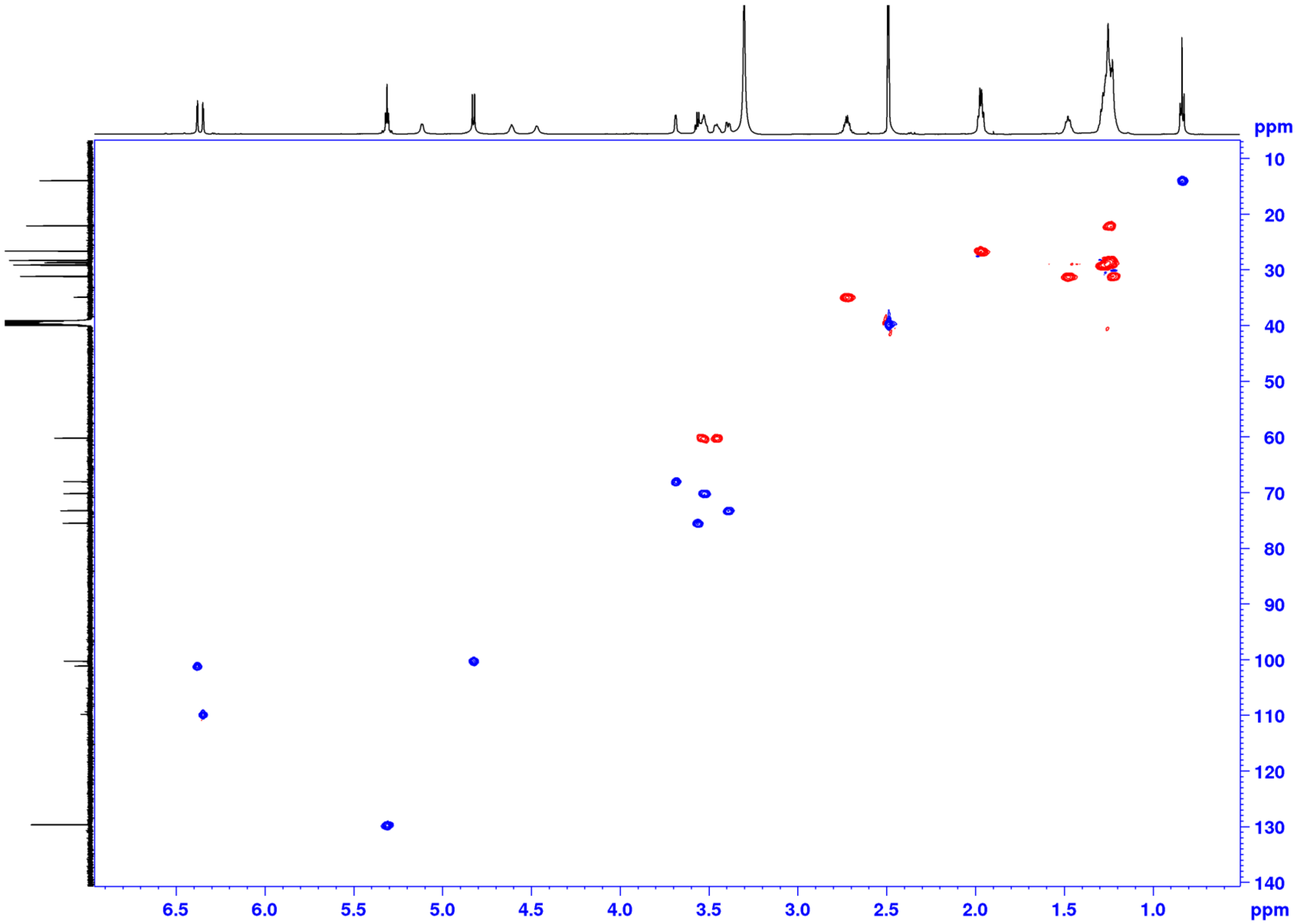

Figure S66. ^{1}H-^{13}C HSQC NMR spectrum (600 MHz) of capricostatin D (**4d**) in DMSO-d_6

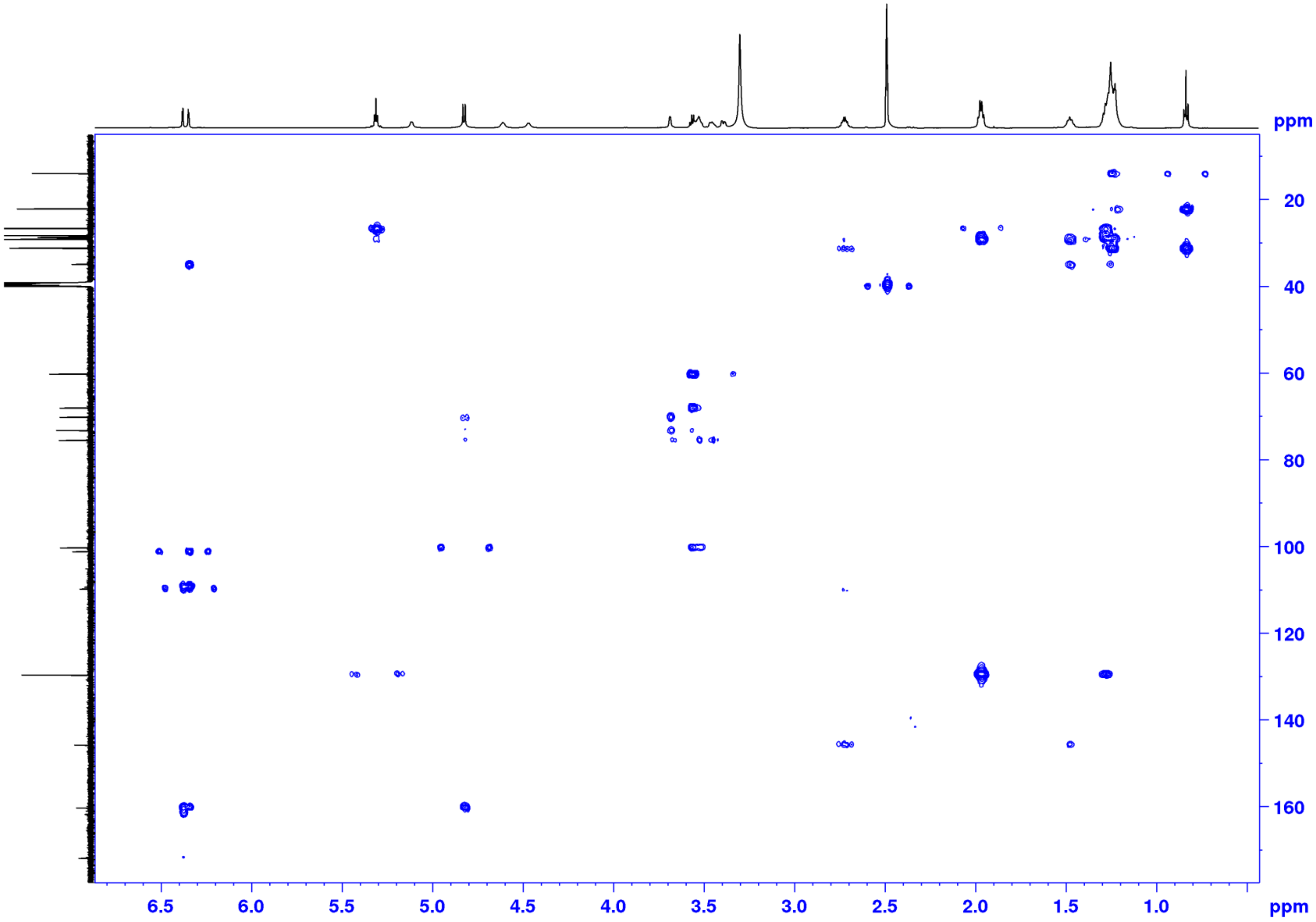

Figure S67. ^{1}H-^{13}C HSQC NMR spectrum (600 MHz) of capricostatin D (**4d**) in DMSO-d_6

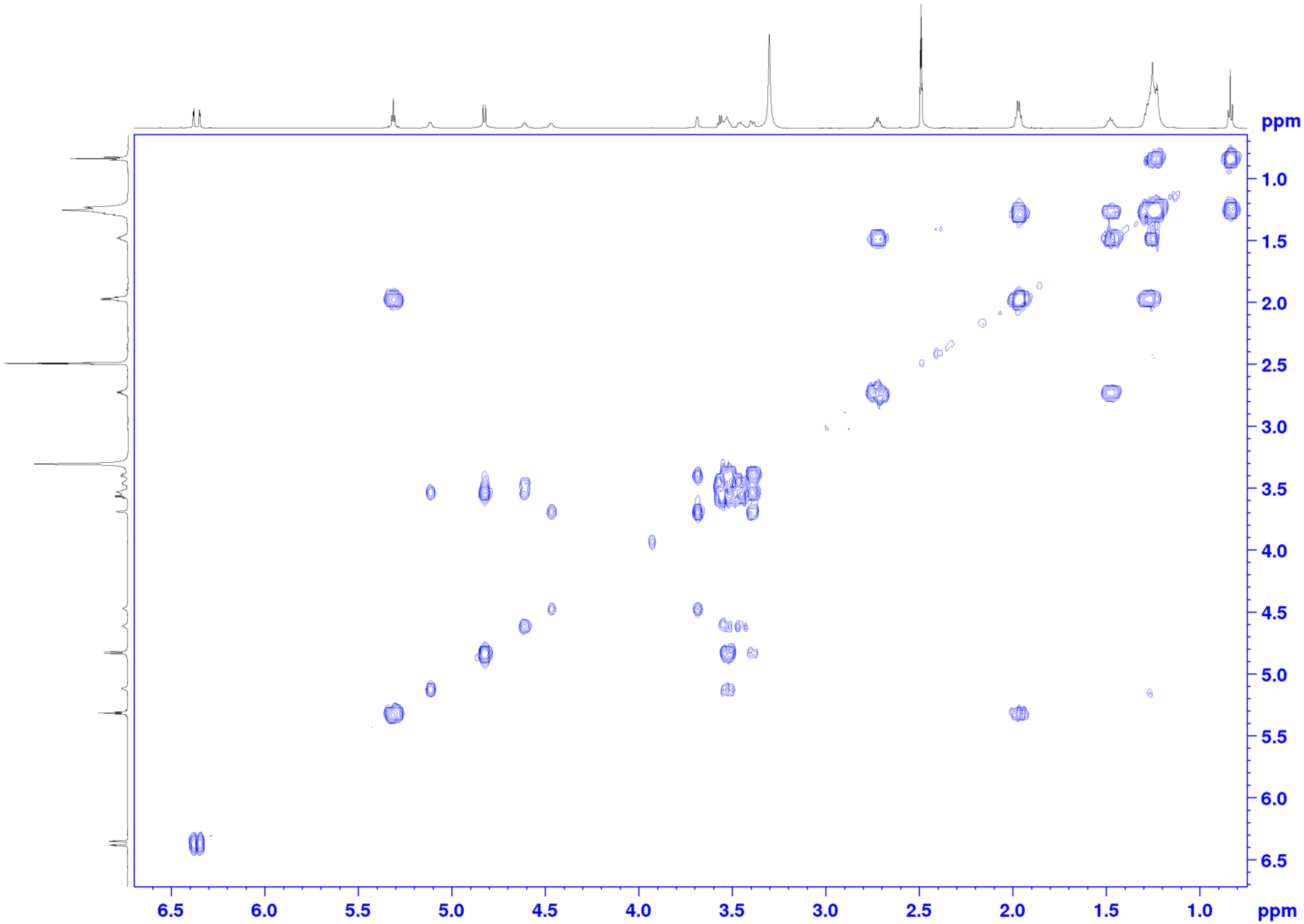

Figure S68. COSY NMR spectrum (600 MHz) of capricostatin D (**4d**) in DMSO-d_6

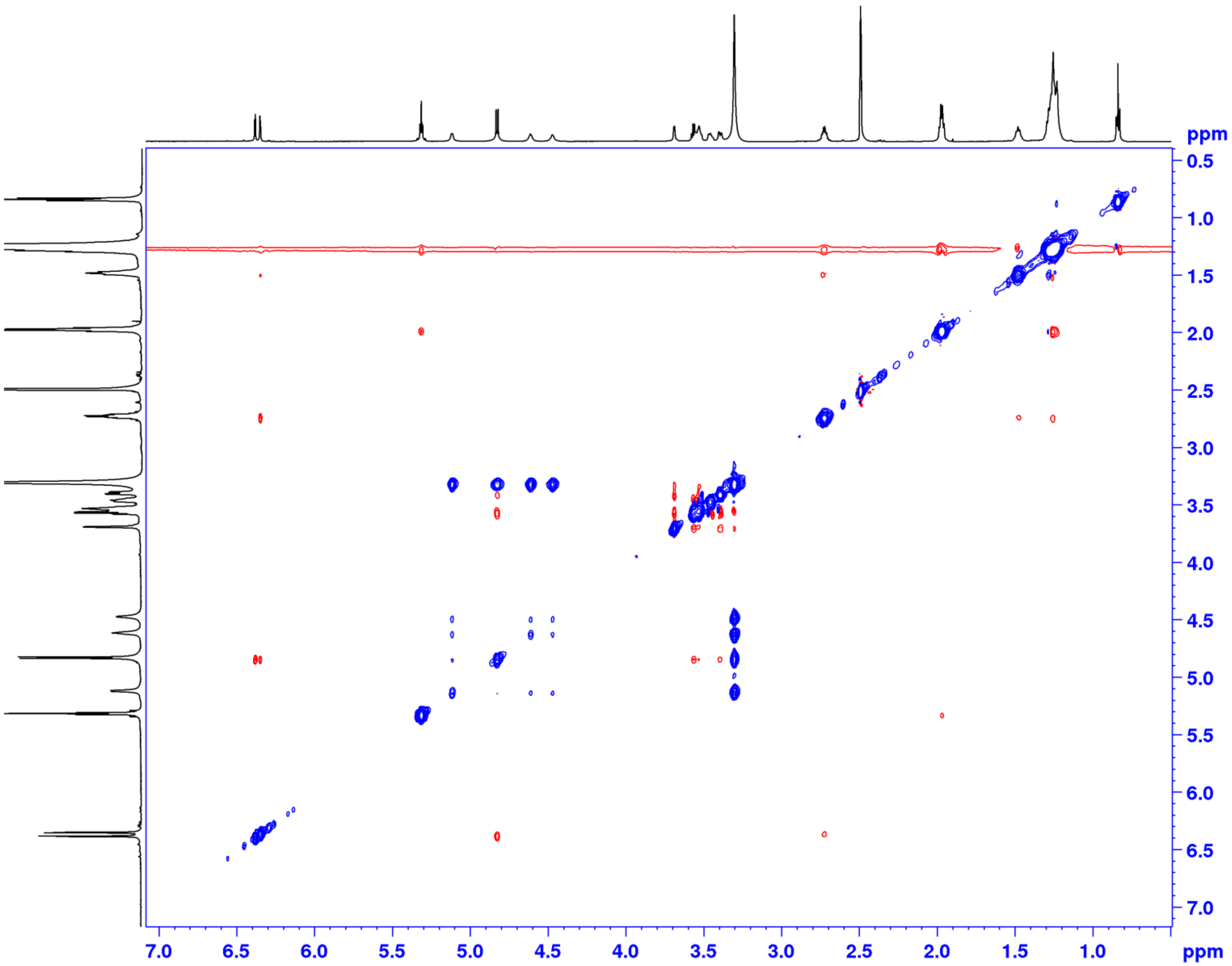

Figure S69. ROESY NMR spectrum (600 MHz) of capricostatin D (**4d**) in DMSO-d_6

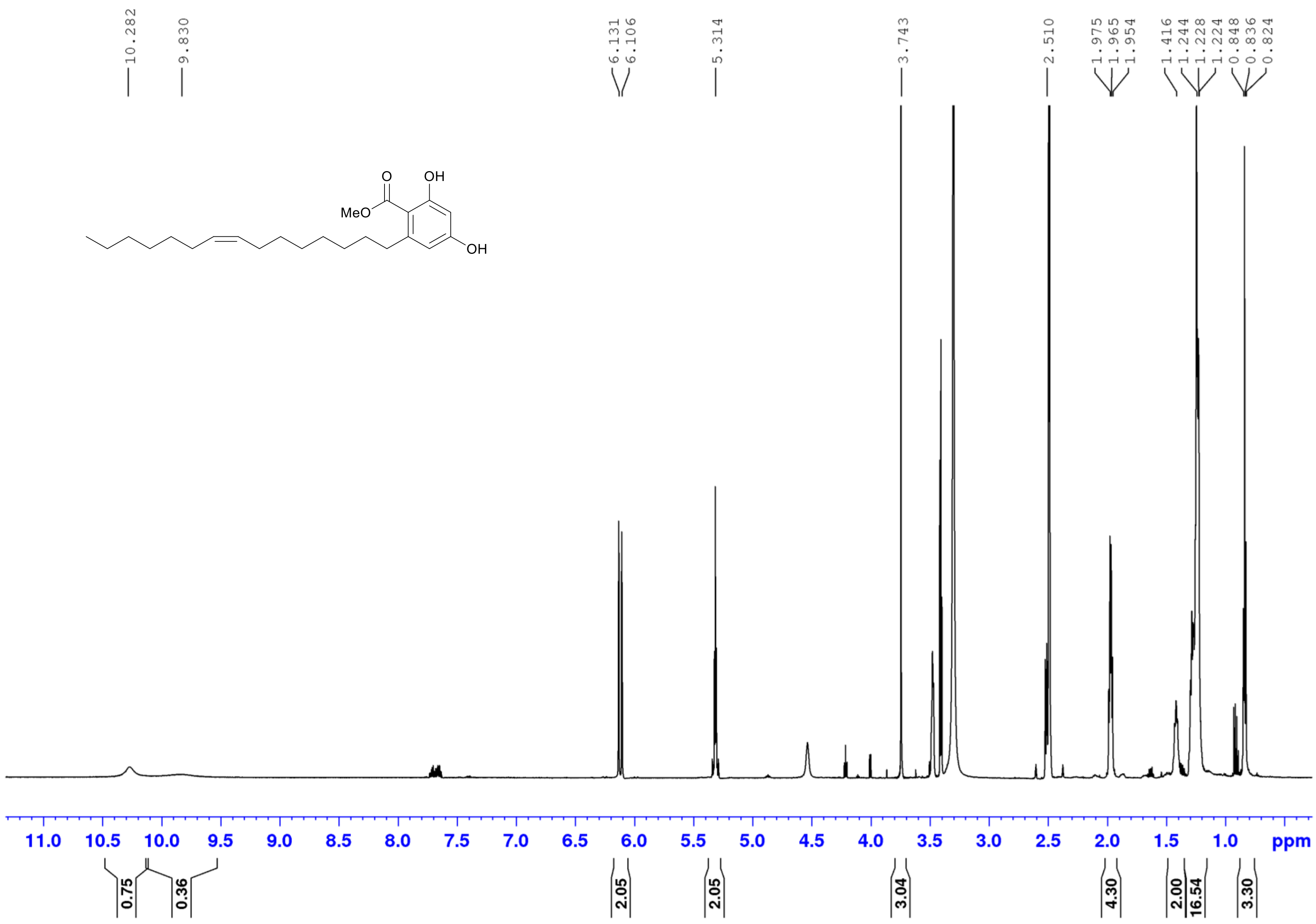

Figure S70. ^{1}H NMR spectrum (600 MHz) of capricostatin E (**4e**) in DMSO-d_6

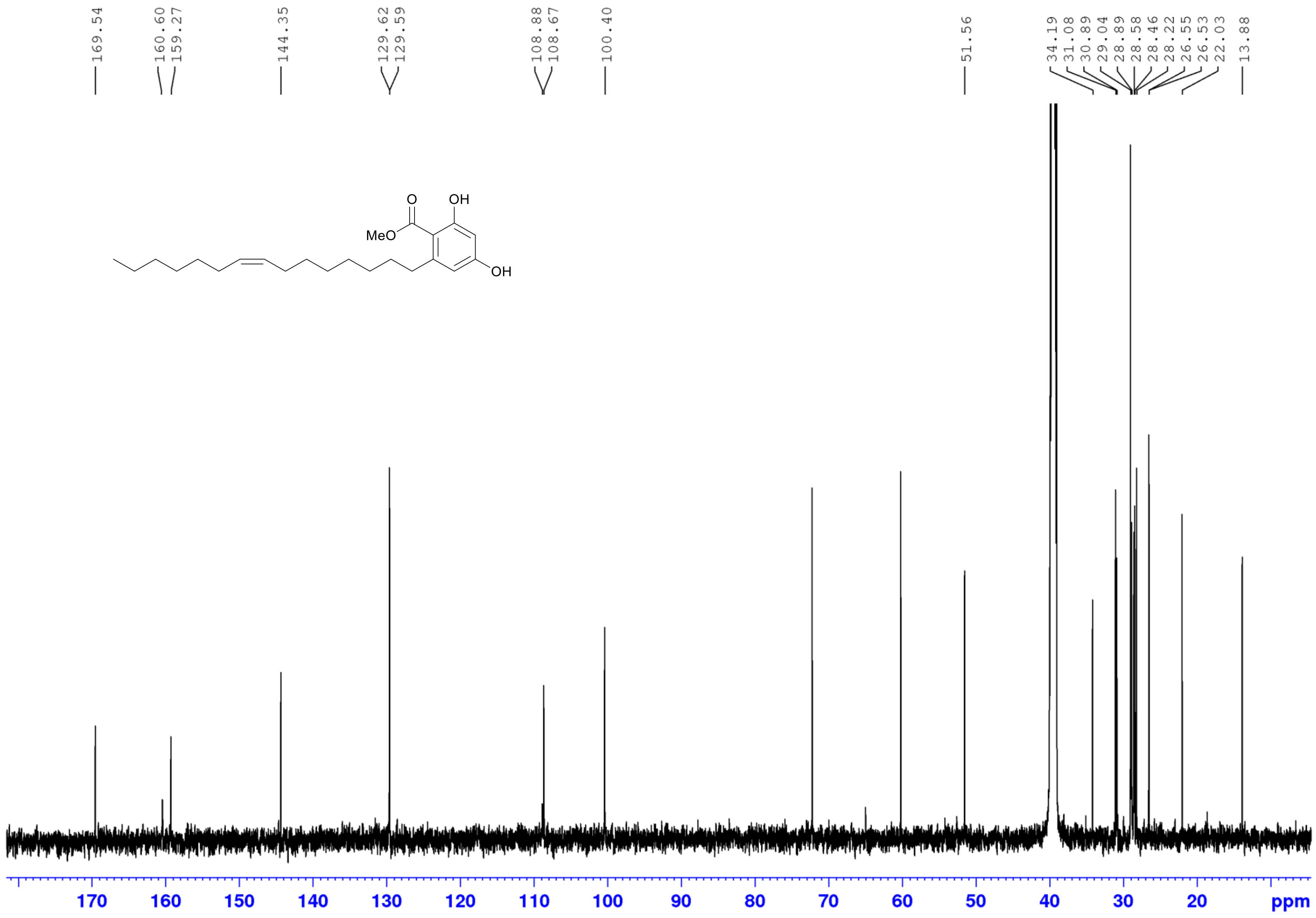

Figure S71. ^{13}C NMR spectrum (150 MHz) of capricostatin E (**4e**) in DMSO-d_6

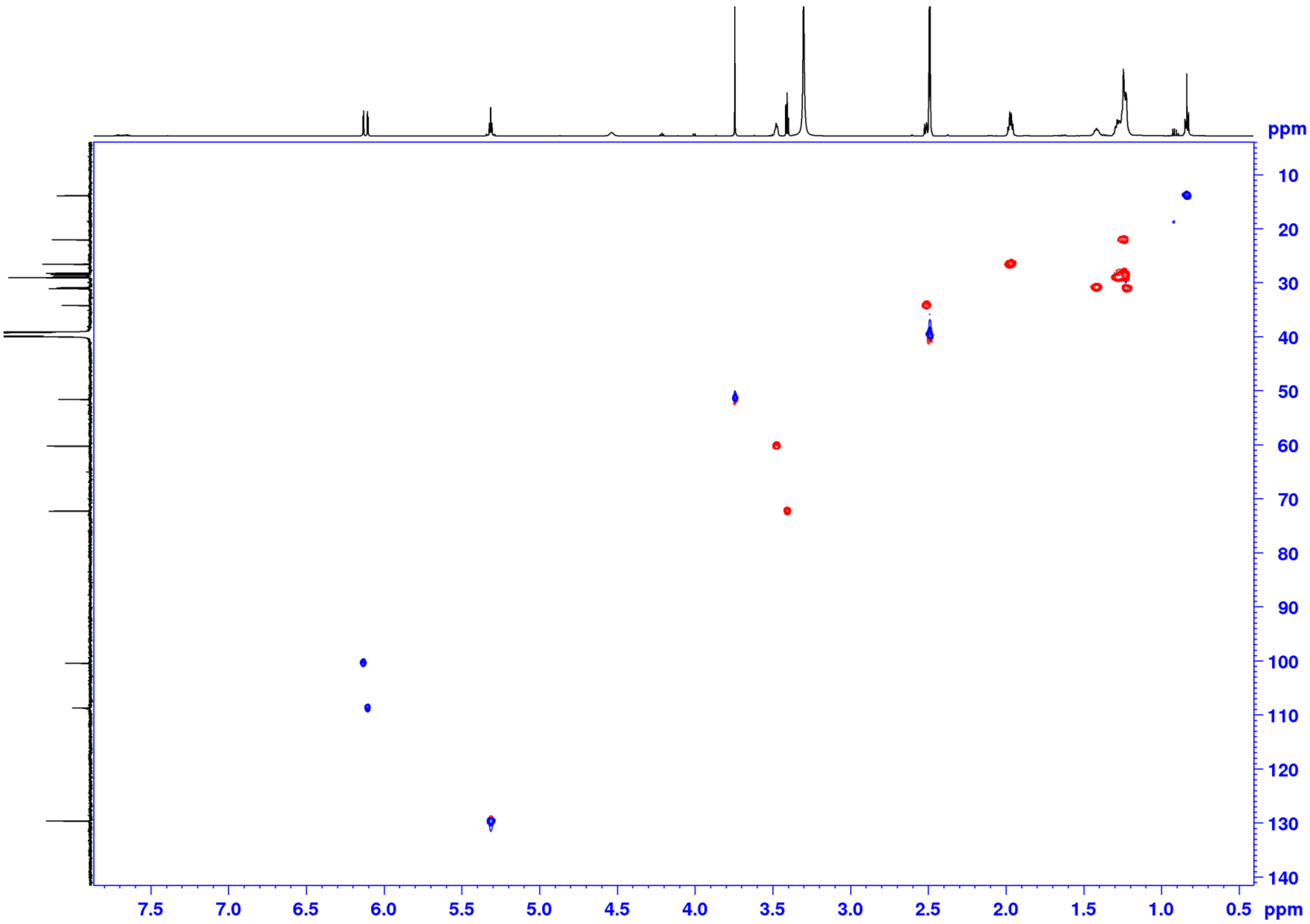

Figure S72. ^{1}H-^{13}C HSQC NMR spectrum (600 MHz) of capricostatin E (**4e**) in DMSO-d_6

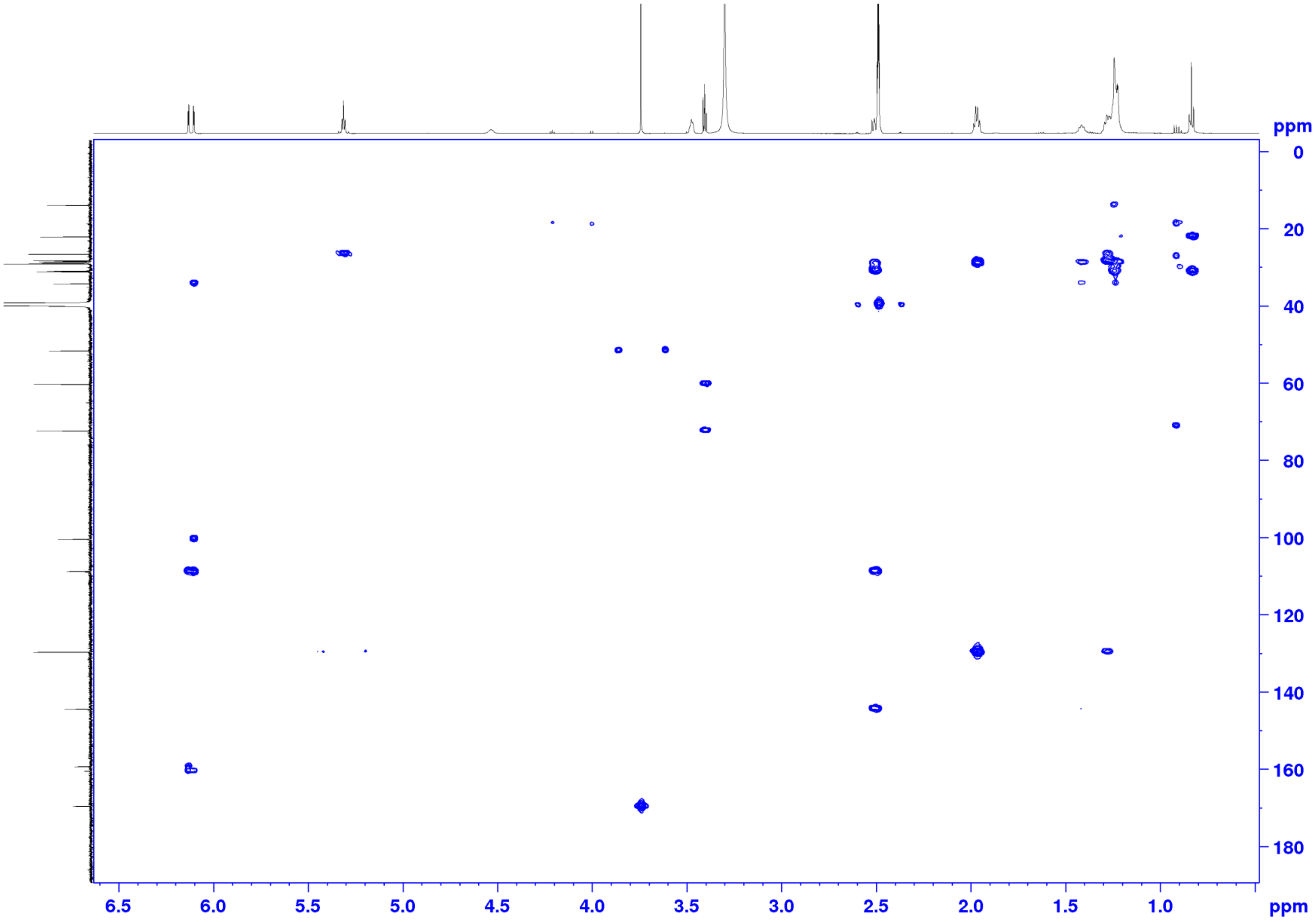

Figure S73. ^1H-^{13}C HSQC NMR spectrum (600 MHz) of capricostatin E (**4e**) in DMSO-d_6

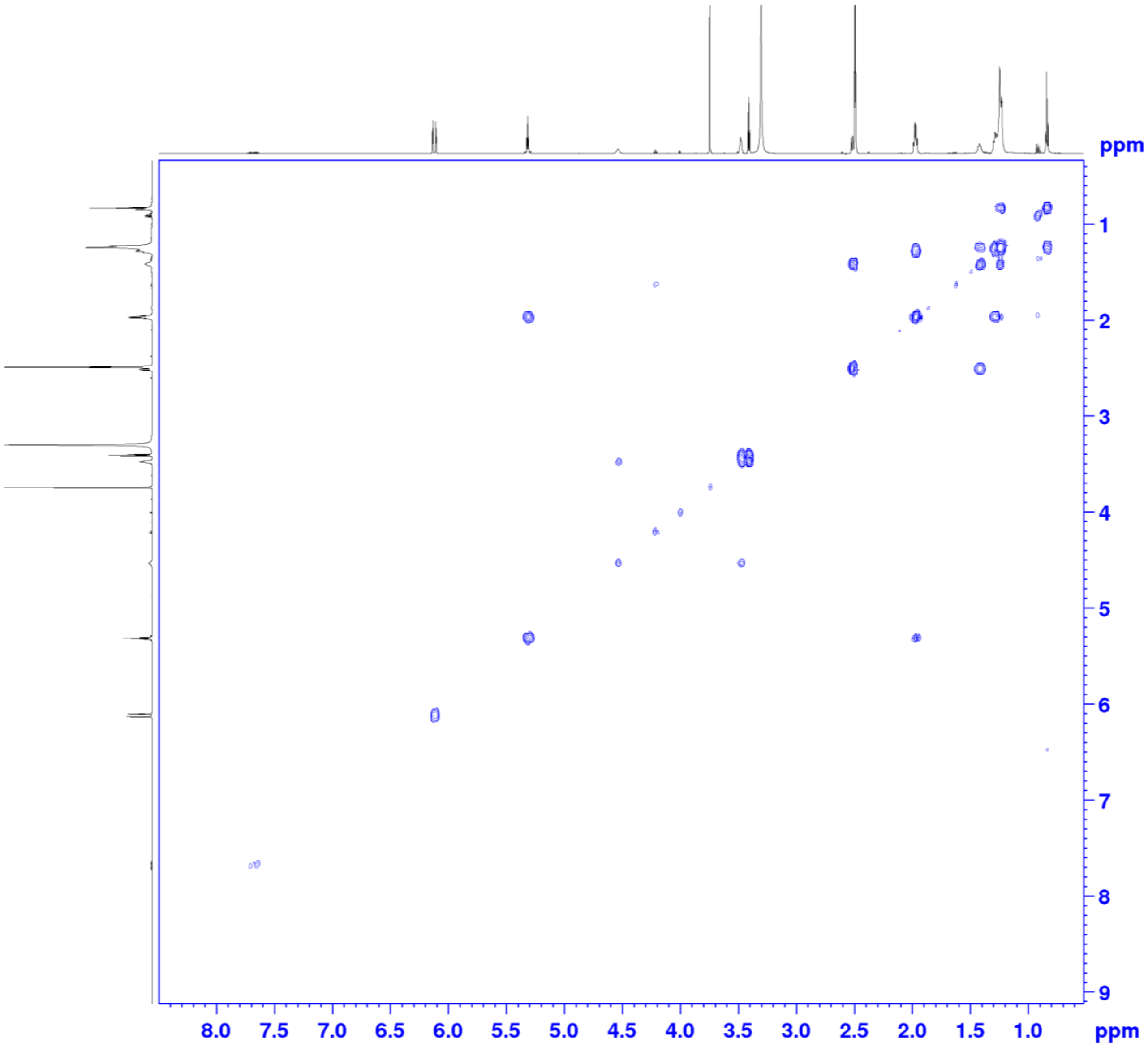

Figure S74. COSY NMR spectrum (600 MHz) of capricostatin E (**4e**) in DMSO-d_6

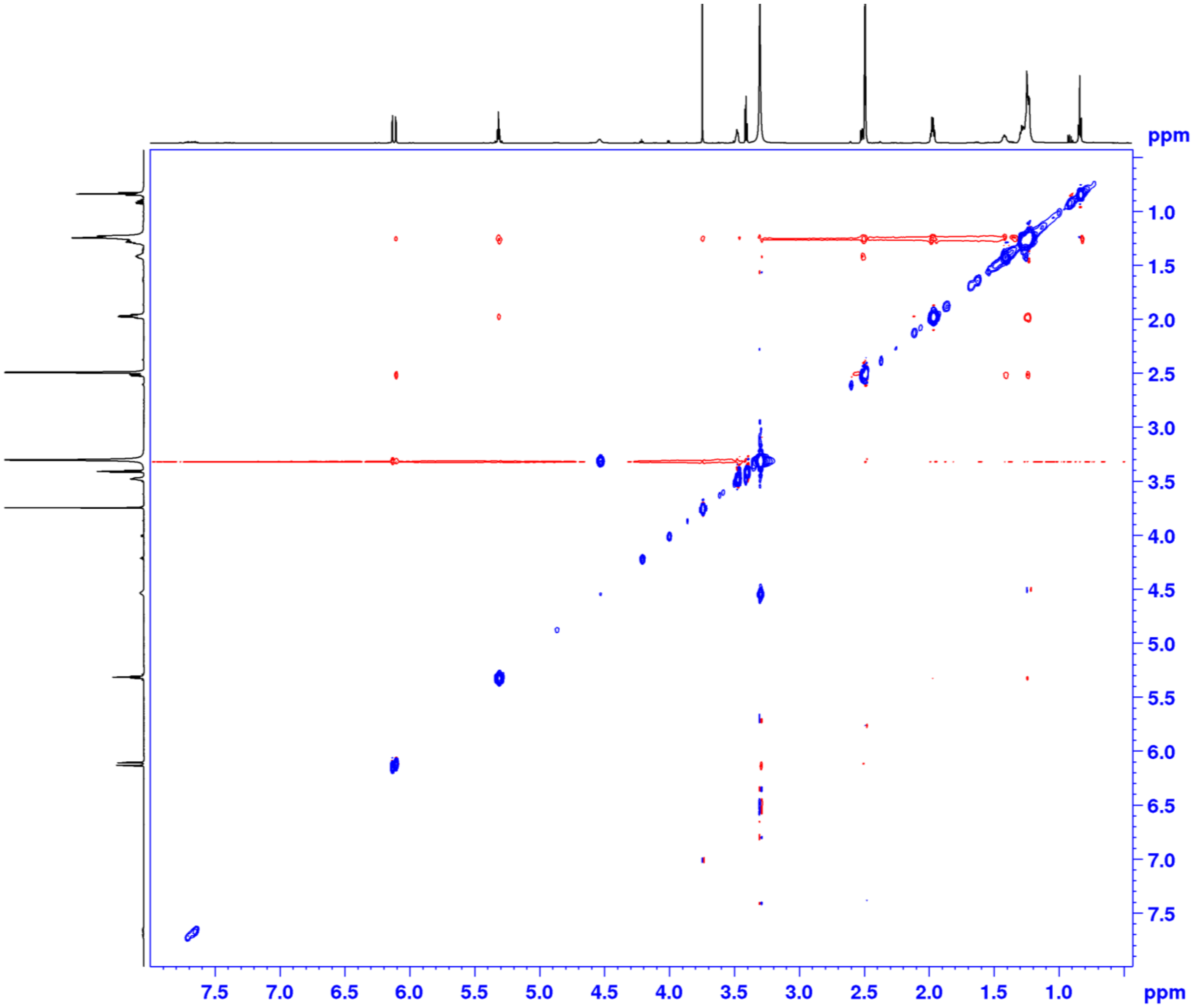

Figure S75. ROESY NMR spectrum (600 MHz) of capricostatin E (**4e**) in DMSO-d_6

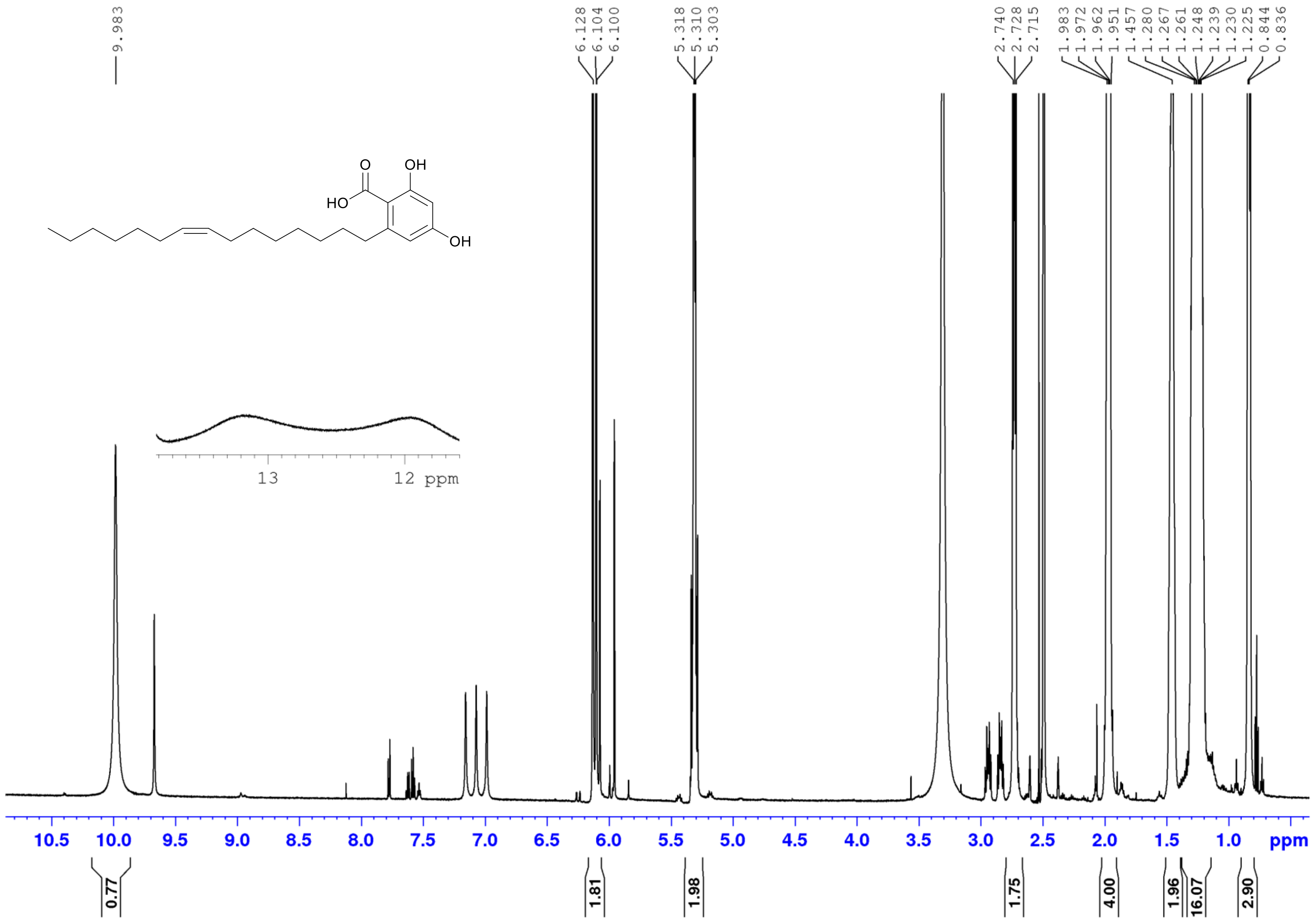

Figure S76. ^{1}H NMR spectrum (600 MHz) of capricostatin F (**4f**) in DMSO-d_6

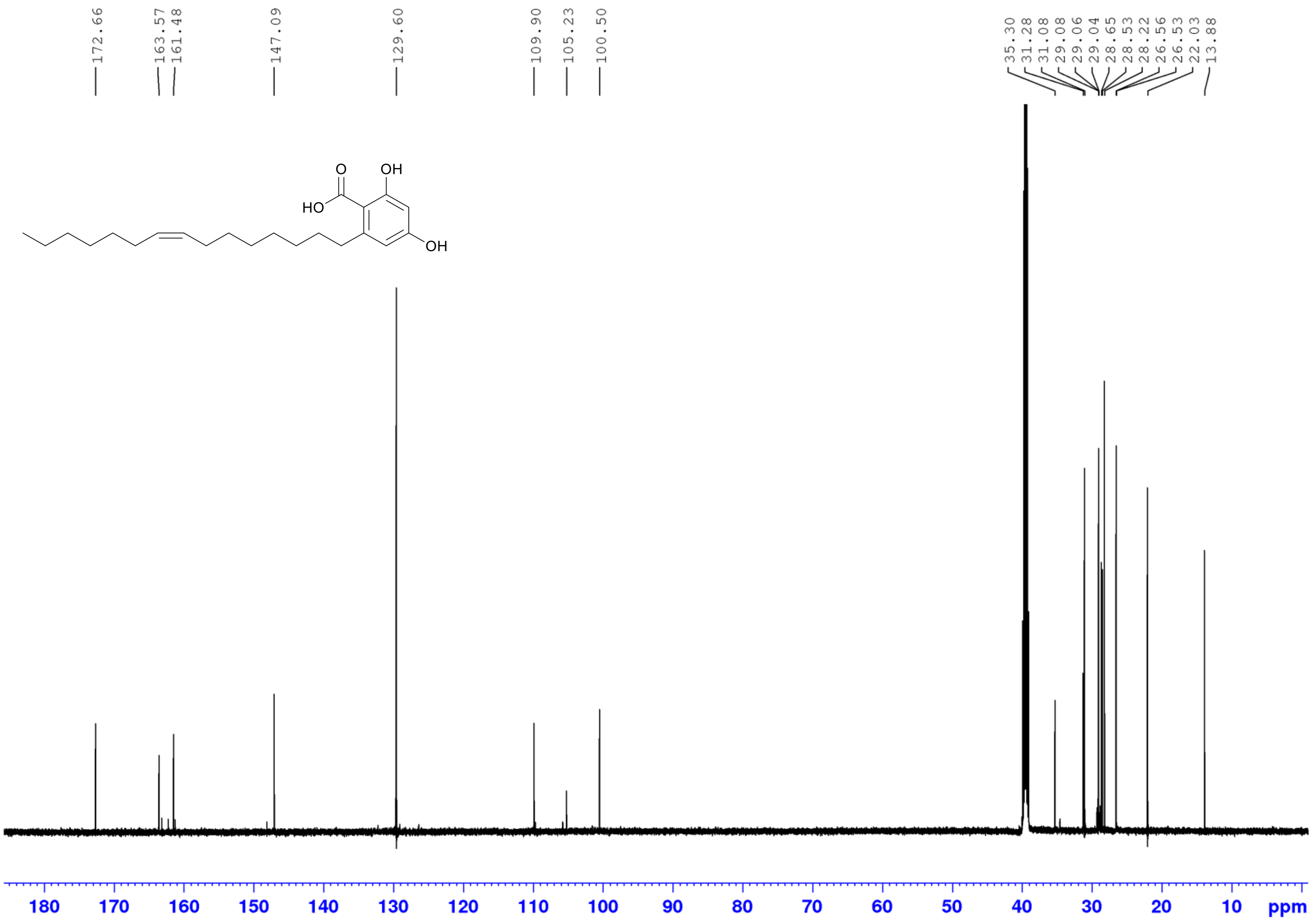

Figure S77. ^{13}C NMR spectrum (150 MHz) of capricostatin F (**4f**) in DMSO-d_6

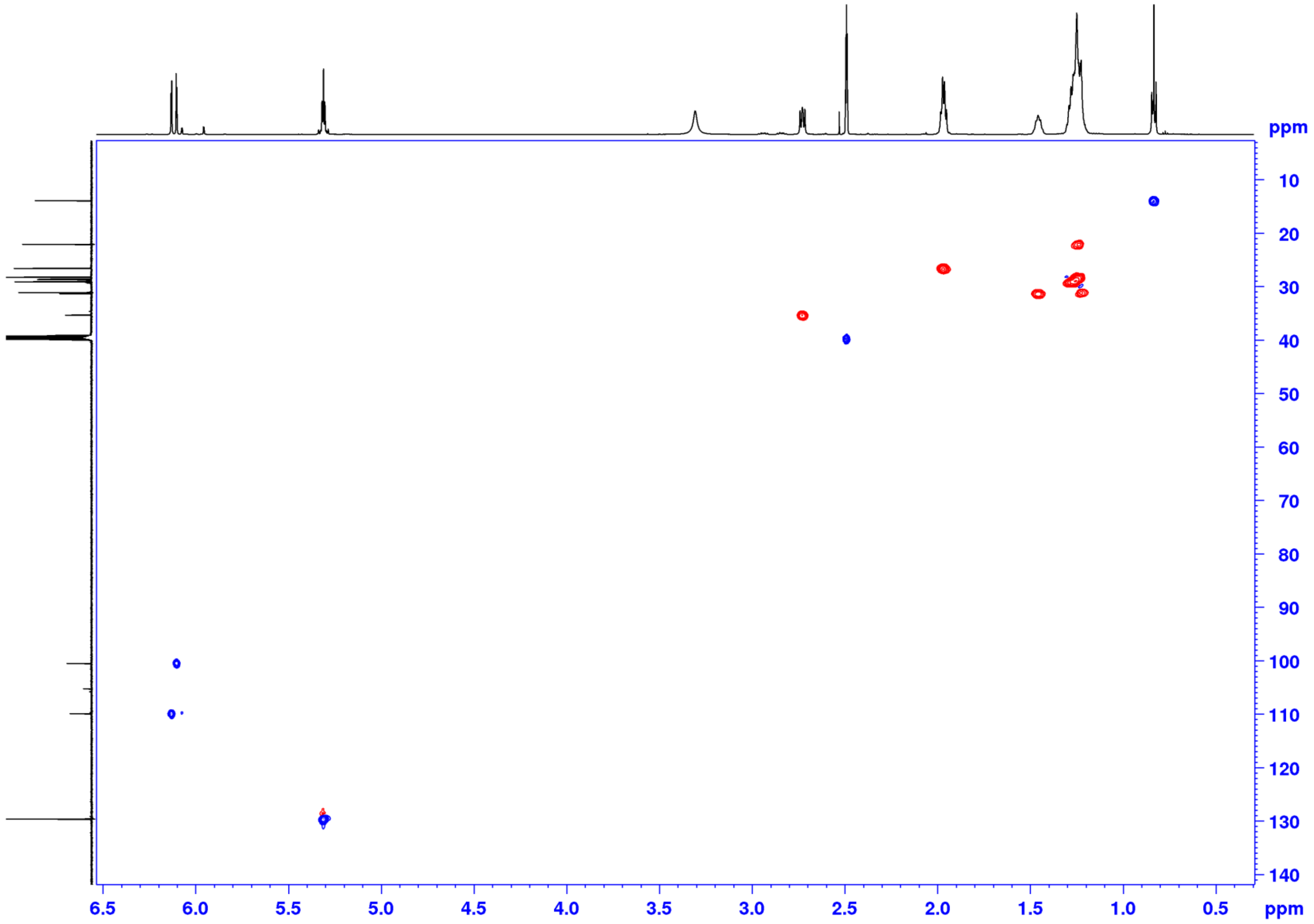

Figure S78. ^{1}H-^{13}C HSQC NMR spectrum (600 MHz) of capricostatin F (**4f**) in DMSO-d_6

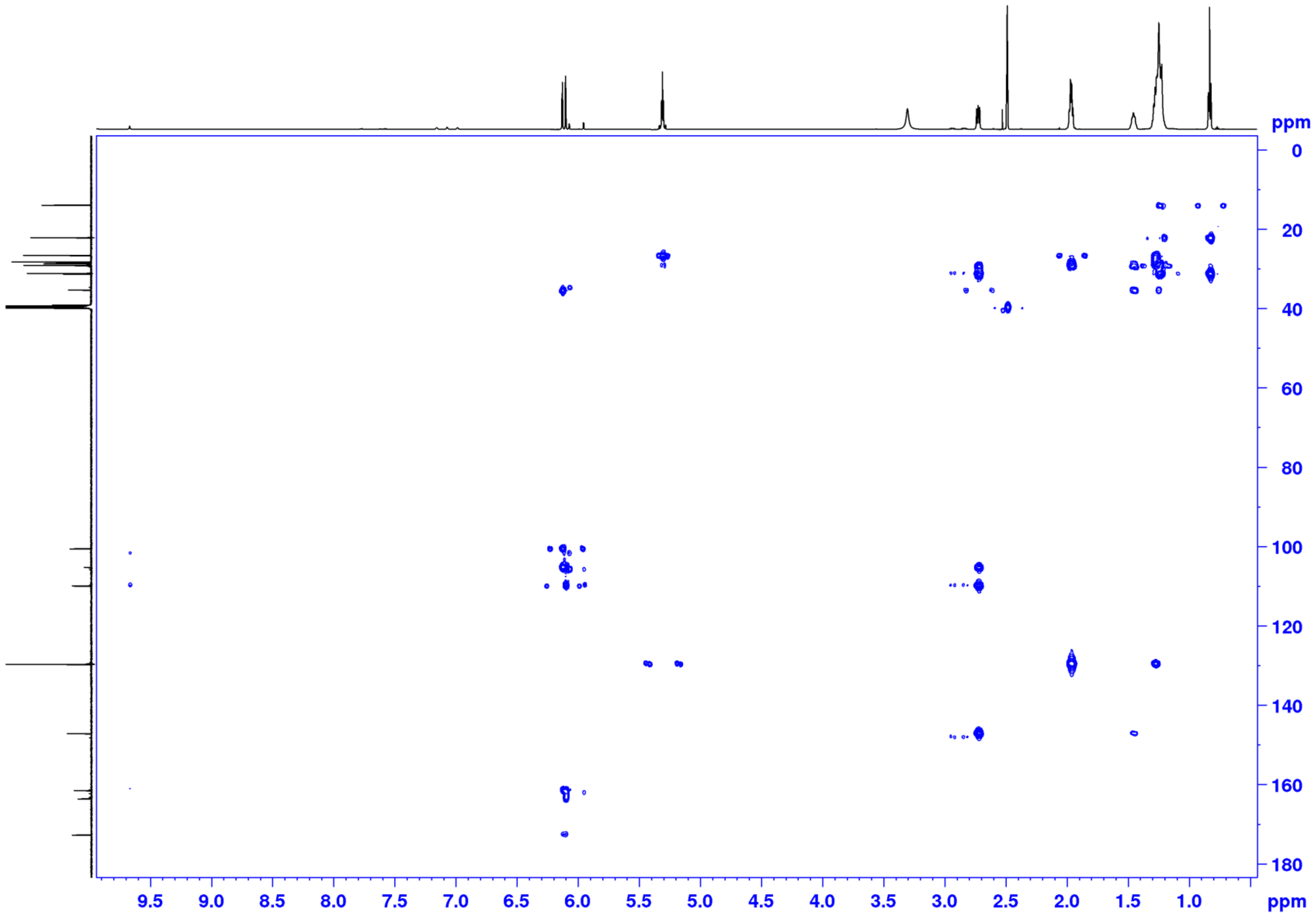

Figure S79. ^{1}H-^{13}C HSQC NMR spectrum (600 MHz) of capricostatin F (**4f**) in DMSO-d_6

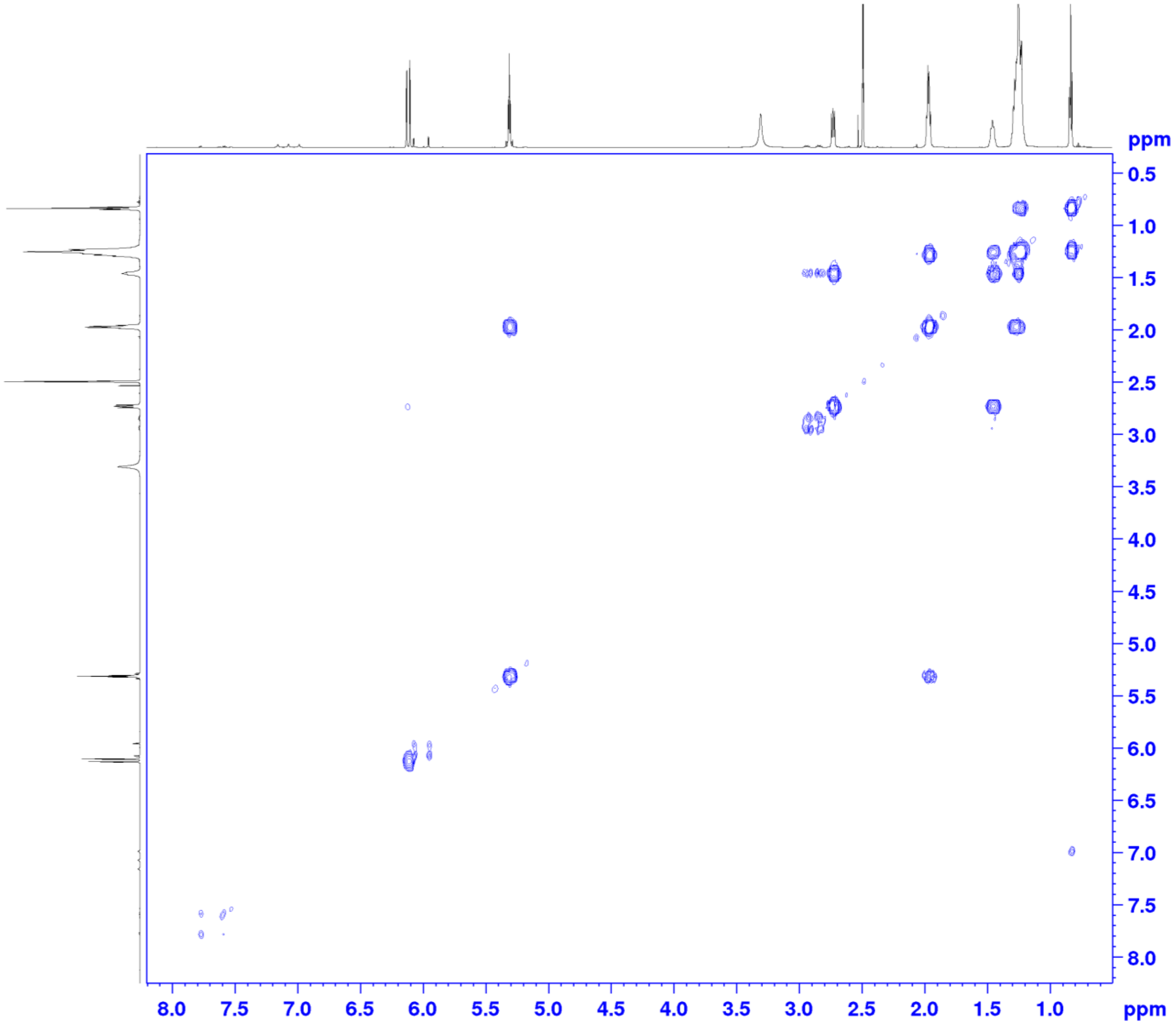

Figure S80. COSY NMR spectrum (600 MHz) of capricostatin F (**4f**) in DMSO-d_6

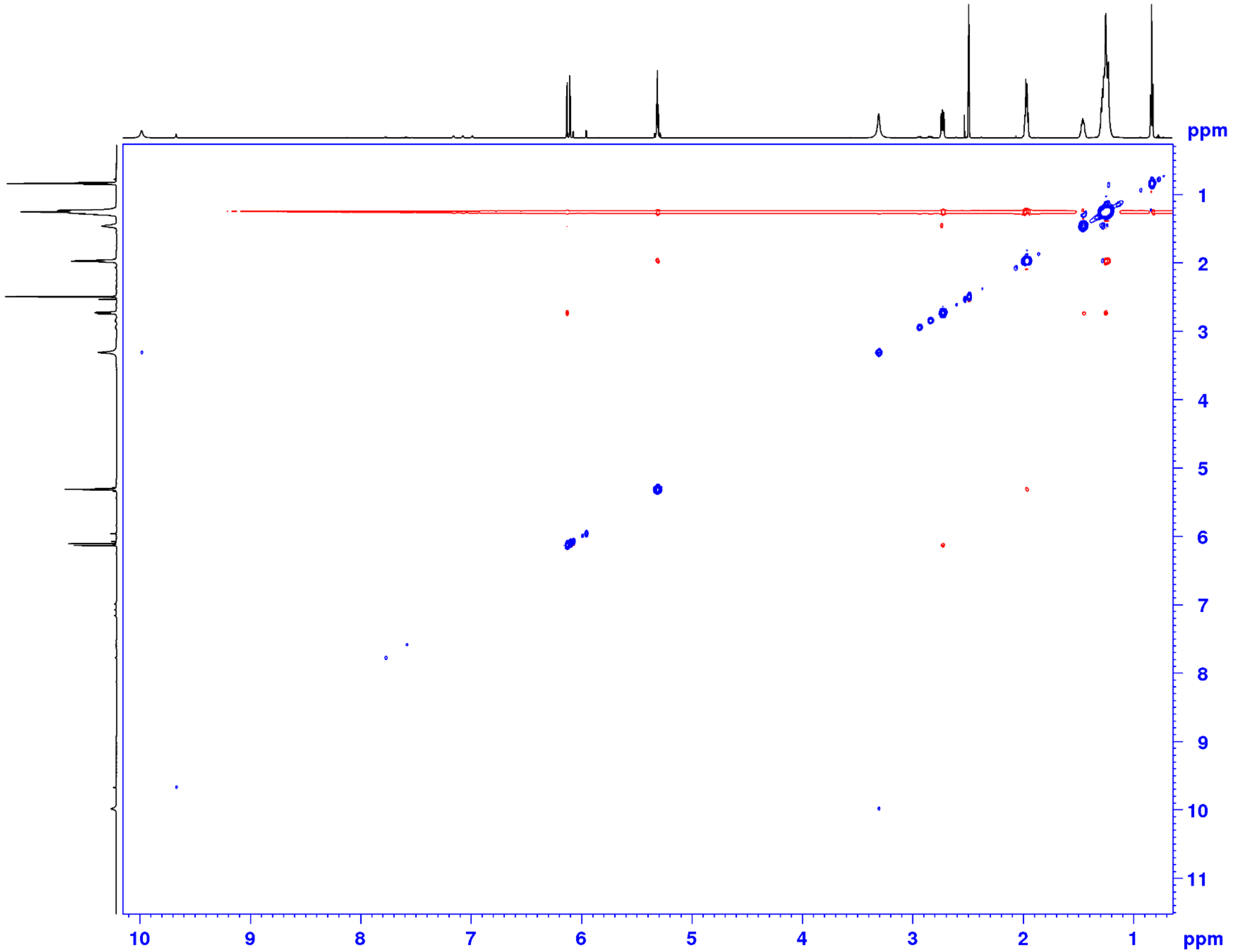

Figure S81. ROESY NMR spectrum (600 MHz) of capricostatin F (**4f**) in DMSO-d_6

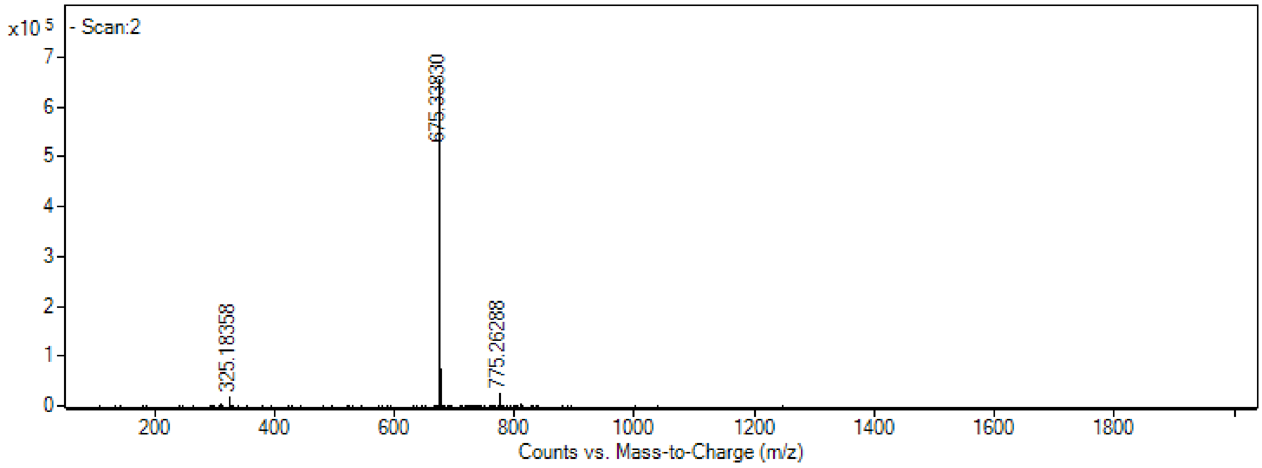

Figure S82. HRESI(−) mass spectrum of aquastatin A (**1a**)

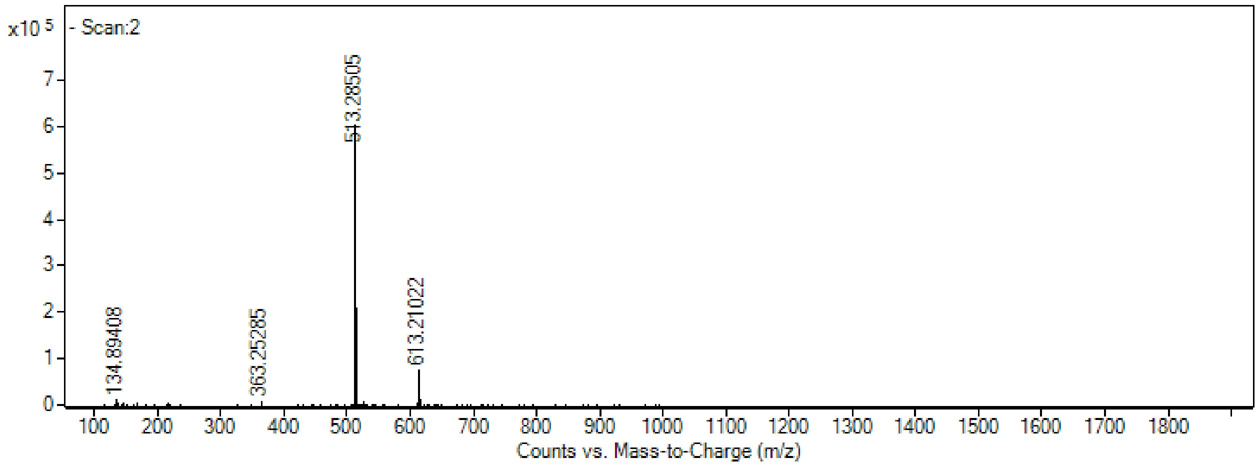

Figure S83. HRESI(−) mass spectrum of aquastatin B (**1b**)

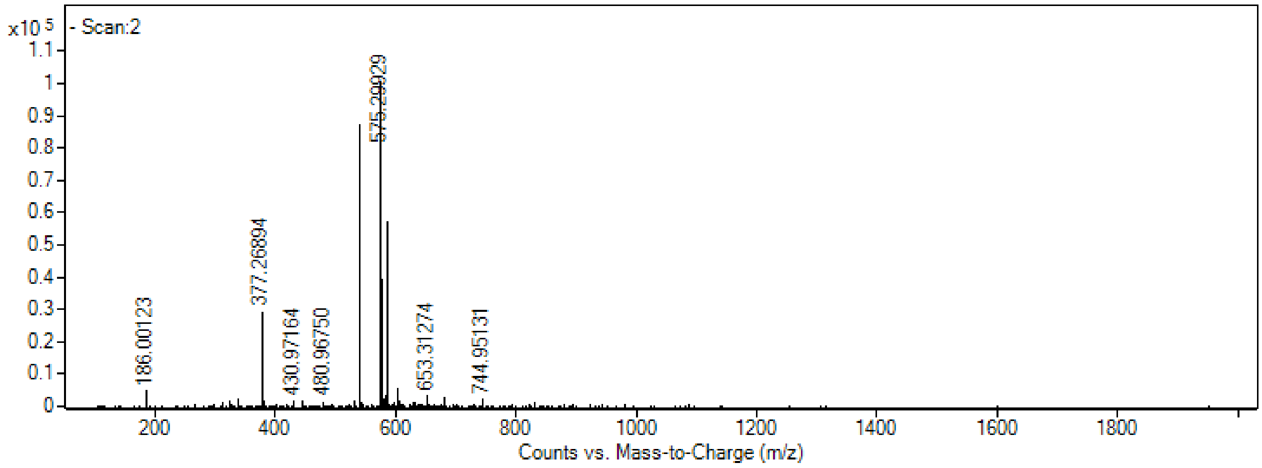

Figure S84. HRESI(−) mass spectrum of aquastatin C (**1c**)

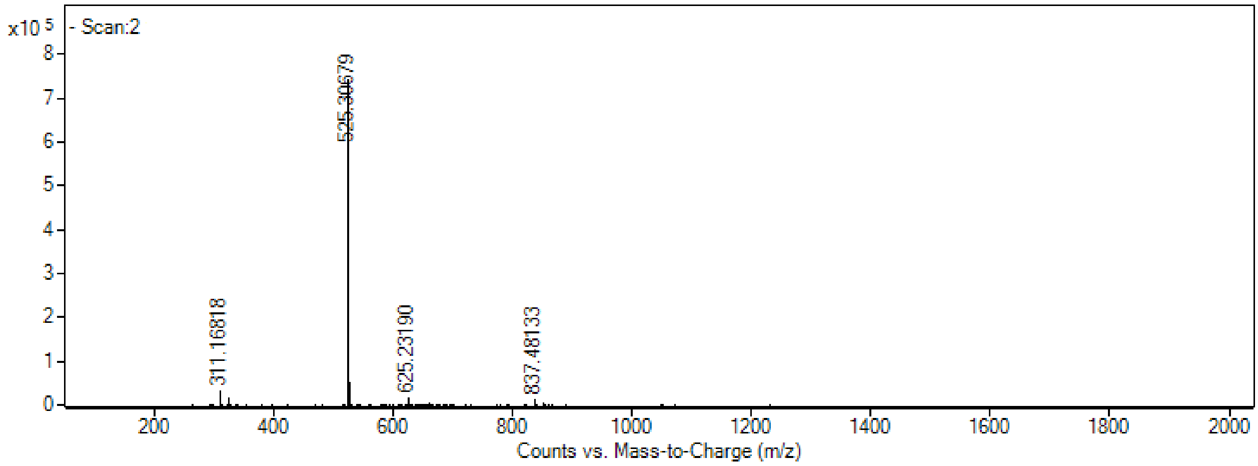

Figure S85. HRESI(−) mass spectrum of aquastatin D (**1d**)

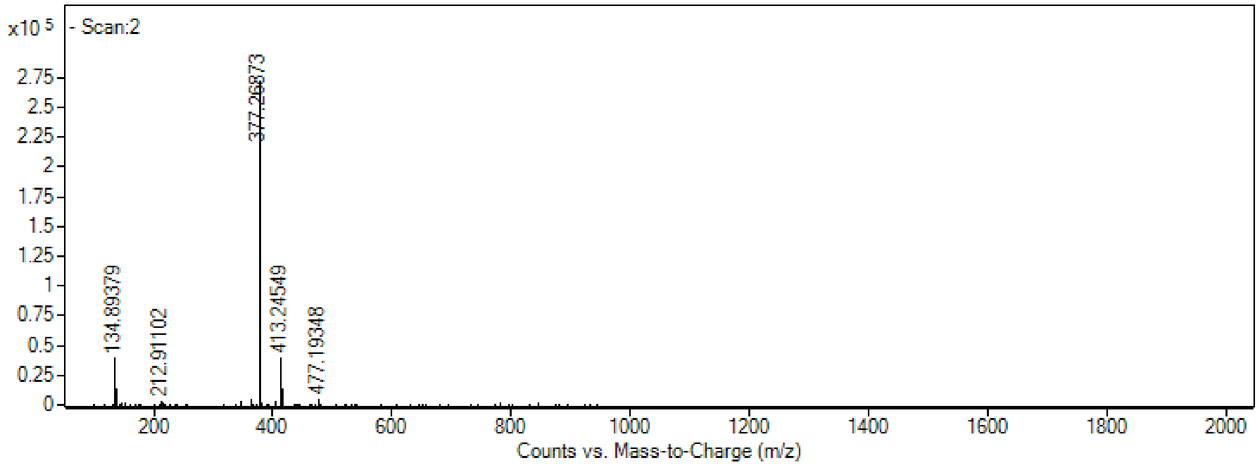

Figure S86. HRESI(−) mass spectrum of aquastatin E (**1e**)

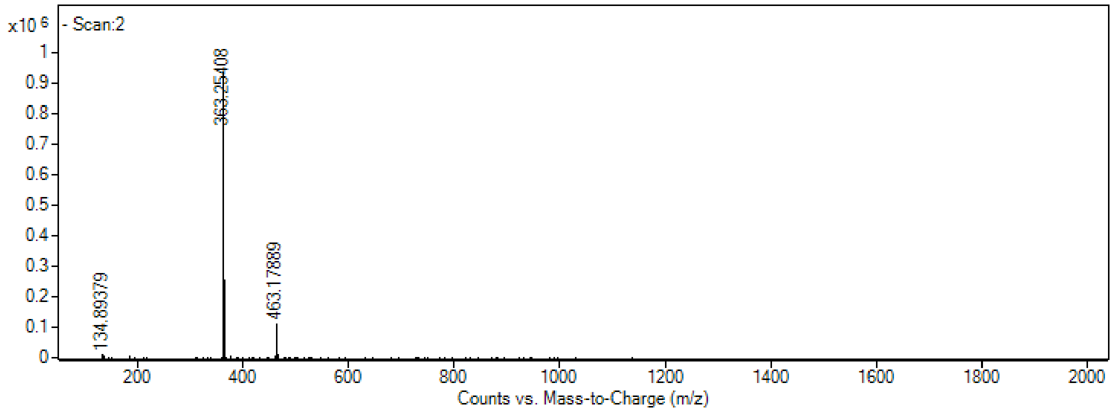

Figure S87. HRESI(−) mass spectrum of corticiolic acid (**1f**)

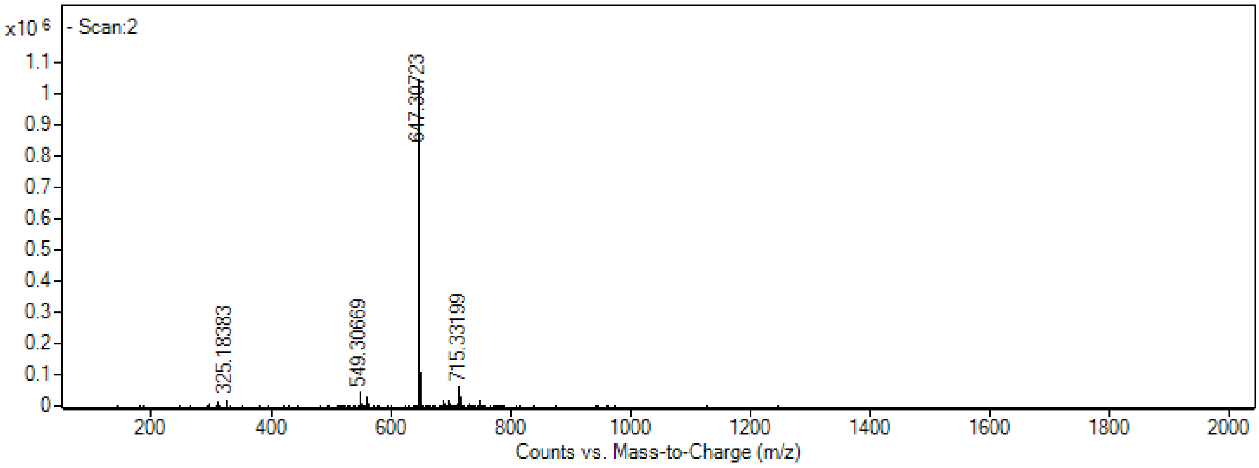

Figure S88. HRESI(−) mass spectrum of ariestatin A (**3a**)

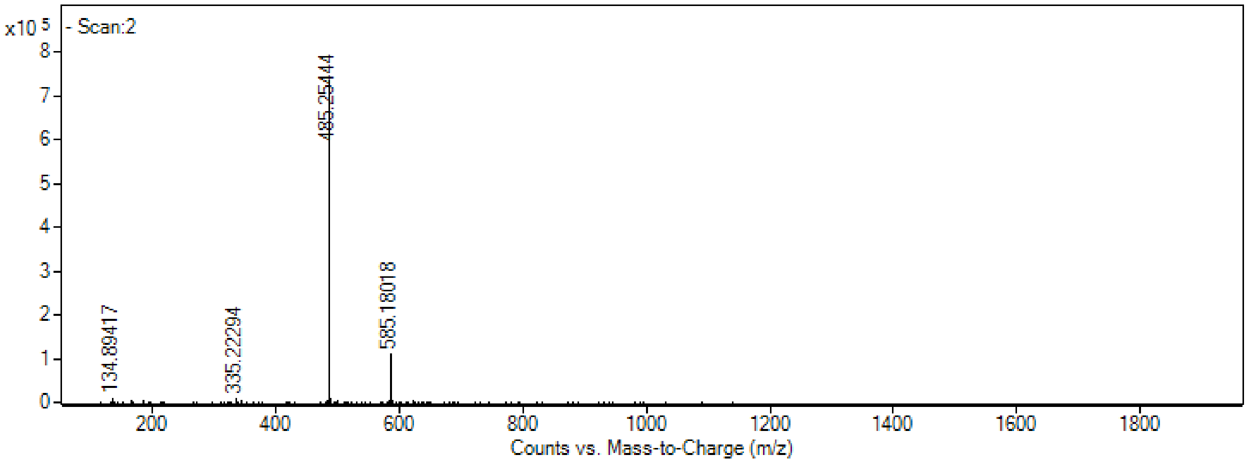

Figure S89. HRESI(−) mass spectrum of ariestatin B (**3b**)

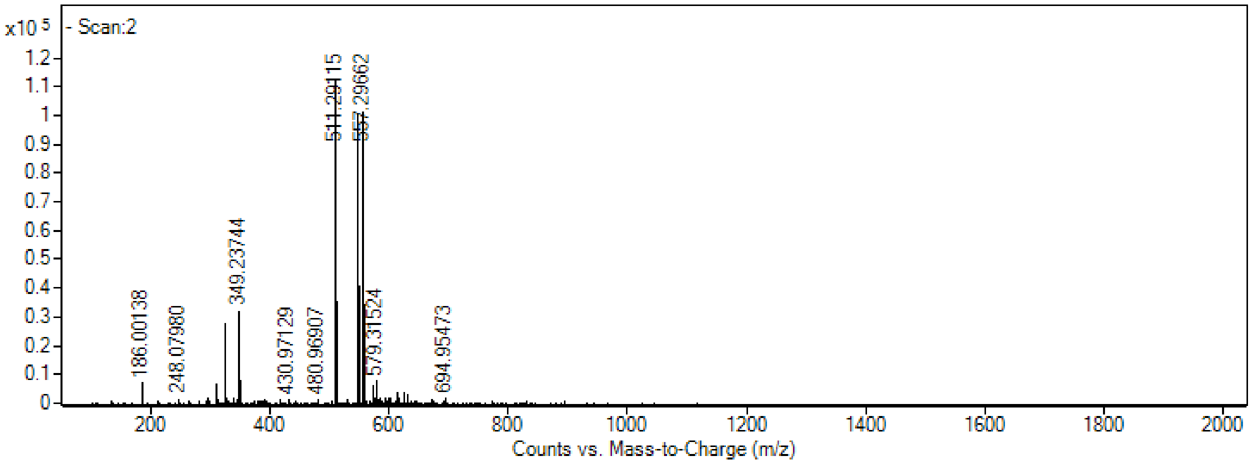

Figure S90. HRESI(−) mass spectrum of ariestatin C (**3c**)

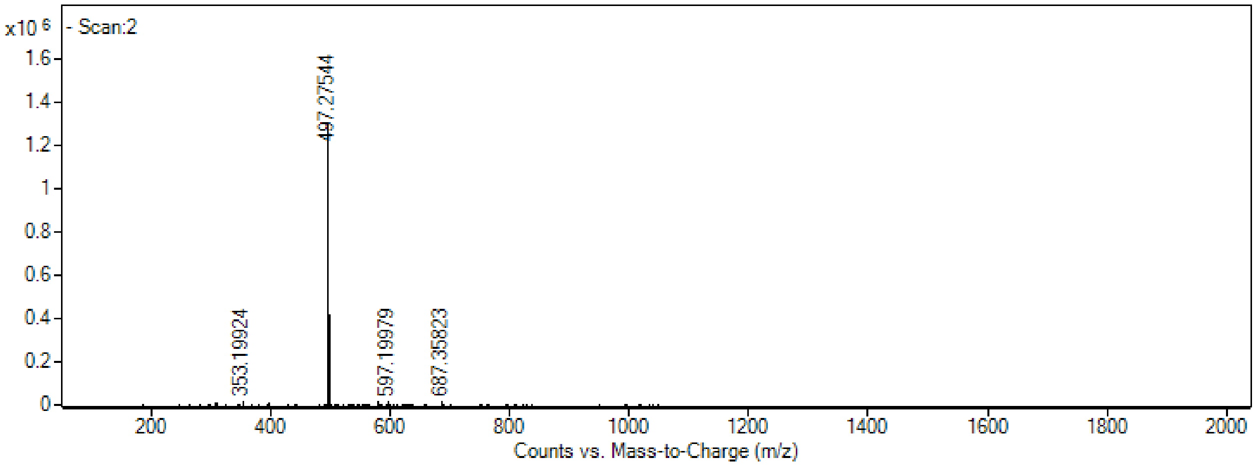

Figure S91. HRESI(−) mass spectrum of ariestatin D (**3d**)

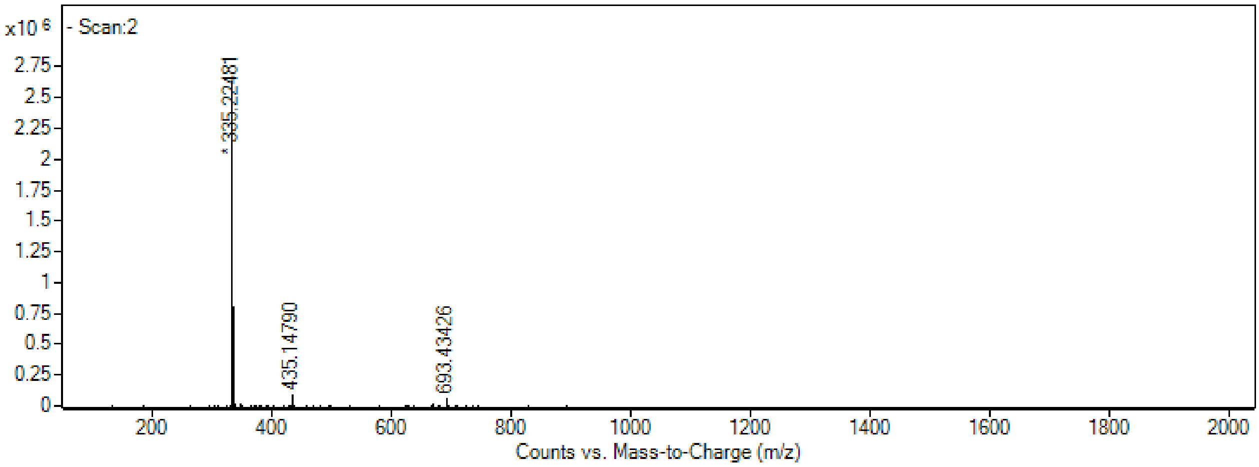

Figure S92. HRESI(−) mass spectrum of ariestatin F (**3f**)

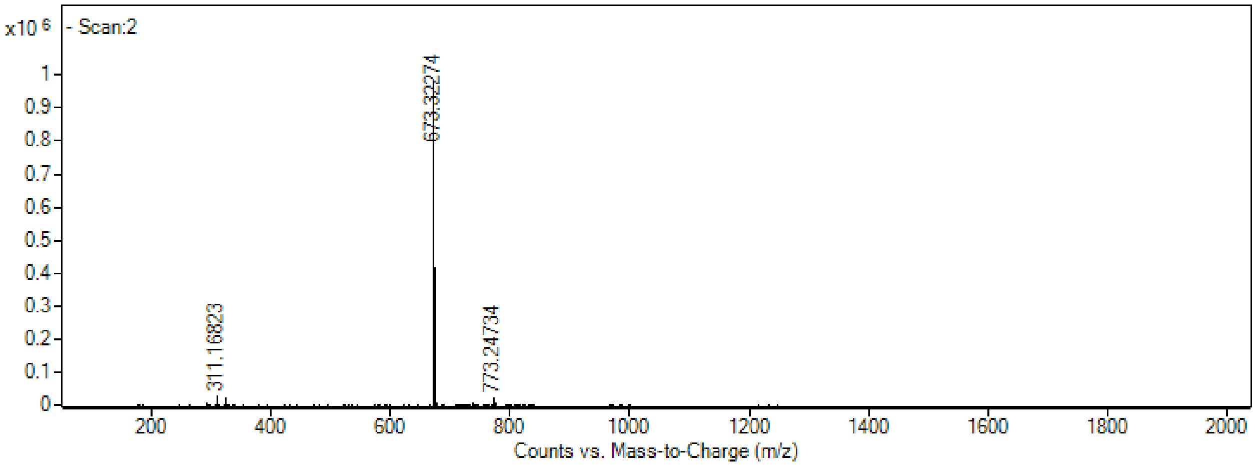

Figure S93. HRESI(−) mass spectrum of capricostatin A (**4a**)

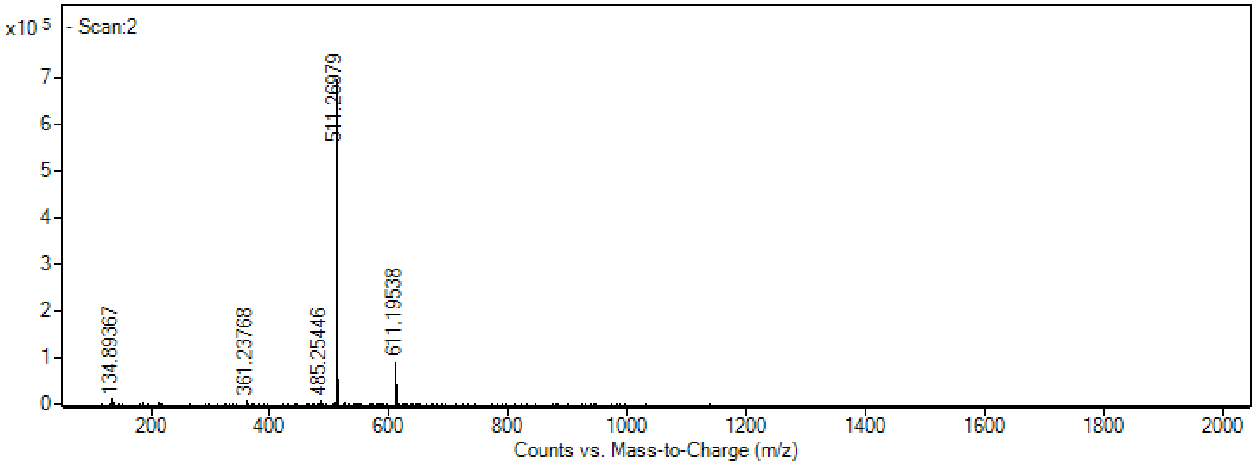

Figure S94. HRESI(−) mass spectrum of capricostatin B (**4b**)

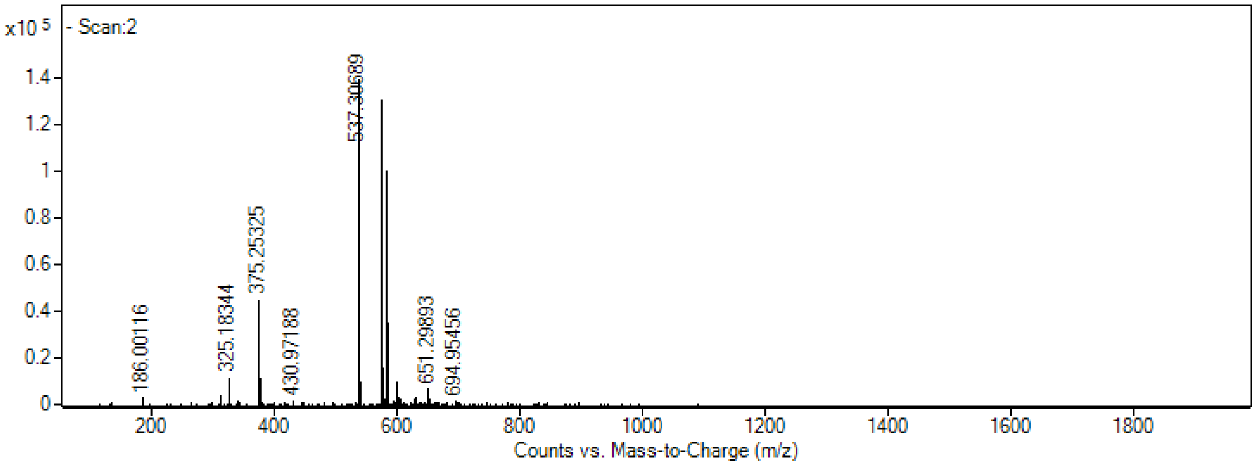

Figure S95. HRESI(−) mass spectrum of capricostatin C (**4c**)

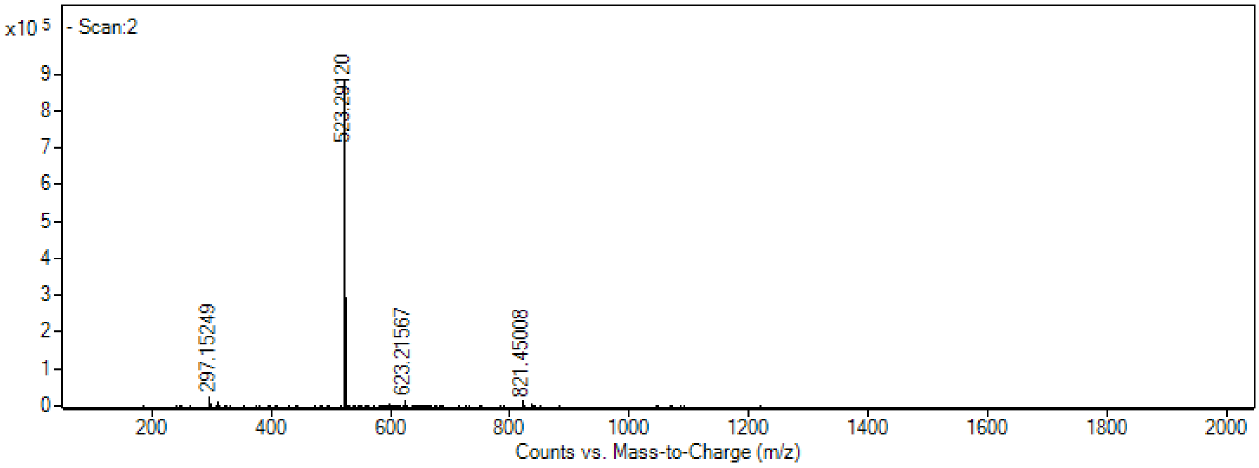

Figure S96. HRESI(−) mass spectrum of capricostatin D (**4d**)

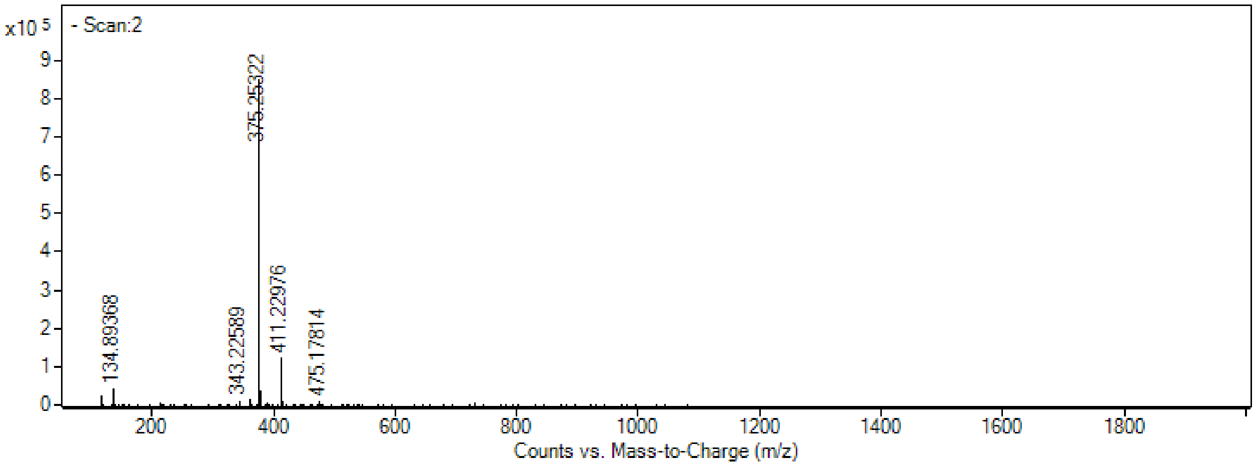

Figure S97. HRESI(−) mass spectrum of capricostatin E (**4e**)

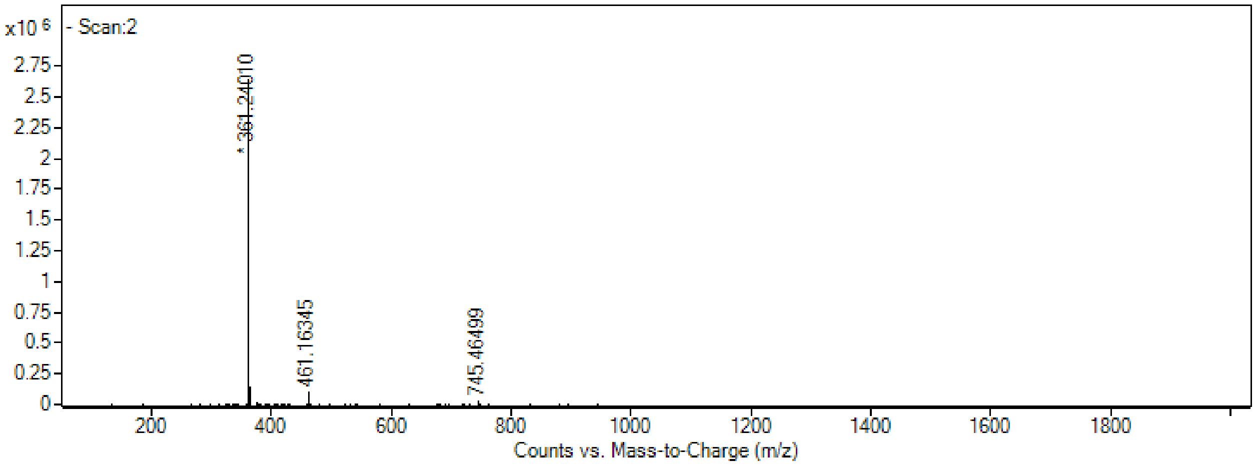

Figure S98. HRESI(−) mass spectrum of capricostatin F (**4f**)

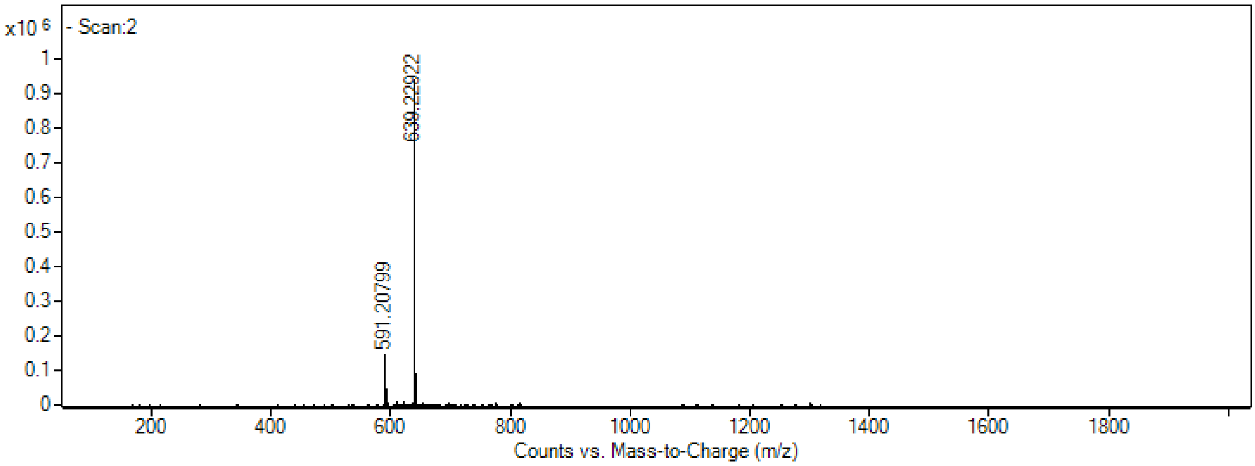

Figure S99. HRESI(−) mass spectrum of capricostatin A (**4a**) ozonolysis product

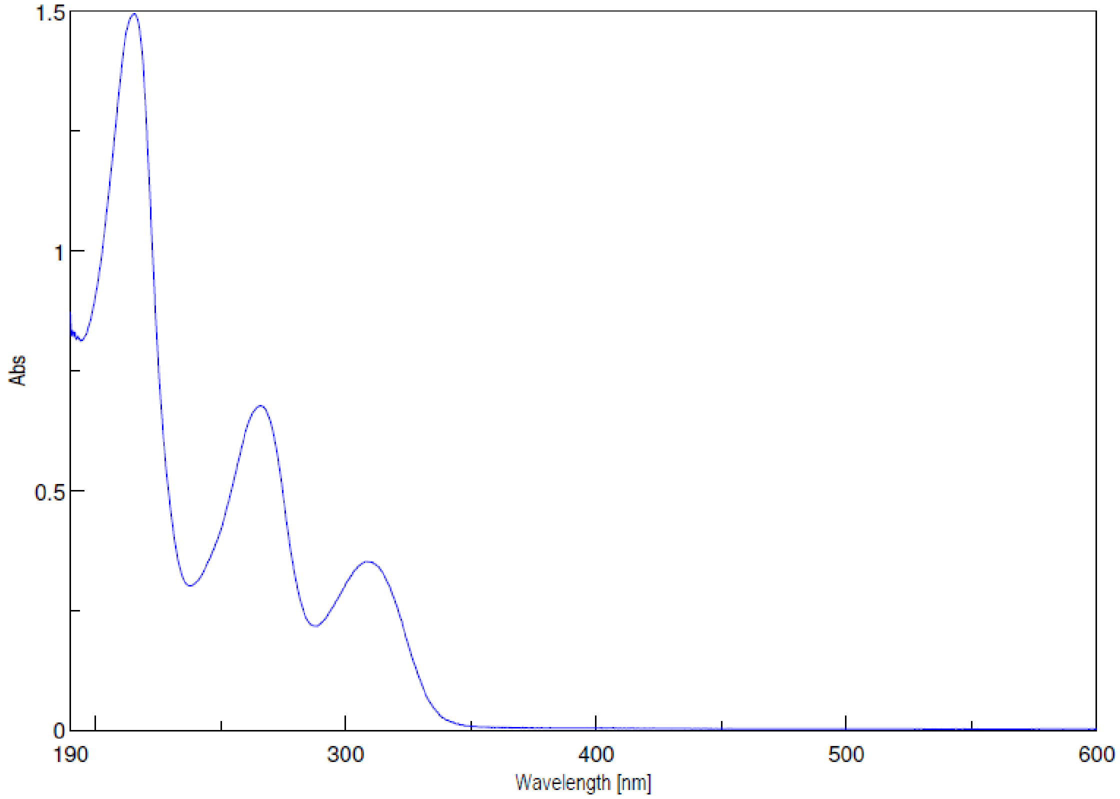

Figure S100. UV-vis spectrum of ariestatin A (**3a**) in MeCN.

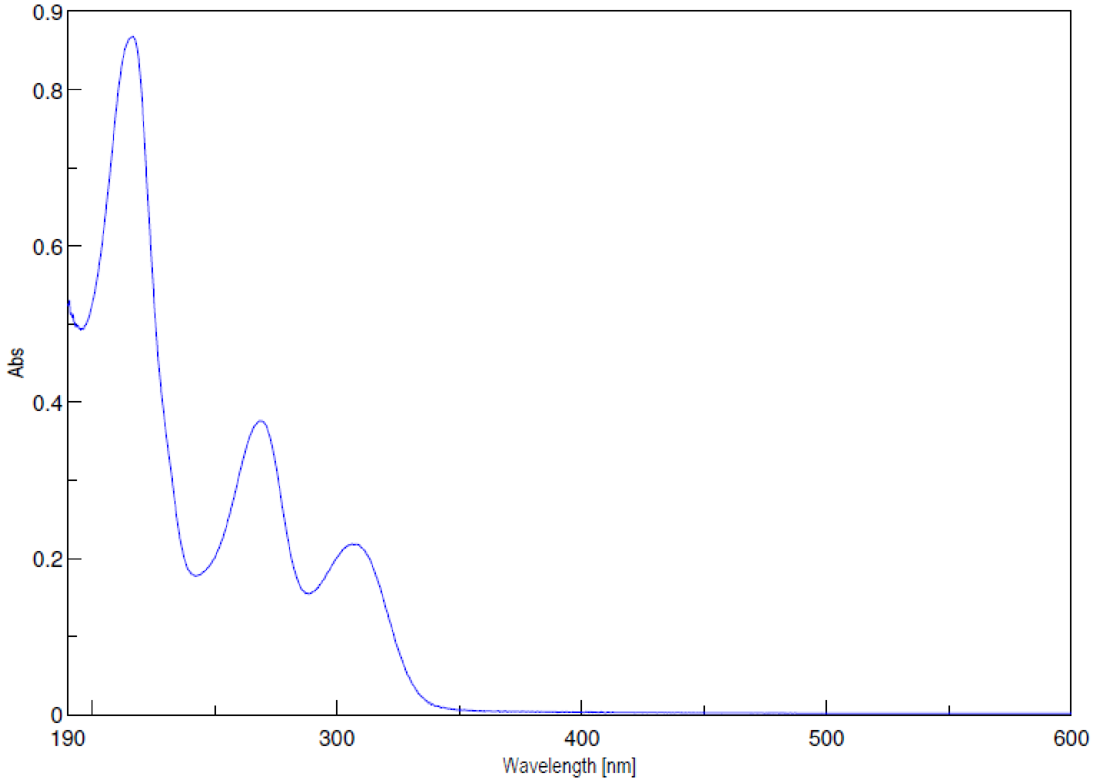

Figure S101. UV-vis spectrum of ariestatin B (**3b**) in MeCN.

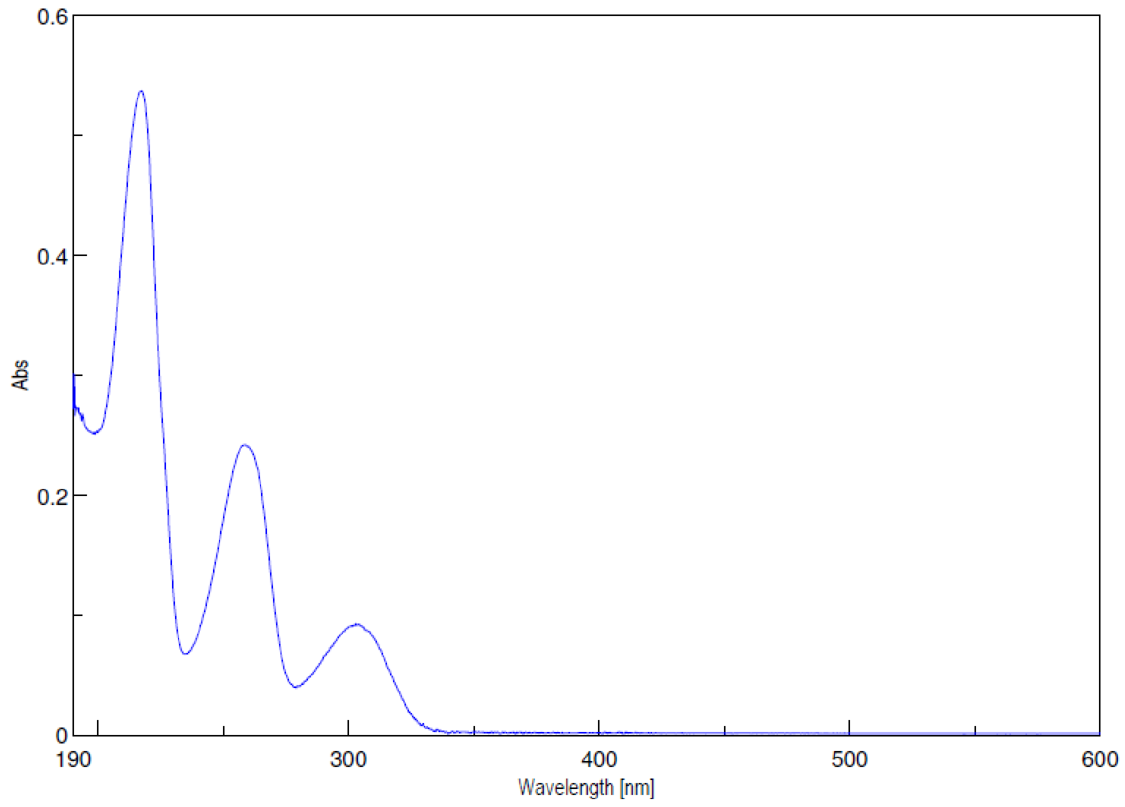

Figure S102. UV-vis spectrum of ariestatin C (**3c**) in MeCN.

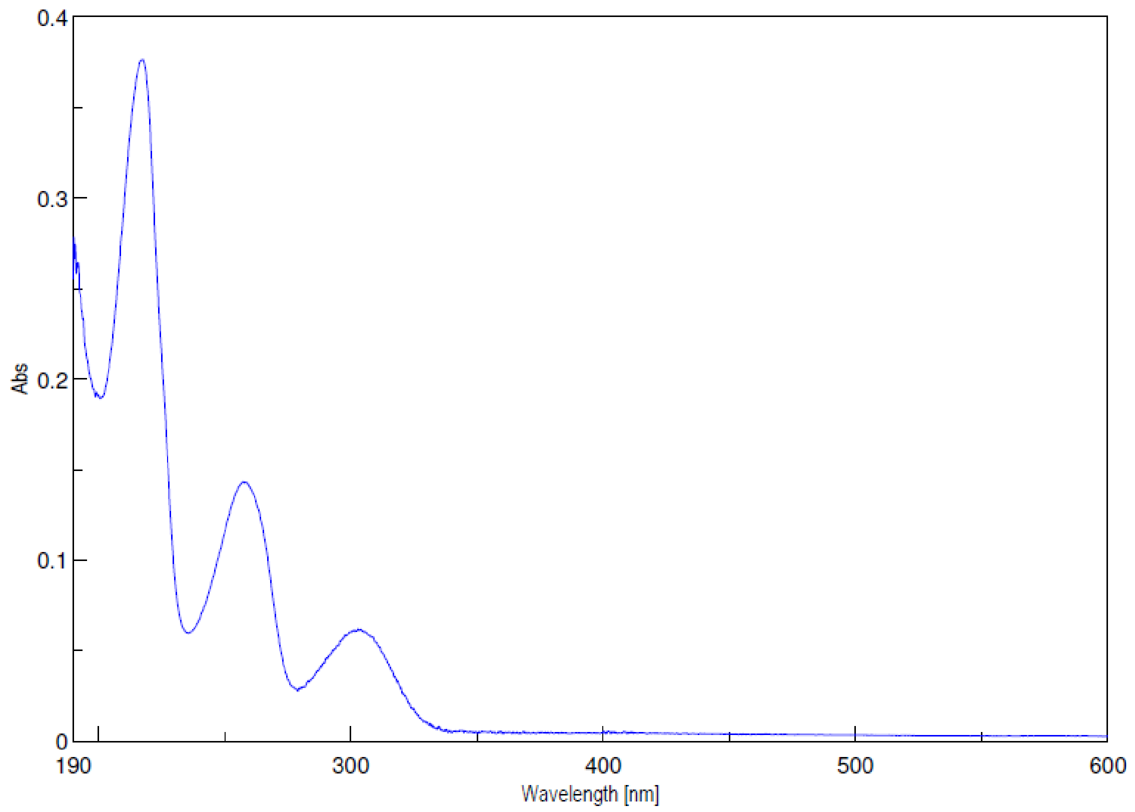

Figure S103. UV-vis spectrum of ariestatin D (**3d**) in MeCN.

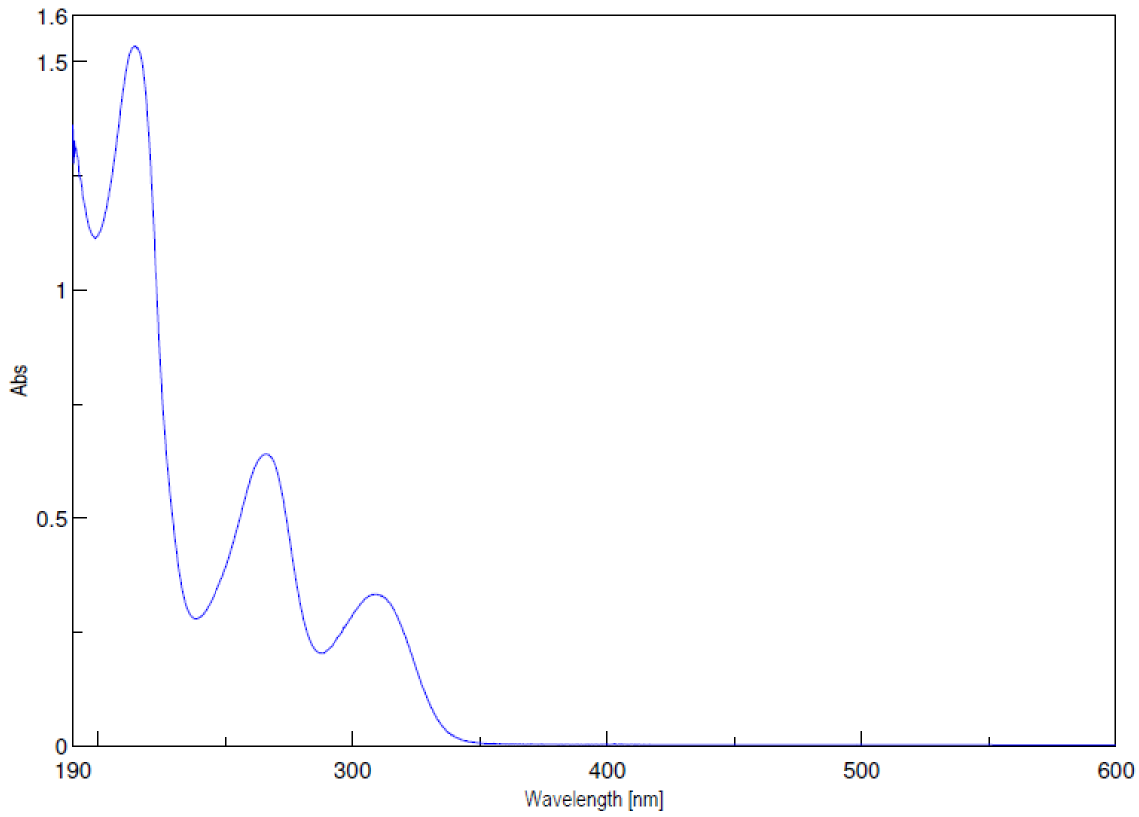

Figure S104. UV-vis spectrum of capricostatin A (**4a**) in MeCN.

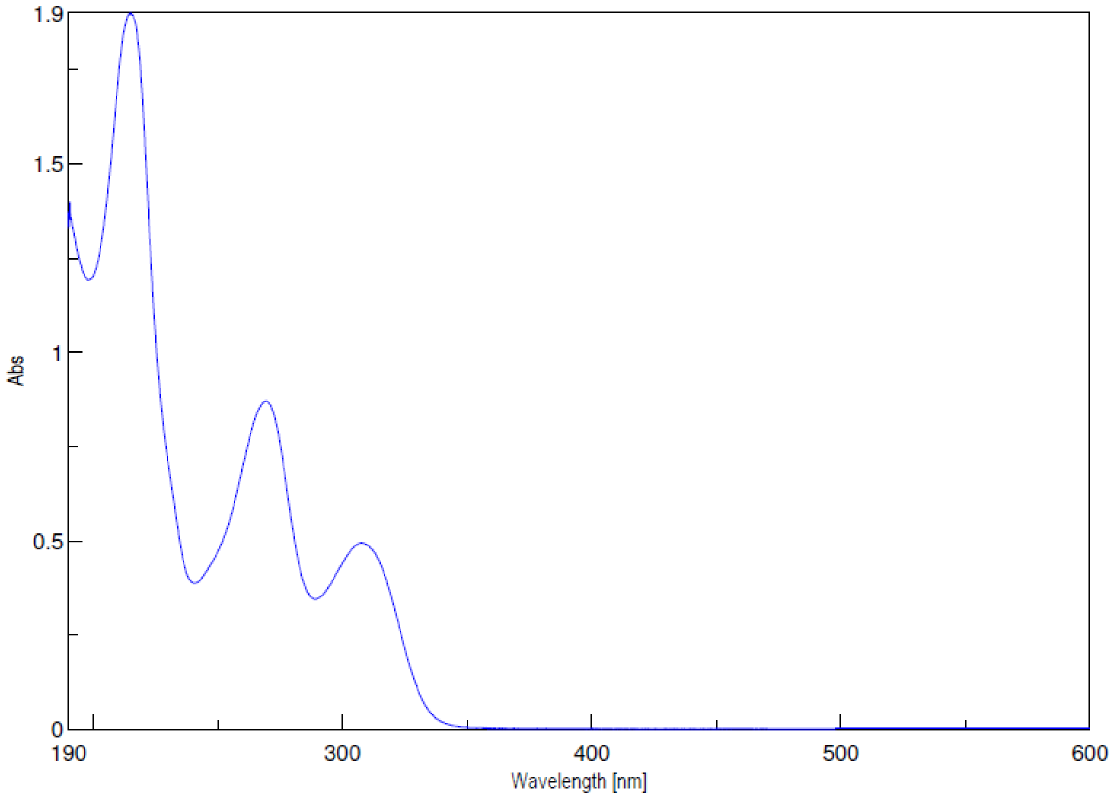

Figure S105. UV-vis spectrum of capricostatin B (**4b**) in MeCN.

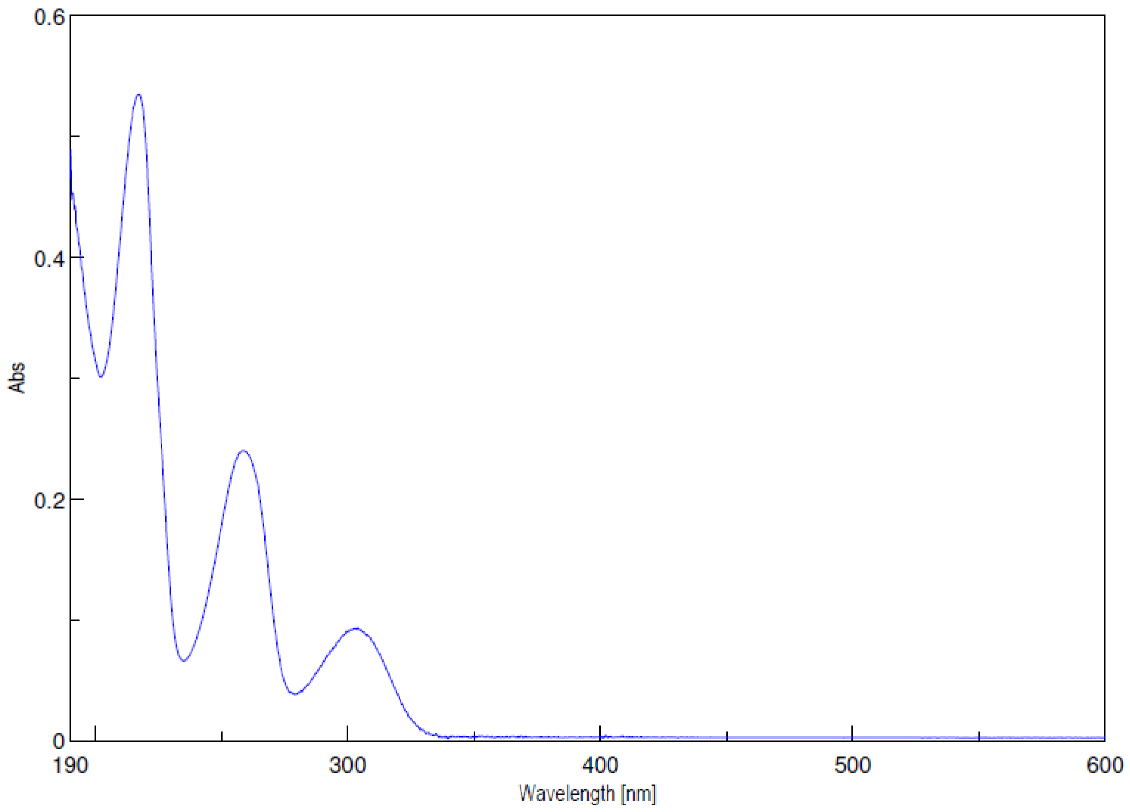

Figure S106. UV-vis spectrum of capricostatin C (**4c**) in MeCN.

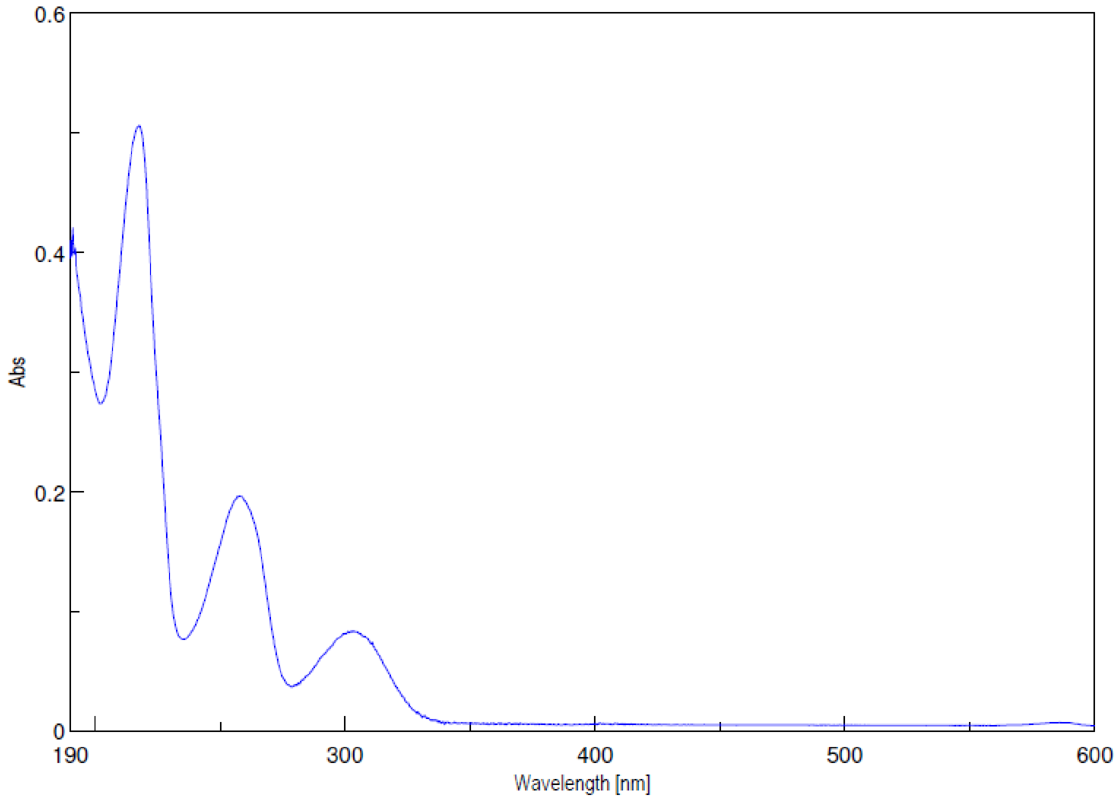

Figure S107. UV-vis spectrum of capricostatin D (**4d**) in MeCN.

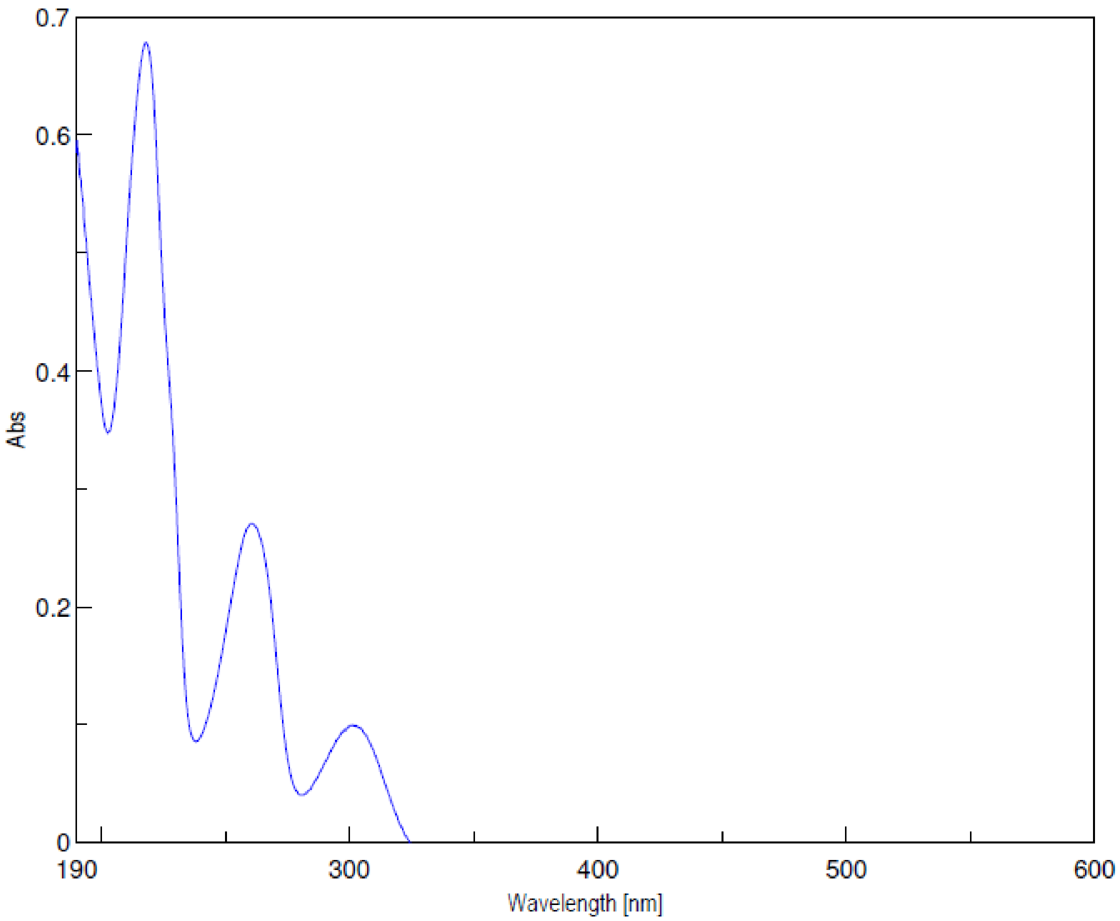

Figure S108. UV-vis spectrum of capricostatin F (**4f**) in MeCN.

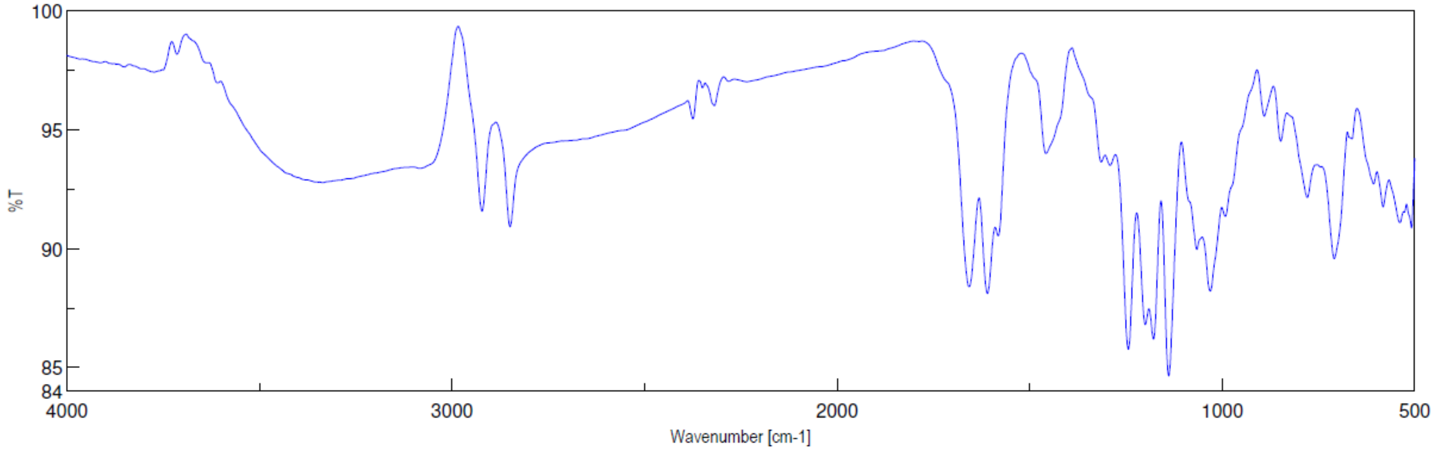

Figure S109. IR spectrum (ATR) of ariestatin A (**3a**).

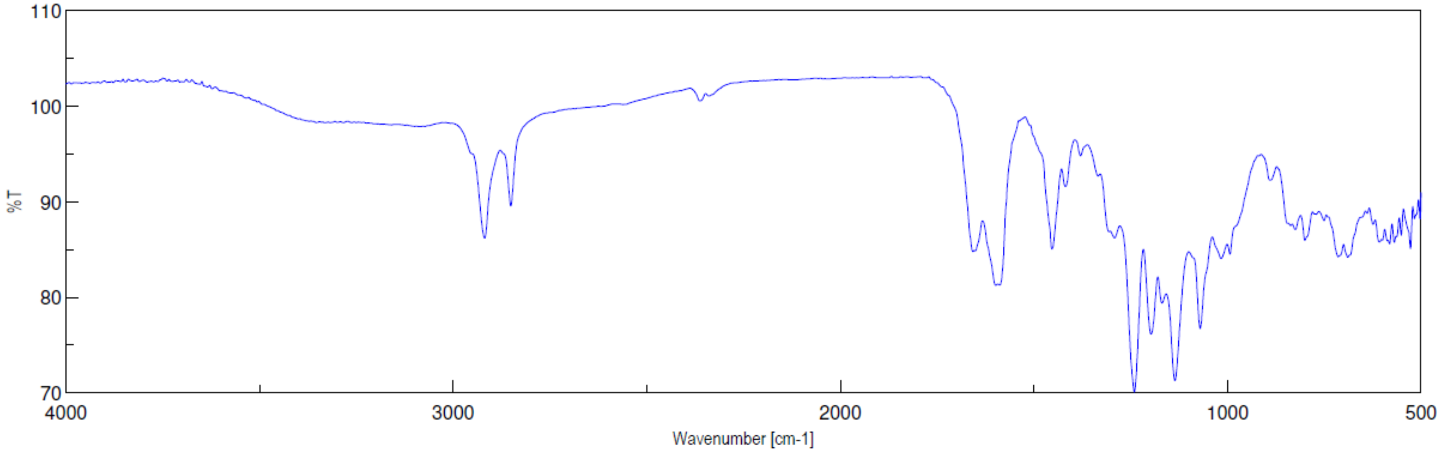

Figure S110. IR spectrum (ATR) of ariestatin B (**3b**).

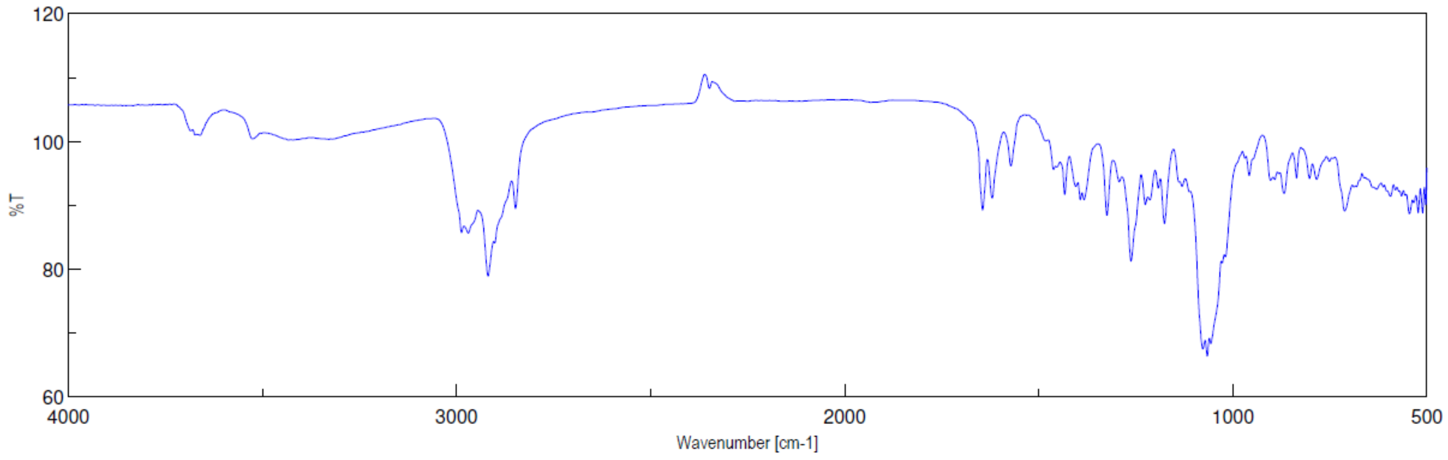

Figure S111. IR spectrum (ATR) of ariestatin C (**3c**).

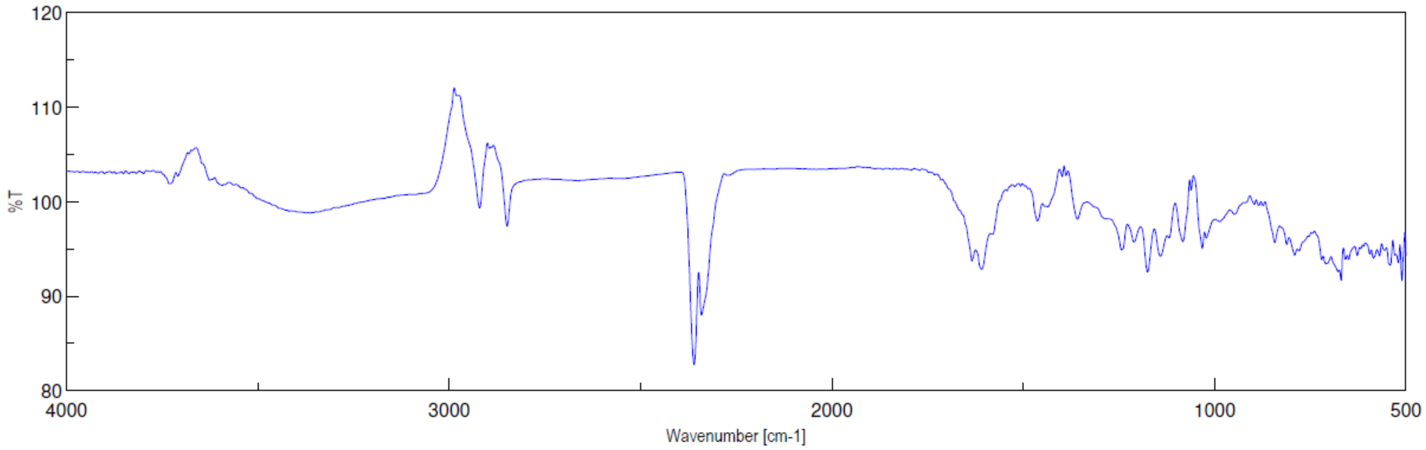

Figure S112. IR spectrum (ATR) of ariestatin D (**3d**).

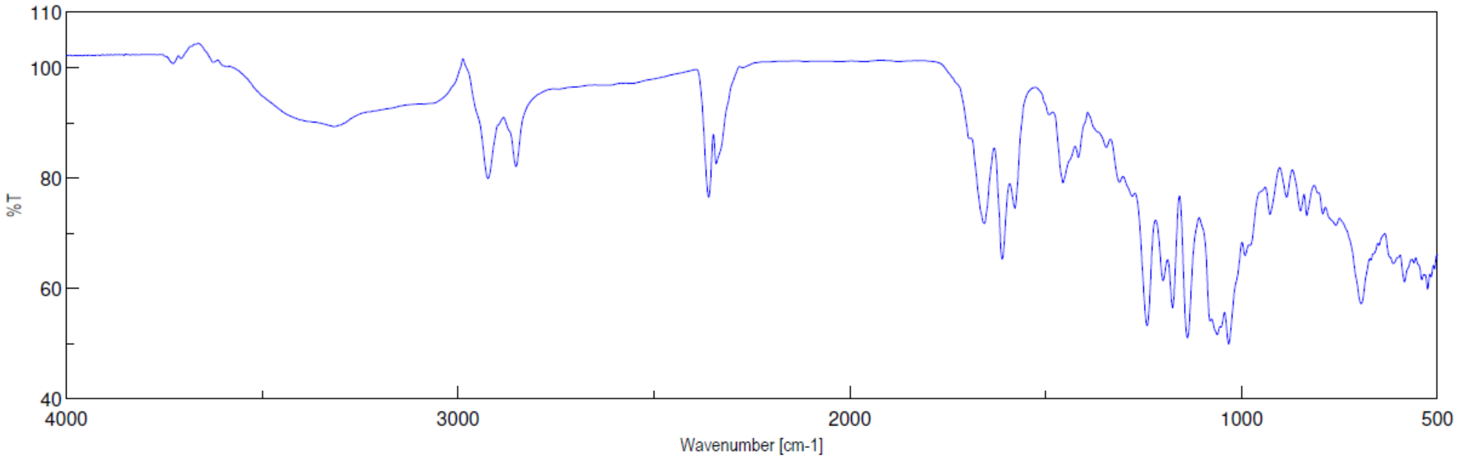

Figure S113. IR spectrum (ATR) of capricostatin A (**4a**).

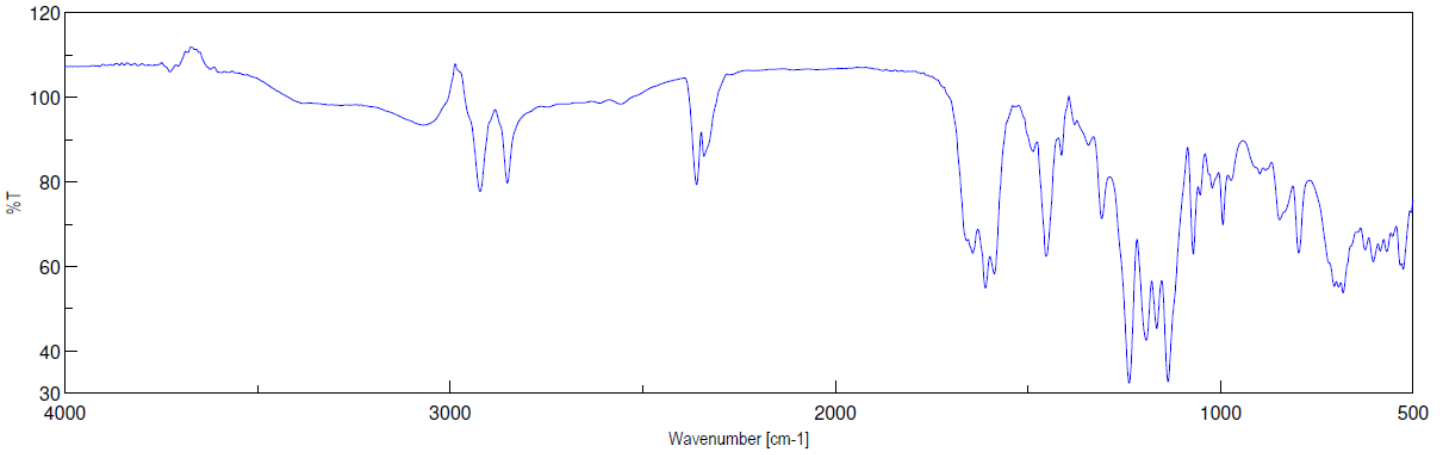

Figure S114. IR spectrum (ATR) of capricostatin B (**4b**).

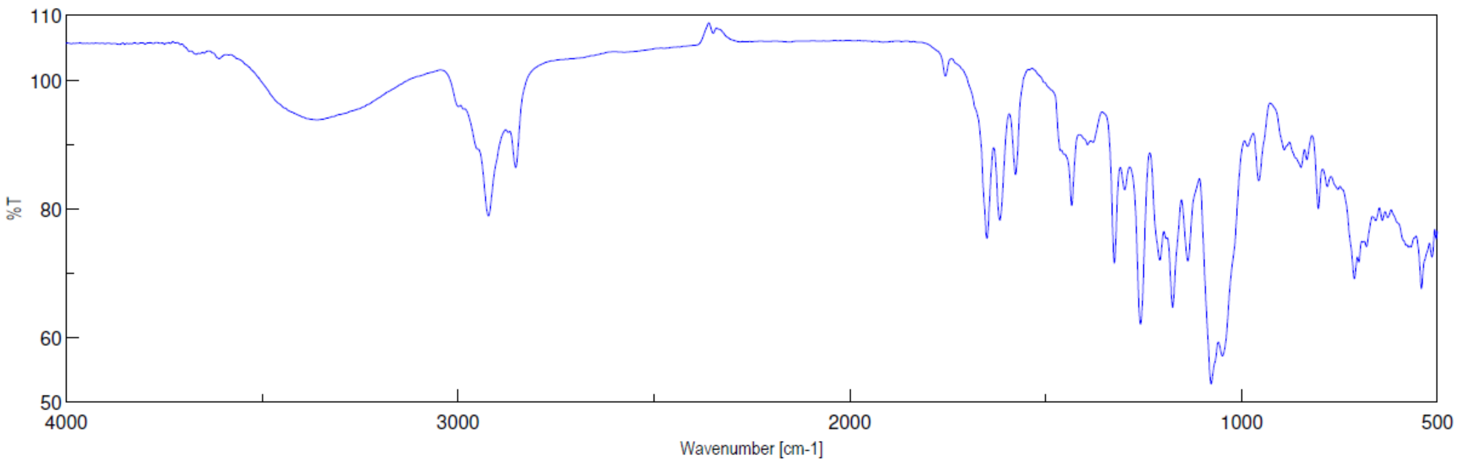

Figure S115. IR spectrum (ATR) of capricostatin C (**4c**).

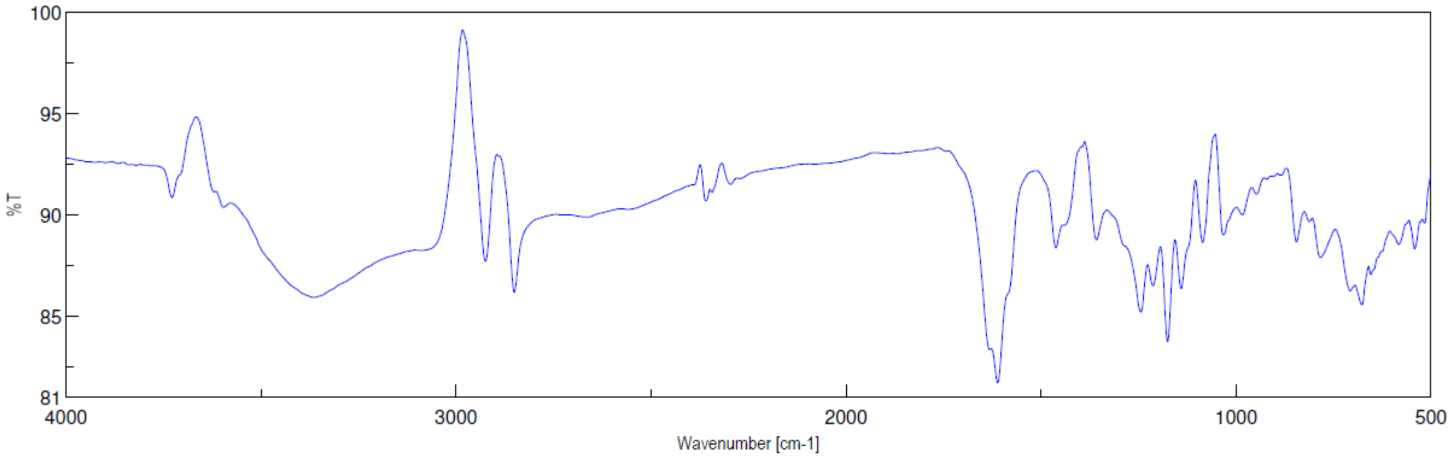

Figure S116. IR spectrum (ATR) of capricostatin D (**4d**).

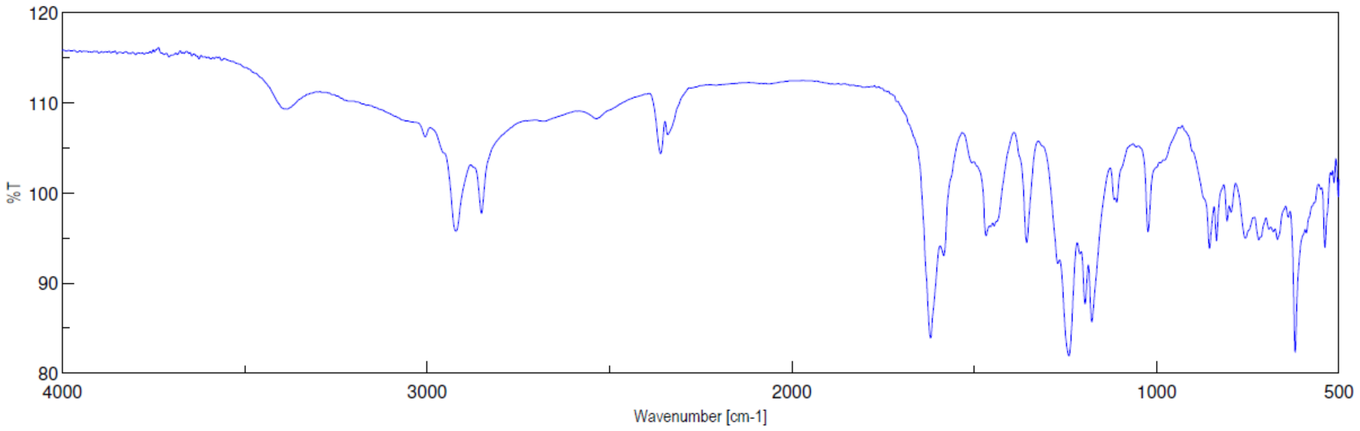

Figure S117. IR spectrum (ATR) of capricostatin F (**4f**).

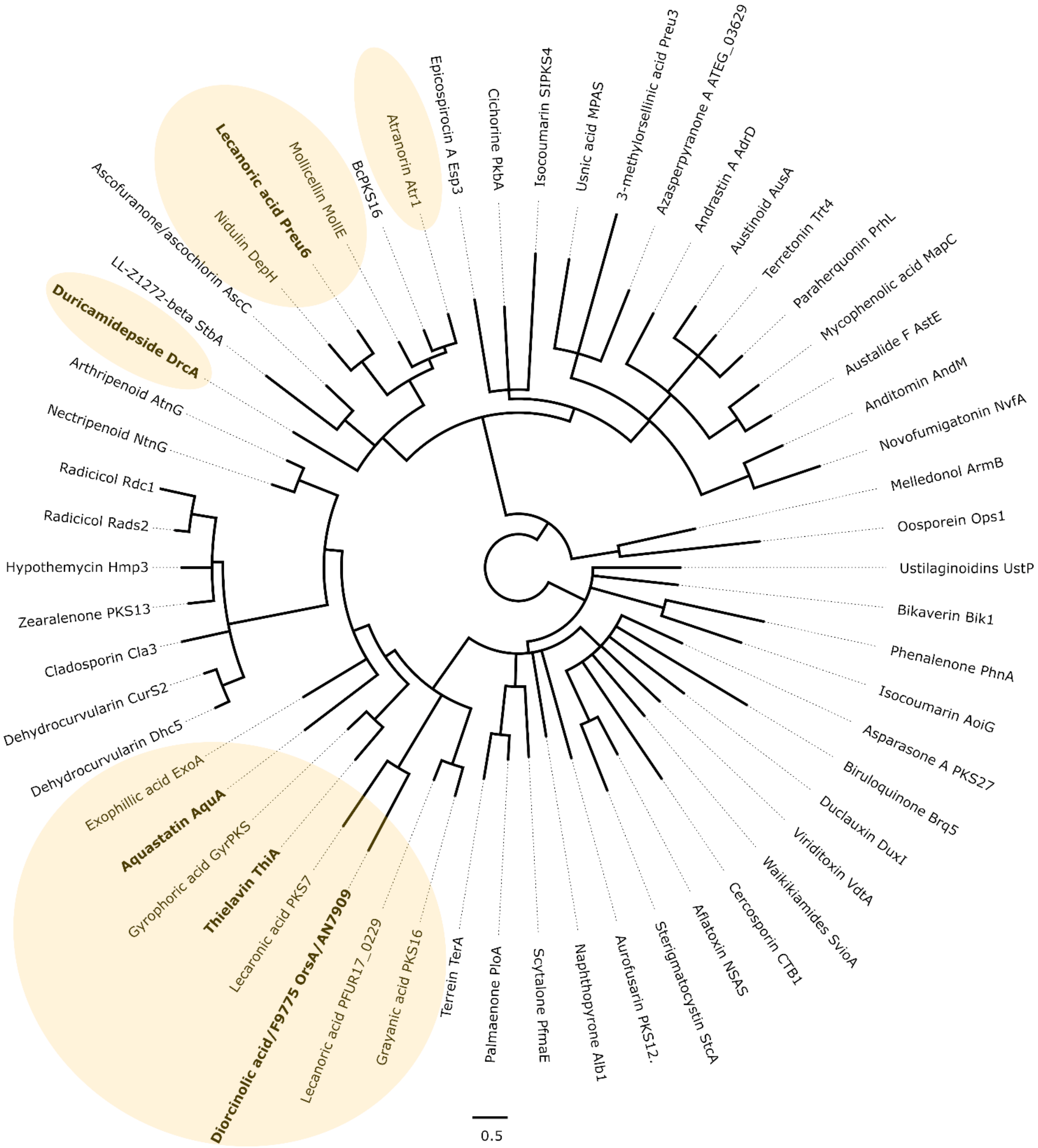

Figure S118. Phylogenetic analysis of SAT domain sequences of selected fungal NR-PKSs.

Depside-forming enzymes are highlighted in yellow. AquA and enzymes with characterised SAT and TE domains are displayed in bold. Branches with aLRT SH-like support < 0.8 have been collapsed.

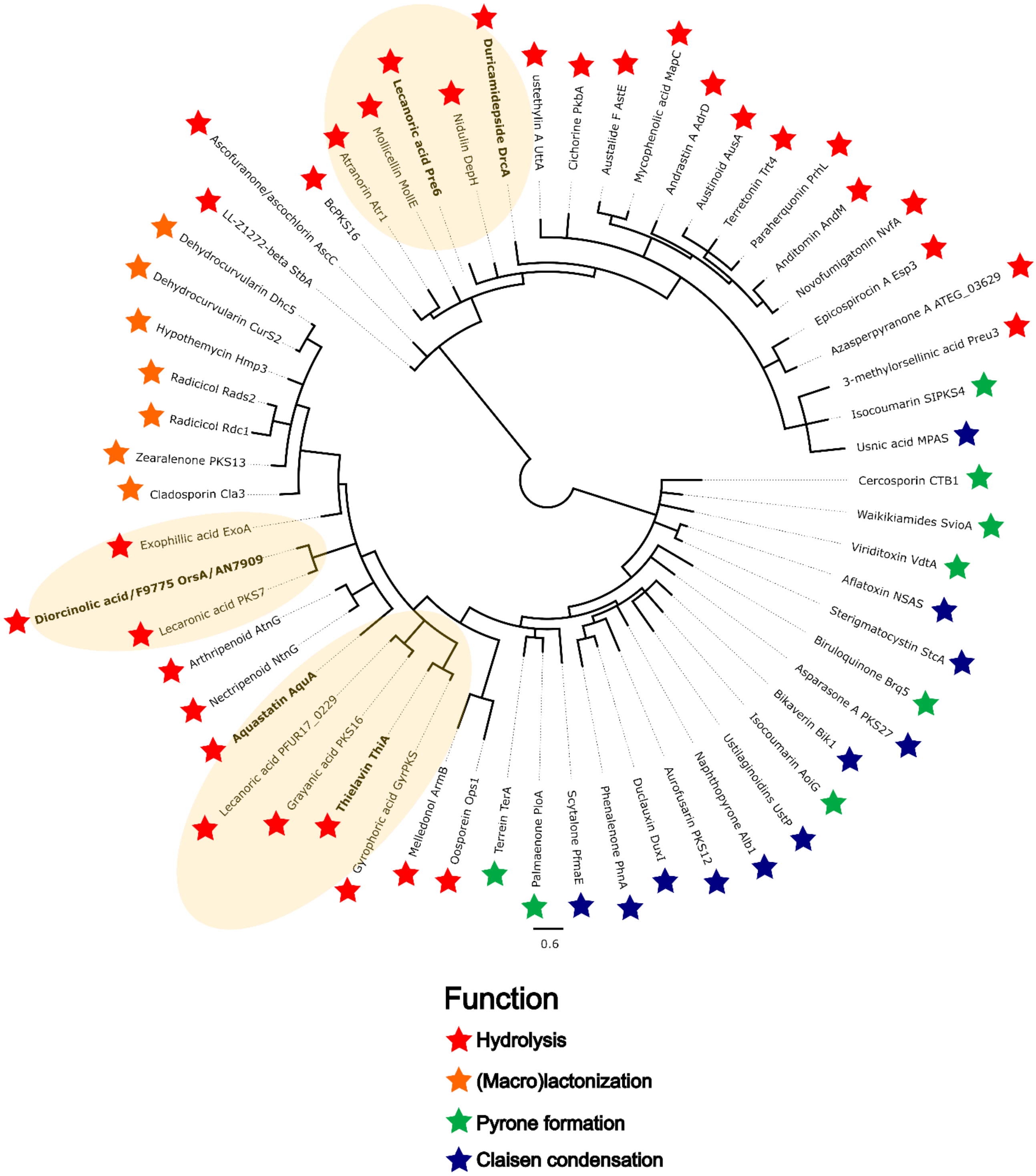

Figure S119. Phylogenetic analysis of TE domain sequences of selected fungal NR-PKSs.

Depside-forming enzymes are highlighted in yellow. AquA and enzymes with characterised SAT and TE domains are displayed in bold. Branches with aLRT SH-like support < 0.8 have been collapsed.

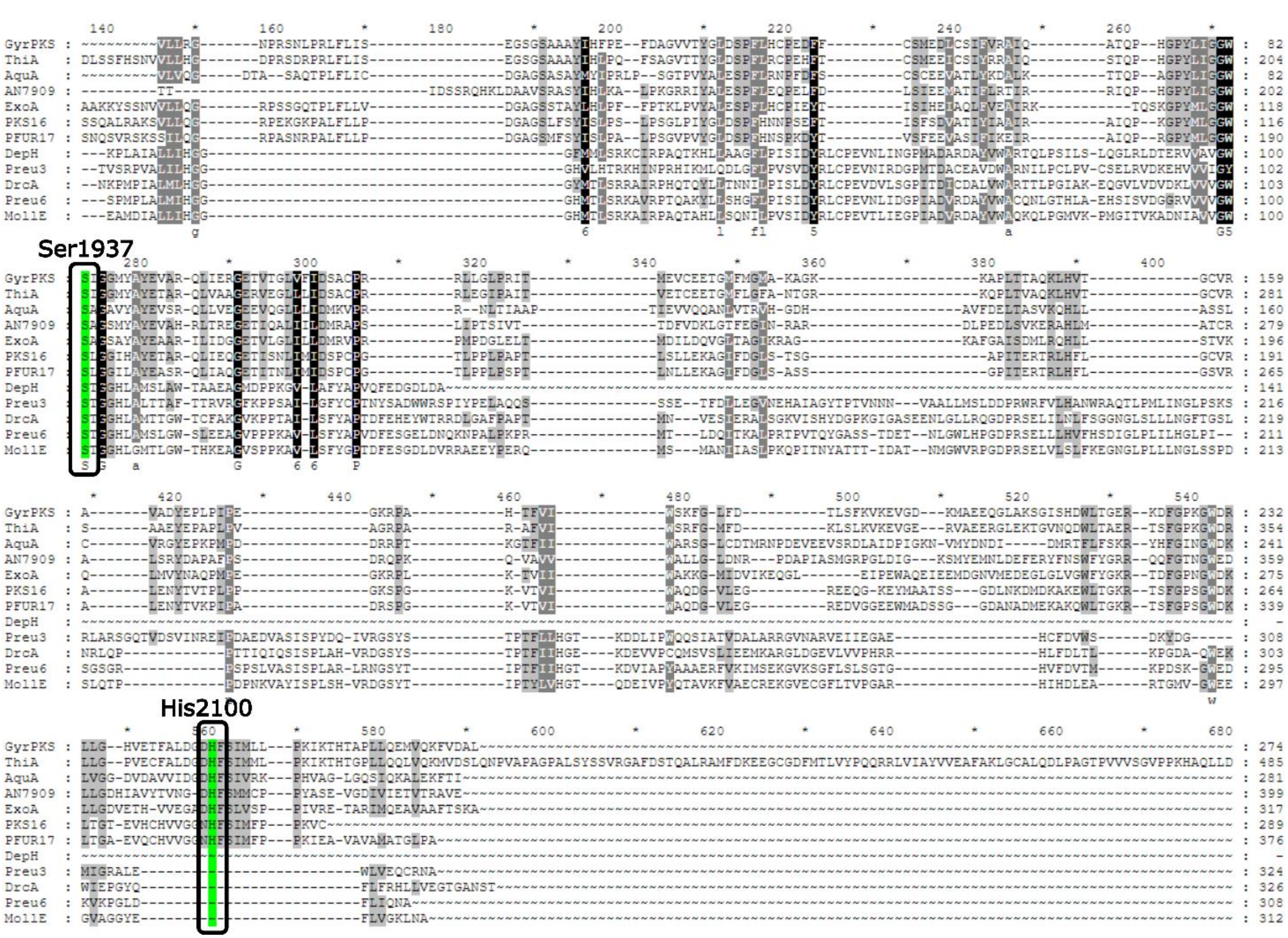

Figure S120. Sequence alignment of TE domain sequences of depside-forming enzymes.

Ser1937 and His2100 of ThiA are highlighted in green.

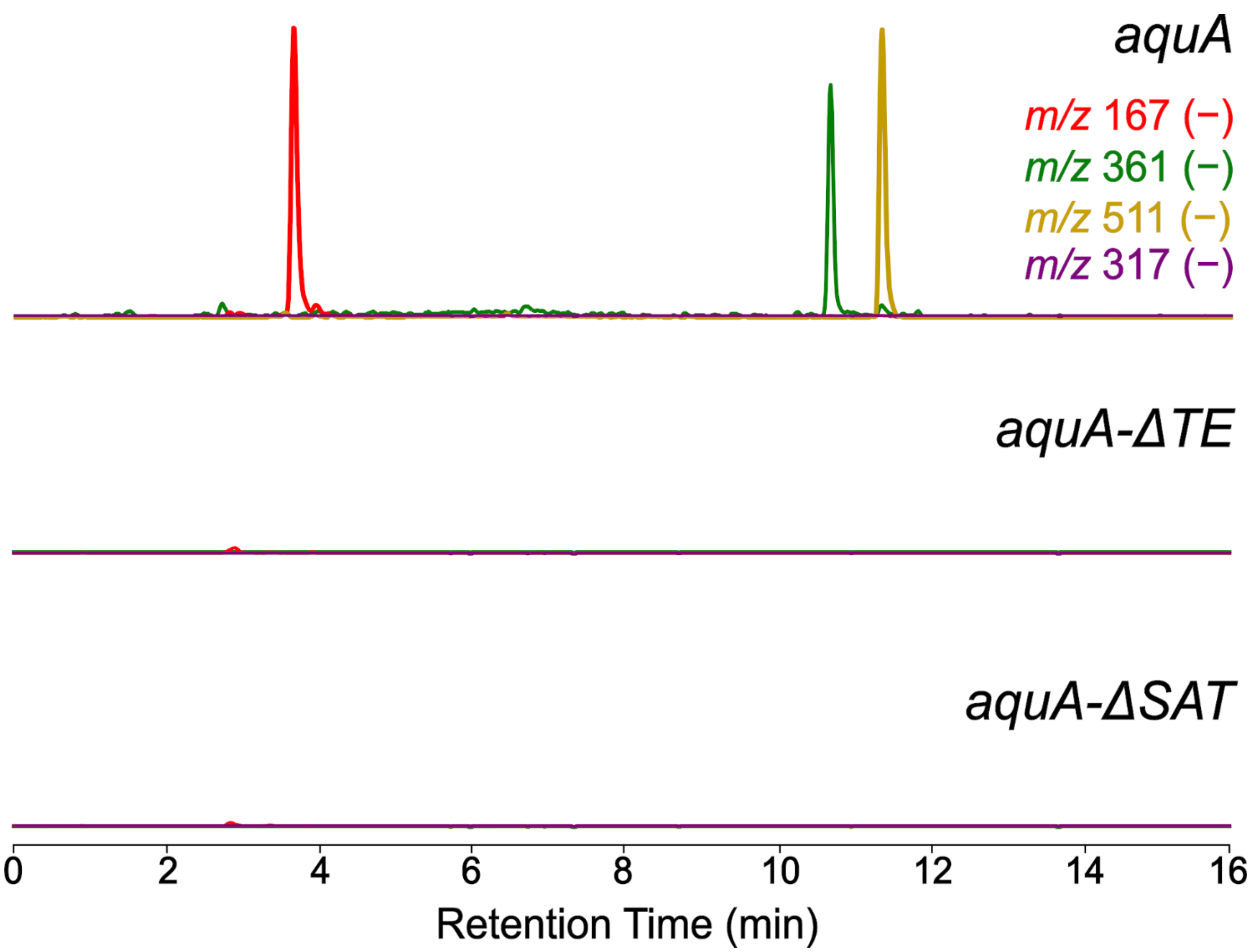

Figure S121. Heterologous expression of truncated versions of *aquA* (Δ*TE* and Δ*SAT*) in *S. cerevisiae*.

EIC(−)MS of **1b** (*m/z* 511), **1f** (*m/z* 361), **5** (*m/z* 167), and lecanoric acid (*m/z* 317).

References

1 R. D. Gietz and R. H. Schiestl, *Nat Protoc*, 2007, **2**, 31.

2 Y.-M. Chiang, C. E. Oakley, M. Ahuja, R. Entwistle, A. Schultz, S.-L. Chang, C. T. Sung, C. C. C. Wang and B. R. Oakley, *J Am Chem Soc*, 2013, **135**, 7720–7731.

3 I. Roux and Y. H. Chooi, in *Engineering Natural Product Biosynthesis: Methods and Protocols*, ed. E. Skellam, Springer US, New York, NY, 2022, pp. 75–92.

4 N. Sbaraini, J. Hu, I. Roux, C.-S. Phan, H. Motta, H. Rezaee, A. Schrank, Y.-H. Chooi and C. Christian Staats, *Fungal Genet. Biol.*, 2021, 103568.

5 J. Stajich and J. Palmer, 2018.

6 A. Bankevich, S. Nurk, D. Antipov, A. A. Gurevich, M. Dvorkin, A. S. Kulikov, V. M. Lesin, S. I. Nikolenko, S. Pham, A. D. Prjibelski, A. V Pyshkin, A. V Sirotkin, N. Vyahhi, G. Tesler, M. A. Alekseyev and P. A. Pevzner, *J Comput Biol*, 2012, **19**, 455–477.

7 N. Pierce, L. Irber, T. Reiter, P. Brooks and C. T. Brown, *Large-scale sequence comparisons with sourmash*, 2019.

8 H. Li and R. Durbin, *Bioinformatics*, 2009, **25**, 1754–1760.

9 H. Li, B. Handsaker, A. Wysoker, T. Fennell, J. Ruan, N. Homer, G. Marth, G. Abecasis, R. Durbin and 1000 Genome Project Data Processing Subgroup, *Bioinformatics*, 2009, **25**, 2078–2079.

10 H. Li, *Bioinformatics*, 2018, **34**, 3094–3100.

11 J. Palmer and J. Stajich, 2019.

12 M. Green and J. Sambrook, *Molecular Cloning: A Laboratory Manual*, Cold Spring Harbour, Spring Harbor Laboratory Press, New York, 4th Edition, 2012.

13 K. Blin, S. Shaw, H. E. Augustijn, Z. L. Reitz, F. Biermann, M. Alanjary, A. Fetter, B. R. Terlouw, W. W. Metcalf, E. J. N. Helfrich, G. P. van Wezel, M. H. Medema and T. Weber, *Nucleic Acids Res*, 2023, **51**, W46–W50.

14 C. L. M. Gilchrist, T. J. Booth, B. van Wersch, L. van Grieken, M. H. Medema and Y.-H. Chooi, *Bioinform. Adv.*, 2021, **1**, vbab016.

15 J. Hu, F. Sarrami, H. Li, G. Zhang, K. A. Stubbs, E. Lacey, S. G. Stewart, A. Karton, A. M. Piggott and Y.-H. Chooi, *Chem Sci*, 2019, **10**, 1457–1465.

16 H. Yang, Z. Shang, Y. Chen, F. Li, K. Li, H. Zhu, M. Peng, J. Yang, C. Cai and J. Ju, *Org Lett*,2024.

17 Q. Ji, H. Xiang, W.-G. Wang and Y. Matsuda, *Angew. Chem. Int. Ed.*, 2024, **63**, e202402663.

18 S. Lu, J. Wang, F. Chitsaz, M. K. Derbyshire, R. C. Geer, N. R. Gonzales, M. Gwadz, D. I. Hurwitz, G. H. Marchler, J. S. Song, N. Thanki, R. A. Yamashita, M. Yang, D. Zhang, C. Zheng, C. J. Lanczycki and A. Marchler-Bauer, *Nucleic Acids Res*, 2020, **48**, D265–D268.

19 F. Madeira, N. Madhusoodanan, J. Lee, A. Eusebi, A. Niewielska, A. R. N. Tivey, R. Lopez and S. Butcher, *Nucleic Acids Res*, 2024, **52**, W521–W525.

20 V. Lefort, J.-E. Longueville and O. Gascuel, *Mol Biol Evol*, 2017, **34**, 2422–2424.

21 S. Guindon, J. F. Dufayard, V. Lefort, M. Anisimova, W. Hordijk and O. Gascuel, *Syst Biol*, 2010, **59**, 307–321.
